# Identification of Genomic Signatures in Bullmastiff Dogs Using Composite Selection Signals Analysis of 23 Purebred Clades

**DOI:** 10.3390/ani13071149

**Published:** 2023-03-24

**Authors:** Wei-Tse Hsu, Peter Williamson, Mehar Singh Khatkar

**Affiliations:** 1 Sydney School of Veterinary Science, Faculty of Science, The University of Sydney, Sydney, NSW 2006, Australia; 2 School of Animal and Veterinary Sciences, Faculty of Sciences, Engineering and Technology, The University of Adelaide, Roseworthy, SA 5371, Australia

**Keywords:** genome, canine, breed, selection, phenotype, diversity

## Abstract

**Simple Summary:**

Purebred dogs form distinct genetic subpopulations, and there are more than 400 of these recognized by breed clubs around worldwide. Their gene pool is limited by the number of dogs used to develop the breed and how the dogs have been bred. The total makeup of DNA in a breed determines the characteristics that we identify as typical of the breed. However, the limited genetic variation within a breed can also contribute to health issues arising from inherited faulty genes or because of complex interactions of many genes. Many studies have been completed in recent yearsso genetic information is now available for thousands of dogs. Together, this makes dog populations informative subjects for analysis. In this study, we applied a relatively new method which combines three ways to measure variation in the DNA of groups of animals, named F*_st_*, ∆DAF, and XP-EHH, into an index and applied it to the Bullmastiff breed. The method allows us to compare the genomic differences and similarities between groups of individuals from many breeds. We show that there are distinct regions of DNA that are specific to the modern Bullmastiff breed. By focusing on these DNA regions, we can understand some of the characteristics that define the breed and use the information to help us understand how some diseases may be more common in this breed.

**Abstract:**

Dog breeds represent canine sub-populations with distinctive phenotypic features and limited genetic diversity. We have established a resource to study breed-specific genetic diversity. Utilising genetic resources within our laboratory biobank, public domain genotype data and the phylogenetic framework of 23 breed clades, the primary objective for this study was to identify genomic regions that differentiate the Bullmastiff breed. Through application of a composite index analysis (CSS), genomic signatures were identified in Bullmastiffs when compared to the formative breeds, Mastiffs and Bulldogs, and to 22 other breed groups. Significant regions were identified on 15 chromosomes, with the most differentiated regions found on CFA1, CFA9, and CFA18. These regions may reflect genetic drift following establishment of the breed or the effects of selective breeding during development of the modern Bullmastiff. This was supported by analysis of genes from the identified genomic regions, including 458 genes from the multi-clade analysis, which revealed enriched pathways that may be related to characteristic traits and distinct morphology of the breed. The study demonstrates the utility of the CSS method in breed-specific genome analysis and advances our understanding of genetic diversity in Bullmastiff dogs.

## 1. Introduction

The domestic dog (*Canis familiaris*) is considered the first domesticated animal arising from interactions of their wild ancestors and humans. Over many centuries, dogs have been selected for an extensive range of phenotypes, including a wide range of body size, specific morphological traits (leg length, hair length, curl, texture, thickness, and tail and skull shape), coat color, and behavioural characteristics (herding, guarding, agility, speed, and companionship) [1,2]. There are now more than 400 breeds recognised by the different breed clubs, such as the Australian National Kennel Council (ANKC), American Kennel Club (AKC), United Kennel Club (UKC), The Kennel Club (KC) in the United Kingdom, or Fédération Cynologique Internationale (FCI). These clubs have established registration requirements and have developed breed standard guidelines [3,4]. Dogs from each breed are relatively genetically homogeneous, characterized by a high frequency of shared alleles and haplotypes with long range linkage disequilibrium. Because of their common ancestry, there is also a significant degree of shared haplotypes between breeds [5].

One group of breeds that has attracted some attention is the Mastiff-like dogs. Within this group are several breeds that share certain morphological features, including a characteristic skull shape described as brachycephalic, but they also exhibit a range of body sizes and susceptibility to some diseases. One example of this group is the Bullmastiff. This breed was derived by crossing Bulldogs (40%) with Mastiffs (60%) in Britain in the mid-19th century. The breed was popular with gamekeepers and was developed as a working dog to apprehend or ward off poachers [6]. A breeding program introduced by The Kennel Club in 1924 established the founders of the modern Bullmastiff purebred dogs. As with other breeds, the founder effect and subsequent selective breeding practises create distinctive genomic signatures. These genomic regions influence phenotype and may contribute to overall breed health. The Bullmastiff is characterised by its heavy musculature and a large square head supported by a muscular neck. It is considered a member of the working dog group in America and Britain, but in Australia, although originally imported from Europe, it is classified as a utility dog. 

We have established a Bullmastiff resource to study genetic diversity of the breed in Australia [6,7]. Utilising the genetic resources of the Bullmastiff biobank, public domain genotype data, and the phylogenetic framework of the 23 clades described by Parker et al. [5], the primary objective for this study was to identify and analyse distinctive genomic signatures in Australian Bullmastiffs. Detection of these signatures may be carried out by comparing genome scans of different breeds. The advantage of this approach is that it does not require detailed phenotyping. A number of statistical methods can be employed to detect such variation, including a multi-statistical index method referred to as combined selection signals (CSS) analysis, which was originally developed for application in farm animals and which we recently showed to be a sensitive test for use in dogs [8].

## 2. Material and Methods

### 2.1. Data Preparation

An in-house CanineHD BeadChip (Illumina Inc., San Diego, CA, USA) genotyping dataset was merged with those downloaded from previously published datasets derived from dogs from across the world. The resulting pooled dataset is summarised in Table 1. The in-house data (*n* = 747) were from four breeds: Bullmastiff, German Shepherd Dogs (GSD), Kelpie, and Border Collie [6,7,9,10,11,12,13]. Public domain data were available from the GEO database and previous studies, contributing an additional total of 7317 samples, representing a broad range of breeds (*n* = 250) [5,14,15,16,17,18,19]. 

Each of the datasets was generated independently, and they needed to be unified to address potential incompatibilities between genotypes arising from different sources, such as reference genome assemblies, labelling of X, Y, and MT chromosome, and the type of SNP identification used (GenBank, Broad Institute, or other database accession code, i.e., Chr_Pos or Chr.Pos). The standard maps of CanineHD BeadChip SNP markers (170K CanFam2, 170K CanFam3, and 220K CanFam3) were sourced from Illumina and applied to ensure that the different datasets were uniform. The SNPs that differed between genome assemblies (CanFam2 vs. CanFam3) were excluded. The genotypes assembled from different studies also employed different versions of the CanineHD BeadChip, with different strand orientations for different versions (TOP vs. BOTTOM) [25]. This was corrected using the function --flip SNPs in PLINK 1.9 [26] prior to merging. 

Five studies had only raw SNP outputs available (idat file format) generated by the Illumina Canine High-Density Beadchip (Illumina Inc., San Diego, CA, USA) and were downloaded from seven GEO database files. These raw data were processed with GenomeStudio 2.0 software (Illumina Inc., San Diego, CA, USA), and each dataset was fit into a given cluster. Standard canine cluster files were retrieved from Illumina with chromosome, position, and genotype for each SNP. The files (map/ped) containing the information used in PLINK for each SNP were obtained from the PLINK Input Report Plug-in v2.1.4 in GenomeStudio 2.0 software (Illumina Inc., San Diego, CA, USA).

The combined genotype dataset was assigned genomic chromosome and positions according to the CanFam 3.1 assembly. Further pruning was conducted in the PLINK 1.9 software to exclude samples with over 10% of missing data and SNPs with less than 0.01 minor allele frequency to give a final sample size of 151,901 SNPs across 38 autosomes in 8005 dogs representing 250 breeds. The high-quality SNP dataset was subset using PLINK v.1.9 [26] with the parameters --geno 0.1 --maf, 0.01 --not-chr 0 39 40 41 42, and --keep sample ID. The resulting data (Supplementary Table S1) were then used for further analysis. 

### 2.2. Phasing and Haplotype

The SNP array yields unphased genotypes. To generate the haplotype phase from the unphased genotype data, the HMM-based sampling approach implemented in the software Beagle 5.0 [27,28] (https://faculty.washington.edu/browning/beagle/beagle.html, accessed on 30 October 2020) was employed.

Prior to phasing, each panel was split by chromosome and converted to VCF format using PLINK v1.9. The VCF files were processed using the default settings (burnin = 3, iterations = 12, phased-states = 280, sliding windows = 40, overlap = 2, and no err parameter). The haplotype information obtained was applied in XP-EHH calculations. 

### 2.3. Composite Selection Signals (CSS) Analysis

The composite selection signals (CSS) approach was used in this analysis. CSS combines different test statistics to generate rank-based empirical values. In this CSS analysis, three constituent statistical tests (F*_st_*, ∆DAF, and XP-EHH) were used. The description of these tests is provided in the following sections. CSS analysis is comprised of several steps described as follows. First, ranking of the SNPs based on estimates from individual tests were obtained, and the ranking was scaled to a fractional ranking (between 0 and 1). Secondly, the fractional scores were transformed into z-scores with the inverse normal cumulative distribution function (CDF). The z-scores were assumed to follow a single normal distribution. Then, the mean of the z-scores from individual tests were obtained for each SNP. This mean z-score follows a normal distribution (0 ~m^−1^, m was the total number of test statistics) and was directly used to generate *p* values. The CSS value is defined as the logarithmic (−log_10_) transformed *p* value. To capture significant genomic regions and account for linked SNPs, the raw CSS scores were smoothed using mean values of CSS scores within a 0.5 Mb sliding window on both sides of each SNP. The top 0.005 fractions (0.5%) of smoothed CSS scores were considered as significant regions. The average CSS value was the mean of smoothed CSS values of all significant SNPs in the region [8].

### 2.4. Cross-Population Extended Haplotype Homozygosity (XP-EHH)

A haplotype-based method was taken into account as a component of the CSS analysis, and the scores of cross-population extended haplotype homozygosity (XP-EHH) were extracted using the SELSCAN package [29]. The required files were generated using the option --trunc-ok as true, which allowed more accurate XP-EHH computation than the default of false. The XP-EHH analyses were performed on pairwise comparisons of Bullmastiff against each of individual or the multiple breed clusters as the reference population. 

### 2.5. Fixation Index (F_st_) Analysis

The fixation index (F*_st_*) was first introduced 70 years ago [30] and subsequently developed to measure genetic differentiation. The most commonly used F*_st_* statistic presented by Weir and Cockerham [31] was applied to estimate the deviation of allele frequency between populations. A high F*_st_* value (0~1) for specific locus means high reproductive isolation levels (fixed) or strong positive selection in one of the populations. It has been shown to be suitable for detecting selection signatures using SNP array data. The F*_st_* scores for each SNP were computed by a pairwise comparison within all sets of the Bullmastiff target group and reference groups. 

### 2.6. Derived Allele Frequency (DAF)

Multiple studies have found evidence that the most modern breeds primarily share European ancestry [32,33]. Hence, 59 European Gray Wolves, which were genotyped on the same CanineHD BeadChip (Illumina, Inc., San Diego, CA, USA) from four population clusters, were used as an ancestral reference in the assessment of derived allele frequency [15,34]. Ancestral genotype data from a merged dataset were extracted by the PLINK --keep function, and the common ancestral variants were defined as monomorphic SNPs. These alleles were validated against representatives of the ancient breed group of Alaskan Huskies (*n* = 10) [35]. 

The derived allele frequency difference was computed depending on the following formula, ∆DAF = DAF_target_ − DAF_reference_. The wolf SNP information was used as ancestral. The ancestral SNPs were assigned for the DAF calculation. The normal distribution of derived allele frequency was estimated, and the ∆DAF values were transferred to Z scores (0,1). In the present study, the major alleles in wolves were assigned as ancestral alleles, following the approach used previously [8,15,36] and in comparison with data from 10 Alaskan Huskies as in a prior study [35]. The major alleles (common variants) from this dataset were assigned as ancestral alleles.

### 2.7. In Silico Functional Analysis of Candidate Genes

Annotated genes within significant genomic regions were identified and used for analyses. These genes were collated and annotated using RefGene (Ensembl) downloaded from the UCSC genome browser CanFam 3.1 (http://genome.ucsc.edu/, accessed on 9 April 2021). The list of genes was then used for functional enrichment analysis. The Database for Annotation, Visualisation and Integrated Discovery (DAVID) v6.8 was used for analysing functional classification, gene ontology, pathway involvement, and for understanding high-level functions and contribution to biological systems from a large-scale molecular dataset (http://david.abcc.ncifcrf.gov/, accessed on 13 September 2021) [37,38] via the Kyoto Encyclopedia of Genes and Genomes (KEGG, https://www.kegg.jp/, accessed on 13 September 2021) and Gene Ontology (GO, http://geneontology.org/, accessed on 13 September 2021) knowledge base database resources. The Benjamini-adjusted *p*-value ≤ 0.05 and FDR ≤ 0.05 were used as thresholds for declaring statistically significant differences.

Visualisation by Cytoscape software (Version 3.8.2) [39] with analysis using the plug-in ClueGO v2.2.8/CluePedia v1.5.8 [40,41] was used to investigate whether identified genes were biologically interconnected. Three independent ontologies, biological process (BP), molecular function (MF) and cellular component (CC) categories, and pathway analysis (KEGG) were captured to construct the integrated network. The network selection parameters applied were hypergeometric tests: two-sided adjusted *p* ≤ 0.05 and kappa score ≥ 0.4.

## 3. Results

**Figure 1:**
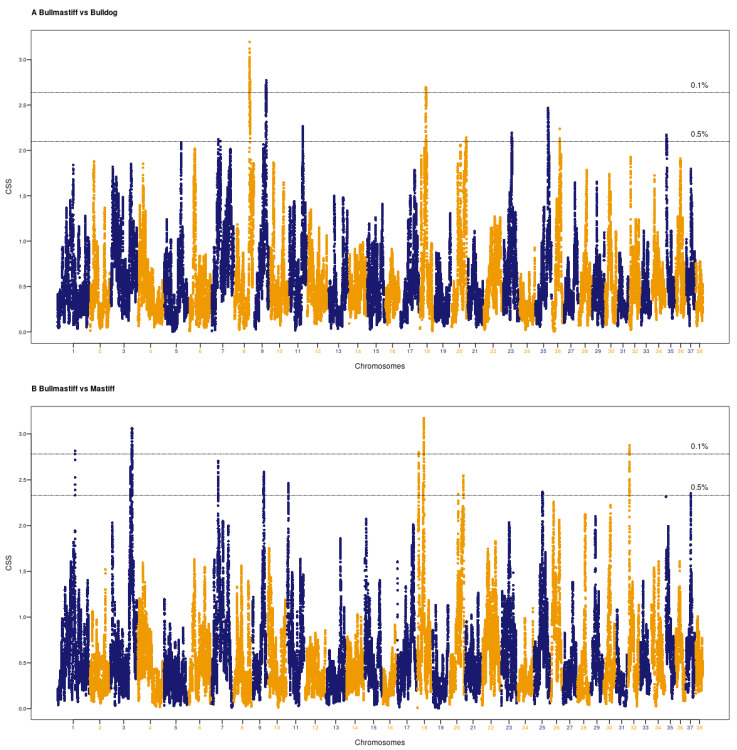
Genomic signals in Bullmastiff dogs detected using the CSS method. The CSS results of the Bullmastiff vs. Bulldog pairwise comparison is shown in the upper Manhattan plot (**A**), and the results of the Bullmastiff vs. Mastiff pairwise comparison is shown in the lower Manhattan plot (**B**). The x-axis represents the 38 autosomes shown in alternate orange and blue color, and the y-axis represents the mean value of CSS scores (F*_st_*, ∆DAF and XP-EHH) in a 1 Mb window size. The horizontal lines are the thresholds for 0.1% and 0.5% CSS value, respectively.

A total of 26 distinctive peaks were found from two breed pair comparisons (Bullmastiffs vs. Mastiff and Bullmastiffs vs. Bulldogs) scanned using a 1 Mb sliding window CSS analysis. These were found on chromosomes CFA1, CFA3, CFA7, CFA8, CFA9, CFA11, CFA18, CFA20, CFA23, CFA25, CFA26, CFA32, CFA35, and CFA37, with CSS values ranging from 2.10 to 2.81 (Table 2). Out of these, 14 regions were detected from the analysis of Bullmastiffs vs. Mastiffs, and 12 regions were detected from the Bullmastiffs vs. Bulldogs comparison. The highest signal (CSS value 2.81) was identified for a region on chromosome 18 (CFA18:29.32–31.85 Mb) in the pairwise comparison of Bullmastiff and Mastiff. This region spanned 113 SNPs and 46 genes (5snoRNA, 21 lncRNA, and 20 protein coding). The region with the highest peak (CSS value 2.57) for the Bullmastiff vs. Bulldog analysis was located at CFA8:56.55–60.35 Mb and included 231 SNPs and 34 genes (four snRNAs, one pseudogene, 13 lncRNAs, and 16 protein coding). None of these regions were found in both comparisons, suggesting that they were derived from the original founder animals. There were four regions found in both comparisons, on CFA7, CFA9, CFA18, and CFA20. These regions are likely to have been selected during breeding of the modern Australian Bullmastiff. The distribution of CSS values for both comparisons is shown in Figure 1.

Further analysis was conducted based on the genes within the identified regions. A total of 2681 significant SNPs were identified for the comparison of Bullmastiff with the two formative breeds (Mastiff and Bulldog). These SNPs were mapped to 487 genes from the pairwise comparison of Bullmastiff and Mastiff and 415 genes for the Bullmastiff/Bulldog comparison (Supplementary Table S2). Gene-set and pathway analysis using DAVID software tools classified the genes into a total of 120 and 163 GO terms (biology process, molecular function, and cellular component) and 10 and six KEGG pathways, respectively (Supplementary Table S2). The genes identified from the two datasets were also analysed using ClueGo software (ver.2.5.0) for enrichment and network analysis. Genes were classified into 42 enrichment groups, shown in Supplementary Table S3. The resulting network is shown in Supplementary Figure S1. The analyses highlight metabolic and growth-related gene pathways and networks captured within significant regions. One notable example is the muscle hypertrophy classification, a pathway that may underlie the highly developed musculature of Bullmastiffs. A genome-wide genotype comparison of Bullmastiffs and dogs within each of the clades defined by Parker et al. [5] revealed consistent distinguishing signatures for the Bullmastiff breed. First, an analysis of 141,302 SNPs from Bullmastiffs and the breeds defined in the previous study as European Mastiff (minus Bullmastiff data) were used for CSS calculations. A total of 689 significant SNPs were located within nine significant regions (Table 3). The genes within these regions were retrieved for in silico functional analysis (Supplementary Table S4).

Six of these significant regions overlapped with the results described in the previous section (CFA8: 56.71–59.88 Mb, CFA9: 49.8–51.77 Mb, CFA18: 26.41–29.93 Mb, 30.02–31.88 Mb, CFA20: 53.16–55.75 Mb, and CFA25: 40.71–41.88 Mb). When both comparisons were collated, there were 490 genes (29 snRNA, eight pseudogenes, 358 protein coding, seven miRNAs, and 88 lncRNAs) within significant regions that were immediately flanking. A gene-set and pathway analysis of these genes using DAVID captured a total of 144 GO terms (biology process, molecular functions, and cellular component) and four KEGG pathways (Supplementary Table S5). Further analysis of the genes was conducted using ClueGo software (ver.2.5.0) to build an integrated network based on enriched functional classifications. The network is shown in Supplementary Figure S2. The genes were classified into 27 groups, with the most significant terms of each group shown in Supplementary Table S6. Interestingly, pathways related to inflammation and cancer are featured. Bullmastiffs are known to have an increased prevalence of cancer and are susceptible to inflammatory conditions.

**Figure 2:**
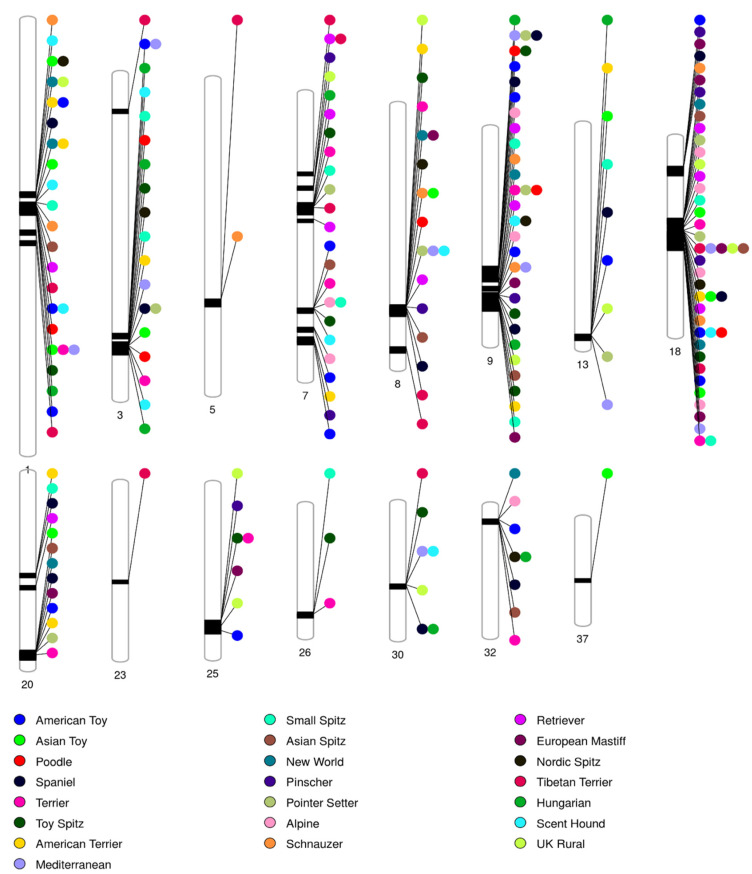
An ideogram of significant genomic regions identified from comparisons between the Bullmastiff and 22 breed clades. PhenoGram software was used to plot significant regions across chromosomes. Lines are plotted on the chromosomes corresponding to the base–pair location of each SNP. The color of circles represents the reference groups.

Dog breeds have previously been grouped into clades based on genomic analysis [5,42]. Genotypes from Bullmastiffs were compared to representative samples of breeds from each of these clades to investigate the most prominent genomic signatures. Pairwise genome-wide scans of Bullmastiffs compared dogs from the 22 clades represented in the combined genotype dataset (described in Materials and Methods) were analysed. The details of each pair-wise analysis are shown in the form of Manhattan plots in Supplementary Files (Figures S4–S25). To simplify these complex analyses, a summary of the results showing significant genomic regions is presented as a phenogram in Figure 2. When considering all comparisons, distinct peaks identifying consistently significant regions in Bullmastiffs were found on chromosomes CFA1, CFA3, CFA5, CFA7, CFA8, CFA9, CFA13, CFA18, CFA20, CFA23, CFA25, CFA26, CFA30, CFA32, and CFA37. Particularly noteworthy were regions on CFA9 and CFA18 where the Bullmastiff genotypes were distinguished from a majority of other breeds.

Genes (*n* = 458) found within these regions and associated annotations are provided in Supplementary Data (Table S6). Gene-set and pathway analyses identified a total of 275 GO terms (biology process, molecular function, and cellular component) and 16 KEGG pathways for genes within the identified regions and those immediately flanking from the pairwise comparisons across all clades (Supplementary Table S6). The genes were subjected to enrichment analysis using ClueGo software (ver.2.5.0), and an enrichment network was constructed. The network is shown in Supplementary Figure S3. The genes were classified into 41 groups, with the significant annotations for each group listed in Supplementary Table S7. The largest groups of genes were classified as being involved in metabolic processes and immune cell function, especially cell migration pathways involving chemokines. One other interesting group contained several micro RNAs (MiR) that have been implicated in cancer.

## 4. Discussion

Modern dog breeds were created by humans through crossing and selection according to breeding schemes and standards. Guidelines have been developed around specific traits for each breed, including desired behavioural traits, morphological characteristics, or the ability to learn and perform different tasks [42,43]. As a result of the origins and selection processes, modern purebred dogs have limited genetic heterogeneity and distinct genomic signatures that underlie their characteristic traits. In this study, the CSS test was used to identify genomic signatures in multiple-breed comparisons with a focus on the Bullmastiff breed. CSS combines three commonly used test statistics into complementary signals so that regions harbouring common signals can be identified with high sensitivity [8,44,45,46,47].

Bullmastiffs were originally created by crossing dogs from the Mastiff and Bulldog breeds to produce a breed that was intermediate in size and temperament. The Bulldog can be identified by its large head with wedge-shaped body, as well as short and folded ears. Other morphological features of Bulldogs include a stocky build with deep furrows of the skin and face, short or corkscrew tail, short thick legs with equally broad paws, and a moderate temperament. Relatively, Mastiffs have a more placid temperament, and are one of the largest and heaviest of dog breeds, weighing up to 100 kg [48]. The strong founder effect and relatively recent origins of the breed mean that Bullmastiff dogs share relatively long haplotypes with Mastiffs and Bulldogs, and as expected, they are classified within the same clade [5,6]. However, selective breeding and genetic drift since formation of the breed has left distinct signatures in the Bullmastiff genome. Indeed, through CSS analyses, notable genomic regions were identified in Bullmastiffs when compared to Mastiffs and Bulldogs. Although the analysis is designed to detect distinguishing genomic regions rather than specific functional variants, the regions are likely to contain some variants that have contributed to breed development or characteristic traits. Some examples of genes of interest within significant regions included the *COL5A1* (Collagen alpha-1(V) chain) and *ADAMTSL2* (ADAMTS-like protein 2) on CFA9 (48.92–51.87 Mb). Collagen is a key structural protein of the connective tissue including skin and supports the connective tissue matrix to maintain shape, strength, and the ability to resist deformation [49]. Genetic variants in the *COL5A1* gene are associated with loose skin, and in extreme cases, with a clinical presentation of Ehlers–Danlos syndromes (EDS) in dogs [49]. *ADAMTSL2* is a secreted extracellular matrix protein. Variants in this gene have been associated with the hereditary disorder Musladin-Lueke Syndrome (MLS) in Beagles, characterised by muscle and skin fibrosis leading to stiff skin and joint contractures [50]. In humans and mice, elevated levels of *ADAMTSL2* are seen in cardiomyopathies [51]. Bullmastiffs are known to have an elevated risk of cardiomyopathy.

The genome-wide scan for the pairwise comparison of Bullmastiff and Mastiff identified a region on CFA1 (62.56–64.02 Mb), which includes the *NKAIN2* (Sodium/potassium-transporting ATPase subunit beta-1-interacting protein 2) gene. This gene plays a role in hair hypopigmentation, craniofacial and limb formation, eye development, and macrocephaly [52]. Similarly, a prominent region on CFA11 (13.25–14.57 Mb) contains the *PRDM8* (PR domain zinc finger protein 8) gene. *PRDM8* has been linked to coat length in dogs [43,53]. This region also contained the *ZNF608* (Zinc Finger Protein 608) gene, which is associated with body mass in dogs [24]. A significant region on CFA25 (27.99–29.17 Mb) was adjacent to the *MSRA* (peptide-methionine (S)-S-oxide reductase) gene. This gene is involved in methionine metabolism and repair of oxidative damage of proteins. It has been associated with adiposity and fat distribution in humans [54], and through whole-genome selection scans and GWAS, with fat deposition and hair growth in other species [55]. Bullmastiffs are generally lean and muscular, but they are prone to obesity if not carefully managed.

A region identified on CFA32 (3.99–5.7 Mb) overlapped growth-related genes that may be associated with morphological traits. *BMP3* (bone morphogenetic protein 3) affects bone growth and development and is associated with skull morphology. The *FGF5* (Fibroblast growth factor 5) gene and *ANTXR2* (anthrax toxin receptor-2) are associated with coat length in dogs. The *PRKG2* (cGMP-dependent protein kinase) and *RASGEF1B* (RasGEF domain family member 1B) genes have been identified as positional candidate genes for growth restriction, aggression, self-injurious behaviours, and mental retardation in affected German Shepherd dogs [43].

Not surprisingly, the results of pairwise comparison of Bullmastiff with a combined reference group consisting of all other breeds in the European Mastiff clade showed substantial overlap with the comparison to Bulldog and Mastiff breeds. Some regions were consistently present in Bullmastiffs, but there were no outstanding gene candidates that could be associated with breed-specific morphological variation, which may require comparison of extreme phenotypes [24]. Instead, similar sets of GO terms and KEGG pathways were identified in this dataset, suggesting that the process of Bullmastiff formation from the Bulldog x Mastiff cross affected the same body systems that had been subject to selection at the onset of original breed formation. However, some functional categories from the gene-set analysis were of interest and related to the adipocytokine signaling pathway (cfa04920) and the PPAR signaling pathway (cfa03320). Pathway-based annotation indicates that the adipo-cytokine signaling pathway and PPAR signaling pathway are significantly correlated with BMI and fat mass, suggesting that these pathways may play a role in the weight and body mass characteristics of the breed [56,57,58].

The region identified on CFA32, mentioned previously, and containing *BMP3* and other genes, was highlighted in many of the pairwise comparisons of Bullmastiffs and dogs from 22 non-Mastiff clades. There were also a number of commonly found enriched gene pathways and GO categories evident in many comparisons, e.g., those associated with neurological, nervous and immune system development, metabolism, and organ growth. This points to the potential of early developmental pathways that may have broad phenotypic effects. As recently highlighted, variation in many of these fundamental pathways may have arisen prior to modern breed formation and arisen from selection pressures on ancestral populations [59]. A good example of this are the neural crest pathways. A role for the neural crest in dog domestication has gained support from researchers over recent years [52]. Neural crest cells (NCCs) are multipotent, transient, embryonic stem cells that are initially located at the crest (or dorsal border) of the neural tube. Regulation of neural crest development requires a network of genes expressed early in embryogenesis that coordinate a multi-stage process, resulting in migration of NCCs to various sites in the developing embryo where they differentiate into a diverse array of cell types [52]. Several of the pathways associated with genomic regions identified in this study are involved in this network, supporting the view that variants in key genes that activate the neural crest and define the migration gates for NCCs contribute to diversity in domestic dog breeds.

## 5. Conclusions

This study demonstrates the utility of applying the composite index method, CSS, to identify genomic signatures in purebred dogs. The results define regions containing variants that are found at higher frequency in the Bullmastiff breed when compared to other dog breeds. Analysis of annotated genes and related pathways found within these regions contributes to understanding diversity in the breed and may underpin further studies of breed health and disease.

## Figures and Tables

**Table 1:** The number of SNPs and dog samples in various datasets. A combined dataset of genotypes from 8006 dogs, representing 250 breeds, were obtained by merging public domain and in-house datasets.

Study	Sample (*n*)	Variants	Reference Genome Assemblies	SNP Converted to CanFam3
Parker et al. [5]	1355	154,277	CanFam3	154,277
Choi et al. [14]	192	173,662	CanFam2	173,662
Hayward et al. [16]	4342	160,727	CanFam3	154,432
Vaysse et al. [18]	547	174,810	CanFam2	172,107
Tonomura et al. [17]	356	173,662	CanFam2	172,107
Yang et al. [19]	170	173,662	CanFam3	173,662
In-house dataset [6,7,9,10,11,12]	339	172,939	CanFam2 or CanFam3	172,246
	408	218,711	CanFam3	176,008
Data retrieved from GEO website (http://www.ncbi.nlm.nih.gov/geo/, accessed on 25 February 2019)				
GSE83225 [20], GSE83151 [20], GSE83154 [20], GSE66677 [21], GSE73400 [22], GSE87642 [23], GSE123368 [24]	296	150,704	CanFam3	150,704
Source for Ancestral population				
Fitak et al. [15]	59	131,118	CanFam2	131,118

**Table 2:** Summary of significant regions identified in Bullmastiff dogs using Bulldog or Mastiff as reference populations.

Bullmastiff vs. Bulldog					Bullmastiff vs. Mastiff				
Chromosome	Region	Average of CSS	Number of Significant SNPs	Number of SNPs	Chromosome	Region	Average of CSS	Number of Significant SNPs	Number of SNPs
7	26.64–27.47 Mb	2.11	38	38	1	62.56–64.02 Mb	2.52	36	47
	36.13–37.07 Mb	2.1	52	56	3	73.14–74.6 Mb	2.49	96	107
8	56.55–60.35 Mb	2.57	231	255		76.9–79.62 Mb	2.68	194	212
9	48.92–51.87 Mb	2.53	212	226	7	25.92–27.31 Mb	2.49	71	71
11	62.98–64.67 Mb	2.18	121	128	9	49.31–51.54 Mb	2.47	153	163
18	27.03–30.61 Mb	2.43	204	223	11	13.25–14.57 Mb	2.4	67	67
20	54.43–55.48 Mb	2.11	67	69	18	8.02–9.84 Mb	2.51	99	101
23	27.58–28.85 Mb	2.14	101	113		27.22–28.51 Mb	2.39	70	81
	28.96–29.99 Mb	2.11	54	62		29.32–31.85 Mb	2.87	133	139
25	40.51–43.58 Mb	2.25	213	229	20	29.92–30.89 Mb	2.34	38	46
26	28.59–29.13 Mb	2.15	30	35		54.45–55.8 Mb	2.46	77	79
35	0.84–2.19 Mb	2.14	82	82	25	27.99–29.17 Mb	2.35	90	95
					32	3.99–5.7 Mb	2.56	99	107
					37	17.81–18.77 Mb	2.35	53	59

**Table 3:** Summary of significant regions identified in the comparison of Bullmastiff and other breeds within the European Mastiff clade.

Chromosome	Region	Average of CSS	Number of SNPs	Number of Significant SNPs
8	56.71–59.88 Mb	2.61	228	163
9	47.15–48.6 Mb	2.23	116	35
	49.8–51.77 Mb	2.42	165	82
18	8.08–9.56 Mb	2.29	91	34
	22.69–25.69 Mb	2.57	188	77
	26.41–29.93 Mb	2.87	239	158
	30.02–31.88 Mb	2.29	149	45
20	53.16–55.75 Mb	2.23	195	78
25	40.71–41.88 Mb	2.22	89	17

## Data Availability

Data from this study is available within this publication and Supplementary Material or in cited sources.

## Supplementary Materials

The following supporting information can be downloaded at: https://www.mdpi.com/article/10.3390/ani13071149/s1. Supplementary Table S1. Summary of the clade, breed, sample size, genotype data of the populations used as reference populations. Bullmastiff dogs (*n* = 538) were used as the target group. Supplementary Table S2 The list of GO terms, KEGG pathways and the associated genes in either of two pairwise comparisons (Bullmastiff vs. Bulldog and Bullmastiff vs. Mastiff). Supplementary Table S3. The list of significant GO terms, KEGG pathways and the associated genes in either of two pairwise comparisons (Bullmastiff vs. Bulldog and Bullmastiff vs. Mastiff). Supplementary Table S4 Summary of significant GO terms, KEGG pathways and involved genes for pairwise comparison of Bullmastiff vs. European Mastiff reference clade. Supplementary Table S5. Overall view of functional classification of candidate gene in Bullmastiffs compared to other breeds within the European Mastiff clade by GO terms and KEGG pathways. Supplementary Table S6. Overall view of functional classification of candidate gene in Bullmastiffs compared across all clades by GO terms and KEGG pathways. Supplementary Table S7. Summary of combined significant GO terms, KEGG pathways and involved genes for pairwise comparison of Bullmastiff vs. each clade. Supplementary Figure S1. KEGG pathway and GO term analyses of genes in the selected regions in the Bullmastiff when compared with Bulldog and Mastiff population. Supplementary Figure S2. KEGG pathway and GO term analysis of genes for the Bullmastiff compared to other breeds within the European Mastiff clade. Supplementary Figure S3. Overview of KEGG pathway and GO term analysis of candidate genes found from the Bullmastiff pairwise comparisons with multiple clades. Supplementary Figure S4. Genomic signals detected in Bullmastiff dogs compared to Asian Spitz groups using the CSS method. Supplementary Figure S5. Genomic signals detected in Bullmastiff dogs compared to Asian Toy groups using the CSS method. Supplementary Figure S6. Genomic signals detected in Bullmastiff dogs compared to Tibetan Terrier groups using the CSS method. Supplementary Figure S7. Genomic signals detected in Bullmastiff dogs compared to Nordic Spitz groups using the CSS method. Supplementary Figure S8. Genomic signals detected in Bullmastiff dogs compared to Schnauzer groups using the CSS method. Supplementary Figure S9. Genomic signals detected in Bullmastiff dogs compared to Small Spitz groups using the CSS method. Supplementary Figure S10. Genomic signals detected in Bullmastiff dogs compared to Toy Spitz groups using the CSS method. Supplementary Figure S11. Genomic signals detected in Bullmastiff dogs compared to Hungarian groups using the CSS method. Supplementary Figure S12. Genomic signals detected in Bullmastiff dogs compared to Poodle group using the CSS method. Supplementary Figure S13. Genomic signals detected in Bullmastiff dogs compared to American Toy groups using the CSS method. Supplementary Figure S14. Genomic signals detected in Bullmastiff dogs compared to American Terrier groups using the CSS method. Supplementary Figure S15. Genomic signals detected in Bullmastiff dogs compared to Pinscher groups using the CSS method. Supplementary Figure S16. Genomic signals detected in Bullmastiff dogs compared to Terrier using the CSS method. Supplementary Figure S17. Genomic signals detected in Bullmastiff dogs compared to New World groups using the CSS method. Supplementary Figure S18. Genomic signals detected in Bullmastiff dogs compared to Mediterranean groups using the CSS method. Supplementary Figure S19. Genomic signals detected in Bullmastiff dogs compared to Scent Hound groups using the CSS method. Supplementary Figure S20. Genomic signals detected in Bullmastiff dogs compared to Spaniel groups using the CSS method. Supplementary Figure S21. Genomic signals detected in Bullmastiff dogs compared to Retriever groups using the CSS method. Supplementary Figure S22. Genomic signals detected in Bullmastiff dogs compared to Pointer Setter groups using the CSS method. Supplementary Figure S23. Genomic signals detected in Bullmastiff dogs compared to UK Rural groups using the CSS method. Supplementary Figure S24. Genomic signals detected in Bullmastiff dogs compared to Alpine groups using the CSS method. Supplementary Figure S25. Genomic signals detected in Bullmastiff dogs compared to European Mastiff groups using the CSS method.
