# Supplementary material for: Identification of Genomic Signatures in Bullmastiff Dogs Using Composite Selection Signals Analysis of 23 Purebred Clades
Source: Animals (Basel). 2023 Mar 24;13(7):1149. doi: 10.3390/ani13071149 (PMC10093657; doi:10.3390/ani13071149)
Supplement: Supplementary file 1 [file animals-13-01149-s001.zip › animals-2281639-supplementary.pdf]

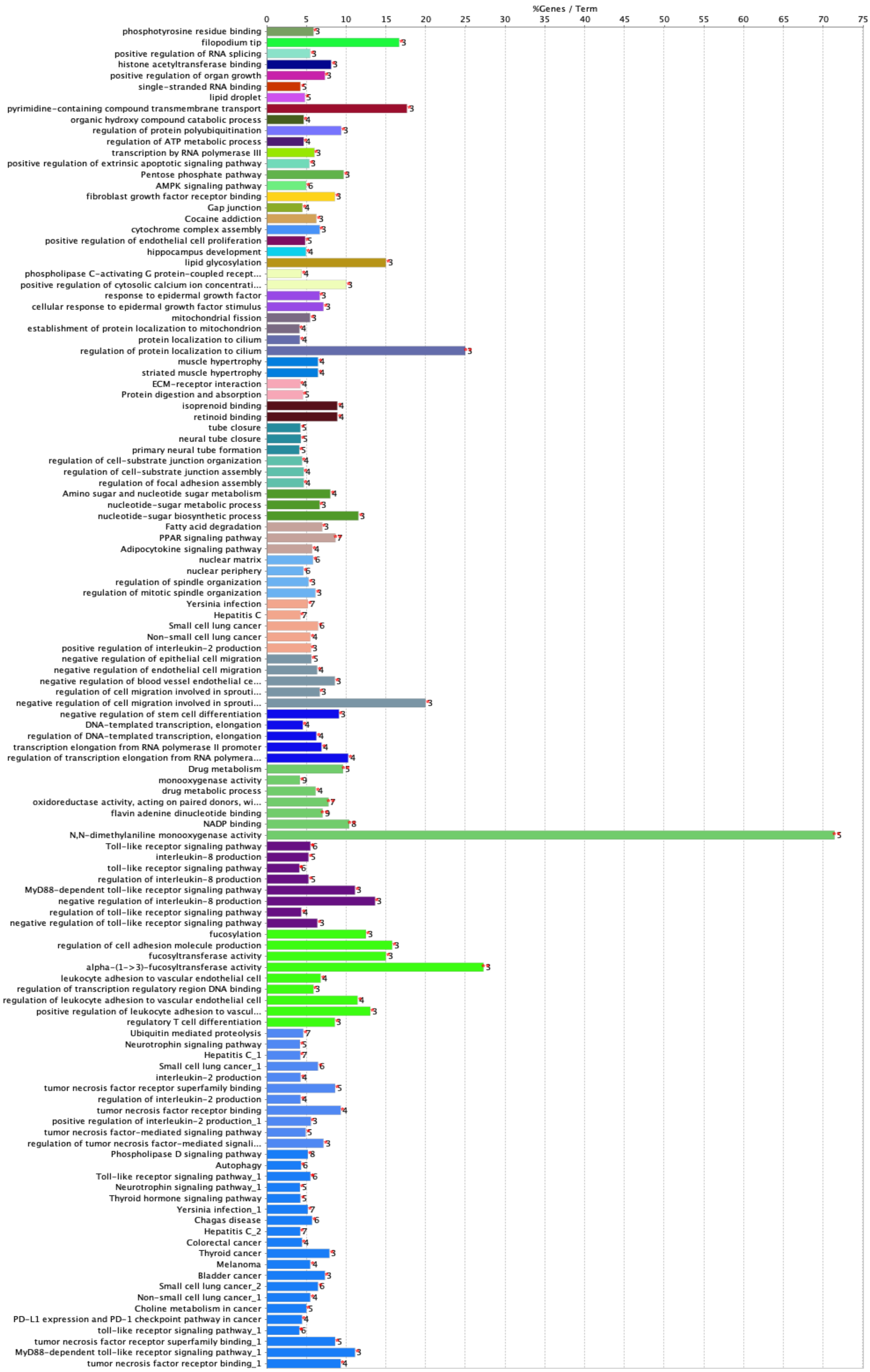

%Genes / Term
0
5
10
15
20
25
30
35
40
45
50
55
60
65
70
75
phosphotyrosine residue binding
filopodium tip
positive regulation of RNA splicing
histone acetyltransferase binding
positive regulation of organ growth
single-stranded RNA binding
lipid droplet
pyrimidine-containing compound transmembrane transport
organic hydroxy compound catabolic process
regulation of protein polyubiquitination
regulation of ATP metabolic process
transcription by RNA polymerase III
positive regulation of extrinsic apoptotic signaling pathway
Pentose phosphate pathway
AMPK signaling pathway
fibroblast growth factor receptor binding
Gap junction
Cocaine addiction
cytochrome complex assembly
positive regulation of endothelial cell proliferation
hippocampus development
lipid glycosylation
phospholipase C-activating G protein-coupled recept...
positive regulation of cytosolic calcium ion concentrati...
response to epidermal growth factor
cellular response to epidermal growth factor stimulus
mitochondrial fission
establishment of protein localization to mitochondrion
protein localization to cilium
regulation of protein localization to cilium
muscle hypertrophy
striated muscle hypertrophy
ECM-receptor interaction
Protein digestion and absorption
isoprenoid binding
retinoid binding
tube closure
neural tube closure
primary neural tube formation
regulation of cell-substrate junction organization
regulation of cell-substrate junction assembly
regulation of focal adhesion assembly
Amino sugar and nucleotide sugar metabolism
nucleotide-sugar metabolic process
nucleotide-sugar biosynthetic process
Fatty acid degradation
PPAR signaling pathway
Adipocytokine signaling pathway
nuclear matrix
nuclear periphery
regulation of spindle organization
regulation of mitotic spindle organization
Yersinia infection
Hepatitis C
Small cell lung cancer
Non-small cell lung cancer
positive regulation of interleukin-2 production
negative regulation of epithelial cell migration
negative regulation of endothelial cell migration
negative regulation of blood vessel endothelial ce...
regulation of cell migration involved in sprouti...
negative regulation of cell migration involved in sprouti...
negative regulation of stem cell differentiation
DNA-templated transcription, elongation
regulation of DNA-templated transcription, elongation
transcription elongation from RNA polymerase II promoter
regulation of transcription elongation from RNA polymera...
Drug metabolism
monooxygenase activity
drug metabolic process
oxidoreductase activity, acting on paired donors, wi...
flavin adenine dinucleotide binding
NADP binding
N,N-dimethylaniline monooxygenase activity
Toll-like receptor signaling pathway
interleukin-8 production
toll-like receptor signaling pathway
regulation of interleukin-8 production
MyD88-dependent toll-like receptor signaling pathway
negative regulation of interleukin-8 production
regulation of toll-like receptor signaling pathway
negative regulation of toll-like receptor signaling pathway
fucosylation
regulation of cell adhesion molecule production
fucosyltransferase activity
alpha-(1->3)-fucosyltransferase activity
leukocyte adhesion to vascular endothelial cell
regulation of transcription regulatory region DNA binding
regulation of leukocyte adhesion to vascular endothelial cell
positive regulation of leukocyte adhesion to vascul...
regulatory T cell differentiation
Ubiquitin mediated proteolysis
Neurotrophin signaling pathway
Hepatitis C_1
Small cell lung cancer_1
interleukin-2 production
tumor necrosis factor receptor superfamily binding
regulation of interleukin-2 production
tumor necrosis factor receptor binding
positive regulation of interleukin-2 production_1
tumor necrosis factor-mediated signaling pathway
regulation of tumor necrosis factor-mediated signali...
Phospholipase D signaling pathway
Autophagy
Toll-like receptor signaling pathway_1
Neurotrophin signaling pathway_1
Thyroid hormone signaling pathway
Yersinia infection_1
Chagas disease
Hepatitis C_2
Colorectal cancer
Thyroid cancer
Melanoma
Bladder cancer
Small cell lung cancer_2
Non-small cell lung cancer_1
Choline metabolism in cancer
PD-L1 expression and PD-1 checkpoint pathway in cancer
toll-like receptor signaling pathway_1
tumor necrosis factor receptor superfamily binding_1
MyD88-dependent toll-like receptor signaling pathway_1
tumor necrosis factor receptor binding_1

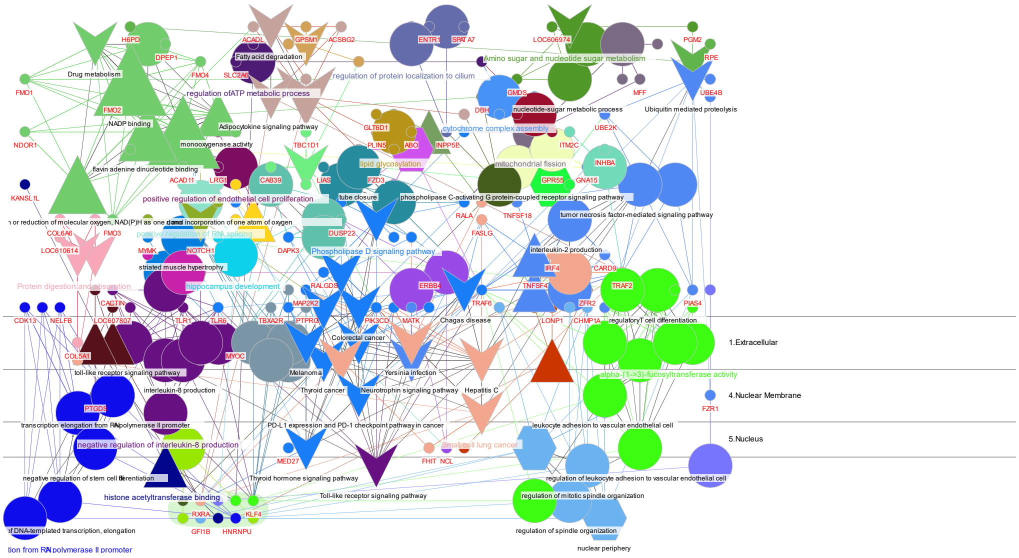

Supplementary Figure S1. KEGG pathway and GO term analyses of genes in the selected regions in the Bullmastiff when compared with Bulldog and Mastiff population.

The bar chart shows the different GO terms. KEGG pathways are grouped according to their similarities. The number of genes is shown next to each GO term and KEGG pathway category. The percentage (%) of genes found from the total number of associated genes. Visualization of the network constructed from GO term and KEGG pathways was carried out using ClueGO in Cytoscape with kappa score threshold level ≥0.4. Only the most significant terms in the group are labelled.

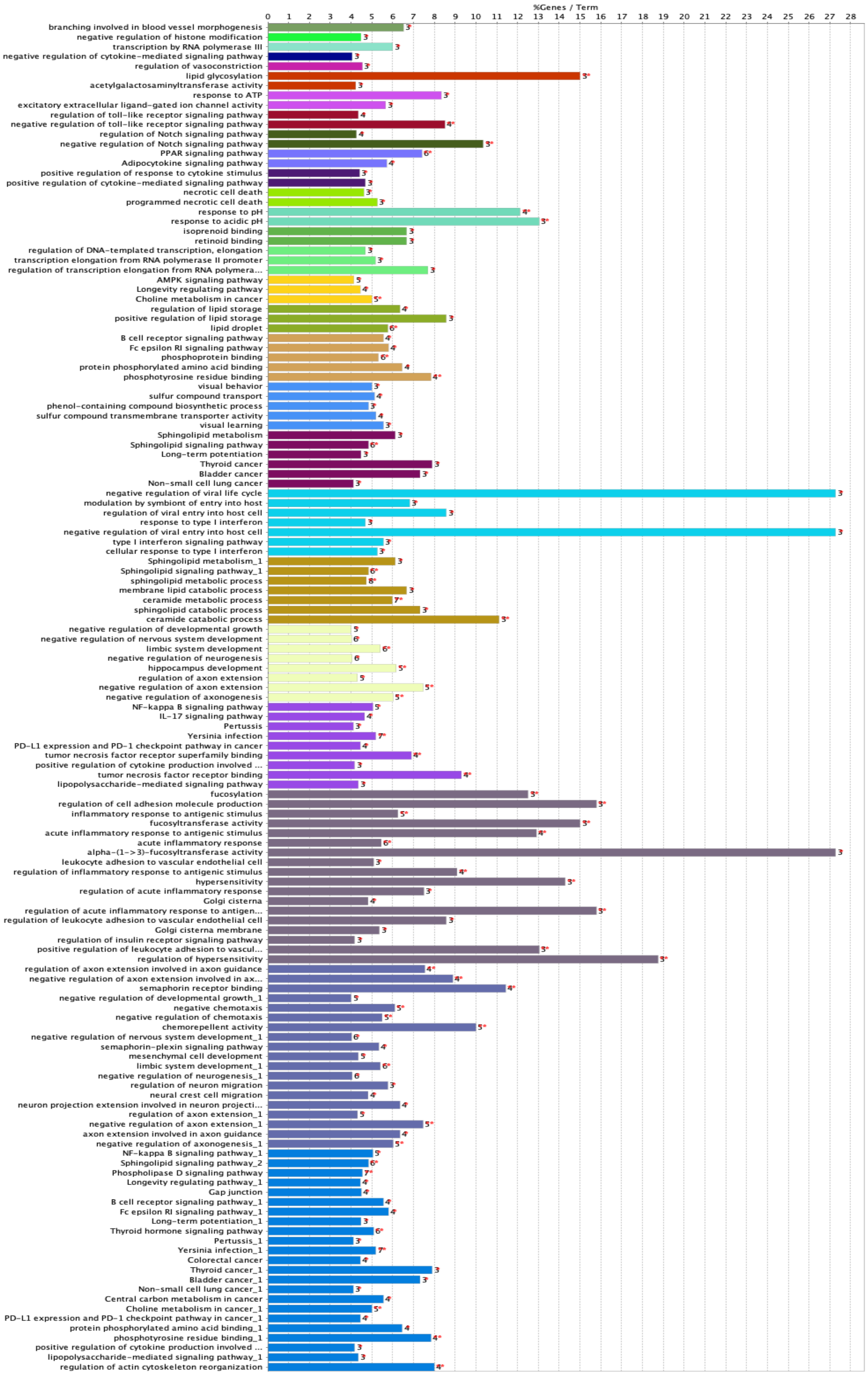
%Genes / Term
0 1 2 3 4 5 6 7 8 9 10 11 12 13 14 15 16 17 18 19 20 21 22 23 24 25 26 27 28
branching involved in blood vessel morphogenesis
negative regulation of histone modification
transcription by RNA polymerase III
negative regulation of cytokine-mediated signaling pathway
regulation of vasoconstriction
lipid glycosylation
acetylgalactosaminyltransferase activity
response to ATP
excitatory extracellular ligand-gated ion channel activity
regulation of toll-like receptor signaling pathway
negative regulation of toll-like receptor signaling pathway
regulation of Notch signaling pathway
negative regulation of Notch signaling pathway
PPAR signaling pathway
Adipocytokine signaling pathway
positive regulation of response to cytokine stimulus
positive regulation of cytokine-mediated signaling pathway
necrotic cell death
programmed necrotic cell death
response to pH
response to acidic pH
isoprenoid binding
retinoid binding
regulation of DNA-templated transcription, elongation
transcription elongation from RNA polymerase II promoter
regulation of transcription elongation from RNA polymera...
AMPK signaling pathway
Longevity regulating pathway
Choline metabolism in cancer
regulation of lipid storage
positive regulation of lipid storage
lipid droplet
B cell receptor signaling pathway
Fc epsilon RI signaling pathway
phosphoprotein binding
protein phosphorylated amino acid binding
phosphotyrosine residue binding
visual behavior
sulfur compound transport
phenol-containing compound biosynthetic process
sulfur compound transmembrane transporter activity
visual learning
Sphingolipid metabolism
Sphingolipid signaling pathway
Long-term potentiation
Thyroid cancer
Bladder cancer
Non-small cell lung cancer
negative regulation of viral life cycle
modulation by symbiont of entry into host
regulation of viral entry into host cell
response to type I interferon
negative regulation of viral entry into host cell
type I interferon signaling pathway
cellular response to type I interferon
Sphingolipid metabolism_1
Sphingolipid signaling pathway_1
sphingolipid metabolic process
membrane lipid catabolic process
ceramide metabolic process
sphingolipid catabolic process
ceramide catabolic process
negative regulation of developmental growth
negative regulation of nervous system development
limbic system development
negative regulation of neurogenesis
hippocampus development
regulation of axon extension
negative regulation of axon extension
negative regulation of axonogenesis
NF-kappa B signaling pathway
IL-17 signaling pathway
Pertussis
Yersinia infection
PD-L1 expression and PD-1 checkpoint pathway in cancer
tumor necrosis factor receptor superfamily binding
positive regulation of cytokine production involved ...
tumor necrosis factor receptor binding
lipopolysaccharide-mediated signaling pathway
fucosylation
regulation of cell adhesion molecule production
inflammatory response to antigenic stimulus
fucosyltransferase activity
acute inflammatory response to antigenic stimulus
acute inflammatory response
alpha-(1->3)-fucosyltransferase activity
leukocyte adhesion to vascular endothelial cell
regulation of inflammatory response to antigenic stimulus
hypersensitivity
regulation of acute inflammatory response
Golgi cisterna
regulation of acute inflammatory response to antigen...
regulation of leukocyte adhesion to vascular endothelial cell
Golgi cisterna membrane
regulation of insulin receptor signaling pathway
positive regulation of leukocyte adhesion to vascul...
regulation of hypersensitivity
regulation of axon extension involved in axon guidance
negative regulation of axon extension involved in ax...
semaphorin receptor binding
negative regulation of developmental growth_1
negative chemotaxis
negative regulation of chemotaxis
chemorepellent activity
negative regulation of nervous system development_1
semaphorin-plexin signaling pathway
mesenchymal cell development
limbic system development_1
negative regulation of neurogenesis_1
regulation of neuron migration
neural crest cell migration
neuron projection extension involved in neuron projecti...
regulation of axon extension_1
negative regulation of axon extension_1
axon extension involved in axon guidance
negative regulation of axonogenesis_1
NF-kappa B signaling pathway_1
Sphingolipid signaling pathway_2
Phospholipase D signaling pathway
Longevity regulating pathway_1
Gap junction
B cell receptor signaling pathway_1
Fc epsilon RI signaling pathway_1
Long-term potentiation_1
Thyroid hormone signaling pathway
Pertussis_1
Yersinia infection_1
Colorectal cancer
Thyroid cancer_1
Bladder cancer_1
Non-small cell lung cancer_1
Central carbon metabolism in cancer
Choline metabolism in cancer_1
PD-L1 expression and PD-1 checkpoint pathway in cancer_1
protein phosphorylated amino acid binding_1
phosphotyrosine residue binding_1
positive regulation of cytokine production involved ...
lipopolysaccharide-mediated signaling pathway_1
regulation of actin cytoskeleton reorganization

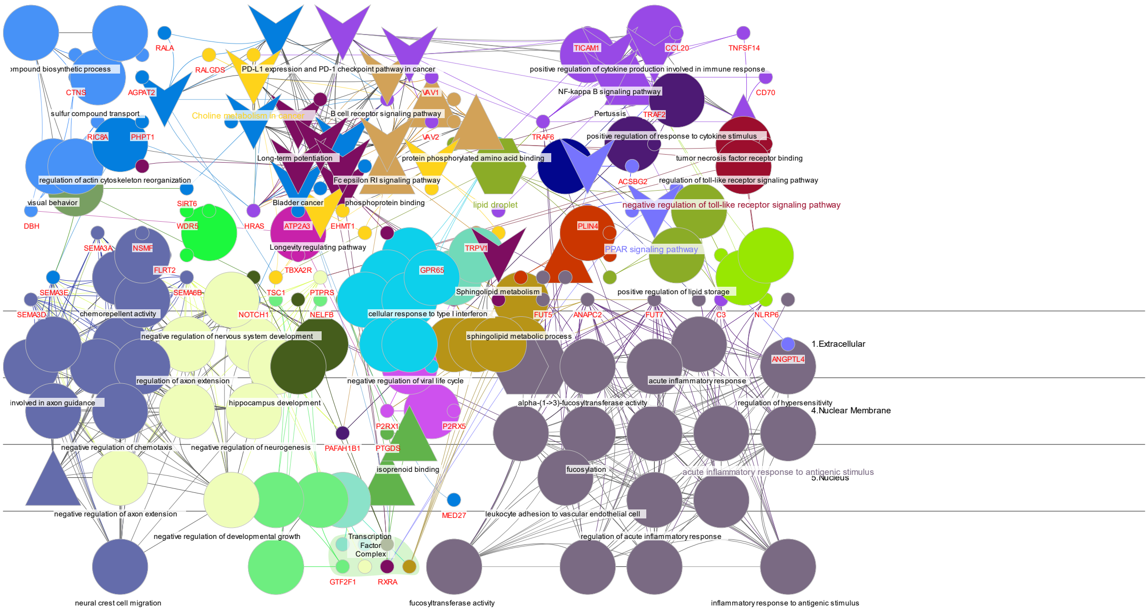

Supplementary Figure S2. KEGG pathway and GO term analysis of genes for the Bullmastiff compared to other breeds within the European Mastiff clade.

The bar chart shows the different GO terms. KEGG pathways are grouped according to their similarities. The number of genes is shown next to each GO term and KEGG pathway category. The percentage (%) of genes found from the total number of associated genes. Visualization of the network constructed from GO term and KEGG pathways was carried out using ClueGO in Cytoscape with kappa score threshold level ≥0.4. Only the most significant terms in the group are labelled.

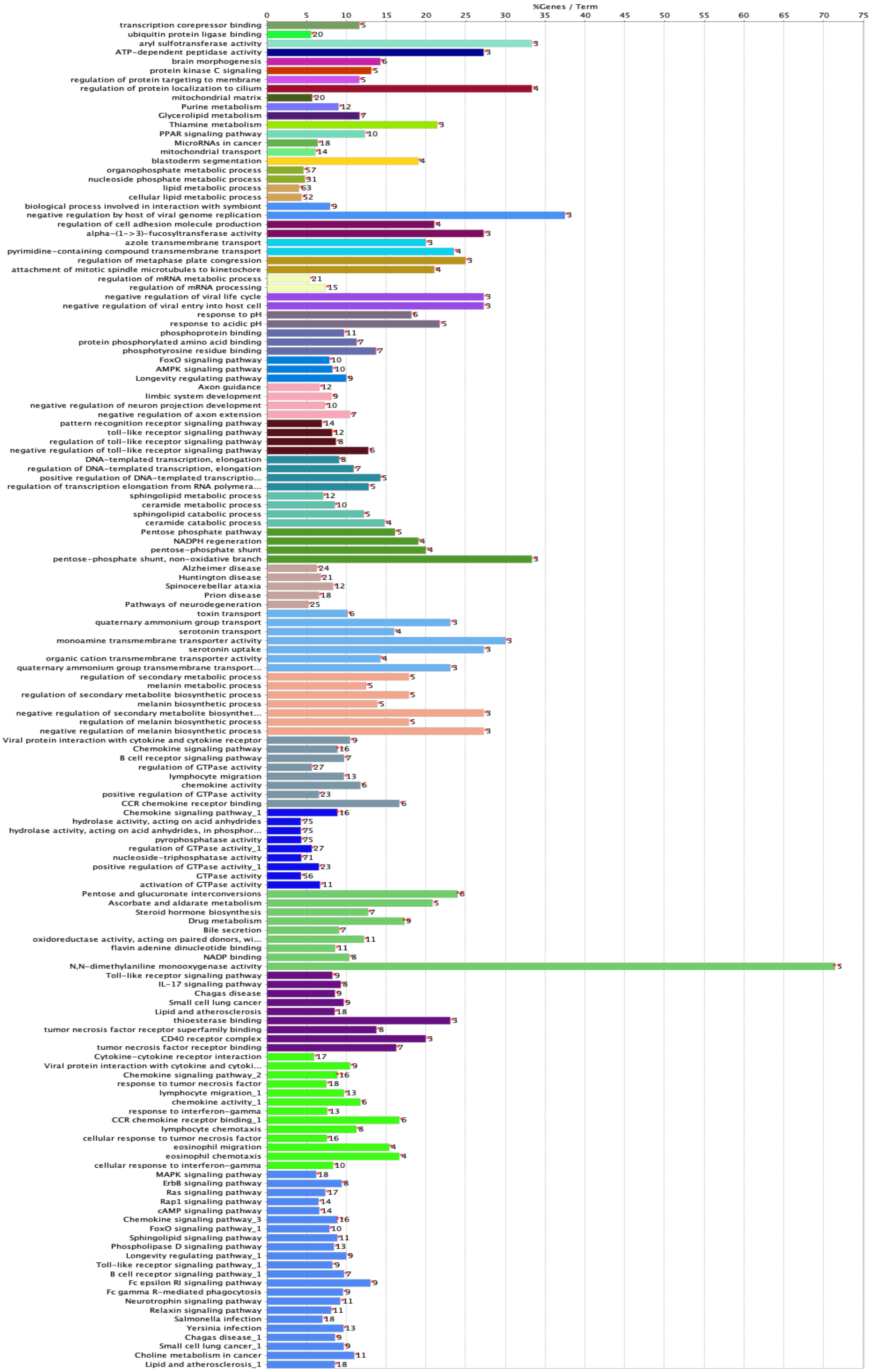

%Genes / Term
0
5
10
15
20
25
30
35
40
45
50
55
60
65
70
75
transcription corepressor binding
ubiquitin protein ligase binding
aryl sulfotransferase activity
ATP-dependent peptidase activity
brain morphogenesis
protein kinase C signaling
regulation of protein targeting to membrane
regulation of protein localization to cilium
mitochondrial matrix
Purine metabolism
Glycerolipid metabolism
Thiamine metabolism
PPAR signaling pathway
MicroRNAs in cancer
mitochondrial transport
blastoderm segmentation
organophosphate metabolic process
nucleoside phosphate metabolic process
lipid metabolic process
cellular lipid metabolic process
biological process involved in interaction with symbiont
negative regulation by host of viral genome replication
regulation of cell adhesion molecule production
alpha-(1->3)-fucosyltransferase activity
azole transmembrane transport
pyrimidine-containing compound transmembrane transport
regulation of metaphase plate congression
attachment of mitotic spindle microtubules to kinetochore
regulation of mRNA metabolic process
regulation of mRNA processing
negative regulation of viral life cycle
negative regulation of viral entry into host cell
response to pH
response to acidic pH
phosphoprotein binding
protein phosphorylated amino acid binding
phosphotyrosine residue binding
FoxO signaling pathway
AMPK signaling pathway
Longevity regulating pathway
Axon guidance
limbic system development
negative regulation of neuron projection development
negative regulation of axon extension
pattern recognition receptor signaling pathway
toll-like receptor signaling pathway
regulation of toll-like receptor signaling pathway
negative regulation of toll-like receptor signaling pathway
DNA-templated transcription, elongation
regulation of DNA-templated transcription, elongation
positive regulation of DNA-templated transcriptio...
regulation of transcription elongation from RNA polymera...
sphingolipid metabolic process
ceramide metabolic process
sphingolipid catabolic process
ceramide catabolic process
Pentose phosphate pathway
NADPH regeneration
pentose-phosphate shunt
pentose-phosphate shunt, non-oxidative branch
Alzheimer disease
Huntington disease
Spinocerebellar ataxia
Prion disease
Pathways of neurodegeneration
toxin transport
quaternary ammonium group transport
serotonin transport
monoamine transmembrane transporter activity
serotonin uptake
organic cation transmembrane transporter activity
quaternary ammonium group transmembrane transport...
regulation of secondary metabolic process
melanin metabolic process
regulation of secondary metabolite biosynthetic process
melanin biosynthetic process
negative regulation of secondary metabolite biosynthet...
regulation of melanin biosynthetic process
negative regulation of melanin biosynthetic process
Viral protein interaction with cytokine and cytokine receptor
Chemokine signaling pathway
B cell receptor signaling pathway
regulation of GTPase activity
lymphocyte migration
chemokine activity
positive regulation of GTPase activity
CCR chemokine receptor binding
Chemokine signaling pathway_1
hydrolase activity, acting on acid anhydrides
hydrolase activity, acting on acid anhydrides, in phosphor...
pyrophosphatase activity
regulation of GTPase activity_1
nucleoside-triphosphatase activity
positive regulation of GTPase activity_1
GTPase activity
activation of GTPase activity
Pentose and glucuronate interconversions
Ascorbate and aldarate metabolism
Steroid hormone biosynthesis
Drug metabolism
Bile secretion
oxidoreductase activity, acting on paired donors, wi...
flavin adenine dinucleotide binding
NADP binding
N,N-dimethylaniline monooxygenase activity
Toll-like receptor signaling pathway
IL-17 signaling pathway
Chagas disease
Small cell lung cancer
Lipid and atherosclerosis
thioesterase binding
tumor necrosis factor receptor superfamily binding
CD40 receptor complex
tumor necrosis factor receptor binding
Cytokine-cytokine receptor interaction
Viral protein interaction with cytokine and cytoki...
Chemokine signaling pathway_2
response to tumor necrosis factor
lymphocyte migration_1
chemokine activity_1
response to interferon-gamma
CCR chemokine receptor binding_1
lymphocyte chemotaxis
cellular response to tumor necrosis factor
eosinophil migration
eosinophil chemotaxis
cellular response to interferon-gamma
MAPK signaling pathway
ErbB signaling pathway
Ras signaling pathway
Rap1 signaling pathway
cAMP signaling pathway
Chemokine signaling pathway_3
FoxO signaling pathway_1
Sphingolipid signaling pathway
Phospholipase D signaling pathway
Longevity regulating pathway_1
Toll-like receptor signaling pathway_1
B cell receptor signaling pathway_1
Fc epsilon RI signaling pathway
Fc gamma R-mediated phagocytosis
Neurotrophin signaling pathway
Relaxin signaling pathway
Salmonella infection
Yersinia infection
Chagas disease_1
Small cell lung cancer_1
Choline metabolism in cancer
Lipid and atherosclerosis_1

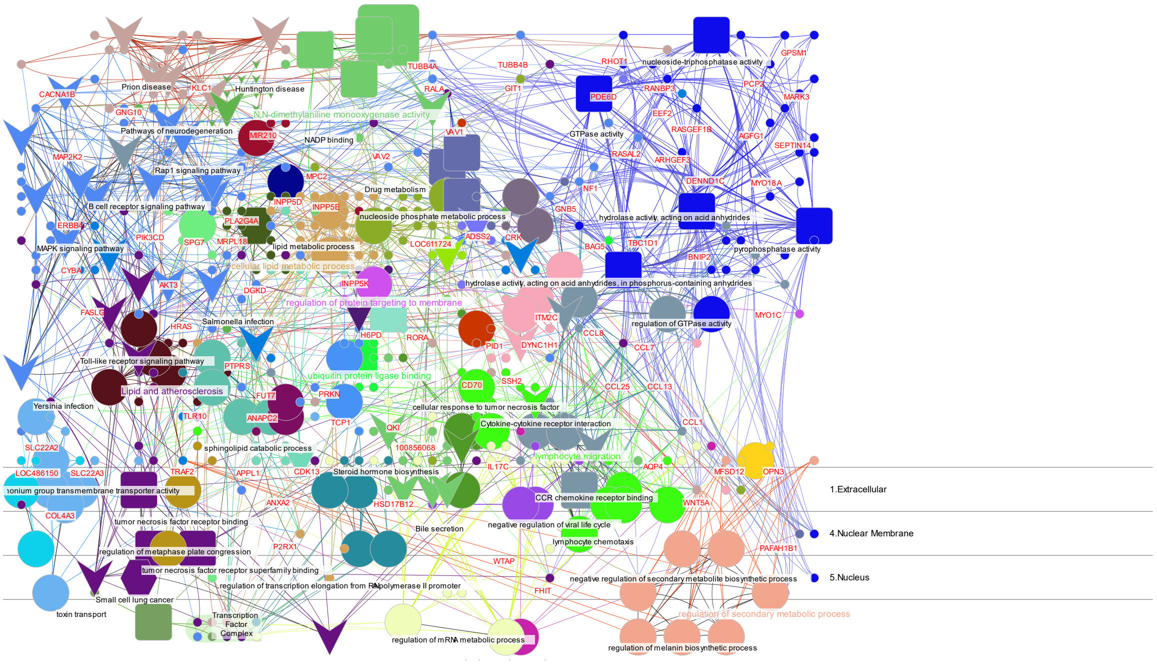

Supplementary Figure S3. Overview of KEGG pathway and GO term analysis of candidate genes found from the Bullmastiff pairwise comparisons with multiple clades.

The bar chart shows the different GO terms. KEGG pathways are grouped according to their semantic similarities. The number of genes is shown next to each GO term and KEGG pathway category. The percentage (%) of genes found from the total number of associated genes. Visualization of the network constructed from GO term and KEGG pathways was carried out using ClueGO in Cytoscape with kappa score threshold level ≥0.4. Only the most significant terms in the group are labelled.

A

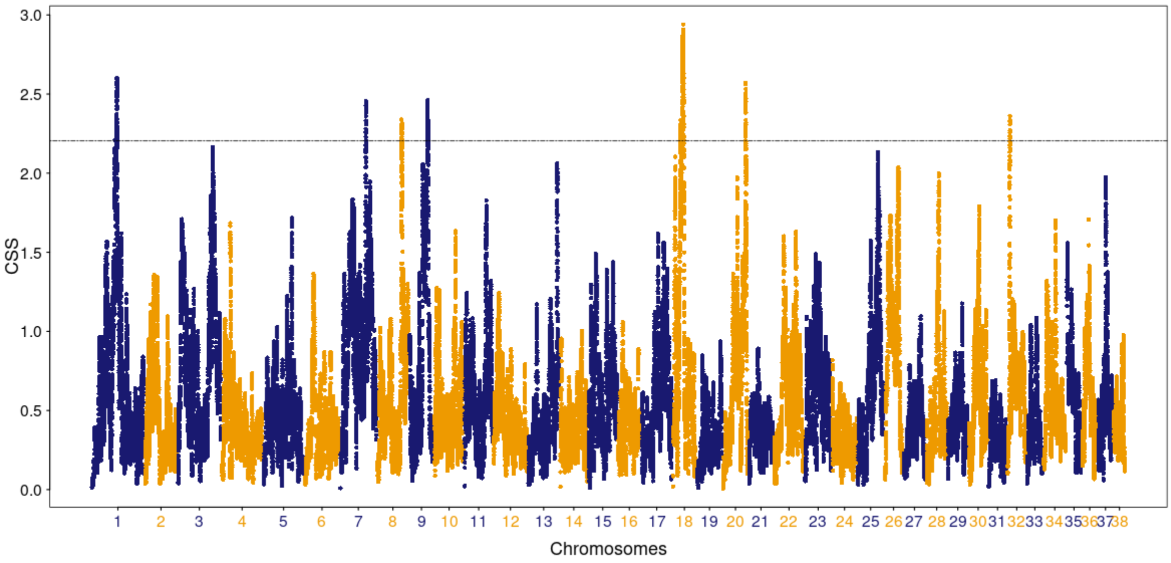

B

Chromosome	Region	CSS	Numbers of significant SNPs
1	49.07-50.04Mb	2.21	74
	52.32-54.79Mb	2.40	182
7	60.71-62.21Mb	2.35	118
8	57.18-58.93Mb	2.27	123
9	48.36-51.56Mb	2.29	239
18	22.91-24.29Mb	2.27	86
	26.41-31.77Mb	2.60	352
20	52.9-54.73Mb	2.39	120
32	4.38-5.55Mb	2.29	67

Supplementary Figure S4. **Genomic signals detected in Bullmastiff dogs compared to Asian Spitz groups using the CSS method.**

The smoothed CSS results of Bullmastiff vs Asian Spitz pairwise comparisons are shown in the Manhattan plot (A). The x-axis represents the 38 autosomes shown in alternate orange and midnight blue color and the y-axis represents the mean value of CSS scores in a one Mb window size. The black line is the thresholds for top 0.5% $-\log_{10}$ *P* values (CSS value). The summary of significant regions identified is shown in (B).

A

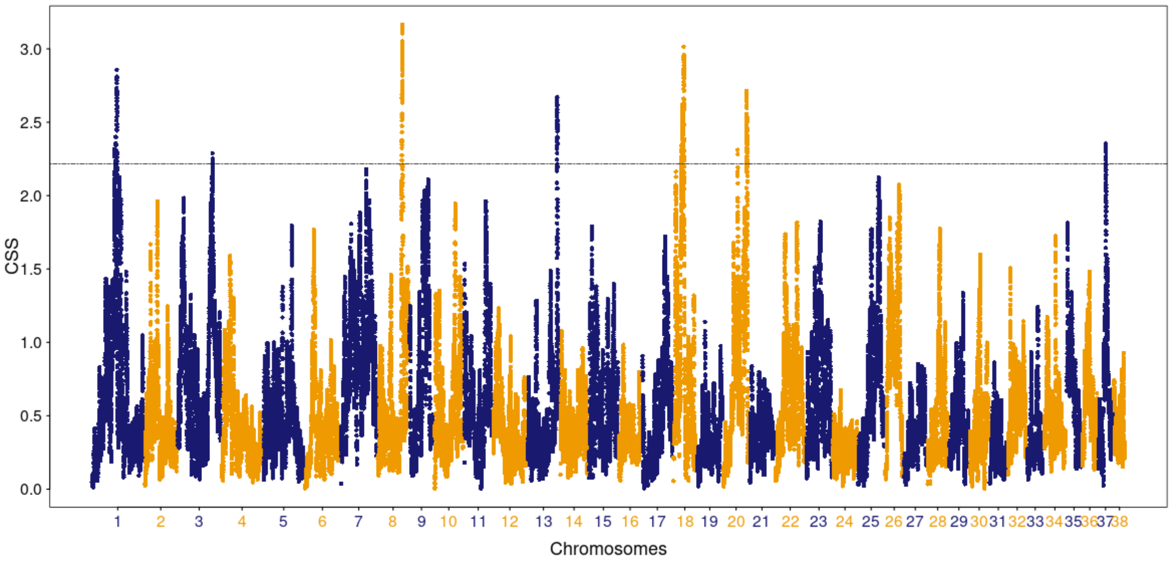

B

Chromosome	Region	CSS	Numbers of significant SNPs
1	48.84-50.04Mb	2.30	87
	51.56-52.73Mb	2.32	75
	53.18-54.95Mb	2.59	132
3	76.87-78.26Mb	2.24	112
8	56.95-59.75Mb	2.79	180
13	59.41-61.14Mb	2.48	133
18	25.33-26.65Mb	2.28	76
	26.85-29.13Mb	2.45	151
	29.52-31.78Mb	2.71	161
20	29.92-30.89Mb	2.30	50
	52.76-55.87Mb	2.32	207
37	17.72-18.99Mb	2.59	80

Supplementary Figure S5. **Genomic signals detected in Bullmastiff dogs compared to Asian Toy groups using the CSS method.**

The smoothed CSS results of Bullmastiff vs Asian Toy pairwise comparisons are shown in the Manhattan plot (A). The x-axis represents the 38 autosomes shown in alternate orange and blue color and the y-axis represents the mean value of CSS scores in a one Mb window size. The black line is the thresholds for top 0.5% $-\log_{10} P$ values (CSS value). The summary of significant regions identified is shown in (B).

A

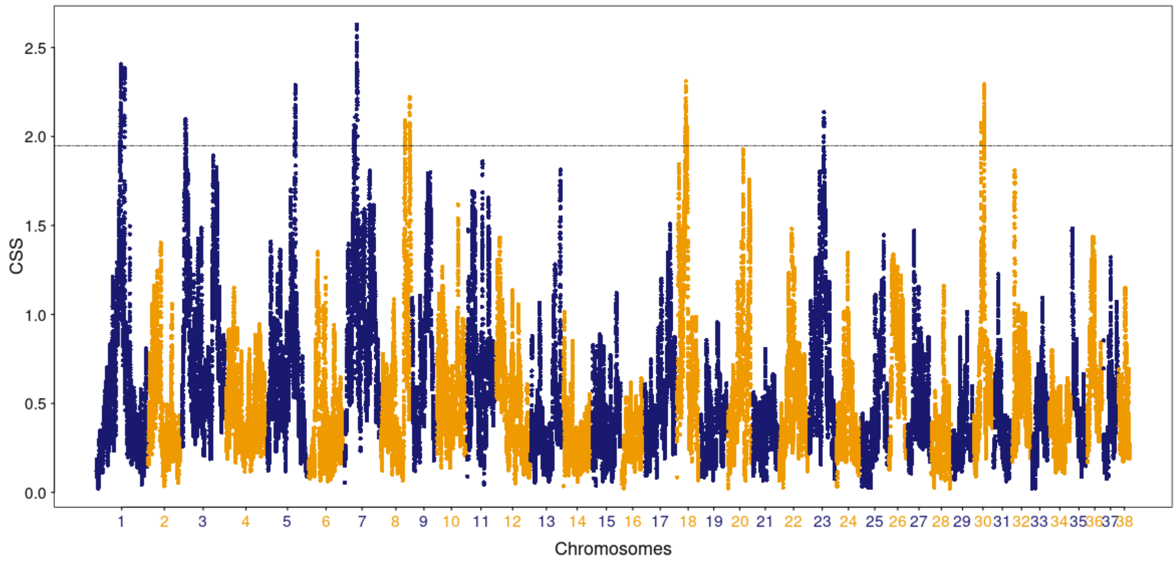

B

Chromosome	Region	Average of CSS	Numbers of significant SNPs
1	52.66-54.63Mb	2.16	128
	62.47-64.02Mb	2.24	65
3	9.81-11.2Mb	2.01	90
5	62.14-64.46Mb	2.13	131
7	20.35-21.42Mb	2.00	44
	22.17-23.34Mb	1.99	72
	25.89-27.47Mb	2.01	83
	31.8-34.45Mb	2.36	157
8	57.65-59.02Mb	2.00	82
	68.38-70.32Mb	2.06	96
	70.67-71.63Mb	1.97	25
18	26.41-28.51Mb	2.09	121
	28.83-31.27Mb	1.98	139
23	28.83-30.09Mb	2.07	73
30	18.33-19.55Mb	2.00	57
	23.99-25.63Mb	2.12	126

Supplementary Figure S6. **Genomic signals detected in Bullmastiff dogs compared to Tibetan Terrier groups using the CSS method.**

The smoothed CSS results of Bullmastiff vs Tibetan Terrier pairwise comparisons are shown in the Manhattan plot (A). The x-axis represents the 38 autosomes shown in alternate orange and blue color and the y-axis represents the mean value of CSS scores in a one Mb window size. The black line is the thresholds for top 0.5% $-\log_{10} P$ values (CSS value). The summary of significant regions identified is shown in (B).

A

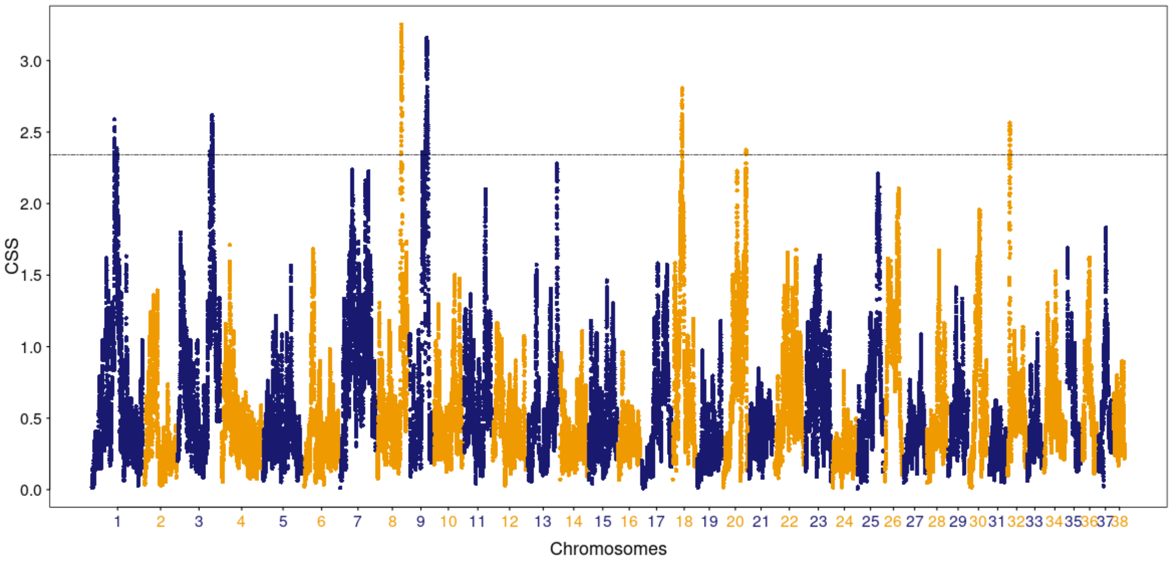

B

Chromosome	Region	CSS	Numbers of significant SNPs
1	48.84-50.09Mb	2.47	92
	51.7-52.65Mb	2.36	61
	53.39-54.59Mb	2.36	88
3	73.53-74.58Mb	2.36	91
	76.36-78.76Mb	2.44	195
8	56.86-59.75Mb	2.85	191
9	39.11-40.11Mb	2.36	77
	41.34-42.38Mb	2.35	63
	46.64-51.85Mb	2.63	375
18	26.83-29.06Mb	2.53	152
20	54.3-55.36Mb	2.36	70
32	4.22-5.5Mb	2.47	79

Supplementary Figure S7. **Genomic signals detected in Bullmastiff dogs compared to Nordic Spitz groups using the CSS method.**

The smoothed CSS results of Bullmastiff vs Nordic Spitz pairwise comparisons are shown in the Manhattan plot (A). The x-axis represents the 38 autosomes shown in alternate orange and blue color and the y-axis represents the mean value of CSS scores in a one Mb window size. The black line is the thresholds for top 0.5% $-\log_{10} P$ values (CSS value). The summary of significant regions identified is shown in (B).

A

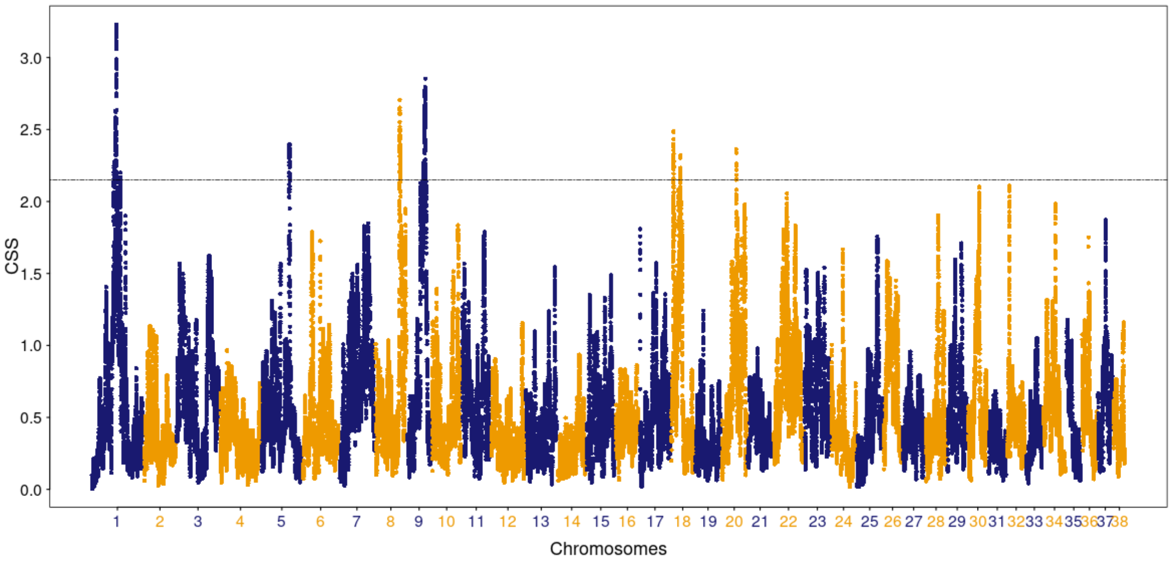

B

Chromosome	Region	CSS	Numbers of significant SNPs
1	48.64-49.99Mb	2.21	90
	51.78-55.42Mb	2.66	240
	62.51-63.58Mb	2.17	52
5	62.32-63.97Mb	2.29	101
8	56.95-59.56Mb	2.35	169
9	44.65-46.13Mb	2.20	86
	46.96-51.19Mb	2.50	278
18	8.38-10.3Mb	2.32	110
	26.95-28.24Mb	2.21	86
20	29.84-30.95Mb	2.25	58

Supplementary Figure S8. **Genomic signals detected in Bullmastiff dogs compared to Schnauzer groups using the CSS method.**

The smoothed CSS results of Bullmastiff vs Schnauzer pairwise comparisons are shown in the Manhattan plot (A). The x-axis represents the 38 autosomes shown in alternate orange and blue color and the y-axis represents the mean value of CSS scores in a one Mb window size. The black line is the thresholds for top 0.5% $-\log_{10}$ *P* values (CSS value). The summary of significant regions identified is shown in (B).

A

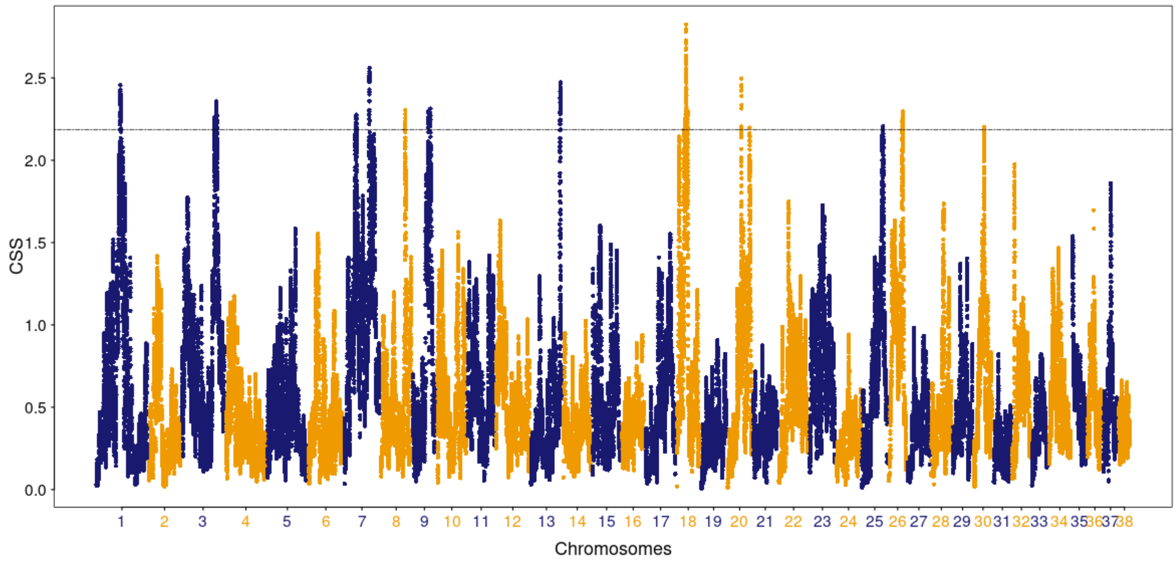

B

Chromosome	Region	CSS	Numbers of significant SNPs
1	51.7-54.87Mb	2.31	209
3	73.58-75.02Mb	2.22	109
	76.54-78.83Mb	2.27	174
7	32.02-33.73Mb	2.23	109
	60.81-62.27Mb	2.42	101
8	57.18-58.3Mb	2.24	77
9	44.6-45.74Mb	2.24	76
	48.95-50.44Mb	2.25	95
13	59.44-61.13Mb	2.36	125
18	23.15-24.13Mb	2.19	59
	25.2-29.47Mb	2.36	244
	30.5-31.71Mb	2.26	85
20	29.79-31.04Mb	2.34	60
	53.6-54.59Mb	2.20	67
25	42.35-43.58Mb	2.19	89
26	31.75-33.69Mb	2.23	131
30	24.33-25.44Mb	2.19	87

Supplementary Figure S9. **Genomic signals detected in Bullmastiff dogs compared to Small Spitz groups using the CSS method.**

The smoothed CSS results of Bullmastiff vs Small Spitz pairwise comparisons are shown in the Manhattan plot (A). The x-axis represents the 38 autosomes shown in alternate orange and blue color and the y-axis represents the mean value of CSS scores in a one Mb window size. The black line is the thresholds for top 0.5% -$\log_{10} P$ values (CSS value). The summary of significant regions identified is shown in (B).

A

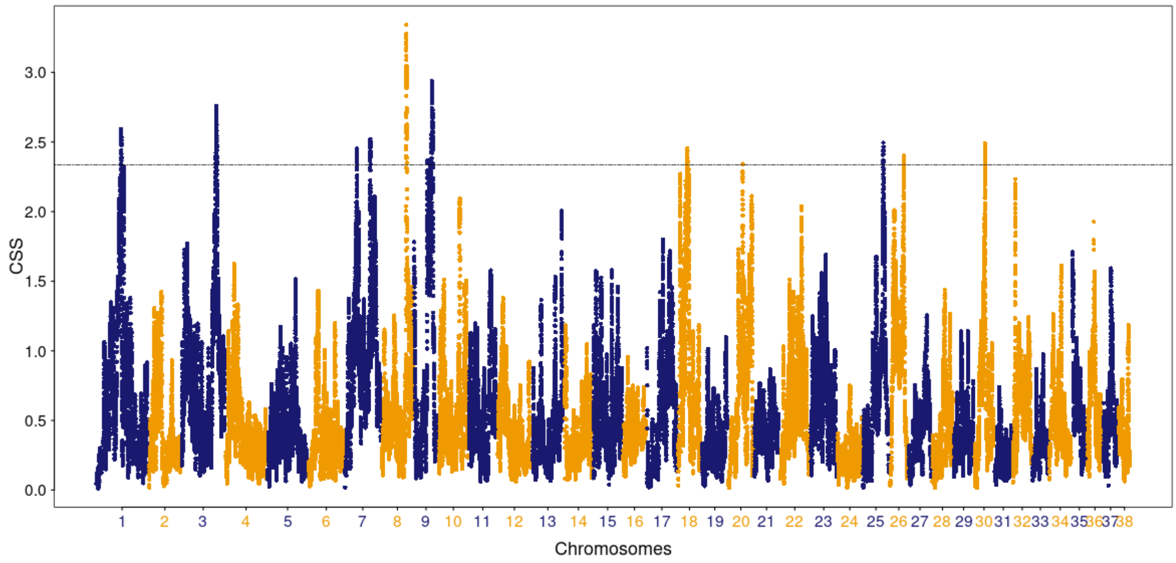

B

Chromosome	Region	CSS	Numbers of significant SNPs
1	53.19-54.9Mb	2.51	117
3	76.02-78.83Mb	2.56	220
7	31.88-33.17Mb	2.40	84
	60.85-62.2Mb	2.45	111
8	56.58-59.51Mb	2.80	196
9	39.05-40.14Mb	2.36	84
	44.65-45.71Mb	2.34	69
	47.17-48.51Mb	2.43	91
	48.57-51.5Mb	2.67	210
18	27.15-28.72Mb	2.38	106
20	29.92-30.89Mb	2.34	51
25	40.66-41.85Mb	2.41	82
26	32.28-33.57Mb	2.37	90
30	24.09-25.53Mb	2.41	120

Supplementary Figure S10. **Genomic signals detected in Bullmastiff dogs compared to Toy Spitz groups using the CSS method.**

The smoothed CSS results of Bullmastiff vs Toy Spitz pairwise comparisons are shown in the Manhattan plot (A). The x-axis represents the 38 autosomes shown in alternate orange and blue color and the y-axis represents the mean value of CSS scores in a one Mb window size. The black line is the thresholds for top 0.5% $-\log_{10} P$ values (CSS value). The summary of significant regions identified is shown in (B).

A

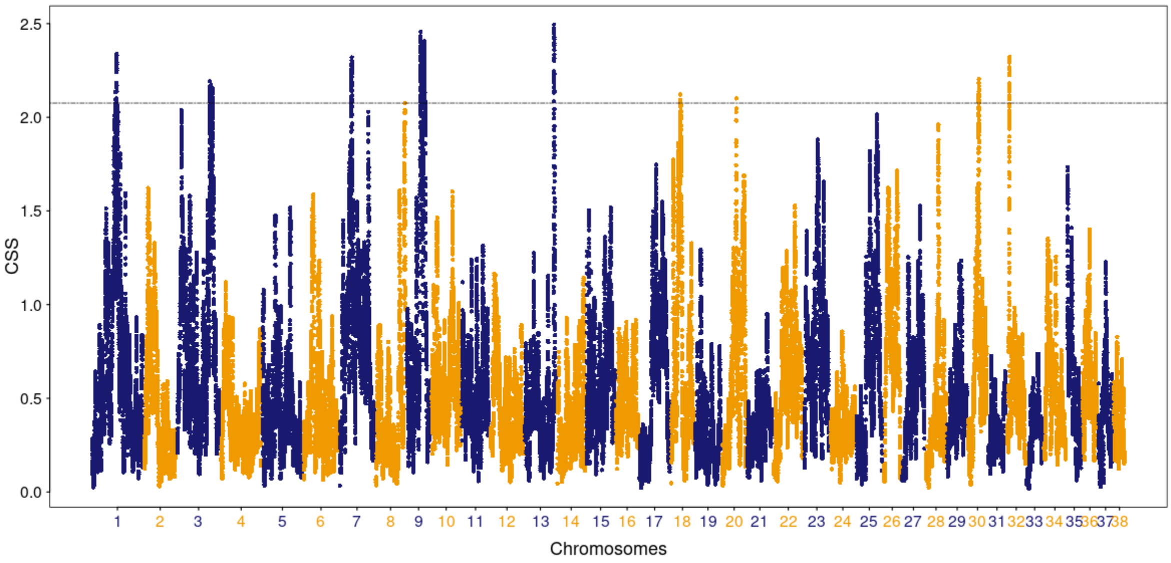

B

Chromosome	Region	CSS	Numbers of significant SNPs
1	50.91-51.79Mb	2.11	62
	53.43-55.08Mb	2.26	102
3	73.49-74.79Mb	2.13	102
	75.79-77.35Mb	2.12	117
	78.3-79.62Mb	2.10	94
7	30.82-33.73Mb	2.20	168
9	38.93-42.39Mb	2.25	204
	47.25-51.21Mb	2.21	250
13	59.29-61.14Mb	2.32	121
18	27.18-28.29Mb	2.09	64
20	29.92-30.82Mb	2.10	43
30	24.31-25.5Mb	2.13	91
32	4.22-5.56Mb	2.19	73

Supplementary Figure S11. **Genomic signals detected in Bullmastiff dogs compared to Hungarian groups using the CSS method.**

The smoothed CSS results of Bullmastiff vs Hungarian pairwise comparisons are shown in the Manhattan plot (A). The x-axis represents the 38 autosomes shown in alternate orange and blue color and the y-axis represents the mean value of CSS scores in a one Mb window size. The black line is the thresholds for top 0.5% $-\log_{10} P$ values (CSS value). The summary of significant regions identified is shown in (B).

A

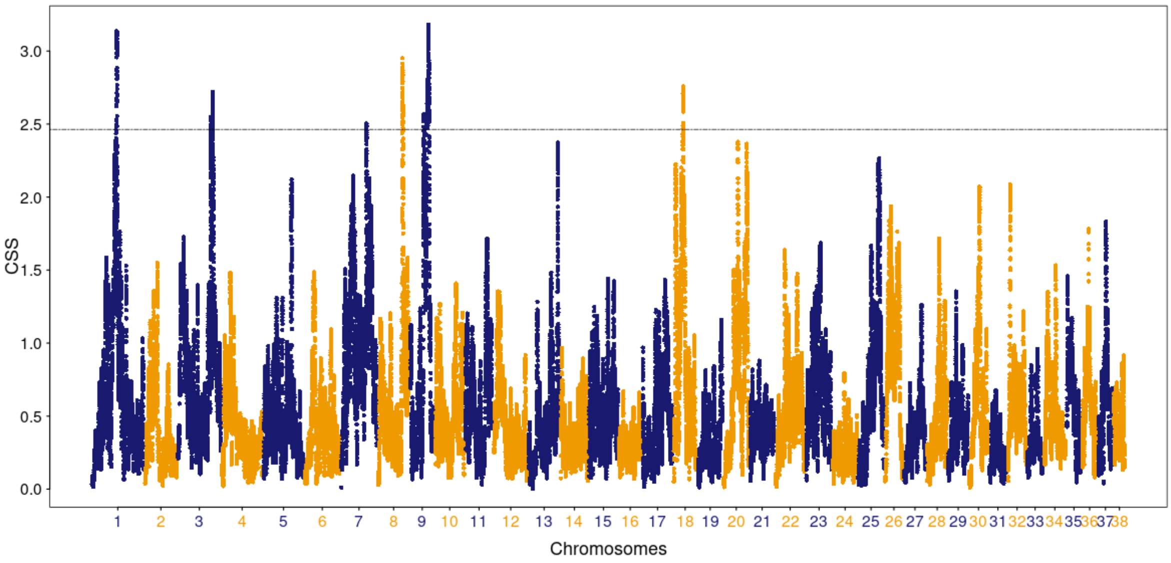

B

Chromosome	Region	CSS	Numbers of significant SNPs
1	52.03-53.01Mb	2.48	71
	53.14-55.08Mb	2.92	147
3	73.76-74.99Mb	2.50	104
	76.88-78.85Mb	2.59	161
7	60.98-62.09Mb	2.49	98
8	56.96-59.48Mb	2.69	173
9	39.05-40.14Mb	2.54	85
	46.49-51.79Mb	2.76	400
18	26.97-29.06Mb	2.64	147

Supplementary Figure S12. **Genomic signals detected in Bullmastiff dogs compared to Poodle group using the CSS method.**

The smoothed CSS results of Bullmastiff vs Poodle pairwise comparisons are shown in the Manhattan plot (A). The x-axis represents the 38 autosomes shown in alternate orange and blue color and the y-axis represents the mean value of CSS scores in a one Mb window size. The black line is the thresholds for top 0.5% $-\log_{10}$ *P* values (CSS value). The summary of significant regions identified is shown in (B).

A

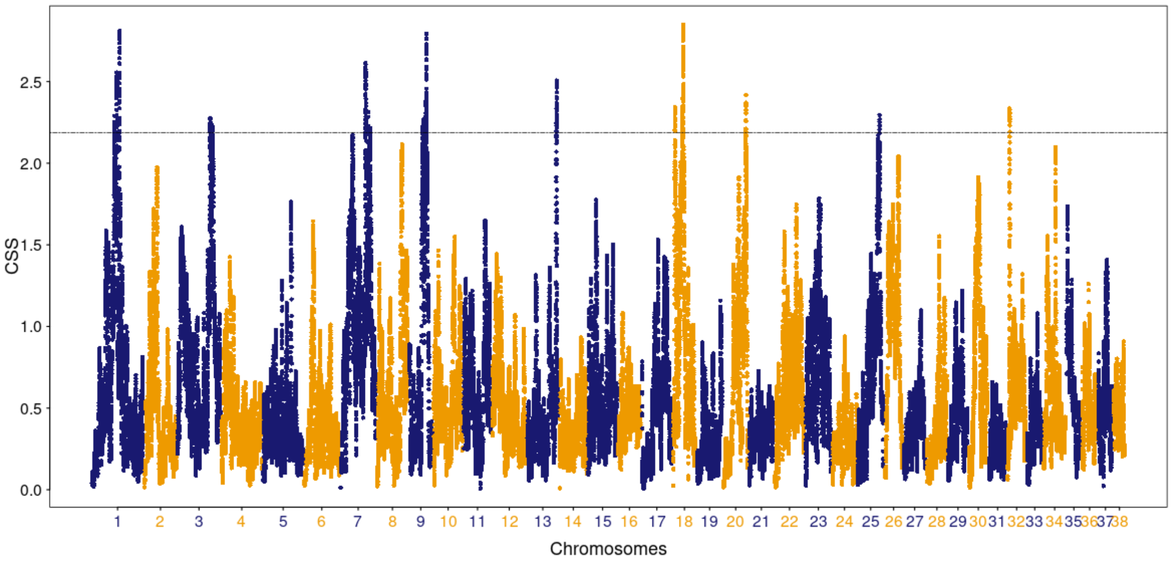

B

Chromosome	Region	CSS	Numbers of significant SNPs
1	48.94-50.04Mb	2.24	84
	53.12-54.95Mb	2.42	125
	59.46-61.06Mb	2.55	95
3	73.33-74.55Mb	2.23	97
	76.94-78.01Mb	2.21	83
7	60.7-62.33Mb	2.45	133
	66.19-67.43Mb	2.22	79
	69.89-71.31Mb	2.20	107
9	39.06-40.14Mb	2.20	79
	40.97-42.38Mb	2.22	84
	46.94-50.29Mb	2.41	224
13	59.48-61.09Mb	2.34	122
18	7.97-9.47Mb	2.28	86
	26.97-28.79Mb	2.25	117
	29.11-31.53Mb	2.57	150
20	53.25-54.73Mb	2.28	102
25	43.68-44.77Mb	2.25	64
32	4.1-5.52Mb	2.28	86

Supplementary Figure S13. **Genomic signals detected in Bullmastiff dogs compared to American Toy groups using the CSS method.**

The smoothed CSS results of Bullmastiff vs American Toy pairwise comparisons are shown in the Manhattan plot (A). The x-axis represents the 38 autosomes shown in alternate orange and blue color and the y-axis represents the mean value of CSS scores in a one Mb window size. The light purple line is the thresholds for top 0.5% $-\log_{10}$ P values (CSS value). The summary of significant regions identified is shown in (B).

A

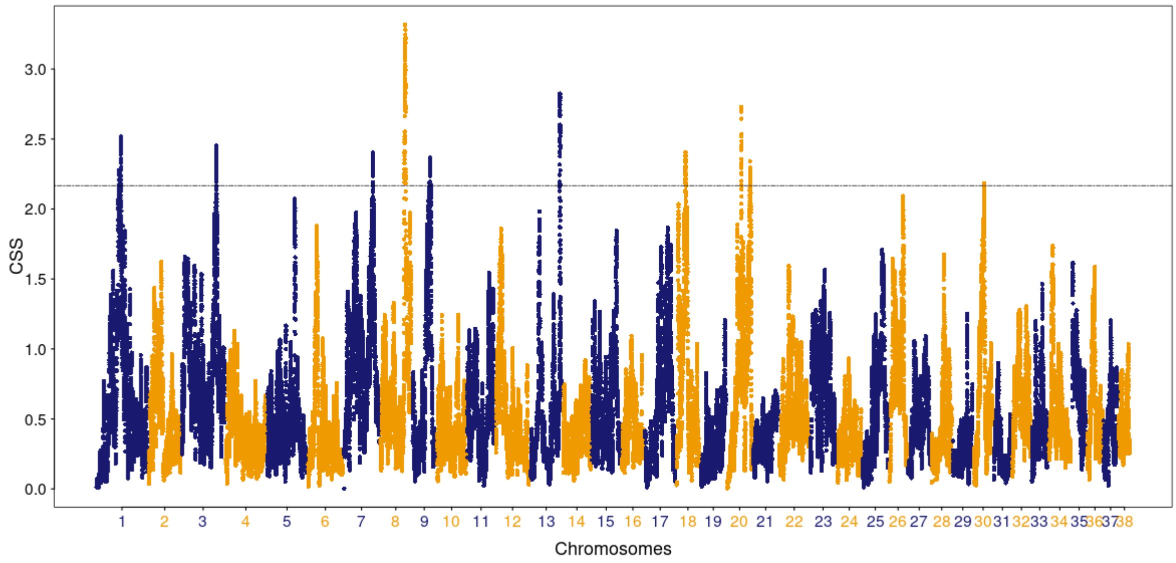

B

Chromosome	Region	CSS	Numbers of significant SNPs
1	48.94-50.04Mb	2.25	76
	51.53-54.99Mb	2.36	220
3	76.68-78.7Mb	2.33	153
7	68.91-70.77Mb	2.30	135
8	56.55-59.88Mb	2.84	205
9	47.25-48.21Mb	2.19	58
	48.92-51.09Mb	2.26	154
13	59.38-61.2Mb	2.57	137
18	26.85-28.72Mb	2.26	116
20	29.75-31.23Mb	2.46	85
	53.4-55.77Mb	2.24	157
30	24.31-25.32Mb	2.18	82

Supplementary Figure S14. **Genomic signals detected in Bullmastiff dogs compared to American Terrier groups using the CSS method.**

The smoothed CSS results of Bullmastiff vs American Terrier pairwise comparisons are shown in the Manhattan plot (A). The x-axis represents the 38 autosomes shown in alternate orange and blue color and the y-axis represents the mean value of CSS scores in a one Mb window size. The black line is the thresholds for top 0.5% $-\log_{10} P$ values (CSS value). The summary of significant regions identified is shown in (B).

A

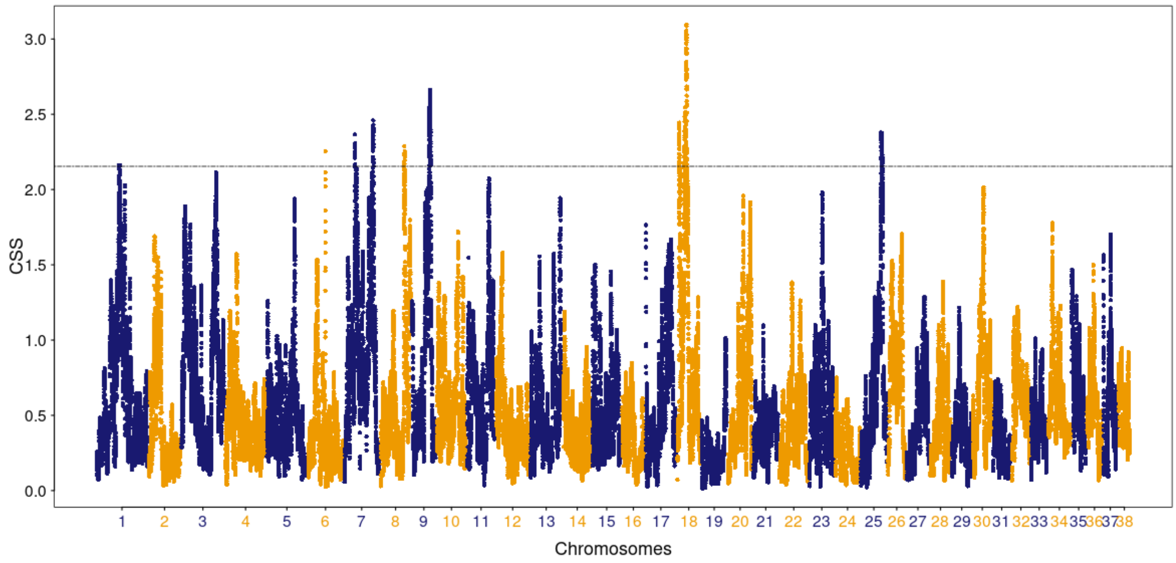

B

Chromosome	Region	CSS	Numbers of significant SNPs
1	48.98-49.95Mb	2.16	65
	51.63-52.57Mb	2.16	56
6	38.94-39.41Mb	2.26	15
7	26.59-27.31Mb	2.26	37
	68.98-70.78Mb	2.32	136
8	57.12-58.93Mb	2.21	123
9	47.16-51.49Mb	2.42	285
18	7.99-10.83Mb	2.30	151
	22.7-24.72Mb	2.38	120
	25.08-26.16Mb	2.18	51
	26.44-29.06Mb	2.62	169
25	40.63-43.58Mb	2.25	184
	43.71-44.74Mb	2.19	62

Supplementary Figure S15. **Genomic signals detected in Bullmastiff dogs compared to Pinscher groups using the CSS method.**

The smoothed CSS results of Bullmastiff vs Pinscher pairwise comparisons are shown in the Manhattan plot (A). The x-axis represents the 38 autosomes shown in alternate orange and blue color and the y-axis represents the mean value of CSS scores in a one Mb window size. The black line is the thresholds for top 0.5% $-\log_{10} P$ values (CSS value). The summary of significant regions identified is shown in (B).

A

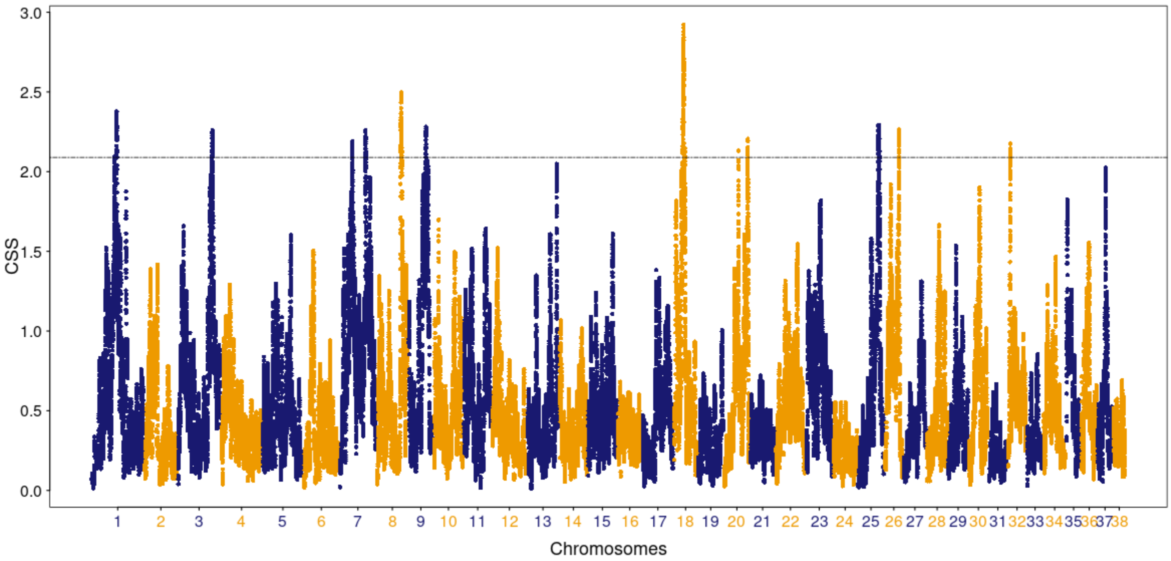

B

Chromosome	Region	CSS	Numbers of significant SNPs
1	49.23-50.27Mb	2.09	75
	53.18-54.99Mb	2.28	132
3	76.94-78.87Mb	2.18	156
7	31.89-33.32Mb	2.13	103
	60.73-62.25Mb	2.17	128
8	56.68-59.75Mb	2.24	203
9	46.49-47.73Mb	2.19	91
18	26.19-29.86Mb	2.52	226
	30.5-31.64Mb	2.12	92
20	29.92-30.89Mb	2.13	52
	54.16-55.36Mb	2.15	89
25	40.66-43.73Mb	2.18	221
26	32.39-33.63Mb	2.18	94
32	4.4-5.56Mb	2.13	75

Supplementary Figure S16. **Genomic signals detected in Bullmastiff dogs compared to Terrier using the CSS method.**

The smoothed CSS results of Bullmastiff vs Terrier pairwise comparisons are shown in the Manhattan plot (A). The x-axis represents the 38 autosomes shown in alternate orange and blue color and the y-axis represents the mean value of CSS scores in a one Mb window size. The black line is the thresholds for top 0.5% $-\log_{10}$ P values (CSS value). The summary of significant regions identified is shown in (B).

A

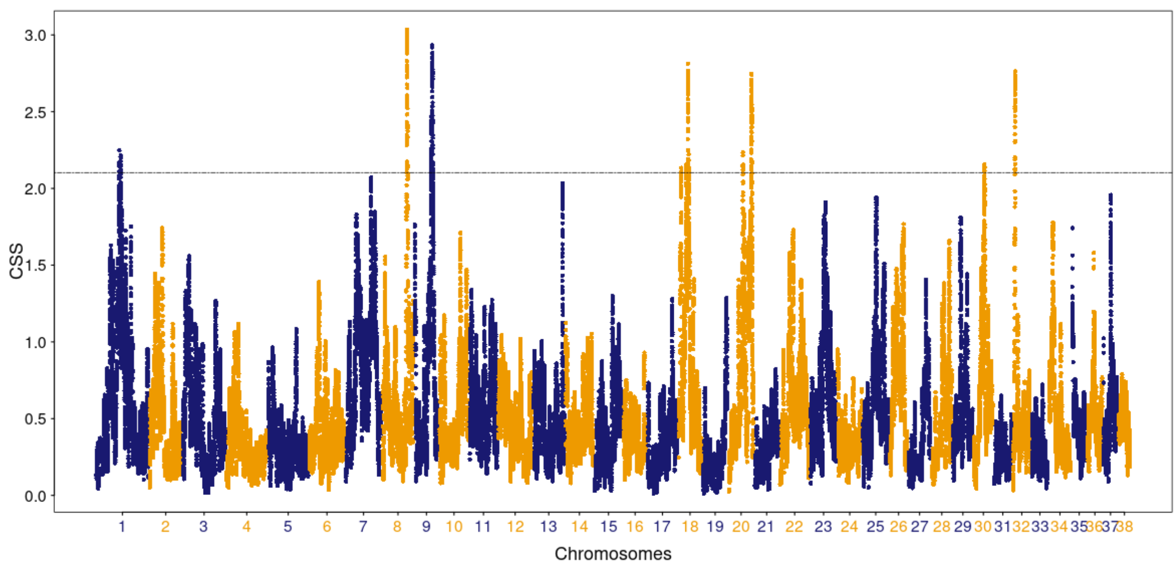

B

Chromosome	Region	CSS	Numbers of significant SNPs
1	48.88-50.04Mb	2.18	87
	51.53-52.76Mb	2.15	81
8	56.71-59.49Mb	2.67	185
9	46.44-50.87Mb	2.41	315
18	9.27-10.95Mb	2.11	83
	22.84-24.23Mb	2.13	93
	27.13-29.13Mb	2.55	133
	29.22-30.66Mb	2.13	79
20	29.86-30.95Mb	2.16	60
	53.01-55.44Mb	2.47	165
30	24.31-25.36Mb	2.14	86
32	3.92-5.64Mb	2.54	107

Supplementary Figure S17. **Genomic signals detected in Bullmastiff dogs compared to New World groups using the CSS method.**

The smoothed CSS results of Bullmastiff vs New World pairwise comparisons are shown in the Manhattan plot (A). The x-axis represents the 38 autosomes shown in alternate orange and blue color and the y-axis represents the mean value of CSS scores in a one Mb window size. The black line is the thresholds for top 0.5% -$\log_{10}$ P values (CSS value). The summary of significant regions identified is shown in (B).

A

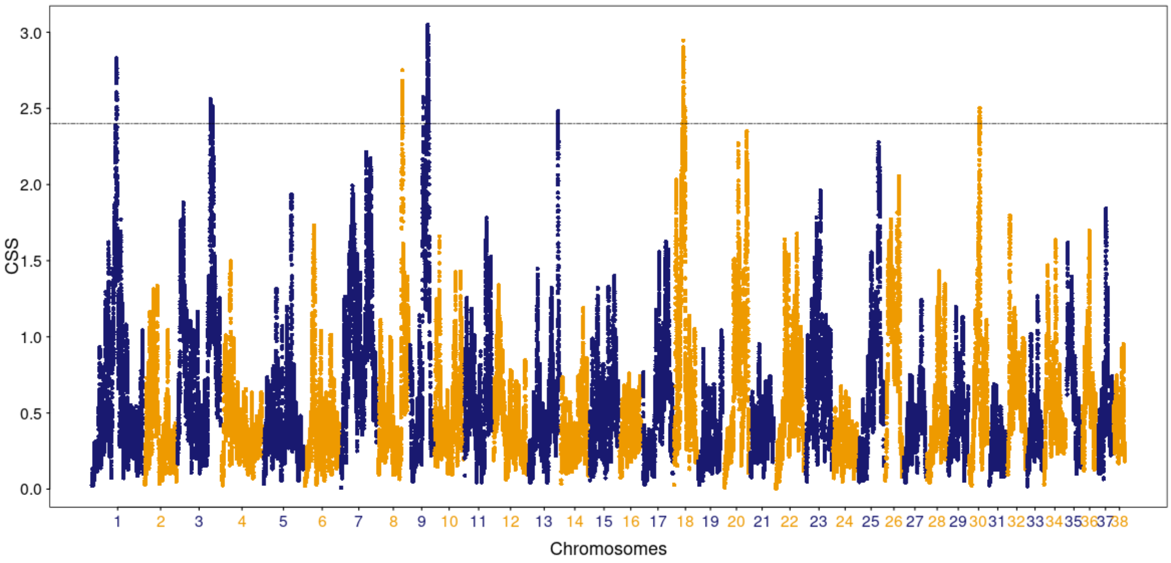

B

Chromosome	Region	CSS	Numbers of significant SNPs
1	53.18-55.02Mb	2.67	137
3	73.33-74.95Mb	2.47	130
	76.83-78.38Mb	2.45	128
8	57.06-59.25Mb	2.55	161
9	39.02-40.17Mb	2.52	88
	46.96-51.79Mb	2.70	352
13	59.64-60.8Mb	2.44	88
18	26.41-29.52Mb	2.65	200
	30.33-31.48Mb	2.44	86
30	24.25-25.36Mb	2.45	91

Supplementary Figure S18. **Genomic signals detected in Bullmastiff dogs compared to Mediterranean groups using the CSS method.**

The smoothed CSS results of Bullmastiff vs Mediterranean pairwise comparisons are shown in the Manhattan plot (A). The x-axis represents the 38 autosomes shown in alternate orange and blue color and the y-axis represents the mean value of CSS scores in a one Mb window size. The black line is the thresholds for top 0.5% $-\log_{10}$ P values (CSS value). The summary of significant regions identified is shown in (B).

A

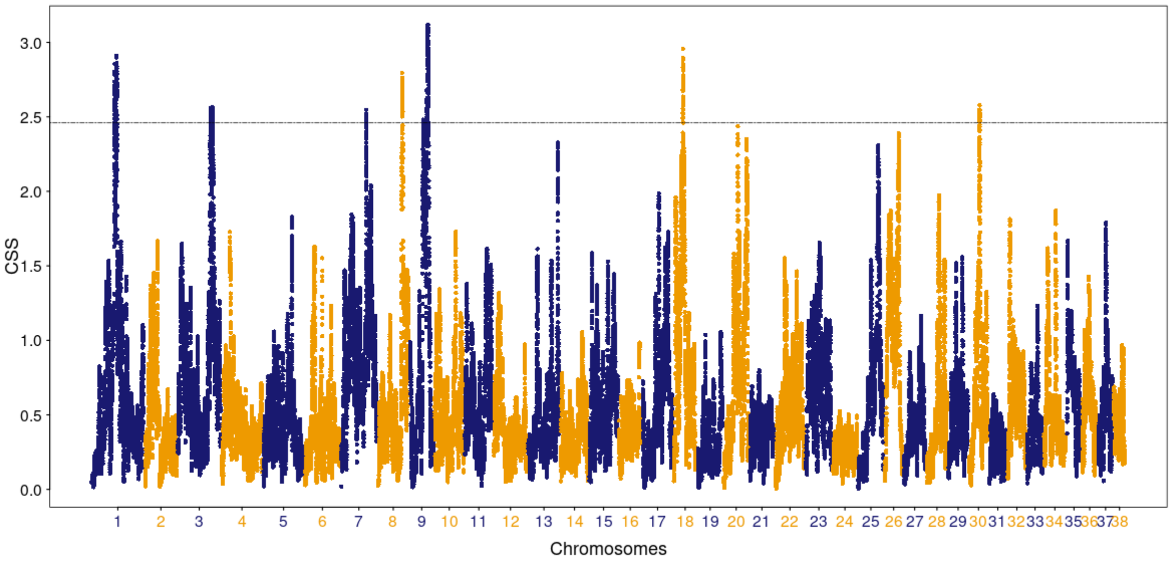

B

Chromosome	Region	CSS	Numbers of significant SNPs
1	48.69-50.4Mb	2.64	125
	51.57-53.01Mb	2.53	92
	53.12-54.9Mb	2.76	130
3	73.5-74.75Mb	2.50	109
	76.96-78.45Mb	2.51	120
7	60.9-62.13Mb	2.49	107
8	57.06-59.48Mb	2.63	170
9	39.06-40.12Mb	2.47	82
	46.64-51.79Mb	2.75	384
18	26.97-28.79Mb	2.70	122
30	24.25-25.48Mb	2.51	101

Supplementary Figure S19. **Genomic signals detected in Bullmastiff dogs compared to Scent Hound groups using the CSS method.**

The smoothed CSS results of Bullmastiff vs Scent Hound pairwise comparisons are shown in the Manhattan plot (A). The x-axis represents the 38 autosomes shown in alternate orange and blue color and the y-axis represents the mean value of CSS scores in a one Mb window size. The black line is the thresholds for top 0.5% $-\log_{10}$ P values (CSS value). The summary of significant regions identified is shown in (B).

A

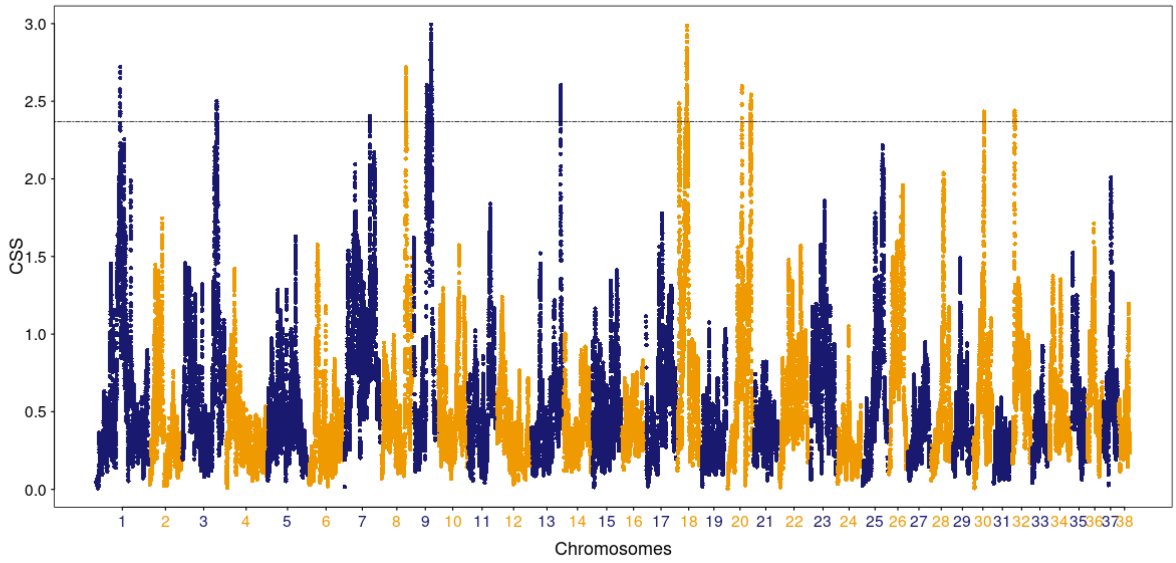

B

Chromosome	Region	CSS	Numbers of significant SNPs
1	51.51-52.73Mb	2.56	82
3	76.85-78.38Mb	2.42	124
7	60.98-62.09Mb	2.39	96
8	57.24-59.48Mb	2.55	149
9	39.02-40.2Mb	2.54	90
	40.43-42.91Mb	2.44	161
	47.23-51.24Mb	2.64	292
13	59.45-61.05Mb	2.47	125
18	8.23-10.26Mb	2.42	119
	26.85-29.57Mb	2.65	169
20	29.84-31.03Mb	2.53	65
	53.13-55.77Mb	2.44	177
30	24.31-25.48Mb	2.40	98
32	4.36-5.5Mb	2.39	67

Supplementary Figure S20. **Genomic signals detected in Bullmastiff dogs compared to Spaniel groups using the CSS method.**

The smoothed CSS results of Bullmastiff vs Spaniel pairwise comparisons are shown in the Manhattan plot (A). The x-axis represents the 38 autosomes shown in alternate orange and blue color and the y-axis represents the mean value of CSS scores in a one Mb window size. The black line is the thresholds for top 0.5% $-\log_{10}$ P values (CSS value). The summary of significant regions identified is shown in (B).

A

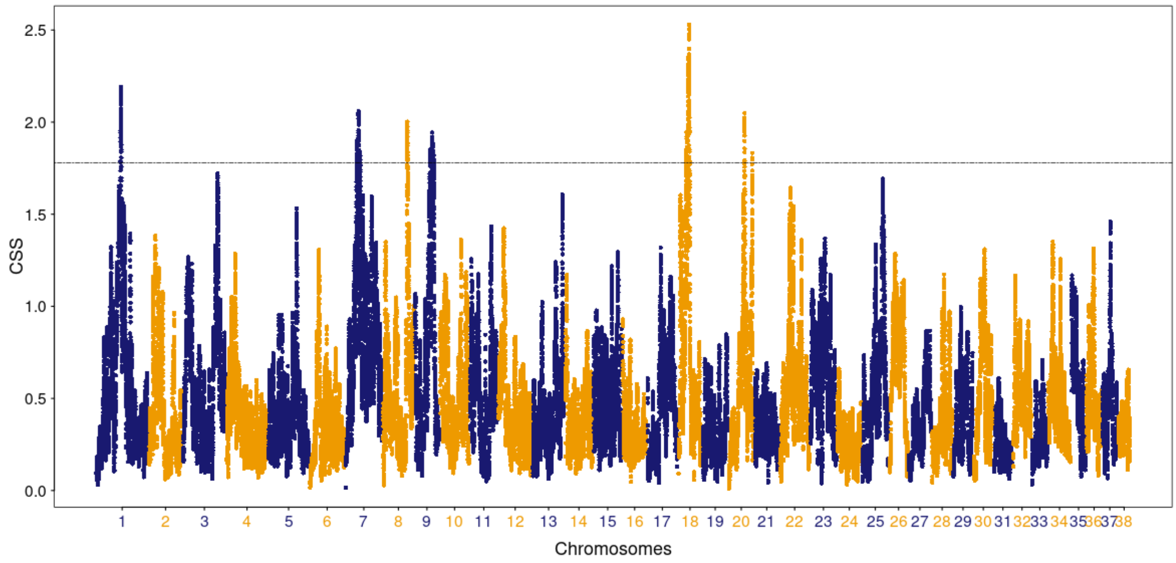

B

Chromosome	Region	CSS	Numbers of significant SNPs
1	52.4-55Mb	2.02	191
7	26.13-27.47Mb	1.84	80
	31.65-33.59Mb	1.94	137
	35.54-36.6Mb	1.81	76
8	57.08-59.02Mb	1.90	143
9	41.82-43.5Mb	1.81	98
	46.6-48.97Mb	1.86	169
	50.27-51.38Mb	1.80	93
18	22.94-24.21Mb	1.82	83
	24.3-25.56Mb	1.88	69
	26.91-31.96Mb	2.16	338
20	33.39-34.89Mb	1.96	102
	54.68-55.69Mb	1.82	71

Supplementary Figure S21. **Genomic signals detected in Bullmastiff dogs compared to Retriever groups using the CSS method.**

The smoothed CSS results of Bullmastiff vs Retriever pairwise comparisons are shown in the Manhattan plot (A). The x-axis represents the 38 autosomes shown in alternate orange and blue color and the y-axis represents the mean value of CSS scores in a one Mb window size. The black line is the thresholds for top 0.5% $-\log_{10}$ P values (CSS value). The summary of significant regions identified is shown in (B).

A

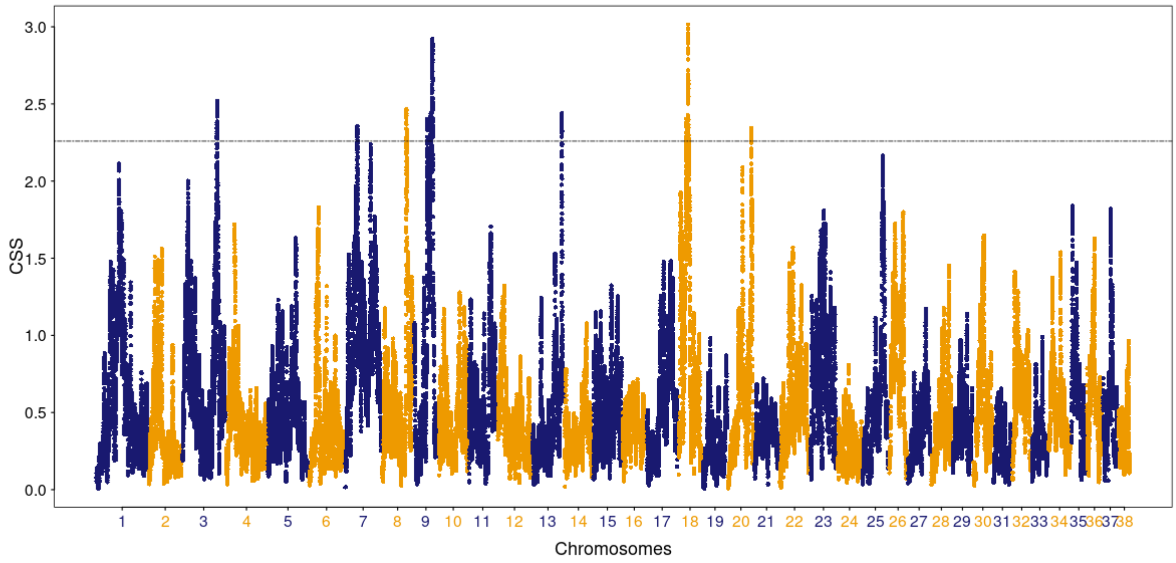

B

Chromosome	Region	CSS	Numbers of significant SNPs
3	76.85-78.83Mb	2.39	161
7	32.05-33.26Mb	2.32	84
8	57.06-59.45Mb	2.32	167
9	39.02-40.2Mb	2.36	90
	40.43-41.46Mb	2.27	69
	46.49-51.79Mb	2.52	394
13	59.59-61.09Mb	2.37	125
18	22.99-24.14Mb	2.33	77
	26.25-29.81Mb	2.53	221
	30.02-31.07Mb	2.27	81
20	53.46-54.64Mb	2.30	89

Supplementary Figure S22. **Genomic signals detected in Bullmastiff dogs compared to Pointer Setter groups using the CSS method.**

The smoothed CSS results of Bullmastiff vs Pointer Setter pairwise comparisons are shown in the Manhattan plot (A). The x-axis represents the 38 autosomes shown in alternate orange and blue color and the y-axis represents the mean value of CSS scores in a one Mb window size. The black line is the thresholds for top 0.5% $-\log_{10}$ P values (CSS value). The summary of significant regions identified is shown in (B).

A

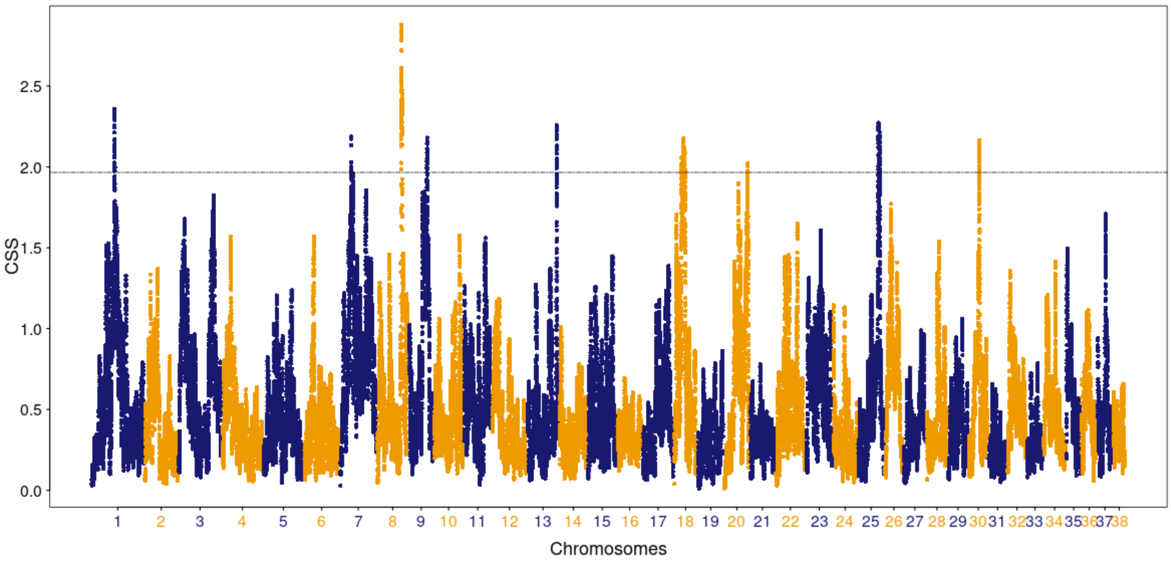

B

Chromosome	Region	CSS	Numbers of significant SNPs
1	48.88-50.69Mb	2.12	128
7	26.62-27.47Mb	2.06	48
8	56.46-59.75Mb	2.39	217
9	48.18-49.72Mb	2.07	107
13	59.55-61.02Mb	2.11	115
18	23.07-24.56Mb	2.01	96
	26.41-31.34Mb	2.05	331
20	54.19-55.3Mb	1.99	81
25	40.58-42.22Mb	2.13	117
	42.29-44.51Mb	2.08	159
30	24.26-25.59Mb	2.07	112

Supplementary Figure S23. **Genomic signals detected in Bullmastiff dogs compared to UK Rural groups using the CSS method.**

The smoothed CSS results of Bullmastiff vs UK Rural pairwise comparisons are shown in the Manhattan plot (A). The x-axis represents the 38 autosomes shown in alternate orange and blue color and the y-axis represents the mean value of CSS scores in a one Mb window size. The black line is the thresholds for top 0.5% $-\log_{10}$ P values (CSS value). The summary of significant regions identified is shown in (B).

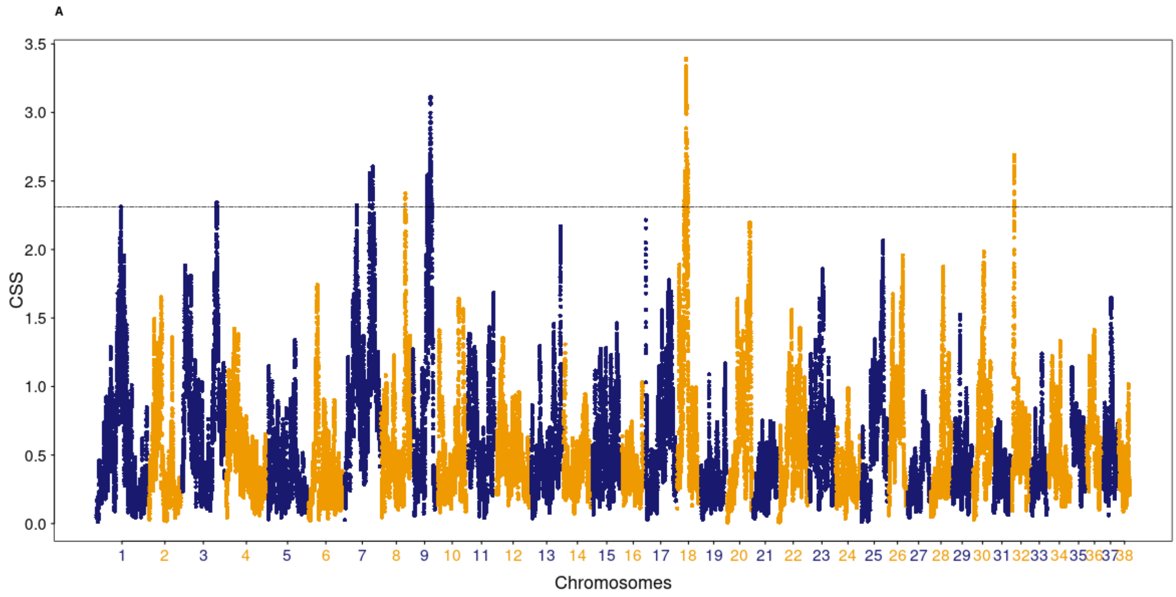

B

Chromosome	Region	CSS	Numbers of significant SNPs
1	53.41-54.37Mb	2.31	68
3	77.33-78.36Mb	2.33	79
7	32.08-33.1Mb	2.32	72
	60.81-62.13Mb	2.45	103
	66.15-67.69Mb	2.45	99
8	57.6-58.65Mb	2.36	68
9	41.08-43.09Mb	2.43	128
	46.88-51.78Mb	2.53	345
18	23.02-24.23Mb	2.33	78
	24.38-26.63Mb	2.40	119
	26.65-29.49Mb	2.85	177
	29.7-31.52Mb	2.48	123
32	4.01-5.61Mb	2.55	105

Supplementary Figure S24. **Genomic signals detected in Bullmastiff dogs compared to Alpine groups using the CSS method.**

The smoothed CSS results of Bullmastiff vs Alpine pairwise comparisons are shown in the Manhattan plot (A). The x-axis represents the 38 autosomes shown in alternate orange and blue color and the y-axis represents the mean value of CSS scores in a one Mb window size. The black line is the thresholds for top 0.5% $-\log_{10}$ P values (CSS value). The summary of significant regions identified is shown in (B).

A

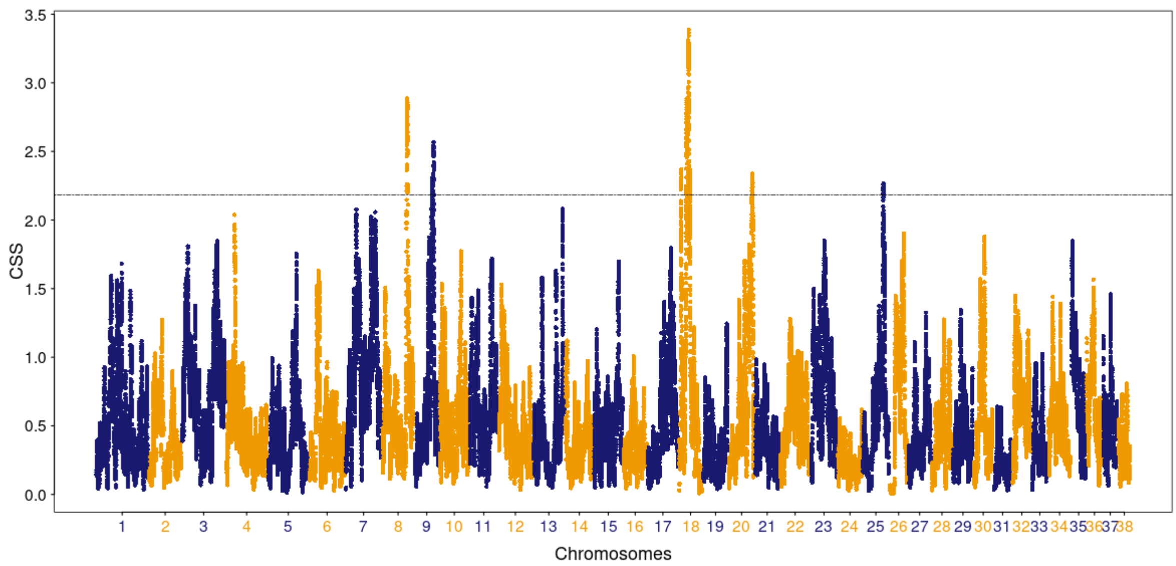

B

Chromosome	Region	CSS	Numbers of significant SNPs
8	56.71-59.88Mb	2.61	206
9	47.15-48.6Mb	2.23	106
	49.8-51.77Mb	2.42	157
18	8.08-9.56Mb	2.29	89
	22.69-25.69Mb	2.57	186
	26.41-29.93Mb	2.87	219
	30.02-31.88Mb	2.29	146
20	53.16-55.75Mb	2.23	187
25	40.71-41.88Mb	2.22	82

Supplementary Figure S25. **Genomic signals detected in Bullmastiff dogs compared to European Mastiff groups using the CSS method.**

The smoothed CSS results of Bullmastiff vs European Mastiff pairwise comparisons are shown in the Manhattan plot (A). The x-axis represents the 38 autosomes shown in alternate orange and blue color and the y-axis represents the mean value of CSS scores in a one Mb window size. The black line is the thresholds for top 0.5% $-\log_{10}$ P values (CSS value). The summary of significant regions identified is shown in (B).

Supplementary Table S1. Summary of the clade, breed, sample size, genotype data of the populations used as reference populations. Bullmastiff dogs (n=538) were used as the target group.

Clade/Group	Breed	Abbrev.	Sample Size (Reference Groups)	Reference dataset code	Number of SNPs (QC*)
-	Mastiff	MAST	32		120,239
	Bull Dog	BULD	86		130,978
Asian Spitz	Xigou	XIGO	233	I	137,315
	Tibetan Mastiff - COO	CHTM			
	Tibetan Mastiff - USA	TIBM			
	Siberian Husky	HUSK			
	Greenland Sledge Dog	GREE			
	Alaskan Malamute	AMAL			
	Shiba Inu	SHIB			
	Akita	AKIT			
	Chinese Shar-Pei	SHAR			
	Chow Chow	CHOW			
Asian Toy	Japanese Chin	CHIN	133	II	135,661
	Tibetan Spaniel	TIBS			
	Pekingese	PEKE			
	Lhasa Apso	LHSA			
	Shih Tzu	SHIH			
Tibetan Terrier	Tibetan Terrier	TIBT	16	III	125,170
Nordic Spitz	Norwegian Elkhound	NELK	65	IV	135,104
	Swedish Vallhund	SVAL			
	Icelandic Sheepdog	ICES			
	Keeshond	KEES			
Schnauzer	Standard Schnauzer	SSNZ	84	V	129,461
	Miniature Schnauzer	MSNZ			
Small Spitz	American Eskimo Dog	AESK	29	VI	128,466
	Volpino	VPIN			
	Pomeranian	POM			
Toy Spitz	Schipperke	SKIP	132	VII	135,772
	Papillon	PAPI			
	Brussels Griffon	BRUS			
	PugDog	PUG			
Hungarian	Puli	PULI	11	VIII	123,206
	Pumi	PUMI			
Poodle	Coton de Tulear	COTO	378	IX	141,141
	Portuguese Water Dog	PTWD			
	Standard Poodle	SPOO			
	Toy Poodle	TPOO			

	Miniature Poodle	MPOO			
	Bichon Frise	BICH			
	Havanese	HAVA			
	Maltese	MALT			
American Toy	Chinese Crested	CRES	51	X	132,869
	Chihuahua	CHIH			
American Terrier	Rat Terrier	RATT	27	XI	128,963
	Toy Fox Terrier	TYFX			
	American Hairless Terrier	AHRT			
Pinscher	Toy Manchester Terrier	MNTY	32	XII	127,391
	Miniature Pinscher	MPIN			
Terrier	Airedale Terrier	AIRT	623	XIII	141,108
	Kerry Blue Terrier	KERY			
	Glen of Imaal Terrier	GLEN			
	Soft Coated Wheaten Terrier	SCWT			
	Irish Terrier	IRIT			
	Bedlington Terrier	BEDT			
	Border Terrier	BORT			
	Parson Russell Terrier	PARS			
	Jack Russell terrier	JACK			
	Wire Fox Terrier	WFOX			
	Australian Terrier	AUST			
	Silky Terrier	SILK			
	Yorkshire Terrier	YORK			
	Norwich Terrier	NOWT			
	Norfolk Terrier	NORF			
	Scottish Terrier	SCOT			
	Cairn Terrier	CAIR			
	West Highland White Terrier	WHWT			
New World	Miniature Xoloitzcuintli	MXOL	304	XIV	135,229
	Xoloitzcuintli	XOLO			
	Peruvian Hairless Dog	INCA			
	Chinook	COOK			
	Berger Picard	BPIC			
	Cane Paratore	CPAT			
	German Shepherd Dog	GSD			
Mediterranean	Great Pyrenees	GPYR	146	XV	139,478
	Pharaoh Hound	PHAR			
	Cirneco dell'Etna	CIRN			
	Ibizan Hound	IBIZ			

	Komondor	KOMO			
	Kuvasz	KUVZ			
	Mastino Abruzzese	MAAB			
	Sloughi	SLOU			
	Levriero Meridionale	LVMD			
	Azawakh	AZWK			
	Anatolian Shepherd	ANAT			
	Afghan Hound	AFGH			
	Saluki - COO	COOS			
	Saluki - USA	SALU			
Scent Hound	Dachshund	DACH	210	XVI	138,597
	Petit Basset Griffon Vendeen	PBGV			
	Basset Hound	BASS			
	Redbone Coonhound	REDB			
	Beagle	BEAG			
	Foxhound	FOXH			
	Otterhound	OTTR			
	Bloodhound	BLDH			
Spaniel	English Springer Spaniel	ESSP	261	XVII	136,569
	Cavalier King Charles Spaniel	CKCS			
	American Cocker Spaniel	ACKR			
	Field Spaniel	FIEL			
	English Cocker Spaniel	ECKR			
Retriever	Nova Scotia Duck Tolling Retriever	NSDT	1549	XVIII	138,540
	Curly-coated Retriever	CCRT			
	Irish Water Spaniel	IWSP			
	Newfoundland	NEWF			
	Labrador Retriever	LAB			
	Golden Retriever	GOLD			
	Flat-coated Retriever	FCR			
Pointer Setter	Dalmatian	DALM	514	XIX	139,813
	Weimaraner	WEIM			
	Large Munsterlander	LMUN			
	Vizsla	VIZS			
	Wirehaired Pointing Griffon	WHPG			
	German Wirehaired Pointer	GWHP			
	German Shorthaired Pointer	GSHP			
	Spinone Italiano	SPIN			
	Brittany	BRIT			
	Irish Setter	ISET			
	English Setter	ESET			

	Gordon Setter	GORD			
UK Rural	Italian Greyhound	ITGY	1190	XX	141,175
	Borzoi	BORZ			
	Irish Wolfhound	IWOF			
	Scottish Deerhound	DEER			
	Whippet	WHIP			
	Greyhound	GREY			
	Old English Sheepdog	OES			
	Australian Cattle Dog	AUCD			
	Bearded Collie	BERD			
	Kelpie	KELP			
	Border Collie	BORD			
	Pembroke Welsh Corgi	PEMB			
	Cardigan Welsh Corgi	CARD			
	Australian Shepherd	AUSS			
	Collie	COLL			
	Shetland Sheepdog	SSHP			
Alpine	Greater Swiss Mountain Dog	GSMD	108	XXI	132,069
	Bernese Mountain Dog	BMD			
	Leonberger	LEON			
	Saint Bernard	STBD			
European Mastiff	Rhodesian Ridgeback	RHOD	519	XXII	141,302
	Great Dane	DANE			
	Cane Corso - COO	ITCC			
	Cane Corso - USA	CANE			
	Neapolitan Mastiff	NEAP			
	Boerboel	BOER			
	English Mastiff	MAST			
	American Staffordshire Terrier	AMST			
	Staffordshire Bull Terrier	STAF			
	Miniature Bull Terrier	MBLT			
	Bull Terrier	BULT			
	French Bulldog	FBUL			
	Boston Terrier	BOST			
	Dogue de Bordeaux	DDBX			
	Bulldog	BULD			
	Boxer	BOX			

SNP: single nucleotide polymorphism; *QC = SNPs retained after filtering for different inclusion criteria described in methods section.

Supplementary Table S2 The list of GO terms, KEGG pathways and the associated genes in either of two pairwise comparisons (Bullmastiff vs Bulldog and Bullmastiff vs Mastiff)

Gene Ontology (GO) terms and KEGG pathways analysis using genes identified in BullMastiff vs Mastiff					
KEGG(Term)		Count	PValue	Genes	Fold Enrichment
cfa04974	Protein digestion and absorption	5	0.02	COL5A1, COL4A3, COL6A6, LOC610614, COL6A5	5.02
cfa04512	ECM-receptor interaction	5	0.02	COL5A1, COL4A3, COL6A6, LOC610614, COL6A5	4.85
cfa05231	Choline metabolism in cancer	5	0.03	MAP2K2, PIK3CD, TSC1, PIP5K1C, RALGDS	4.26
cfa00601	Glycosphingolipid biosynthesis - lacto and neolacto series	3	0.04	FUT5, FUT7, ABO	9.38
cfa04151	PI3K-Akt signaling pathway	9	0.04	RXRA, MAP2K2, COL5A1, COL4A3, PIK3CD, COL6A6, TSC1, LOC610614, COL6A5	2.25
cfa04919	Thyroid hormone signaling pathway	5	0.04	NOTCH1, RXRA, MAP2K2, PIK3CD, MED27	3.73
cfa04920	Adipocytokine signaling pathway	4	0.05	RXRA, LOC485024, LOC476732, TRAF2	4.75
cfa01100	Metabolic pathways	21	0.05	H6PD, NDUFA11, LOC476732, DBH, ENO1, CEL, AGPAT2, ABO, AK8, GBGT1, GALC, FUT5, FUT7, NMNAT1, GMDS, INPP5E, LOC485024, SARDH, PIP5K1C, PTGDS, LOC480667	1.49
cfa05222	Small cell lung cancer	4	0.07	RXRA, COL4A3, PIK3CD, TRAF2	4.07
cfa04510	Focal adhesion	6	0.09	COL5A1, COL4A3, PIK3CD, COL6A6, LOC610614, COL6A5	2.45
Biological Process (GO Term)		Count	PValue	Genes	Fold Enrichment
GO:0072531	pyrimidine-containing compound transmembrane transport	3	0.00	LOC486150, SLC19A3, SLC25A33	39.32
GO:0031323	regulation of cellular metabolic process	71	0.01	BARHL1, TCF25, GPR65, HTR2B, HNRNPU, ENO1, IKBKAP, MYDGF, NPPC, CNST, ATCAY, CHMP1A, SNAPC4, WDR5, HMG20B, NELFB, NUDT16, PIAS4, MAP2K2, DAPK3, RFX2, TRAF2, SIRT6, TSC1, TFB2M, TICAM1, SAFB, MED27, SPIDR, RAD23B, DUSP22, NCL, COL4A3, DPEP1, CACTIN, PLIN5, NOTCH1, CAB39, UHRF1, PIK3R4, FZR1, RXRA, FLRT2,	1.32

				NACC2, SH3BP5, SAFB2, LRRC4C, ZC3H14, PPP1R26, ABCA2, ZNF462, KDM4B, FOXF2, CCL20, GPR55, CARD9, EAF1, FANCA, EEF2, LOC479600, RAX2, GRIN1, SETX, GFI1B, LHX3, NFIC, CDK10, TRIP12, SLC25A33, ITM2C, PTPN3	
GO:0019222	regulation of metabolic process	75	0.01	BARHL1, TCF25, GPR65, HTR2B, PIK3CD, HNRNPU, ENO1, IKBKAP, MYDGF, EPB41L4B, NPPC, CNST, ATCAY, CHMP1A, SNAPC4, WDR5, PSMD1, HMG20B, NELFB, NUDT16, PIAS4, MAP2K2, DAPK3, RFX2, TRAF2, SIRT6, TSC1, TFB2M, TICAM1, SAFB, MED27, SPIDR, RAD23B, DUSP22, NCL, COL4A3, DPEP1, CACTIN, PLIN5, NOTCH1, CAB39, UHRF1, PIK3R4, FZR1, RXRA, FLRT2, NACC2, SH3BP5, SAFB2, LRRC4C, ZC3H14, PPP1R26, ABCA2, ZNF462, KDM4B, FOXF2, CCL20, GPR55, CARD9, EAF1, FANCA, EEF2, LOC479600, RAX2, GRIN1, SETX, GFI1B, LHX3, NFIC, CDK10, TRIP12, APBA3, SLC25A33, ITM2C, PTPN3	1.30
GO:0008152	metabolic process	127	0.01	GPR65, HNRNPU, ENO1, TTF1, NPPC, CNST, ATCAY, CAPN7, PSMD1, PMPCA, NUDT16, PTGDS, LOC480667, ACAD11, PIAS4, MAP2K2, FBXW5, ENTPD2, DAPK3, RFX2, MATK, UBE4B, HDGFRP2, TSC1, CEL, TICAM1, GBGT1, NCL, COL4A3, LOC485024, DPEP1, PHPT1, NOTCH1, H6PD, UBA5, PIK3R4, COPS7B, ABO, AK8, FUT5, FUT7, DPP7, ADAMTS13, NDOR1, FLRT2, SOHLH1, LCN15, NACC2, INPP5E, SAFB2, LRRC4C, ZC3H14, PPP1R26, LOC607011, ABCA2, SURF1, FOXF2, EAF1, FANCA, EEF2, LOC100684996, RAX2, SETX, LHX3, COL5A1, CDK10, ITM2C, BARHL1, TCF25, LOC607002, HTR2B, PIK3CD, DBH, IKBKAP, MYDGF, MRPL3, EPB41L4B, RPL7A, DNER, CHMP1A, SNAPC4, WDR5, CA6, HMG20B, NELFB, DIS3L2, TRAF2, SIRT6, TFB2M, SAFB, MED27, RAD23B, SPIDR, TMEM210, DUSP22, PLIN4, ACPP, CACTIN, PLIN5, CAB39, UHRF1, GLT6D1, SPG7, AGPAT2, FZR1, RXRA, GMDS, SH3BP5, RPL13, ZNF462, KDM4B, CCL20, GPR55, CARD9, LOC476732, DOHH, LOC479600, GRIN1, GALC, GFI1B, NMNAT1, NFIC, TRIP12, APBA3, SLC25A33, NEK11, PTPN3	1.16
GO:0006753	nucleoside phosphate metabolic process	14	0.01	SURF1, H6PD, ENTPD2, GPR65, HTR2B, SIRT6, ENO1, LOC100684996, AK8, NPPC, NMNAT1, NUDT16, ACPP, SLC25A33	2.34
GO:0006928	movement of cell or subcellular component	30	0.01	BARHL1, RERE, NOTCH1, SSNA1, HTR2B, PIK3CD, DBH, IKBKAP, SPG7, TMEM141, EPB41L4B, TMEM201, FUT7, FLRT2, CAPN7, KIF1B, NRTN, SEMA6B, MAP2K2, CCL20,	1.64

				DAPK3, MATK, TTC8, LHX3, COL5A1, CATSPERD, KIF26B, DPEP1, PHPT1, GAS8	
GO:2000779	regulation of double-strand break repair	4	0.01	SIRT6, TRIP12, NUDT16, SPIDR	9.04
GO:0040011	locomotion	27	0.01	BARHL1, RERE, NOTCH1, HTR2B, PIK3CD, DBH, IKBKAP, EPB41L4B, TMEM201, FUT7, FLRT2, CAPN7, NRTN, SEMA6B, MAP2K2, CCL20, DAPK3, MATK, TTC8, LHX3, COL5A1, CATSPERD, KIF26B, ACKR4, DPEP1, PHPT1, GAS8	1.67
GO:0080090	regulation of primary metabolic process	69	0.01	BARHL1, TCF25, GPR65, HTR2B, HNRNPU, ENO1, IKBKAP, MYDGF, NPPC, CHMP1A, SNAPC4, WDR5, PSMD1, HMG20B, NELFB, NUDT16, PIAS4, MAP2K2, DAPK3, RFX2, TRAF2, SIRT6, TSC1, TFB2M, TICAM1, SAFB, MED27, SPIDR, RAD23B, DUSP22, NCL, COL4A3, DPEP1, CACTIN, PLIN5, NOTCH1, CAB39, UHRF1, PIK3R4, FZR1, RXRA, FLRT2, NACC2, SH3BP5, SAFB2, LRRC4C, ZC3H14, ABCA2, ZNF462, KDM4B, FOXF2, CCL20, GPR55, CARD9, EAF1, FANCA, EEF2, LOC479600, RAX2, GRIN1, SETX, GFI1B, LHX3, NFIC, CDK10, TRIP12, SLC25A33, ITM2C, PTPN3	1.29
GO:0019438	aromatic compound biosynthetic process	52	0.01	BARHL1, TCF25, GPR65, HTR2B, HNRNPU, DBH, ENO1, IKBKAP, TTF1, MYDGF, NPPC, CHMP1A, SNAPC4, WDR5, NELFB, PIAS4, RFX2, TRAF2, HDGFRP2, SIRT6, TFB2M, TICAM1, SAFB, MED27, DUSP22, NCL, CACTIN, NOTCH1, UHRF1, AK8, RXRA, SOHLH1, GMDS, NACC2, SAFB2, LOC607011, ABCA2, ZNF462, SURF1, FOXF2, EAF1, FANCA, LOC100684996, LOC479600, RAX2, GRIN1, SETX, GFI1B, NMNAT1, LHX3, NFIC, SLC25A33	1.37
GO:0034654	nucleobase-containing compound biosynthetic process	51	0.01	BARHL1, TCF25, GPR65, HTR2B, HNRNPU, ENO1, IKBKAP, TTF1, MYDGF, NPPC, CHMP1A, SNAPC4, WDR5, NELFB, PIAS4, RFX2, TRAF2, HDGFRP2, SIRT6, TFB2M, TICAM1, SAFB, MED27, DUSP22, NCL, CACTIN, NOTCH1, UHRF1, AK8, RXRA, SOHLH1, GMDS, NACC2, SAFB2, LOC607011, ABCA2, ZNF462, SURF1, FOXF2, EAF1, FANCA, LOC100684996, LOC479600, RAX2, GRIN1, SETX, GFI1B, NMNAT1, LHX3, NFIC, SLC25A33	1.38
GO:0055086	nucleobase-containing small molecule metabolic process	14	0.01	SURF1, H6PD, ENTPD2, GPR65, HTR2B, SIRT6, ENO1, LOC100684996, AK8, NPPC, NMNAT1, NUDT16, ACPP, SLC25A33	2.12
GO:0071704	organic substance metabolic process	121	0.02	GPR65, HNRNPU, ENO1, TTF1, NPPC, ATCAY, CAPN7, PSMD1, PMPCA, NUDT16, PTGDS, LOC480667, ACAD11,	1.15

				PIAS4, MAP2K2, FBXW5, ENTPD2, DAPK3, RFX2, MATK, UBE4B, HDGFRP2, TSC1, CEL, TICAM1, GBGT1, NCL, COL4A3, DPEP1, PHPT1, NOTCH1, H6PD, UBA5, PIK3R4, COPS7B, ABO, AK8, FUT5, FUT7, DPP7, ADAMTS13, FLRT2, SOHLH1, LCN15, NACC2, INPP5E, SAFB2, LRRC4C, ZC3H14, LOC607011, ABCA2, SURF1, FOXF2, EAF1, FANCA, EEF2, LOC100684996, RAX2, SETX, LHX3, COL5A1, CDK10, ITM2C, BARHL1, TCF25, LOC607002, HTR2B, PIK3CD, DBH, IKBKAP, MYDGF, MRPL3, EPB41L4B, RPL7A, DNER, CHMP1A, SNAPC4, WDR5, HMG20B, NELFB, DIS3L2, TRAF2, SIRT6, TFB2M, SAFB, MED27, RAD23B, SPIDR, TMEM210, DUSP22, PLIN4, ACPP, CACTIN, PLIN5, CAB39, UHRF1, GLT6D1, SPG7, AGPAT2, FZR1, RXRA, GMDS, SH3BP5, RPL13, ZNF462, KDM4B, CCL20, GPR55, CARD9, DOHH, LOC479600, GRIN1, GALC, GFI1B, NMNAT1, NFIC, TRIP12, APBA3, SLC25A33, NEK11, PTPN3	
GO:0018130	heterocycle biosynthetic process	51	0.02	BARHL1, TCF25, GPR65, HTR2B, HNRNPU, ENO1, IKBKAP, TTF1, MYDGF, NPPC, CHMP1A, SNAPC4, WDR5, NELFB, PIAS4, RFX2, TRAF2, HDGFRP2, SIRT6, TFB2M, TICAM1, SAFB, MED27, DUSP22, NCL, CACTIN, NOTCH1, UHRF1, AK8, RXRA, SOHLH1, GMDS, NACC2, SAFB2, LOC607011, ABCA2, ZNF462, SURF1, FOXF2, EAF1, FANCA, LOC100684996, LOC479600, RAX2, GRIN1, SETX, GFI1B, NMNAT1, LHX3, NFIC, SLC25A33	1.35
GO:1901362	organic cyclic compound biosynthetic process	52	0.02	BARHL1, TCF25, GPR65, HTR2B, HNRNPU, DBH, ENO1, IKBKAP, TTF1, MYDGF, NPPC, CHMP1A, SNAPC4, WDR5, NELFB, PIAS4, RFX2, TRAF2, HDGFRP2, SIRT6, TFB2M, TICAM1, SAFB, MED27, DUSP22, NCL, CACTIN, NOTCH1, UHRF1, AK8, RXRA, SOHLH1, GMDS, NACC2, SAFB2, LOC607011, ABCA2, ZNF462, SURF1, FOXF2, EAF1, FANCA, LOC100684996, LOC479600, RAX2, GRIN1, SETX, GFI1B, NMNAT1, LHX3, NFIC, SLC25A33	1.34
GO:0031175	neuron projection development	16	0.02	RERE, SEMA6B, NOTCH1, MAP2K2, UBE4B, CAMSAP1, GRIN1, SETX, TTC8, ATCAY, FLRT2, LHX3, KIF26B, NRTN, LRRC4C, ITM2C	1.95
GO:1901135	carbohydrate derivative metabolic process	20	0.02	LOC607011, SURF1, H6PD, GPR65, LOC607002, HTR2B, SIRT6, ENO1, LOC100684996, GBGT1, GALC, FUT5, NPPC, FUT7, GMDS, NUDT16, ACPP, LOC480667, SLC25A33, ITM2C	1.75

GO:0044271	cellular nitrogen compound biosynthetic process	58	0.02	BARHL1, TCF25, GPR65, HTR2B, HNRNPU, DBH, ENO1, IKBKAP, TTF1, MYDGF, RPL7A, NPPC, MRPL3, CHMP1A, SNAPC4, WDR5, NELFB, PIAS4, DAPK3, RFX2, TRAF2, HDGFRP2, SIRT6, TSC1, TFB2M, TICAM1, SAFB, MED27, DUSP22, NCL, CACTIN, NOTCH1, UHRF1, AK8, RXRA, SOHLH1, GMDS, NACC2, SAFB2, RPL13, LOC607011, ABCA2, ZNF462, SURF1, FOXF2, EAF1, FANCA, EEF2, LOC100684996, LOC479600, RAX2, GRIN1, SETX, GFI1B, NMNAT1, LHX3, NFIC, SLC25A33	1.30
GO:0031324	negative regulation of cellular metabolic process	31	0.02	UHRF1, TCF25, HNRNPU, ENO1, CNST, RXRA, ATCAY, FLRT2, NACC2, CHMP1A, SH3BP5, HMG20B, NELFB, LRRC4C, ZC3H14, PPP1R26, PIAS4, KDM4B, FOXF2, DAPK3, SIRT6, TSC1, LOC479600, DUSP22, NFIC, DPEP1, TRIP12, CACTIN, ITM2C, PTPN3, PLIN5	1.49
GO:0019637	organophosphate metabolic process	17	0.03	SURF1, H6PD, ENTPD2, GPR65, PIK3R4, HTR2B, SIRT6, ENO1, LOC100684996, AGPAT2, AK8, NPPC, NMNAT1, INPP5E, NUDT16, ACPP, SLC25A33	1.80
GO:1901137	carbohydrate derivative biosynthetic process	14	0.03	LOC607011, SURF1, GPR65, LOC607002, HTR2B, SIRT6, LOC100684996, GBGT1, FUT5, NPPC, FUT7, GMDS, LOC480667, ITM2C	1.96
GO:0044249	cellular biosynthetic process	69	0.03	BARHL1, TCF25, LOC607002, GPR65, HTR2B, HNRNPU, DBH, ENO1, IKBKAP, TTF1, MYDGF, RPL7A, NPPC, MRPL3, CHMP1A, SNAPC4, WDR5, NELFB, PTGDS, LOC480667, PIAS4, DAPK3, RFX2, TRAF2, HDGFRP2, SIRT6, TSC1, TFB2M, TICAM1, SAFB, MED27, GBGT1, DUSP22, NCL, CACTIN, PLIN5, NOTCH1, UHRF1, AGPAT2, AK8, FUT5, FUT7, RXRA, NDOR1, SOHLH1, GMDS, NACC2, SAFB2, RPL13, LOC607011, ABCA2, ZNF462, SURF1, FOXF2, CARD9, EAF1, FANCA, EEF2, LOC100684996, LOC479600, RAX2, GRIN1, SETX, GFI1B, NMNAT1, LHX3, NFIC, SLC25A33, ITM2C	1.24
GO:0051674	localization of cell	23	0.03	RERE, SEMA6B, BARHL1, NOTCH1, MAP2K2, CCL20, DAPK3, HTR2B, MATK, PIK3CD, DBH, IKBKAP, EPB41L4B, TMEM201, FUT7, FLRT2, COL5A1, CAPN7, CATSPERD, DPEP1, NRTN, PHPT1, GAS8	1.60
GO:0048870	cell motility	23	0.03	RERE, SEMA6B, BARHL1, NOTCH1, MAP2K2, CCL20, DAPK3, HTR2B, MATK, PIK3CD, DBH, IKBKAP, EPB41L4B, TMEM201, FUT7, FLRT2, COL5A1, CAPN7, CATSPERD, DPEP1, NRTN, PHPT1, GAS8	1.60
GO:0009058	biosynthetic process	71	0.03	BARHL1, TCF25, LOC607002, GPR65, HTR2B, HNRNPU, DBH, ENO1, IKBKAP, TTF1, MYDGF, RPL7A, NPPC, MRPL3,	1.23

				CHMP1A, SNAPC4, WDR5, NELFB, PTGDS, LOC480667, PIAS4, DAPK3, RFX2, TRAF2, HDGFRP2, SIRT6, TSC1, TFB2M, TICAM1, SAFB, MED27, GBGT1, DUSP22, NCL, CACTIN, PLIN5, NOTCH1, UHRF1, AGPAT2, AK8, FUT5, FUT7, RXRA, NDOR1, SOHLH1, GMDS, NACC2, SAFB2, RPL13, LOC607011, ABCA2, ZNF462, SURF1, FOXF2, CARD9, EAF1, FANCA, DOHH, EEF2, LOC100684996, LOC479600, RAX2, GRIN1, SETX, GFI1B, NMNAT1, COL5A1, LHX3, NFIC, SLC25A33, ITM2C	
GO:0060255	regulation of macromolecule metabolic process	67	0.03	BARHL1, TCF25, HTR2B, PIK3CD, HNRNPU, ENO1, IKBKAP, MYDGF, EPB41L4B, CHMP1A, SNAPC4, WDR5, PSMD1, HMG20B, NELFB, NUDT16, PIAS4, MAP2K2, DAPK3, RFX2, TRAF2, SIRT6, TSC1, TFB2M, TICAM1, SAFB, MED27, SPIDR, RAD23B, DUSP22, NCL, COL4A3, DPEP1, CACTIN, NOTCH1, CAB39, UHRF1, FZR1, RXRA, FLRT2, NACC2, SH3BP5, SAFB2, LRRC4C, ZC3H14, ABCA2, ZNF462, KDM4B, FOXF2, CCL20, GPR55, CARD9, EAF1, FANCA, EEF2, LOC479600, RAX2, GRIN1, SETX, GFI1B, LHX3, NFIC, CDK10, TRIP12, APBA3, ITM2C, PTPN3	1.24
GO:2001020	regulation of response to DNA damage stimulus	6	0.03	PIAS4, NACC2, SIRT6, TRIP12, NUDT16, SPIDR	3.45
GO:0071934	thiamine transmembrane transport	2	0.03	LOC486150, SLC19A3	65.54
GO:0044238	primary metabolic process	113	0.03	GPR65, HNRNPU, ENO1, TTF1, NPPC, CAPN7, PSMD1, PMPCA, NUDT16, PTGDS, LOC480667, ACAD11, PIAS4, MAP2K2, FBXW5, ENTPD2, DAPK3, RFX2, MATK, UBE4B, HDGFRP2, TSC1, CEL, TICAM1, GBGT1, NCL, COL4A3, DPEP1, PHPT1, NOTCH1, H6PD, UBA5, PIK3R4, COPS7B, ABO, AK8, FUT5, FUT7, DPP7, FLRT2, SOHLH1, LCN15, NACC2, INPP5E, SAFB2, LRRC4C, ZC3H14, LOC607011, ABCA2, SURF1, FOXF2, EAF1, FANCA, EEF2, LOC100684996, RAX2, SETX, LHX3, CDK10, ITM2C, BARHL1, TCF25, LOC607002, HTR2B, IKBKAP, MYDGF, MRPL3, RPL7A, DNER, CHMP1A, SNAPC4, WDR5, HMG20B, NELFB, DIS3L2, TRAF2, SIRT6, TFB2M, SAFB, MED27, RAD23B, SPIDR, TMEM210, DUSP22, ACPP, CACTIN, PLIN5, CAB39, UHRF1, GLT6D1, SPG7, AGPAT2, FZR1, RXRA, GMDS, SH3BP5, RPL13, ZNF462, KDM4B, CCL20, GPR55, CARD9, DOHH, LOC479600, GRIN1,	1.14

				GALC, GFI1B, NMNAT1, NFIC, TRIP12, SLC25A33, NEK11, PTPN3	
GO:0009117	nucleotide metabolic process	12	0.03	NPPC, SURF1, H6PD, NMNAT1, GPR65, HTR2B, SIRT6, NUDT16, ENO1, ACPP, LOC100684996, SLC25A33	2.05
GO:0006351	transcription, DNA-templated	38	0.04	BARHL1, NOTCH1, UHRF1, TCF25, ENO1, IKBKAP, TTF1, MYDGF, RXRA, SOHLH1, NACC2, SNAPC4, CHMP1A, SAFB2, WDR5, NELFB, PIAS4, ABCA2, ZNF462, FOXF2, FANCA, RFX2, TRAF2, HDGFRP2, SIRT6, TFB2M, TICAM1, MED27, SAFB, LOC479600, GRIN1, DUSP22, SETX, LHX3, NFIC, NCL, CACTIN, SLC25A33	1.37
GO:0001938	positive regulation of endothelial cell proliferation	4	0.04	MYDGF, EGFL7, LRG1, HTR2B	5.46
GO:1901576	organic substance biosynthetic process	69	0.04	BARHL1, TCF25, LOC607002, GPR65, HTR2B, HNRNPU, DBH, ENO1, IKBKAP, TTF1, MYDGF, RPL7A, NPPC, MRPL3, CHMP1A, SNAPC4, WDR5, NELFB, PTGDS, LOC480667, PIAS4, DAPK3, RFX2, TRAF2, HDGFRP2, SIRT6, TSC1, TFB2M, TICAM1, SAFB, MED27, GBGT1, DUSP22, NCL, CACTIN, PLIN5, NOTCH1, UHRF1, AGPAT2, AK8, FUT5, FUT7, RXRA, SOHLH1, GMDS, NACC2, SAFB2, RPL13, LOC607011, ABCA2, ZNF462, SURF1, FOXF2, CARD9, EAF1, FANCA, EEF2, LOC100684996, LOC479600, RAX2, GRIN1, SETX, GFI1B, NMNAT1, COL5A1, LHX3, NFIC, SLC25A33, ITM2C	1.22
GO:0006796	phosphate-containing compound metabolic process	38	0.04	H6PD, CAB39, GPR65, PIK3R4, HTR2B, ENO1, AGPAT2, AK8, MYDGF, NPPC, CNST, FLRT2, INPP5E, SH3BP5, NUDT16, LRRC4C, PPP1R26, SURF1, MAP2K2, ENTPD2, CCL20, GPR55, CARD9, DAPK3, MATK, TRAF2, SIRT6, TSC1, LOC100684996, DUSP22, SETX, NMNAT1, CDK10, ACPP, PHPT1, CACTIN, SLC25A33, NEK11	1.37
GO:2000147	positive regulation of cell motility	10	0.04	SEMA6B, EPB41L4B, NOTCH1, MAP2K2, CCL20, CAPN7, DAPK3, PIK3CD, IKBKAP, PHPT1	2.21
GO:0044237	cellular metabolic process	114	0.04	GPR65, HNRNPU, ENO1, TTF1, NPPC, CNST, ATCAY, PSMD1, PMPCA, NUDT16, PTGDS, LOC480667, ACAD11, PIAS4, MAP2K2, FBXW5, ENTPD2, DAPK3, RFX2, MATK, UBE4B, HDGFRP2, TSC1, CEL, TICAM1, GBGT1, NCL, COL4A3, DPEP1, PHPT1, NOTCH1, H6PD, UBA5, PIK3R4, COPS7B, AK8, FUT5, FUT7, ADAMTS13, NDOR1, FLRT2, SOHLH1, NACC2, INPP5E, SAFB2, LRRC4C, ZC3H14, PPP1R26, LOC607011, ABCA2, SURF1, FOXF2, EAF1, FANCA, EEF2, LOC100684996, RAX2, SETX, LHX3, CDK10, ITM2C, BARHL1, TCF25,	1.13

				LOC607002, HTR2B, DBH, IKBKAP, MYDGF, MRPL3, RPL7A, DNER, CHMP1A, SNAPC4, WDR5, CA6, HMG20B, NELFB, DIS3L2, TRAF2, SIRT6, TFB2M, SAFB, MED27, RAD23B, SPIDR, TMEM210, DUSP22, ACPP, CACTIN, PLIN5, CAB39, UHRF1, AGPAT2, FZR1, RXRA, GMDS, SH3BP5, RPL13, ZNF462, KDM4B, CCL20, GPR55, CARD9, DOHH, LOC479600, GRIN1, GALC, GFI1B, NMNAT1, NFIC, TRIP12, SLC25A33, NEK11, PTPN3	
GO:0006793	phosphorus metabolic process	38	0.04	H6PD, CAB39, GPR65, PIK3R4, HTR2B, ENO1, AGPAT2, AK8, MYDGF, NPPC, CNST, FLRT2, INPP5E, SH3BP5, NUDT16, LRRC4C, PPP1R26, SURF1, MAP2K2, ENTPD2, CCL20, GPR55, CARD9, DAPK3, MATK, TRAF2, SIRT6, TSC1, LOC100684996, DUSP22, SETX, NMNAT1, CDK10, ACPP, PHPT1, CACTIN, SLC25A33, NEK11	1.36
GO:0051272	positive regulation of cellular component movement	10	0.04	SEMA6B, EPB41L4B, NOTCH1, MAP2K2, CCL20, CAPN7, DAPK3, PIK3CD, IKBKAP, PHPT1	2.16
GO:0051171	regulation of nitrogen compound metabolic process	49	0.04	BARHL1, TCF25, GPR65, HNRNPU, ENO1, IKBKAP, MYDGF, NPPC, ATCAY, CHMP1A, SNAPC4, WDR5, NELFB, NUDT16, PIAS4, DAPK3, RFX2, TRAF2, SIRT6, TSC1, TFB2M, TICAM1, SAFB, MED27, SPIDR, DUSP22, NCL, CACTIN, NOTCH1, UHRF1, RXRA, NACC2, SAFB2, ZC3H14, ABCA2, ZNF462, FOXF2, EAF1, FANCA, EEF2, LOC479600, RAX2, GRIN1, SETX, GFI1B, LHX3, NFIC, TRIP12, SLC25A33	1.28
GO:2001022	positive regulation of response to DNA damage stimulus	4	0.04	PIAS4, NACC2, NUDT16, SPIDR	5.04
GO:0015888	thiamine transport	2	0.04	LOC486150, SLC19A3	43.69
GO:0060528	secretory columnal luminar epithelial cell differentiation involved in prostate glandular acinus development	2	0.04	NOTCH1, RXRA	43.69
GO:0040017	positive regulation of locomotion	10	0.05	SEMA6B, EPB41L4B, NOTCH1, MAP2K2, CCL20, CAPN7, DAPK3, PIK3CD, IKBKAP, PHPT1	2.13
GO:0019693	ribose phosphate metabolic process	10	0.05	NPPC, SURF1, H6PD, GPR65, HTR2B, SIRT6, NUDT16, ENO1, LOC100684996, SLC25A33	2.13
GO:0009179	purine ribonucleoside	4	0.05	ENTPD2, SIRT6, NUDT16, ENO1	4.95

	diphosphate metabolic process				
GO:0009135	purine nucleoside diphosphate metabolic process	4	0.05	ENTPD2, SIRT6, NUDT16, ENO1	4.95
GO:0009892	negative regulation of metabolic process	31	0.05	UHRF1, TCF25, HNRNPU, ENO1, CNST, RXRA, ATCAY, FLRT2, NACC2, CHMP1A, SH3BP5, HMG20B, NELFB, LRRC4C, ZC3H14, PPP1R26, PIAS4, KDM4B, FOXF2, DAPK3, SIRT6, TSC1, LOC479600, DUSP22, NFIC, DPEP1, TRIP12, CACTIN, ITM2C, PTPN3, PLIN5	1.40
GO:0009185	ribonucleoside diphosphate metabolic process	4	0.05	ENTPD2, SIRT6, NUDT16, ENO1	4.77
GO:2000241	regulation of reproductive process	5	0.05	NPPC, FZR1, NOTCH1, RXRA, PTGDS	3.56
GO:0006282	regulation of DNA repair	4	0.06	SIRT6, TRIP12, NUDT16, SPIDR	4.60
GO:0035461	vitamin transmembrane transport	2	0.06	LOC486150, SLC19A3	32.77
GO:0009181	purine ribonucleoside diphosphate catabolic process	2	0.06	ENTPD2, NUDT16	32.77
GO:0009137	purine nucleoside diphosphate catabolic process	2	0.06	ENTPD2, NUDT16	32.77
GO:0006357	regulation of transcription from RNA polymerase II promoter	25	0.06	BARHL1, NOTCH1, UHRF1, TCF25, IKBKAP, MYDGF, RXRA, NACC2, SNAPC4, SAFB2, WDR5, NELFB, PIAS4, ABCA2, ZNF462, RFX2, MED27, SAFB, LOC479600, GRIN1, DUSP22, SETX, LHX3, NFIC, NCL	1.44
GO:0009889	regulation of biosynthetic process	47	0.06	BARHL1, NOTCH1, UHRF1, TCF25, GPR65, HTR2B, HNRNPU, ENO1, IKBKAP, MYDGF, RXRA, NACC2, SNAPC4, CHMP1A, SAFB2, WDR5, NELFB, PIAS4, ABCA2, ZNF462, FOXF2, CARD9, DAPK3, EAF1, FANCA, RFX2, TRAF2, SIRT6, TSC1, TFB2M, TICAM1, MED27, EEF2, SAFB, LOC479600, RAX2, GRIN1, DUSP22, SETX, GFI1B, LHX3, NFIC, NCL, CACTIN, SLC25A33, ITM2C, PLIN5	1.26
GO:0019219	regulation of nucleobase-	45	0.07	BARHL1, NOTCH1, UHRF1, TCF25, GPR65, HNRNPU, ENO1, IKBKAP, MYDGF, NPPC, RXRA, NACC2, SNAPC4, CHMP1A,	1.26

	containing compound metabolic process			SAFB2, WDR5, NELFB, NUDT16, ZC3H14, PIAS4, ABCA2, ZNF462, FOXF2, EAF1, FANCA, RFX2, TRAF2, SIRT6, TFB2M, TICAM1, MED27, SAFB, SPIDR, LOC479600, RAX2, GRIN1, DUSP22, SETX, GFI1B, LHX3, NFIC, NCL, TRIP12, CACTIN, SLC25A33	
GO:0003018	vascular process in circulatory system	5	0.07	NPPC, TBXA2R, HTR2B, DBH, LOC479600	3.21
GO:0048666	neuron development	16	0.07	RERE, SEMA6B, NOTCH1, MAP2K2, UBE4B, CAMSAP1, GRIN1, SETX, TTC8, ATCAY, FLRT2, LHX3, KIF26B, NRTN, LRRC4C, ITM2C	1.61
GO:0072521	purine-containing compound metabolic process	10	0.07	NPPC, SURF1, GPR65, HTR2B, SIRT6, NUDT16, ENO1, ACPP, LOC100684996, SLC25A33	1.95
GO:0009132	nucleoside diphosphate metabolic process	4	0.07	ENTPD2, SIRT6, NUDT16, ENO1	4.16
GO:0031326	regulation of cellular biosynthetic process	46	0.07	BARHL1, NOTCH1, UHRF1, TCF25, GPR65, HTR2B, HNRNPU, ENO1, IKBKAP, MYDGF, RXRA, NACC2, SNAPC4, CHMP1A, SAFB2, WDR5, NELFB, PIAS4, ABCA2, ZNF462, FOXF2, CARD9, DAPK3, EAF1, FANCA, RFX2, TRAF2, SIRT6, TSC1, TFB2M, TICAM1, MED27, EEF2, SAFB, LOC479600, RAX2, GRIN1, DUSP22, SETX, GFI1B, LHX3, NFIC, NCL, CACTIN, ITM2C, PLIN5	1.25
GO:1904587	response to glycoprotein	2	0.07	LOC607002, LOC480667	26.21
GO:0097466	glycoprotein ERAD pathway	2	0.07	LOC607002, LOC480667	26.21
GO:1901293	nucleoside phosphate biosynthetic process	7	0.08	NPPC, SURF1, NMNAT1, GPR65, HTR2B, LOC100684996, AK8	2.36
GO:0044092	negative regulation of molecular function	14	0.08	PPP1R26, PIAS4, ZNF462, LOC479600, TTC8, CNST, FLRT2, PDE6D, SH3BP5, DPEP1, PHPT1, CACTIN, LRRC4C, PLIN5	1.67
GO:0009150	purine ribonucleotide metabolic process	9	0.08	NPPC, SURF1, GPR65, HTR2B, SIRT6, NUDT16, ENO1, LOC100684996, SLC25A33	2.02
GO:0030030	cell projection organization	20	0.08	RERE, SEMA6B, NOTCH1, MAP2K2, SSNA1, RFX2, UBE4B, TSC1, TMEM141, CAMSAP1, GRIN1, SETX, TTC8, ATCAY, FLRT2, LHX3, KIF26B, NRTN, LRRC4C, ITM2C	1.49
GO:0001936	regulation of endothelial cell proliferation	4	0.08	MYDGF, EGFL7, LRG1, HTR2B	3.86

GO:0009259	ribonucleotide metabolic process	9	0.09	NPPC, SURF1, GPR65, HTR2B, SIRT6, NUDT16, ENO1, LOC100684996, SLC25A33	1.97
GO:0030198	extracellular matrix organization	6	0.09	NOTCH1, FLRT2, COL5A1, FOXF2, COL4A3, ADAMTSL2	2.55
GO:0043062	extracellular structure organization	6	0.09	NOTCH1, FLRT2, COL5A1, FOXF2, COL4A3, ADAMTSL2	2.54
GO:0006487	protein N-linked glycosylation	4	0.09	FUT5, FUT7, LOC607002, LOC480667	3.80
GO:0030705	cytoskeleton-dependent intracellular transport	4	0.09	TMEM201, KIF26B, KIF1B, SPG7	3.80
GO:1902231	positive regulation of intrinsic apoptotic signaling pathway in response to DNA damage	2	0.09	PIAS4, NACC2	21.85
GO:0009191	ribonucleoside diphosphate catabolic process	2	0.09	ENTPD2, NUDT16	21.85
GO:0007202	activation of phospholipase C activity	2	0.09	GPR55, HTR2B	21.85
GO:0050679	positive regulation of epithelial cell proliferation	5	0.09	MYDGF, EGFL7, NOTCH1, LRG1, HTR2B	2.95
GO:0044267	cellular protein metabolic process	55	0.09	LOC607002, HTR2B, MYDGF, RPL7A, MRPL3, DNER, WDR5, PSMD1, HMG20B, PMPCA, LOC480667, PIAS4, MAP2K2, FBXW5, DAPK3, MATK, TRAF2, SIRT6, TSC1, UBE4B, TICAM1, RAD23B, GBGT1, TMEM210, DUSP22, COL4A3, DPEP1, CACTIN, PHPT1, CAB39, UHRF1, UBA5, PIK3R4, COPS7B, FUT5, FZR1, FUT7, RXRA, FLRT2, SH3BP5, RPL13, LRRC4C, KDM4B, CCL20, GPR55, CARD9, DOHH, EEF2, SETX, GFI1B, CDK10, TRIP12, SLC25A33, PTPN3, NEK11	1.20
GO:0051301	cell division	7	0.09	SDCCAG3, AHCTF1, NOTCH1, CHMP1A, PIK3R4, HTR2B, DIS3L2	2.26
GO:0055010	ventricular cardiac muscle tissue morphogenesis	3	0.09	NOTCH1, RXRA, UBE4B	5.96

GO:0016477	cell migration	19	0.09	RERE, SEMA6B, BARHL1, NOTCH1, CCL20, DAPK3, HTR2B, MATK, PIK3CD, DBH, IKBKAP, EPB41L4B, TMEM201, FUT7, FLRT2, COL5A1, CAPN7, DPEP1, NRTN	1.48
GO:0019538	protein metabolic process	59	0.09	LOC607002, HTR2B, MYDGF, RPL7A, MRPL3, CAPN7, DNER, WDR5, PSMD1, HMG20B, PMPCA, LOC480667, PIAS4, MAP2K2, FBXW5, DAPK3, MATK, TRAF2, SIRT6, TSC1, UBE4B, TICAM1, RAD23B, GBGT1, TMEM210, DUSP22, COL4A3, DPEP1, CACTIN, PHPT1, CAB39, UHRF1, UBA5, PIK3R4, COPS7B, SPG7, FUT5, FZR1, FUT7, RXRA, DPP7, FLRT2, SH3BP5, RPL13, LRRC4C, KDM4B, CCL20, GPR55, CARD9, DOHH, EEF2, SETX, GFI1B, CDK10, TRIP12, SLC25A33, ITM2C, PTPN3, NEK11	1.19
GO:0097659	nucleic acid-templated transcription	41	0.09	BARHL1, NOTCH1, UHRF1, TCF25, ENO1, IKBKAP, TTF1, MYDGF, RXRA, SOHLH1, NACC2, SNAPC4, CHMP1A, SAFB2, WDR5, NELFB, PIAS4, ABCA2, ZNF462, FOXF2, EAF1, FANCA, RFX2, TRAF2, HDGFRP2, SIRT6, TFB2M, TICAM1, MED27, SAFB, LOC479600, RAX2, GRIN1, DUSP22, SETX, GFI1B, LHX3, NFIC, NCL, CACTIN, SLC25A33	1.25
GO:0032774	RNA biosynthetic process	41	0.10	BARHL1, NOTCH1, UHRF1, TCF25, ENO1, IKBKAP, TTF1, MYDGF, RXRA, SOHLH1, NACC2, SNAPC4, CHMP1A, SAFB2, WDR5, NELFB, PIAS4, ABCA2, ZNF462, FOXF2, EAF1, FANCA, RFX2, TRAF2, HDGFRP2, SIRT6, TFB2M, TICAM1, MED27, SAFB, LOC479600, RAX2, GRIN1, DUSP22, SETX, GFI1B, LHX3, NFIC, NCL, CACTIN, SLC25A33	1.24
GO:0010605	negative regulation of macromolecule metabolic process	27	0.10	UHRF1, TCF25, HNRNPU, ENO1, RXRA, FLRT2, NACC2, CHMP1A, SH3BP5, HMG20B, NELFB, LRRC4C, ZC3H14, PIAS4, KDM4B, FOXF2, DAPK3, SIRT6, TSC1, LOC479600, DUSP22, NFIC, DPEP1, TRIP12, CACTIN, ITM2C, PTPN3	1.34
Molecular functions (GO Terms)		Count	PValue	Genes	Fold Enrichment
GO:0030234	enzyme regulator activity	18	0.01	PPP1R26, GPSM1, NOTCH1, CAB39, HTR2B, TSC1, IKBKAP, GALC, FZR1, FLRT2, PDE6D, SH3BP5, PSMD1, DPEP1, LOC610614, APBA3, LRRC4C, RALGDS	1.89
GO:0016836	hydro-lyase activity	4	0.02	GMDS, UBA5, CA6, ENO1	6.67
GO:0050662	coenzyme binding	7	0.02	NDOR1, H6PD, GMDS, LOC608697, SIRT6, ACAD11, DUS3L	3.17
GO:0019887	protein kinase regulator activity	6	0.03	GALC, FLRT2, CAB39, SH3BP5, IKBKAP, LRRC4C	3.36
GO:0004857	enzyme inhibitor activity	9	0.03	PPP1R26, NOTCH1, FLRT2, PDE6D, SH3BP5, DPEP1, LOC610614, APBA3, LRRC4C	2.41

GO:0098772	molecular function regulator	21	0.04	PPP1R26, GPSM1, LRRC26, NOTCH1, CAB39, HTR2B, TSC1, IKBKAP, GALC, FZR1, FLRT2, PDE6D, SH3BP5, PSMD1, DPEP1, LOC610614, PHPT1, APBA3, LRRC4C, RALGDS, PTPN3	1.59
GO:0015563	uptake transmembrane transporter activity	2	0.04	LOC486150, SLC19A3	45.56
GO:0015403	thiamine uptake transmembrane transporter activity	2	0.04	LOC486150, SLC19A3	45.56
GO:0015234	thiamine transmembrane transporter activity	2	0.04	LOC486150, SLC19A3	45.56
GO:0016835	carbon-oxygen lyase activity	4	0.05	GMDS, UBA5, CA6, ENO1	4.97
GO:0019207	kinase regulator activity	6	0.05	GALC, FLRT2, CAB39, SH3BP5, IKBKAP, LRRC4C	3.04
GO:0043565	sequence-specific DNA binding	19	0.06	RERE, BARHL1, NOTCH1, FOXF2, RFX2, EDF1, SAFB, TTF1, RAX2, SETX, RXRA, LHX3, SOHLH1, NFIC, NACC2, NCL, SNAPC4, SAFB2, ZBTB7A	1.56
GO:0046920	alpha-(1->3)-fucosyltransferase activity	2	0.07	FUT5, FUT7	27.34
GO:0003824	catalytic activity	77	0.07	LOC608697, LOC607002, PIK3CD, PTPN21, STKLD1, DBH, ENO1, CAPN7, ADAMTSL2, WDR5, CA6, PMPCA, KIF1B, NUDT16, QSOX2, PTGDS, LOC480667, ACAD11, DIS3L2, PIAS4, MAP2K2, ENTPD2, DAPK3, MATK, HSD11B1L, TRAF2, SIRT6, UBE4B, CEL, TFB2M, GBGT1, DUSP22, LOC485024, DPEP1, ACPP, PHPT1, DUS3L, H6PD, CAB39, UHRF1, UBA5, PIK3R4, GLT6D1, SPG7, ATP2C1, AGPAT2, ABO, AK8, FUT5, FUT7, DPP7, NDOR1, ADAMTS13, GMDS, DPP9, NACC2, INPP5E, PDE6D, PIP5K1C, LOC607011, ABCA2, SURF1, KDM4B, LOC476732, DOHH, EEF2, TUBB4B, GALC, SPSB1, SCCPDH, NMNAT1, KIF26B, SARDH, CDK10, TRIP12, PTPN3, NEK11	1.15
GO:0008237	metallopeptidase activity	6	0.08	ADAMTS13, ADAMTSL2, DPEP1, PMPCA, NUDT16, SPG7	2.60
GO:0090482	vitamin transmembrane transporter activity	2	0.08	LOC486150, SLC19A3	22.78

GO:0045118	azole transporter activity	2	0.08	LOC486150, SLC19A3	22.78
GO:1901474	azole transmembrane transporter activity	2	0.08	LOC486150, SLC19A3	22.78
GO:0001076	transcription factor activity, RNA polymerase II transcription factor binding	5	0.09	RERE, NOTCH1, LHX3, NACC2, SNAPC4	2.97
GO:0050661	NADP binding	3	0.09	NDOR1, H6PD, GMDS	5.86
GO:0000989	transcription factor activity, transcription factor binding	10	0.10	RERE, PIAS4, NOTCH1, LHX3, NACC2, SNAPC4, SIRT6, EDF1, TFB2M, MED27	1.82
GO:0048037	cofactor binding	7	0.10	NDOR1, H6PD, GMDS, LOC608697, SIRT6, ACAD11, DUS3L	2.19
Cellular component (GO Term)		Count	PValue	Genes	Fold Enrichment
GO:0016363	nuclear matrix	5	0.01	PIAS4, GFI1B, AHCTF1, UHRF1, CHMP1A	6.47
GO:0005930	axoneme	5	0.02	INPP5E, PIK3R4, TMEM141, AK8, SPATA7	5.09
GO:0097014	ciliary plasm	5	0.02	INPP5E, PIK3R4, TMEM141, AK8, SPATA7	5.09
GO:0034399	nuclear periphery	5	0.02	PIAS4, GFI1B, AHCTF1, UHRF1, CHMP1A	4.64
GO:0005811	lipid particle	4	0.03	SCCPDH, PLIN4, PLIN3, PLIN5	5.65
GO:0044441	ciliary part	8	0.04	TTC8, CATSPERD, SSNA1, INPP5E, PIK3R4, TMEM141, AK8, SPATA7	2.58
GO:0031090	organelle membrane	28	0.05	AHCTF1, NDUFA11, DBH, SPG7, ATP2C1, FUT5, FZR1, TMEM201, FUT7, ATCAY, INPP5E, CHMP1A, PDE6D, PMPCA, KIF1B, QSOX2, C20H19ORF70, PTGDS, ACAD11, SURF1, SEC16A, DNAJC13, TRAF2, LOC100684996, MFSD12, GBGT1, ACPP, SLC25A33	1.44
GO:0005720	nuclear heterochromatin	3	0.06	KDM4B, UHRF1, SIRT6	7.77
GO:0005929	cilium	10	0.07	TTC8, CATSPERD, ACTL7A, SSNA1, INPP5E, PIK3R4, TMEM141, AK8, SPATA7, GAS8	1.98
GO:0005578	proteinaceous extracellular matrix	8	0.07	ADAMTS13, ENTPD2, COL5A1, COL4A3, ADAMTSL2, COL6A6, LOC610614, COL6A5	2.18
GO:0044445	cytosolic part	7	0.08	RPL7A, SURF6, PSMD1, RPL13, TSC1, ENO1, TICAM1	2.35
GO:0043226	organelle	159	0.08	NDUFA11, CPNE4, CPNE7, LOC489640, HNRNPU, ENO1, TTF1, TMEM141, CNST, ATCAY, CAPN7, PSMD1, PMPCA, NUDT16,	1.07

				PTGDS, C20H19ORF70, LOC480667, ACAD11, PIAS4, ENTPD2, SP110, DAPK3, RFX2, UBE4B, HDGFRP2, TSC1, MFSD12, GBGT1, EML5, NCL, COL4A3, DPEP1, PHPT1, ZNF276, NOTCH1, ACTL7A, ANKRD11, UBA5, PIK3R4, FAM206A, COPS7B, SPATA7, AK8, CAMSAP1, FUT5, FUT7, DPP7, FLRT2, SOHLH1, NACC2, INPP5E, SAFB2, SURF6, ZC3H14, RALGDS, PPP1R26, ABCA2, SURF1, SPHKAP, FOXF2, SURF2, SEC16A, SURF4, EAF1, FANCA, DNAJC13, EEF2, LOC100684996, UBAC1, RAX2, SETX, SCCPDH, LHX3, COL5A1, ITM2C, BARHL1, RERE, AHCTF1, CLIC3, TCF25, LOC607002, PTPN21, LOC100686622, DBH, IKBKAP, MYDGF, MRPL3, FAM166A, EPB41L4B, RPL7A, C8G, CHAF1A, OBP2B, DNER, CHMP1A, SNAPC4, WDR5, CA6, HMG20B, KIF1B, NELFB, QSOX2, UBXN6, DIS3L2, EDF1, TRAF2, SIRT6, TFB2M, SAFB, MED27, RAD23B, SPIDR, REXO4, TMEM210, CATSPERD, DNLZ, PLIN4, PLIN3, ACPP, CACTIN, GAS8, EXOC2, PLIN5, SDCCAG3, GTF3C4, CAB39, UHRF1, SSNA1, SPG7, ATP2C1, AGPAT2, MRPL54, TMEM201, FZR1, RXRA, TMEM203, GMDS, PDE6D, RPL13, ZNF462, LRRC26, KDM4B, RANBP3, TUBB4B, LOC479600, GRIN1, GALC, GFI1B, TTC8, LRG1, NMNAT1, KIF26B, NFIC, SARDH, TRIP12, SLC25A33, TJP3, NEK11, PTPN3	
GO:0005576	extracellular region	58	0.09	AHCTF1, CLIC3, CPNE4, CPNE7, LOC489640, DBH, ENO1, MYDGF, RPL7A, NPPC, LCNL1, CANF2, C8G, CAPN7, OBP2B, ADAMTSL2, CHMP1A, PSMD1, CA6, PMPCA, NRTN, QSOX2, PTGDS, UBXN6, ENTPD2, LOC102153243, EDF1, EML5, NCL, COL4A3, PLIN4, DPEP1, COL6A6, COL6A5, ACPP, CACTIN, PHPT1, LOC491264, CAB39, DPP7, ADAMTS13, FLRT2, GMDS, LCN15, SAFB2, LRRC4C, LRRC26, SPHKAP, CCL20, EAF1, DNAJC13, EEF2, TUBB4B, UBAC1, LRG1, COL5A1, LOC610614, ITM2C	1.19

Gene Ontology (GO) terms and KEGG pathways analysis using genes identified in BullMastiff vs Bulldog					
KEGG(Term)		Count	PValue	Genes	Fold Enrichment
cfa00982	Drug metabolism - cytochrome P450	5	0.00	FMO1, FMO2, FMO3, FMO4, LOC490346	9.70
cfa01100	Metabolic pathways	22	0.01	NDUFA11, RPE, LOC476732, DBH, CEL, LIAS, PRDX6, AGPAT2, ABO, AK8, GBGT1, GK2, PIGC, UGDH, FUT5, ACADL, CPS1, INPP5E, SARDH, PGM2, PIP5K1C, EXTL3	1.69

cfa03320	PPAR signaling pathway	4	0.04	RXRA, ACADL, LOC476732, GK2	5.07
cfa04068	FoxO signaling pathway	5	0.05	MAP2K2, FASLG, RAG2, S1PR4, RAG1	3.48
cfa05200	Pathways in cancer	9	0.06	FGF5, RALA, FZD3, RXRA, MAP2K2, DAPK3, FASLG, RALGDS, GLI3	2.10
cfa05231	Choline metabolism in cancer	4	0.09	MAP2K2, TSC1, PIP5K1C, RALGDS	3.69
Biological Process (GO Term)		Count	PValue	Genes	Fold Enrichment
GO:0021915	neural tube development	7	0.01	RALA, FZD3, NOTCH1, WDR19, TSC1, LIAS, GLI3	4.34
GO:0008152	metabolic process	106	0.01	KLB, PRDM6, LDLRAD3, TTF1, RPL9, SLC9C2, FGF5, ATCAY, PMPCA, RAG2, RAG1, PIAS4, CSNK1G3, TLE2, MAP2K2, SCARA5, DAPK3, RFX2, MATK, HDGFRP2, TSC1, CEL, TICAM1, GBGT1, ZNF395, NOTCH1, PDS5A, LIAS, FHIT, ABO, AK8, FUT5, HMBOX1, ADAMTS13, SOHLH1, LOC100686484, NACC2, INPP5E, SAFB2, LRRC4C, PPP1R26, SURF1, SUCO, INHBA, EEF2, KLHL20, RAX2, SETX, MSRA, CPS1, LHX3, COL5A1, TNFSF4, INTS9, CDK13, UBE2K, BARHL1, FASLG, DBH, GLI3, MYDGF, RPL7A, YAE1D1, ACADL, SNAPC4, WDR5, PGM2, HMG20B, MYOC, RFC1, SIRT6, SAFB, PIGC, DNM3, TLR1, UGDH, PLIN4, CACTIN, PLIN5, UHRF1, RPE, PRR5L, GLT6D1, AGPAT2, GK2, FZR1, RXRA, ERBB4, KANSL1L, EXTL3, KDM4B, CARD9, NAA11, LOC476732, FMO1, FMO2, ELP3, FMO3, DOHH, TPD52L1, FMO4, LOC490346, GFI1B, KLHL5, NFIC, APBA3	1.18
GO:0002237	response to molecule of bacterial origin	8	0.01	TLR1, CPS1, TBXA2R, TNFSF4, CARD9, LIAS, TICAM1, CACTIN	3.64
GO:0017144	drug metabolic process	3	0.01	FMO1, FMO2, FMO4	20.00
GO:0051240	positive regulation of multicellular organismal process	22	0.01	FZD3, NOTCH1, MAP2K2, CARD9, SUCO, DBH, INHBA, TICAM1, AGPAT2, GLI3, TRDN, TLR1, SETX, MYDGF, LRG1, CPS1, TBXA2R, ERBB4, TNFSF4, HMG20B, RAG2, RAG1	1.79
GO:0022008	neurogenesis	22	0.01	SEMA6B, BARHL1, FZD3, NOTCH1, MAP2K2, MYOC, PRDM6, CEP120, ELP3, INHBA, GLI3, CAMSAP1, FGF5, SETX, ATCAY, LHX3, ERBB4, MAP2, KIF13B, HMG20B, NRTN, LRRC4C	1.76
GO:0007399	nervous system development	28	0.01	BARHL1, RALA, NOTCH1, PRDM6, CEP120, LIAS, GLI3, AK8, CAMSAP1, FGF5, ATCAY, ERBB4, MAP2, KIF13B,	1.61

				HMG20B, NRTN, LRRC4C, SEMA6B, FZD3, MAP2K2, MYOC, WDR19, TSC1, ELP3, CEL, INHBA, SETX, LHX3	
GO:0071704	organic substance metabolic process	101	0.01	KLB, PRDM6, LDLRAD3, TTF1, RPL9, SLC9C2, FGF5, ATCAY, PMPCA, RAG2, RAG1, PIAS4, CSNK1G3, TLE2, MAP2K2, SCARA5, DAPK3, RFX2, MATK, HDGFRP2, TSC1, CEL, TICAM1, GBGT1, ZNF395, NOTCH1, PDS5A, LIAS, FHIT, ABO, AK8, FUT5, HMBOX1, ADAMTS13, SOHLH1, LOC100686484, NACC2, INPP5E, SAFB2, LRRC4C, SURF1, SUCO, INHBA, EEF2, KLHL20, RAX2, SETX, MSRA, CPS1, LHX3, COL5A1, TNFSF4, INTS9, CDK13, UBE2K, BARHL1, FASLG, DBH, GLI3, MYDGF, RPL7A, YAE1D1, ACADL, SNAPC4, WDR5, PGM2, HMG20B, MYOC, RFC1, SIRT6, SAFB, PIGC, DNM3, TLR1, UGDH, PLIN4, CACTIN, PLIN5, UHRF1, RPE, PRR5L, GLT6D1, AGPAT2, GK2, FZR1, RXRA, ERBB4, KANSL1L, EXTL3, KDM4B, CARD9, NAA11, FMO1, FMO2, ELP3, DOHH, TPD52L1, GFI1B, KLHL5, NFIC, APBA3	1.17
GO:0009059	macromolecule biosynthetic process	52	0.01	BARHL1, PRDM6, FASLG, TTF1, RPL9, GLI3, MYDGF, RPL7A, YAE1D1, SNAPC4, WDR5, PIAS4, TLE2, RFC1, DAPK3, RFX2, HDGFRP2, SIRT6, TSC1, TICAM1, SAFB, GBGT1, TLR1, PIGC, UGDH, CACTIN, ZNF395, NOTCH1, UHRF1, PDS5A, FUT5, HMBOX1, RXRA, LOC100686484, SOHLH1, ERBB4, NACC2, SAFB2, EXTL3, CARD9, SUCO, ELP3, INHBA, EEF2, RAX2, SETX, GFI1B, COL5A1, LHX3, NFIC, TNFSF4, CDK13	1.35
GO:1901576	organic substance biosynthetic process	60	0.01	BARHL1, PRDM6, FASLG, DBH, TTF1, RPL9, GLI3, MYDGF, RPL7A, YAE1D1, SNAPC4, WDR5, PIAS4, TLE2, RFC1, DAPK3, RFX2, HDGFRP2, SIRT6, TSC1, TICAM1, SAFB, GBGT1, TLR1, PIGC, UGDH, CACTIN, PLIN5, ZNF395, NOTCH1, UHRF1, LIAS, PDS5A, AGPAT2, AK8, GK2, FUT5, HMBOX1, RXRA, LOC100686484, SOHLH1, ERBB4, NACC2, SAFB2, EXTL3, SURF1, CARD9, SUCO, ELP3, INHBA, EEF2, RAX2, SETX, GFI1B, COL5A1, CPS1, LHX3, NFIC, TNFSF4, CDK13	1.30
GO:0009058	biosynthetic process	61	0.02	BARHL1, PRDM6, FASLG, DBH, TTF1, RPL9, GLI3, MYDGF, RPL7A, YAE1D1, SNAPC4, WDR5, PIAS4, TLE2, RFC1, DAPK3, RFX2, HDGFRP2, SIRT6, TSC1, TICAM1, SAFB, GBGT1, TLR1, PIGC, UGDH, CACTIN,	1.29

				PLIN5, ZNF395, NOTCH1, UHRF1, LIAS, PDS5A, AGPAT2, AK8, GK2, FUT5, HMBOX1, RXRA, LOC100686484, SOHLH1, ERBB4, NACC2, SAFB2, EXTL3, SURF1, CARD9, SUCO, ELP3, INHBA, DOHH, EEF2, RAX2, SETX, GFI1B, COL5A1, CPS1, LHX3, NFIC, TNFSF4, CDK13	
GO:0031175	neuron projection development	14	0.02	SEMA6B, FZD3, NOTCH1, MAP2K2, MYOC, GLI3, CAMSAP1, SETX, ATCAY, LHX3, MAP2, KIF13B, NRTN, LRRC4C	2.09
GO:0045089	positive regulation of innate immune response	6	0.02	TLR1, CARD9, TLR10, TICAM1, CACTIN, UBE2K	3.93
GO:0032755	positive regulation of interleukin-6 production	4	0.02	TLR1, TNFSF4, CARD9, TICAM1	6.96
GO:0014068	positive regulation of phosphatidylinositol 3-kinase signaling	4	0.02	MYDGF, MYOC, ERBB4, PRR5L	6.81
GO:0070995	NADPH oxidation	2	0.02	FMO1, FMO2	80.01
GO:0022603	regulation of anatomical structure morphogenesis	16	0.02	SEMA6B, CSNK1G3, FZD3, NOTCH1, MAP2K2, MYOC, CEP120, DAPK3, FASLG, CAMSAP1, MYDGF, RXRA, LRG1, COL5A1, KIF13B, LRRC4C	1.85
GO:0030030	cell projection organization	19	0.03	SEMA6B, RALA, FZD3, NOTCH1, MAP2K2, MYOC, CEP120, WDR19, RFX2, TSC1, GLI3, CAMSAP1, SETX, ATCAY, LHX3, MAP2, KIF13B, NRTN, LRRC4C	1.72
GO:0031349	positive regulation of defense response	7	0.03	TLR1, TNFSF4, CARD9, TLR10, TICAM1, CACTIN, UBE2K	3.08
GO:0030154	cell differentiation	42	0.03	BARHL1, NOTCH1, PRDM6, CEP120, FASLG, TTF1, GLI3, CAMSAP1, FGF5, FZR1, RXRA, ATCAY, SOHLH1, ERBB4, MAP2, SNAPC4, SAFB2, TMEM8C, KIF13B, HMG20B, NRTN, RAG2, LRRC4C, RAG1, SEMA6B, FZD3, MAP2K2, MYOC, MATK, RFX2, SUCO, PRRC2C, TSC1, ELP3, INHBA, EEF2, SETX, LRG1, LHX3, COL5A1, CATSPERD, TNFSF4	1.36
GO:0008284	positive regulation of cell proliferation	13	0.03	KLB, FZD3, EGFL7, NOTCH1, SIRT6, TICAM1, GLI3, FGF5, MYDGF, FZR1, LRG1, ERBB4, TNFSF4	2.01
GO:0006739	NADP metabolic process	3	0.03	RPE, FMO1, FMO2	10.91
GO:0044707	single-multicellular organism process	61	0.03	BARHL1, PRDM6, CEP120, FASLG, DBH, GLI3, PTPRG, FGF5, MYDGF, ATCAY, OBP2B, ADAMTSL2, KIF13B, WDR5, HMG20B, NRTN, RAG2, RAG1, SEMA6B, MAP2K2, SCARA5, MYOC, TSC1, CEL, TICAM1, TRDN,	1.25

				TLR1, UGDH, TLR10, CACTIN, RALA, NOTCH1, LIAS, AGPAT2, AK8, CAMSAP1, FZR1, RXRA, ERBB4, MAP2, TBXA2R, SAFB2, LRRC4C, FZD3, CARD9, WDR19, SUCO, PRRC2C, ELP3, INHBA, EEF2, SETX, GFI1B, LRG1, COL5A1, CPS1, LHX3, NFIC, TNFSF4, APBA3, CDK13	
GO:0060255	regulation of macromolecule metabolic process	56	0.03	KLB, BARHL1, PRDM6, LDLRAD3, FASLG, GLI3, FGF5, MYDGF, SNAPC4, WDR5, HMG20B, RAG2, RAG1, PIAS4, TLE2, MAP2K2, SCARA5, MYOC, DAPK3, RFX2, SIRT6, TSC1, TICAM1, SAFB, TLR1, CACTIN, ZNF395, NOTCH1, UHRF1, PRR5L, PDS5A, FHIT, FZR1, HMBOX1, RXRA, LOC100686484, ERBB4, NACC2, SAFB2, LRRC4C, KDM4B, CARD9, SUCO, ELP3, INHBA, TPD52L1, EEF2, RAX2, SETX, GFI1B, LHX3, NFIC, TNFSF4, APBA3, CDK13, UBE2K	1.26
GO:0019222	regulation of metabolic process	59	0.03	KLB, BARHL1, PRDM6, LDLRAD3, FASLG, GLI3, FGF5, MYDGF, ATCAY, SNAPC4, WDR5, HMG20B, RAG2, RAG1, PIAS4, TLE2, MAP2K2, SCARA5, MYOC, DAPK3, RFX2, SIRT6, TSC1, TICAM1, SAFB, TLR1, CACTIN, PLIN5, ZNF395, NOTCH1, UHRF1, PRR5L, PDS5A, FHIT, FZR1, HMBOX1, RXRA, LOC100686484, ERBB4, NACC2, SAFB2, LRRC4C, PPP1R26, KDM4B, CARD9, SUCO, ELP3, INHBA, TPD52L1, EEF2, RAX2, SETX, GFI1B, LHX3, NFIC, TNFSF4, APBA3, CDK13, UBE2K	1.25
GO:0048869	cellular developmental process	45	0.03	BARHL1, NOTCH1, PRDM6, CEP120, FASLG, TTF1, GLI3, CAMSAP1, FGF5, FZR1, RXRA, ATCAY, SOHLH1, ERBB4, MAP2, SNAPC4, SAFB2, TMEM8C, KIF13B, HMG20B, NRTN, RAG2, LRRC4C, RAG1, SEMA6B, CSNK1G3, FZD3, MAP2K2, MYOC, DAPK3, WDR19, MATK, RFX2, SUCO, PRRC2C, TSC1, ELP3, INHBA, EEF2, SETX, LRG1, LHX3, COL5A1, CATSPERD, TNFSF4	1.31
GO:0005975	carbohydrate metabolic process	13	0.03	KLB, RPE, SIRT6, GLT6D1, ABO, GBGT1, GK2, UGDH, FUT5, CPS1, INPP5E, WDR5, PGM2	1.94
GO:0014066	regulation of phosphatidylinositol 3-kinase signaling	4	0.04	MYDGF, MYOC, ERBB4, PRR5L	5.52
GO:0044249	cellular biosynthetic process	57	0.04	BARHL1, PRDM6, FASLG, DBH, TTF1, RPL9, GLI3, MYDGF, RPL7A, YAE1D1, SNAPC4, WDR5, PIAS4, TLE2, RFC1, DAPK3, RFX2, HDGFRP2, SIRT6, TSC1,	1.25

				TICAM1, SAFB, GBGT1, TLR1, PIGC, UGDH, CACTIN, PLIN5, ZNF395, NOTCH1, UHRF1, LIAS, PDS5A, AGPAT2, AK8, FUT5, HMBOX1, RXRA, LOC100686484, SOHLH1, ERBB4, NACC2, SAFB2, EXTL3, SURF1, CARD9, ELP3, INHBA, EEF2, RAX2, SETX, GFI1B, CPS1, LHX3, NFIC, TNFSF4, CDK13	
GO:0045088	regulation of innate immune response	6	0.04	TLR1, CARD9, TLR10, TICAM1, CACTIN, UBE2K	3.27
GO:0060528	secretory columnal luminar epithelial cell differentiation involved in prostate glandular acinus development	2	0.04	NOTCH1, RXRA	53.34
GO:0002331	pre-B cell allelic exclusion	2	0.04	RAG2, RAG1	53.34
GO:0048699	generation of neurons	19	0.04	SEMA6B, BARHL1, FZD3, NOTCH1, MAP2K2, MYOC, ELP3, INHBA, GLI3, CAMSAP1, SETX, ATCAY, LHX3, ERBB4, MAP2, KIF13B, HMG20B, NRTN, LRRC4C	1.65
GO:2000648	positive regulation of stem cell proliferation	3	0.04	FZD3, SIRT6, GLI3	9.60
GO:0060038	cardiac muscle cell proliferation	3	0.04	NOTCH1, RXRA, ERBB4	9.60
GO:0006915	apoptotic process	20	0.04	BARHL1, PIAS4, FZD3, NOTCH1, DAPK3, FASLG, DBH, INHBA, TICAM1, TPD52L1, FHIT, GLI3, KLHL20, SETX, MYDGF, ATCAY, LHX3, ERBB4, NACC2, RAG1	1.61
GO:0010556	regulation of macromolecule biosynthetic process	39	0.04	BARHL1, ZNF395, NOTCH1, UHRF1, PRDM6, FASLG, PDS5A, GLI3, MYDGF, HMBOX1, RXRA, LOC100686484, ERBB4, NACC2, SNAPC4, SAFB2, WDR5, PIAS4, TLE2, CARD9, DAPK3, RFX2, SUCO, SIRT6, TSC1, ELP3, INHBA, TICAM1, EEF2, SAFB, RAX2, TLR1, SETX, GFI1B, LHX3, NFIC, TNFSF4, CDK13, CACTIN	1.34
GO:0051239	regulation of multicellular organismal process	32	0.04	NOTCH1, FASLG, DBH, AGPAT2, GLI3, PTPRG, MYDGF, RXRA, TBXA2R, ERBB4, KIF13B, HMG20B, RAG2, LRRC4C, RAG1, SEMA6B, FZD3, MAP2K2, SCARA5, CARD9, SUCO, INHBA, TICAM1, TRDN, TLR1, SETX, LRG1, COL5A1, CPS1, TNFSF4, TLR10, CACTIN	1.40
GO:0006796	phosphate-containing compound metabolic process	32	0.04	KLB, RPE, PRR5L, FHIT, AGPAT2, AK8, GK2, MYDGF, ERBB4, INPP5E, LRRC4C, PPP1R26, CSNK1G3, SURF1, MAP2K2, MYOC, CARD9, DAPK3, MATK, FMO1, FMO2, SIRT6, TSC1, INHBA, TPD52L1, TLR1, PIGC, SETX, CPS1, CDK13, CACTIN, UBE2K	1.40

GO:0006793	phosphorus metabolic process	32	0.04	KLB, RPE, PRR5L, FHIT, AGPAT2, AK8, GK2, MYDGF, ERBB4, INPP5E, LRRC4C, PPP1R26, CSNK1G3, SURF1, MAP2K2, MYOC, CARD9, DAPK3, MATK, FMO1, FMO2, SIRT6, TSC1, INHBA, TPD52L1, TLR1, PIGC, SETX, CPS1, CDK13, CACTIN, UBE2K	1.40
GO:0012501	programmed cell death	21	0.04	BARHL1, PIAS4, FZD3, NOTCH1, CARD9, DAPK3, FASLG, DBH, INHBA, TICAM1, TPD52L1, FHIT, GLI3, KLHL20, SETX, MYDGF, ATCAY, LHX3, ERBB4, NACC2, RAG1	1.57
GO:0002224	toll-like receptor signaling pathway	4	0.04	TLR1, TLR10, TICAM1, CACTIN	5.08
GO:0046622	positive regulation of organ growth	3	0.04	NOTCH1, ERBB4, RAG2	8.89
GO:0006464	cellular protein modification process	38	0.04	KLB, UHRF1, PRR5L, LIAS, FUT5, MYDGF, FZR1, ERBB4, WDR5, HMG20B, LRRC4C, KANSL1L, RAG1, PIAS4, CSNK1G3, KDM4B, MAP2K2, MYOC, CARD9, DAPK3, NAA11, MATK, SIRT6, TSC1, DOHH, INHBA, TICAM1, TPD52L1, GBGT1, KLHL20, TLR1, PIGC, SETX, GFI1B, KLHL5, CDK13, CACTIN, UBE2K	1.34
GO:0036211	protein modification process	38	0.04	KLB, UHRF1, PRR5L, LIAS, FUT5, MYDGF, FZR1, ERBB4, WDR5, HMG20B, LRRC4C, KANSL1L, RAG1, PIAS4, CSNK1G3, KDM4B, MAP2K2, MYOC, CARD9, DAPK3, NAA11, MATK, SIRT6, TSC1, DOHH, INHBA, TICAM1, TPD52L1, GBGT1, KLHL20, TLR1, PIGC, SETX, GFI1B, KLHL5, CDK13, CACTIN, UBE2K	1.34
GO:0043170	macromolecule metabolic process	83	0.05	KLB, BARHL1, PRDM6, LDLRAD3, FASLG, TTF1, RPL9, GLI3, FGF5, MYDGF, RPL7A, YAE1D1, SNAPC4, WDR5, HMG20B, PMPCA, RAG2, RAG1, PIAS4, CSNK1G3, TLE2, MAP2K2, RFC1, SCARA5, MYOC, DAPK3, MATK, RFX2, HDGFRP2, SIRT6, TSC1, TICAM1, SAFB, GBGT1, TLR1, PIGC, DNM3, UGDH, PLIN4, CACTIN, ZNF395, NOTCH1, UHRF1, PRR5L, LIAS, PDS5A, FHIT, FUT5, FZR1, HMBOX1, RXRA, LOC100686484, SOHLH1, ERBB4, NACC2, SAFB2, KANSL1L, LRRC4C, EXTL3, KDM4B, CARD9, NAA11, SUCO, ELP3, INHBA, DOHH, TPD52L1, EEF2, RAX2, KLHL20, SETX, MSRA, GFI1B, KLHL5, COL5A1, CPS1, LHX3, NFIC, TNFSF4, APBA3, INTS9, CDK13, UBE2K	1.16
GO:0010468	regulation of gene expression	41	0.05	BARHL1, ZNF395, NOTCH1, UHRF1, PRDM6, LDLRAD3, FASLG, GLI3, FGF5, MYDGF, HMBOX1, RXRA,	1.31

				LOC100686484, ERBB4, NACC2, SNAPC4, SAFB2, WDR5, RAG2, RAG1, PIAS4, TLE2, SCARA5, DAPK3, RFX2, SIRT6, TSC1, ELP3, INHBA, TICAM1, EEF2, SAFB, RAX2, SETX, GFI1B, LHX3, NFIC, TNFSF4, CDK13, CACTIN, APBA3	
GO:0043379	memory T cell differentiation	2	0.05	TNFSF4, TSC1	40.00
GO:0042981	regulation of apoptotic process	18	0.05	BARHL1, PIAS4, FZD3, NOTCH1, CARD9, DAPK3, FASLG, DBH, INHBA, TPD52L1, GLI3, KLHL20, SETX, MYDGF, LHX3, ERBB4, NACC2, RAG1	1.62
GO:0022604	regulation of cell morphogenesis	10	0.05	SEMA6B, CSNK1G3, NOTCH1, MAP2K2, MYOC, CEP120, DAPK3, KIF13B, LRRC4C, CAMSAP1	2.08
GO:0033993	response to lipid	10	0.05	SETX, RXRA, CPS1, TBXA2R, TNFSF4, SAFB2, SAFB, LIAS, TICAM1, CACTIN	2.07
GO:1902533	positive regulation of intracellular signal transduction	14	0.05	KLB, PIAS4, NOTCH1, MYOC, CARD9, PRR5L, FASLG, TICAM1, TPD52L1, TRDN, TLR1, MYDGF, ERBB4, NACC2	1.77
GO:0044262	cellular carbohydrate metabolic process	6	0.05	CPS1, RPE, INPP5E, WDR5, SIRT6, GK2	2.95
GO:0032496	response to lipopolysaccharide	6	0.05	CPS1, TBXA2R, TNFSF4, LIAS, TICAM1, CACTIN	2.95
GO:0009889	regulation of biosynthetic process	40	0.05	BARHL1, ZNF395, NOTCH1, UHRF1, PRDM6, FASLG, PDS5A, GLI3, MYDGF, HMBOX1, RXRA, LOC100686484, ERBB4, NACC2, SNAPC4, SAFB2, WDR5, PIAS4, TLE2, CARD9, DAPK3, RFX2, SUCO, SIRT6, TSC1, ELP3, INHBA, TICAM1, EEF2, SAFB, RAX2, TLR1, SETX, GFI1B, LHX3, NFIC, TNFSF4, CDK13, CACTIN, PLIN5	1.31
GO:0043067	regulation of programmed cell death	18	0.05	BARHL1, PIAS4, FZD3, NOTCH1, CARD9, DAPK3, FASLG, DBH, INHBA, TPD52L1, GLI3, KLHL20, SETX, MYDGF, LHX3, ERBB4, NACC2, RAG1	1.60
GO:0030182	neuron differentiation	17	0.05	SEMA6B, FZD3, NOTCH1, MAP2K2, MYOC, INHBA, GLI3, CAMSAP1, SETX, ATCAY, LHX3, ERBB4, MAP2, KIF13B, HMG20B, NRTN, LRRC4C	1.63
GO:0014065	phosphatidylinositol 3-kinase signaling	4	0.06	MYDGF, MYOC, ERBB4, PRR5L	4.57
GO:0055017	cardiac muscle tissue growth	3	0.06	NOTCH1, RXRA, ERBB4	7.74
GO:0043412	macromolecule modification	39	0.06	KLB, UHRF1, PRR5L, LIAS, FUT5, MYDGF, FZR1, ERBB4, WDR5, HMG20B, LRRC4C, KANSL1L, RAG1, PIAS4, CSNK1G3, KDM4B, MAP2K2, MYOC, CARD9, DAPK3, NAA11, MATK, SIRT6, TSC1, ELP3, DOHH,	1.31

				INHBA, TICAM1, TPD52L1, GBGT1, KLHL20, TLR1, PIGC, SETX, GFI1B, KLHL5, CDK13, CACTIN, UBE2K	
GO:0032640	tumor necrosis factor production	4	0.06	TLR1, CARD9, TICAM1, CACTIN	4.44
GO:0006351	transcription, DNA-templated	31	0.06	BARHL1, ZNF395, NOTCH1, UHRF1, PRDM6, FASLG, TTF1, GLI3, MYDGF, HMBOX1, RXRA, SOHLH1, ERBB4, NACC2, SNAPC4, SAFB2, WDR5, PIAS4, RFX2, HDGFRP2, SIRT6, ELP3, INHBA, TICAM1, SAFB, SETX, LHX3, NFIC, TNFSF4, CDK13, CACTIN	1.36
GO:0002329	pre-B cell differentiation	2	0.06	RAG2, RAG1	32.00
GO:0050793	regulation of developmental process	26	0.06	NOTCH1, PRDM6, CEP120, FASLG, GLI3, CAMSAP1, MYDGF, FZR1, RXRA, ERBB4, KIF13B, HMG20B, RAG2, LRRC4C, RAG1, SEMA6B, CSNK1G3, FZD3, MAP2K2, MYOC, DAPK3, INHBA, SETX, LRG1, COL5A1, TNFSF4	1.42
GO:0035148	tube formation	5	0.06	RALA, FZD3, NOTCH1, TSC1, LIAS	3.36
GO:0048666	neuron development	14	0.06	SEMA6B, FZD3, NOTCH1, MAP2K2, MYOC, GLI3, CAMSAP1, SETX, ATCAY, LHX3, MAP2, KIF13B, NRTN, LRRC4C	1.72
GO:0032680	regulation of tumor necrosis factor production	4	0.06	TLR1, CARD9, TICAM1, CACTIN	4.38
GO:0001843	neural tube closure	4	0.06	RALA, FZD3, TSC1, LIAS	4.38
GO:0044267	cellular protein metabolic process	47	0.06	KLB, UHRF1, PRR5L, FASLG, LIAS, RPL9, FHIT, FUT5, MYDGF, RPL7A, YAE1D1, FZR1, RXRA, ERBB4, WDR5, HMG20B, PMPCA, LRRC4C, KANSL1L, RAG1, PIAS4, CSNK1G3, KDM4B, MAP2K2, MYOC, CARD9, DAPK3, NAA11, MATK, SIRT6, TSC1, DOHH, INHBA, TICAM1, EEF2, TPD52L1, GBGT1, KLHL20, TLR1, PIGC, SETX, MSRA, GFI1B, KLHL5, CDK13, CACTIN, UBE2K	1.25
GO:0044271	cellular nitrogen compound biosynthetic process	46	0.06	BARHL1, ZNF395, NOTCH1, UHRF1, PRDM6, FASLG, DBH, TTF1, RPL9, GLI3, AK8, MYDGF, RPL7A, YAE1D1, HMBOX1, RXRA, LOC100686484, SOHLH1, ERBB4, NACC2, SNAPC4, SAFB2, WDR5, PIAS4, TLE2, SURF1, DAPK3, RFX2, HDGFRP2, SIRT6, TSC1, ELP3, INHBA, TICAM1, EEF2, SAFB, RAX2, SETX, UGDH, GFI1B, LHX3, CPS1, NFIC, TNFSF4, CDK13, CACTIN	1.26
GO:0060419	heart growth	3	0.06	NOTCH1, RXRA, ERBB4	7.27
GO:0031326	regulation of cellular biosynthetic process	39	0.06	BARHL1, ZNF395, NOTCH1, UHRF1, PRDM6, FASLG, PDS5A, GLI3, MYDGF, HMBOX1, RXRA, LOC100686484, ERBB4, NACC2, SNAPC4, SAFB2, WDR5, PIAS4, TLE2,	1.29

				CARD9, DAPK3, RFX2, SIRT6, TSC1, ELP3, INHBA, TICAM1, EEF2, SAFB, RAX2, TLR1, SETX, GFI1B, LHX3, NFIC, TNFSF4, CDK13, CACTIN, PLIN5	
GO:0008283	cell proliferation	21	0.06	KLB, FZD3, EGFL7, NOTCH1, MAP2K2, CEP120, EBI3, SIRT6, TSC1, INHBA, TICAM1, GLI3, FGF5, MYDGF, FZR1, RXRA, LRG1, ERBB4, TNFSF4, NACC2, RAG2	1.50
GO:0060606	tube closure	4	0.06	RALA, FZD3, TSC1, LIAS	4.32
GO:0014855	striated muscle cell proliferation	3	0.07	NOTCH1, RXRA, ERBB4	7.06
GO:0019362	pyridine nucleotide metabolic process	4	0.07	RPE, FMO1, SIRT6, FMO2	4.27
GO:0046496	nicotinamide nucleotide metabolic process	4	0.07	RPE, FMO1, SIRT6, FMO2	4.27
GO:1903555	regulation of tumor necrosis factor superfamily cytokine production	4	0.07	TLR1, CARD9, TICAM1, CACTIN	4.27
GO:0034645	cellular macromolecule biosynthetic process	47	0.07	BARHL1, ZNF395, NOTCH1, UHRF1, PRDM6, FASLG, TTF1, PDS5A, RPL9, GLI3, FUT5, MYDGF, RPL7A, YAE1D1, HMBOX1, RXRA, LOC100686484, SOHLH1, ERBB4, NACC2, SNAPC4, SAFB2, WDR5, EXTL3, PIAS4, TLE2, RFC1, DAPK3, RFX2, HDGFRP2, SIRT6, TSC1, ELP3, INHBA, TICAM1, EEF2, SAFB, GBGT1, RAX2, PIGC, SETX, GFI1B, LHX3, NFIC, TNFSF4, CDK13, CACTIN	1.24
GO:0009617	response to bacterium	8	0.07	TLR1, CPS1, TBXA2R, TNFSF4, CARD9, LIAS, TICAM1, CACTIN	2.22
GO:0007423	sensory organ development	10	0.07	FZR1, FZD3, NOTCH1, RXRA, LHX3, COL5A1, WDR19, FASLG, INHBA, GLI3	1.96
GO:0032675	regulation of interleukin-6 production	4	0.07	TLR1, TNFSF4, CARD9, TICAM1	4.21
GO:0008219	cell death	21	0.07	BARHL1, PIAS4, FZD3, NOTCH1, CARD9, DAPK3, FASLG, DBH, INHBA, TICAM1, TPD52L1, FHIT, GLI3, KLHL20, SETX, MYDGF, ATCAY, LHX3, ERBB4, NACC2, RAG1	1.48
GO:0051893	regulation of focal adhesion assembly	3	0.07	MYOC, DAPK3, TSC1	6.86
GO:0070231	T cell apoptotic process	3	0.07	FASLG, GLI3, RAG1	6.86
GO:0090109	regulation of cell-substrate junction assembly	3	0.07	MYOC, DAPK3, TSC1	6.86
GO:0046486	glycerolipid metabolic process	6	0.07	PIGC, CPS1, INPP5E, AGPAT2, GK2, PLIN5	2.71

GO:0060322	head development	11	0.07	BARHL1, FZD3, NOTCH1, MAP2K2, LHX3, ERBB4, CEP120, TSC1, INHBA, AK8, GLI3	1.86
GO:0072524	pyridine-containing compound metabolic process	4	0.07	RPE, FMO1, SIRT6, FMO2	4.16
GO:0014020	primary neural tube formation	4	0.07	RALA, FZD3, TSC1, LIAS	4.16
GO:0006357	regulation of transcription from RNA polymerase II promoter	21	0.07	BARHL1, PIAS4, ZNF395, NOTCH1, UHRF1, RFX2, ELP3, FASLG, INHBA, SAFB, GLI3, SETX, MYDGF, RXRA, LHX3, NFIC, NACC2, SNAPC4, SAFB2, WDR5, CDK13	1.47
GO:1902231	positive regulation of intrinsic apoptotic signaling pathway in response to DNA damage	2	0.07	PIAS4, NACC2	26.67
GO:0071706	tumor necrosis factor superfamily cytokine production	4	0.07	TLR1, CARD9, TICAM1, CACTIN	4.10
GO:0032635	interleukin-6 production	4	0.08	TLR1, TNFSF4, CARD9, TICAM1	4.05
GO:0097659	nucleic acid-templated transcription	35	0.08	BARHL1, ZNF395, NOTCH1, UHRF1, PRDM6, FASLG, TTF1, GLI3, MYDGF, HMBOX1, RXRA, LOC100686484, SOHLH1, ERBB4, NACC2, SNAPC4, SAFB2, WDR5, PIAS4, TLE2, RFX2, HDGFRP2, SIRT6, ELP3, INHBA, TICAM1, SAFB, RAX2, SETX, GFI1B, LHX3, NFIC, TNFSF4, CDK13, CACTIN	1.30
GO:1903391	regulation of adherens junction organization	3	0.08	MYOC, DAPK3, TSC1	6.49
GO:0032774	RNA biosynthetic process	35	0.08	BARHL1, ZNF395, NOTCH1, UHRF1, PRDM6, FASLG, TTF1, GLI3, MYDGF, HMBOX1, RXRA, LOC100686484, SOHLH1, ERBB4, NACC2, SNAPC4, SAFB2, WDR5, PIAS4, TLE2, RFX2, HDGFRP2, SIRT6, ELP3, INHBA, TICAM1, SAFB, RAX2, SETX, GFI1B, LHX3, NFIC, TNFSF4, CDK13, CACTIN	1.30
GO:0002221	pattern recognition receptor signaling pathway	4	0.08	TLR1, TLR10, TICAM1, CACTIN	3.95
GO:0031324	negative regulation of cellular metabolic process	24	0.08	PPP1R26, PIAS4, KDM4B, UHRF1, PRDM6, DAPK3, SIRT6, TSC1, PRR5L, FASLG, PDS5A, FHIT, GLI3, HMBOX1, RXRA, ATCAY, NFIC, TNFSF4, NACC2, HMG20B, CACTIN, LRRC4C, PLIN5, RAG1	1.41
GO:0010941	regulation of cell death	18	0.08	BARHL1, PIAS4, FZD3, NOTCH1, CARD9, DAPK3, FASLG, DBH, INHBA, TPD52L1, GLI3, KLHL20, SETX, MYDGF, LHX3, ERBB4, NACC2, RAG1	1.52
GO:0032268	regulation of cellular protein metabolic process	26	0.08	KLB, PRR5L, FASLG, FHIT, MYDGF, FZR1, RXRA, ERBB4, HMG20B, LRRC4C, RAG1, PIAS4, KDM4B,	1.38

				MAP2K2, MYOC, CARD9, DAPK3, TSC1, INHBA, TICAM1, EEF2, TPD52L1, TLR1, GFI1B, CACTIN, UBE2K	
GO:0032501	multicellular organismal process	66	0.08	BARHL1, PRDM6, CEP120, FASLG, DBH, GLI3, PTPRG, FGF5, MYDGF, ATCAY, OBP2B, ADAMTSL2, KIF13B, WDR5, HMG20B, NRTN, RAG2, RAG1, SEMA6B, MAP2K2, SCARA5, MYOC, RFX2, TSC1, CEL, TICAM1, TRDN, TLR1, UGDH, MYL1, CATSPERD, TLR10, CACTIN, RALA, NOTCH1, LIAS, AGPAT2, AK8, CAMSAP1, FZR1, RXRA, SOHLH1, ERBB4, MAP2, TBXA2R, SAFB2, LRRC4C, FZD3, CARD9, WDR19, SUCO, PRRC2C, ELP3, INHBA, EEF2, SETX, GFI1B, LRG1, COL5A1, CPS1, LHX3, NFIC, TNFSF4, PNOC, APBA3, CDK13	1.17
GO:0032760	positive regulation of tumor necrosis factor production	3	0.08	TLR1, CARD9, TICAM1	6.32
GO:1901362	organic cyclic compound biosynthetic process	40	0.08	BARHL1, ZNF395, NOTCH1, UHRF1, PRDM6, FASLG, DBH, TTF1, GLI3, AK8, MYDGF, HMBOX1, RXRA, LOC100686484, SOHLH1, ERBB4, NACC2, SNAPC4, SAFB2, WDR5, PIAS4, TLE2, SURF1, RFX2, HDGFRP2, SIRT6, ELP3, INHBA, TICAM1, SAFB, RAX2, SETX, UGDH, GFI1B, LHX3, CPS1, NFIC, TNFSF4, CDK13, CACTIN	1.26
GO:0048731	system development	45	0.08	BARHL1, RALA, NOTCH1, PRDM6, CEP120, FASLG, LIAS, GLI3, AK8, CAMSAP1, FGF5, MYDGF, FZR1, RXRA, ATCAY, ERBB4, MAP2, ADAMTSL2, SAFB2, WDR5, KIF13B, HMG20B, NRTN, RAG2, LRRC4C, RAG1, SEMA6B, FZD3, MAP2K2, MYOC, WDR19, PRRC2C, TSC1, ELP3, CEL, INHBA, EEF2, SETX, GFI1B, LRG1, LHX3, COL5A1, NFIC, TNFSF4, CDK13	1.24
GO:0048518	positive regulation of biological process	53	0.08	KLB, CEP120, FASLG, DBH, GLI3, FGF5, MYDGF, WDR5, HMG20B, RAG2, RAG1, SEMA6B, PIAS4, EGFL7, MAP2K2, MYOC, DAPK3, HDGFRP2, SIRT6, TSC1, TICAM1, SAFB, TRDN, TLR1, TLR10, CACTIN, PLIN5, RALA, NOTCH1, PRR5L, AGPAT2, FZR1, HMBOX1, RXRA, ERBB4, TBXA2R, NACC2, FZD3, RANBP3, SURF4, CARD9, SUCO, ELP3, INHBA, TPD52L1, EEF2, SETX, LRG1, CPS1, LHX3, NFIC, TNFSF4, UBE2K	1.20
GO:0031323	regulation of cellular metabolic process	53	0.08	KLB, BARHL1, PRDM6, FASLG, GLI3, MYDGF, ATCAY, SNAPC4, WDR5, HMG20B, RAG1, PIAS4, TLE2,	1.20

				MAP2K2, MYOC, DAPK3, RFX2, SIRT6, TSC1, TICAM1, SAFB, TLR1, CACTIN, PLIN5, ZNF395, NOTCH1, UHRF1, PRR5L, PDS5A, FHIT, FZR1, HMBOX1, RXRA, LOC100686484, ERBB4, NACC2, SAFB2, LRRC4C, PPP1R26, KDM4B, CARD9, ELP3, INHBA, TPD52L1, EEF2, RAX2, SETX, GFI1B, LHX3, NFIC, TNFSF4, CDK13, UBE2K	
GO:0009404	toxin metabolic process	2	0.08	FMO1, FMO2	22.86
GO:0001841	neural tube formation	4	0.08	RALA, FZD3, TSC1, LIAS	3.86
GO:0002758	innate immune response-activating signal transduction	4	0.08	TLR1, TLR10, TICAM1, CACTIN	3.86
GO:1903557	positive regulation of tumor necrosis factor superfamily cytokine production	3	0.08	TLR1, CARD9, TICAM1	6.15
GO:0055114	oxidation-reduction process	8	0.08	ACADL, CPS1, FMO1, FMO2, DBH, FMO3, PLIN5, LOC490346	2.11
GO:0018130	heterocycle biosynthetic process	39	0.09	BARHL1, ZNF395, NOTCH1, UHRF1, PRDM6, FASLG, TTF1, GLI3, AK8, MYDGF, HMBOX1, RXRA, LOC100686484, SOHLH1, ERBB4, NACC2, SNAPC4, SAFB2, WDR5, PIAS4, TLE2, SURF1, RFX2, HDGFRP2, SIRT6, ELP3, INHBA, TICAM1, SAFB, RAX2, SETX, UGDH, GFI1B, LHX3, CPS1, NFIC, TNFSF4, CDK13, CACTIN	1.26
GO:0009967	positive regulation of signal transduction	18	0.09	KLB, PIAS4, NOTCH1, MYOC, CARD9, DAPK3, PRR5L, FASLG, INHBA, TICAM1, TPD52L1, TRDN, TLR1, MYDGF, LRG1, ERBB4, NACC2, UBE2K	1.50
GO:0060429	epithelium development	15	0.09	RALA, FZD3, NOTCH1, MAP2K2, WDR19, TSC1, INHBA, LIAS, GLI3, FZR1, RXRA, COL5A1, ERBB4, ADAMTSL2, SAFB2	1.58
GO:0051094	positive regulation of developmental process	15	0.09	FZD3, NOTCH1, MAP2K2, MYOC, CEP120, INHBA, GLI3, SETX, MYDGF, LRG1, ERBB4, TNFSF4, HMG20B, RAG2, RAG1	1.58
GO:0044237	cellular metabolic process	92	0.09	KLB, BARHL1, PRDM6, FASLG, DBH, TTF1, RPL9, GLI3, MYDGF, RPL7A, YAE1D1, ATCAY, ACADL, SNAPC4, WDR5, HMG20B, PMPCA, RAG2, RAG1, PIAS4, CSNK1G3, TLE2, MAP2K2, RFC1, MYOC, DAPK3, MATK, RFX2, HDGFRP2, SIRT6, TSC1, CEL, TICAM1, SAFB, GBGT1, TLR1, PIGC, DNM3, UGDH, CACTIN, PLIN5, ZNF395, NOTCH1, UHRF1, RPE, PRR5L, LIAS,	1.11

				PDS5A, FHIT, AGPAT2, AK8, GK2, FUT5, FZR1, HMBOX1, RXRA, ADAMTS13, LOC100686484, SOHLH1, ERBB4, NACC2, INPP5E, SAFB2, KANSL1L, LRRC4C, EXTL3, PPP1R26, SURF1, KDM4B, CARD9, NAA11, FMO1, ELP3, FMO2, INHBA, DOHH, TPD52L1, EEF2, FMO4, RAX2, KLHL20, SETX, MSRA, GFI1B, KLHL5, CPS1, LHX3, NFIC, TNFSF4, INTS9, CDK13, UBE2K	
GO:0019438	aromatic compound biosynthetic process	39	0.09	BARHL1, ZNF395, NOTCH1, UHRF1, PRDM6, FASLG, DBH, TTF1, GLI3, AK8, MYDGF, HMBOX1, RXRA, LOC100686484, SOHLH1, ERBB4, NACC2, SNAPC4, SAFB2, WDR5, PIAS4, TLE2, SURF1, RFX2, HDGFRP2, SIRT6, ELP3, INHBA, TICAM1, SAFB, RAX2, SETX, UGDH, GFI1B, LHX3, NFIC, TNFSF4, CDK13, CACTIN	1.26
GO:0006733	oxidoreduction coenzyme metabolic process	4	0.09	RPE, FMO1, SIRT6, FMO2	3.77
GO:2000112	regulation of cellular macromolecule biosynthetic process	36	0.09	BARHL1, ZNF395, NOTCH1, UHRF1, PRDM6, FASLG, PDS5A, GLI3, MYDGF, HMBOX1, RXRA, LOC100686484, ERBB4, NACC2, SNAPC4, SAFB2, WDR5, PIAS4, TLE2, DAPK3, RFX2, SIRT6, TSC1, ELP3, INHBA, TICAM1, EEF2, SAFB, RAX2, SETX, GFI1B, LHX3, NFIC, TNFSF4, CDK13, CACTIN	1.27
GO:0021953	central nervous system neuron differentiation	5	0.09	LHX3, ERBB4, MAP2, INHBA, GLI3	2.92
GO:0002218	activation of innate immune response	4	0.09	TLR1, TLR10, TICAM1, CACTIN	3.68
GO:0002327	immature B cell differentiation	2	0.10	RAG2, RAG1	20.00
GO:0007420	brain development	10	0.10	BARHL1, FZD3, NOTCH1, LHX3, ERBB4, CEP120, TSC1, INHBA, AK8, GLI3	1.83
GO:0016444	somatic cell DNA recombination	3	0.10	TNFSF4, RAG2, RAG1	5.71
GO:0002562	somatic diversification of immune receptors via germline recombination within a single locus	3	0.10	TNFSF4, RAG2, RAG1	5.71
GO:0007167	enzyme linked receptor protein signaling pathway	12	0.10	KLB, FGF5, SETX, NOTCH1, LRG1, MYOC, ERBB4, ADAMTSL2, MATK, TSC1, NRTN, INHBA	1.68
GO:0019538	protein metabolic process	49	0.10	KLB, LDLRAD3, FASLG, RPL9, GLI3, MYDGF, RPL7A, YAE1D1, WDR5, HMG20B, PMPCA, RAG1, PIAS4, CSNK1G3, MAP2K2, MYOC, DAPK3, MATK, SIRT6, TSC1, TICAM1, GBGT1, TLR1, PIGC, CACTIN, UHRF1,	1.20

				PRR5L, LIAS, FHIT, FUT5, FZR1, RXRA, ERBB4, KANSL1L, LRRC4C, KDM4B, CARD9, NAA11, INHBA, DOHH, TPD52L1, EEF2, KLHL20, SETX, MSRA, GFI1B, KLHL5, CDK13, UBE2K	
GO:0050767	regulation of neurogenesis	10	0.10	SETX, SEMA6B, FZD3, NOTCH1, MAP2K2, ERBB4, KIF13B, HMG20B, LRRC4C, GLI3	1.81
GO:0034654	nucleobase-containing compound biosynthetic process	38	0.10	BARHL1, ZNF395, NOTCH1, UHRF1, PRDM6, FASLG, TTF1, GLI3, AK8, MYDGF, HMBOX1, RXRA, LOC100686484, SOHLH1, ERBB4, NACC2, SNAPC4, SAFB2, WDR5, PIAS4, TLE2, SURF1, RFX2, HDGFRP2, SIRT6, ELP3, INHBA, TICAM1, SAFB, RAX2, SETX, UGDH, GFI1B, LHX3, NFIC, TNFSF4, CDK13, CACTIN	1.25
GO:0030900	forebrain development	7	0.10	NOTCH1, LHX3, ERBB4, CEP120, TSC1, INHBA, GLI3	2.19
Molecular functions (GO Terms)		Count	PValue	Genes	Fold Enrichment
GO:0004499	N,N-dimethylaniline monooxygenase activity	5	0.00	FMO1, FMO2, FMO3, FMO4, LOC490346	57.23
GO:0050660	flavin adenine dinucleotide binding	7	0.00	ACADL, FMO1, FMO2, FMO3, FMO4, DUS3L, LOC490346	8.13
GO:0016709	oxidoreductase activity, acting on paired donors, with incorporation or reduction of molecular oxygen, NAD(P)H as one donor, and incorporation of one atom of oxygen	5	0.00	FMO1, FMO2, FMO3, FMO4, LOC490346	16.02
GO:0004497	monooxygenase activity	7	0.00	FMO1, FMO2, DBH, DOHH, FMO3, FMO4, LOC490346	6.76
GO:0050662	coenzyme binding	9	0.00	UGDH, ACADL, FMO1, SIRT6, FMO2, FMO3, FMO4, DUS3L, LOC490346	4.78
GO:0050661	NADP binding	5	0.00	FMO1, FMO2, FMO3, FMO4, LOC490346	11.45
GO:0048037	cofactor binding	10	0.00	UGDH, ACADL, FMO1, SIRT6, FMO2, FMO3, FMO4, DUS3L, SLC9C2, LOC490346	3.68
GO:0016491	oxidoreductase activity	17	0.00	SURF1, HSD11B1L, FMO1, FMO2, DBH, DOHH, FMO3, FMO4, PRDX6, SLC9C2, LOC490346, UGDH, MSRA, ACADL, SARDH, QSOX2, DUS3L	2.28
GO:0042277	peptide binding	7	0.01	TLR1, LOC102151683, LANCL1, LDLRAD3, INHBA, APBA3, PPIC	4.31
GO:0016705	oxidoreductase activity, acting on paired donors, with	7	0.01	FMO1, FMO2, DBH, DOHH, FMO3, FMO4, LOC490346	4.12

	incorporation or reduction of molecular oxygen				
GO:0005488	binding	109	0.01	KLB, FCN2, PRDM6, STKLD1, LDLRAD3, ANTXR2, TTF1, RPL9, SLC9C2, FGF5, ZNF608, PMPCA, RAG2, RAG1, SEMA6B, PIAS4, CSNK1G3, MAP2K2, DAPK3, RFX2, MATK, HDGFRP2, CEL, GBGT1, MYL1, DUS3L, PPIC, ZNF395, NOTCH1, LIAS, AK8, HMBOX1, ADAMTS13, SOHLH1, LOC100686484, MAP2, NACC2, SAFB2, SURF6, ZBTB7A, PPP1R26, FZD3, ZFR2, PRRC2C, INHBA, EEF2, KLHL20, RAX2, SETX, CPS1, LHX3, COL5A1, TNFSF4, PNOC, CDK13, UBE2K, BARHL1, CELF5, DBH, GLI3, RPL7A, CANF2, ACADL, ADAMTSL2, SNAPC4, KIF13B, PRKG2, PGM2, EGFL7, MYOC, RFC1, LANCL1, SIRT6, SAFB, REXO4, TRDN, DNM3, TLR1, UGDH, TBC1D1, DNLZ, PLIN4, VAMP4, CACTIN, LOC491264, RALA, UHRF1, RPE, MRPL54, GNA15, RXRA, ERBB4, KANSL1L, TRIM44, KDM4B, RANBP3, LOC102151683, PCDH7, FMO1, FMO2, ELP3, FMO3, DOHH, FMO4, KLF3, LOC490346, GFI1B, NFIC, APBA3	1.15
GO:0033218	amide binding	7	0.01	TLR1, LOC102151683, LANCL1, LDLRAD3, INHBA, APBA3, PPIC	4.01
GO:0003824	catalytic activity	72	0.01	KLB, STKLD1, DBH, SLC9C2, PTPRG, METTL13, ACADL, ADAMTSL2, KIF13B, WDR5, PGM2, PRKG2, PMPCA, QSOX2, RAG2, RAG1, PIAS4, CSNK1G3, MAP2K2, RFC1, LANCL1, DAPK3, MATK, HSD11B1L, SIRT6, CEL, GBGT1, PIGC, DNM3, UGDH, DUS3L, PPIC, RALA, UHRF1, RPE, GLT6D1, LIAS, FHIT, AGPAT2, ABO, AK8, GK2, FUT5, GNA15, HMBOX1, ADAMTS13, DPP9, ERBB4, NACC2, INPP5E, PIP5K1C, EXTL3, SURF1, KDM4B, NAA11, LOC476732, FMO1, ELP3, FMO2, FMO3, DOHH, EEF2, FMO4, PRDX6, KLHL20, LOC490346, MSRA, CPS1, LOC480074, SARDH, CDK13, UBE2K	1.26
GO:0043565	sequence-specific DNA binding	19	0.01	BARHL1, ZNF395, NOTCH1, RFX2, SAFB, TTF1, GLI3, RAX2, SETX, HMBOX1, RXRA, LHX3, SOHLH1, NFIC, NACC2, SNAPC4, SAFB2, ZBTB7A, RAG1	1.83
GO:0044212	transcription regulatory region DNA binding	16	0.02	BARHL1, ZNF395, NOTCH1, RFX2, SAFB, TTF1, GLI3, SETX, RXRA, SOHLH1, ERBB4, NFIC, NACC2, SNAPC4, KANSL1L, ZBTB7A	1.94

GO:0000975	regulatory region DNA binding	16	0.02	BARHL1, ZNF395, NOTCH1, RFX2, SAFB, TTF1, GLI3, SETX, RXRA, SOHLH1, ERBB4, NFIC, NACC2, SNAPC4, KANSL1L, ZBTB7A	1.93
GO:0001067	regulatory region nucleic acid binding	16	0.02	BARHL1, ZNF395, NOTCH1, RFX2, SAFB, TTF1, GLI3, SETX, RXRA, SOHLH1, ERBB4, NFIC, NACC2, SNAPC4, KANSL1L, ZBTB7A	1.93
GO:0016740	transferase activity	33	0.02	UHRF1, STKLD1, GLT6D1, LIAS, AGPAT2, ABO, AK8, GK2, FUT5, HMBOX1, METTL13, ERBB4, WDR5, PRKG2, PIP5K1C, RAG2, EXTL3, RAG1, PIAS4, CSNK1G3, MAP2K2, DAPK3, NAA11, MATK, SIRT6, ELP3, GBGT1, KLHL20, PIGC, CPS1, LOC480074, CDK13, UBE2K	1.45
GO:0005102	receptor binding	11	0.03	KLB, FGF5, TLR1, SEMA6B, MYOC, TNFSF4, PNOC, MATK, CEL, INHBA, TRDN	2.21
GO:0003677	DNA binding	27	0.03	BARHL1, ZNF395, NOTCH1, UHRF1, TTF1, GLI3, HMBOX1, RXRA, SOHLH1, ERBB4, NACC2, SNAPC4, SAFB2, SURF6, RAG2, KANSL1L, ZBTB7A, RAG1, PIAS4, RFC1, RFX2, HDGFRP2, SAFB, RAX2, SETX, LHX3, NFIC	1.49
GO:0003690	double-stranded DNA binding	14	0.03	BARHL1, ZNF395, UHRF1, RFX2, SAFB, GLI3, SETX, HMBOX1, RXRA, SOHLH1, NFIC, NACC2, SAFB2, ZBTB7A	1.88
GO:0016758	transferase activity, transferring hexosyl groups	6	0.04	PIGC, FUT5, GLT6D1, EXTL3, ABO, GBGT1	3.16
GO:0005515	protein binding	23	0.05	KLB, PPP1R26, SEMA6B, FZD3, MAP2K2, RANBP3, MYOC, MATK, INHBA, CEL, KLHL20, TRDN, FGF5, DNM3, TLR1, TBC1D1, MAP2, TNFSF4, PNOC, DNLZ, KANSL1L, VAMP4, UBE2K	1.48
GO:0016757	transferase activity, transferring glycosyl groups	7	0.06	PIGC, FUT5, SIRT6, GLT6D1, EXTL3, ABO, GBGT1	2.46
GO:0005104	fibroblast growth factor receptor binding	2	0.07	KLB, FGF5	26.71
Cellular component (GO Term)		Count	PValue	Genes	Fold Enrichment
GO:0005720	nuclear heterochromatin	3	0.04	KDM4B, UHRF1, SIRT6	10.06
GO:0044445	cytosolic part	6	0.08	RPL7A, NAA11, SURF6, TSC1, TICAM1, RPL9	2.61
GO:0031974	membrane-enclosed lumen	41	0.08	UHRF1, DBH, TTF1, PDS5A, RPL9, GLI3, RPL7A, FZR1, HMBOX1, RXRA, CHAF1A, MAP2, NACC2, SAFB2, WDR5, SURF6, HMG20B, KANSL1L, PPP1R26, PIAS4, KDM4B, RANBP3, RFC1, MYOC, SURF2, DAPK3,	1.26

				WDR19, SIRT6, ELP3, SAFB, REXO4, KLHL20, SETX, UGDH, MSRA, GFI1B, CPS1, NFIC, INTS9, CDK13, CACTIN	
GO:0031981	nuclear lumen	38	0.09	UHRF1, TTF1, PDS5A, RPL9, GLI3, RPL7A, FZR1, HMBOX1, RXRA, CHAF1A, MAP2, NACC2, SAFB2, WDR5, SURF6, HMG20B, KANSL1L, PPP1R26, PIAS4, KDM4B, RANBP3, RFC1, SURF2, DAPK3, WDR19, SIRT6, ELP3, SAFB, REXO4, KLHL20, SETX, UGDH, MSRA, GFI1B, NFIC, INTS9, CDK13, CACTIN	1.27
GO:0032433	filopodium tip	2	0.09	FZD3, UBE2K	20.11
GO:0031248	protein acetyltransferase complex	4	0.10	NAA11, WDR5, ELP3, KANSL1L	3.66
GO:1902493	acetyltransferase complex	4	0.10	NAA11, WDR5, ELP3, KANSL1L	3.66

Supplementary Table S3. The list of significant GO terms, KEGG pathways and the associated genes in either of two pairwise comparisons (Bullmastiff vs Bulldog and Bullmastiff vs Mastiff)

Function	Group	Group Genes
phosphotyrosine residue binding	1	PTPN3|SHD|VAV2
filopodium tip	2	ABITRAM|FZD3|UBE2K
positive regulation of RNA splicing	3	NCL|PRDX6|SETX
histone acetyltransferase binding	4	GLI3|KANSL1L|ZBTB7A
positive regulation of organ growth	5	ERBB4|NOTCH1|RAG2
single-stranded RNA binding	6	DIS3L2|HNRNPU|LONP1|ZC3H14|ZFR2
lipid droplet	7	PLIN3|PLIN4|PLIN5|TRAF6|TSC1
pyrimidine-containing compound transmembrane transport	8	LOC486150|LOC486151|SLC25A33
organic hydroxy compound catabolic process	9	CEL|DBH|GK2|INPP5E
regulation of protein polyubiquitination	10	FOXF2|TRIM44|TRIP12
regulation of ATP metabolic process	11	PID1|SLC25A33|SLC2A6|ZBTB7A
transcription by RNA polymerase III	12	CEL|GTF3C4|SNAPC4
positive regulation of extrinsic apoptotic signaling pathway	13	INHBA|ITM2C|TRAF2
Pentose phosphate pathway	14	H6PD|PGM2|RPE

AMPK signaling pathway	15	CAB39|CREB3L3|EEF2|PIK3CD|TBC1D1|TSC1
fibroblast growth factor receptor binding	16	FGF5|FLRT2|KLB
Gap junction	17	HTR2B|MAP2K2|PRKG2|TUBB4B
Cocaine addiction	18	CREB3L3|GPSM1|GRIN1
cytochrome complex assembly	19	COX20|SLC25A33|SURF1
positive regulation of endothelial cell proliferation	20	HTR2B|LRG1|MYDGF|PIK3CD|SIRT6
hippocampus development	21	GLI3|PTPRS|SEMA6B|TSC1
lipid glycosylation	22	ABO|GBGT1|GLT6D1
positive regulation of cytosolic calcium ion concentration involved in phospholipase C-activating G protein-coupled signaling pathway	23	GNA15|GPR55|GPR65|HTR2B
cellular response to epidermal growth factor stimulus	24	DUSP22|ERBB4|NCL
mitochondrial fission	25	DNLZ|DNM3|MFF|PMPCA|RALA
regulation of protein localization to cilium	26	ENTR1|GAS8|INPP5E|SPATA7
muscle hypertrophy	27	HTR2B|MYMK|MYOC|NOTCH1
Protein digestion and absorption	28	COL4A3|COL5A1|COL6A5|COL6A6|LOC610614
retinoid binding	29	LOC607807|PTGDS|RBP7|RXRA
neural tube closure	30	FZD3|LIAS|RALA|TRAF6|TSC1
regulation of focal adhesion assembly	31	DAPK3|DUSP22|MYOC|TSC1
Amino sugar and nucleotide sugar metabolism	32	GMDS|LOC606974|PGM2|UGDH
PPAR signaling pathway	33	ACADL|ACSBG2|GK2|LOC485024|PLIN4|PLIN5|RXRA|TRAF2
nuclear matrix	34	AHCTF1|CHMP1A|FSD1|GFI1B|HNRNPU|PIAS4|UHRF1
Small cell lung cancer	35	COL4A3|FASLG|FHIT|IRF4|MAP2K2|PIK3CD|PIP5K1C|RXRA|TICAM1|TRAF2|TRAF6|VAV2
negative regulation of cell migration involved in sprouting angiogenesis	36	KLF4|NOTCH1|PTPRG|SP100|TBXA2R
regulation of transcription elongation from RNA polymerase II promoter	37	CDK13|EAF1|HNRNPU|NELFB|NOTCH1
N,N-dimethylaniline monooxygenase activity	38	ACAD11|ACADL|DBH|DOHH|DPEP1|DUS3L|FMO1|FMO2|FMO3|FMO4|GMDS|H6PD|HTR2B|LOC490346|NDOR1
negative regulation of interleukin-8 production	39	CACTIN|FCN2|KLF4|MAP2K2|PIK3CD|PTPRS|TICAM1|TLR1|TLR10|TLR6|TRAF6

alpha-(1->3)-fucosyltransferase activity	40	ANAPC2|FANCA|FUT5|FUT7|KLF4|NOTCH1|TRAF6|ZBTB7A
tumor necrosis factor receptor superfamily binding	41	ANAPC2|CARD9|COL4A3|FASLG|FHIT|FZR1|IRF4|MAP2K2|MATK|PIAS4|PIK3CD|RXRA|TICAM1|TNFSF18|TNFSF4|TRAF2|TRAF6|TRIP12|UBE2K|UBE4B
Phospholipase D signaling pathway	42	AGPAT2|CACTIN|COL4A3|DAPK3|DNM3|FASLG|FGF5|FHIT|GNA15|LOC489647|MAP2K2|MATK|MED27|NOTCH1|PIK3CD|PIK3R4|PIP5K1C|PTPRS|RALA|RALGDS|RXRA|TICAM1|TLR1|TLR10|TLR6|TNFSF18|TNFSF4|TRAF2|TRAF6|TSC1|VAV2

Supplementary Table S4 Summary of significant GO terms, KEGG pathways and involved genes for pairwise comparison of Bullmastiff vs European Mastiff reference clade

Function	Groups	Group Genes
branching involved in blood vessel morphogenesis	1	NRARP|SEMA3E|SIRT6
negative regulation of histone modification	2	KDM4B|TRIP12|WDR5
transcription by RNA polymerase III	3	CEL|GTF3C4|SNAPC4
negative regulation of cytokine-mediated signaling pathway	4	CACTIN|PIAS4|SIGIRR
regulation of vasoconstriction	5	DBH|P2RX1|TBXA2R
lipid glycosylation	6	ABO|B4GALNT4|GBGT1|GLT6D1
response to ATP	7	P2RX1|P2RX5|TRPV1
negative regulation of toll-like receptor signaling pathway	8	CACTIN|GPR108|NLRP6|PTPRS
negative regulation of Notch signaling pathway	9	ARRDC1|NOTCH1|NRARP|ZBTB7A
PPAR signaling pathway	10	ACSBG2|ANGPTL4|LOC485024|PLIN4|PLIN5|RXRA|TRAF2
positive regulation of cytokine-mediated signaling pathway	11	PAFAH1B1|TRAF2|TRIM44
programmed necrotic cell death	12	ALKBH7|NLRP6|TRAF2
response to acidic pH	13	ACER1|GPR65|RAB11B|TRPV1
retinoid binding	14	LOC607807|PTGDS|RXRA
regulation of transcription elongation from RNA polymerase II promoter	15	CDK13|GTF2F1|NELFB
Choline metabolism in cancer	16	CREB3L3|EEF2|EHMT1|ELAVL1|HRAS|MAP2K2|PIP5K1C|RAB11B|RALGDS|TSC1
lipid droplet	17	ALKBH7|C3|PLIN3|PLIN4|PLIN5|PNPLA7|TRAF6|TSC1
phosphotyrosine residue binding	18	HRAS|MAP2K2|PAFAH1B1|SH2D3A|SHD|TRPV1|VAV1|VAV2
sulfur compound transmembrane transporter activity	19	CTNS|DBH|LOC486150|LOC486151|MFSD12|RIC8A
Sphingolipid signaling pathway	20	ACER1|CERS4|DAPK3|GALC|GRIN1|HRAS|MAP2K2|RXRA|S1PR4|TRAF2
regulation of viral entry into host cell	21	CACTIN|FCN2|LOC475935|LOC483397
ceramide metabolic process	22	ACER1|ANAPC2|CEL|CERS4|FUT7|GALC|GBGT1|HRAS|MAP2K2|P2RX1|S1PR4|TRAF2
negative regulation of axonogenesis	23	GLI3|NOTCH1|PAFAH1B1|PTPRS|SEMA3A|SEMA3D|SEMA3E|SEMA6B|TSC1

Yersinia infection	24	C3|CACTIN|CCL20|CD70|ELAVL1|HRAS|MAP2K2|PIAS4|PIP5K1C|TICAM1|TNFSF14|TRAF2|TRAF6|VAV1|VAV2
acute inflammatory response to antigenic stimulus	25	ANAPC2|B4GALNT4|BET1L|C3|FUT5|FUT7|NLRP6|NOTCH1|PID1|SIGIRR|TRAF6|TRPV1
semaphorin receptor binding	26	FLRT2|GLI3|NOTCH1|NSMF|PAFAH1B1|PTPRS|SEMA3A|SEMA3D|SEMA3E|SEMA6B|TSC1
Phospholipase D signaling pathway	27	ACER1|AGPAT2|ATP2A3|C3|CACTIN|CERS4|CREB3L3|DAPK3|EHMT1|GRIN1|HRAS|MAP2K2|MED27|NOTCH1|PHPT1|PIAS4|PIP5K1C|RALA|RALGDS|RXRA|S1PR4|SEMA3E|SH2D3A|SHD|SIRT3|SIRT6|TICAM1|TNFSF14|TRAF2|TRAF6|TSC1|TUBB4A|TUBB4B|VAV1|VAV2

Supplementary Table S5. Overall view of functional classification of candidate gene in Bullmastiffs compared to other breeds within the European Mastiff clade by GO terms and KEGG pathways.

KEGG(Term)		Count	PValue	Genes	Fold Enrichment
cfa04920	Adipocytokine signaling pathway	5	0.02	RXRA, LOC485024, LOC476732, TRAF2, CAMKK1	4.44
cfa04020	Calcium signaling pathway	7	0.06	GNA15, P2RX5, TBXA2R, P2RX1, CACNA1B, ATP2A3, GRIN1	2.47
cfa00601	Glycosphingolipid biosynthesis - lacto and neolacto series	3	0.07	FUT5, FUT7, ABO	7.01
cfa01100	Metabolic pathways	26	0.07	NDUFA11, DBH, HSD17B12, AGPAT2, ABO, AK8, FUT5, PTDSS2, FUT7, INPP5E, PIP5K1C, PTGDS, LOC480667, CERS4, NDUFA7, LOC476732, TALDO1, CEL, GBGT1, GALC, ACER1, SARDH, LOC485024, LOC611724, ASPA, PAFAH1B1	1.38
Biological Process (GO Term)		Count	PValue	Genes	Fold Enrichment
GO:0008542	visual learning	5	0.00	RIC8A, DEAF1, CTNS, DBH, GRIN1	7.87
GO:0007632	visual behavior	5	0.00	RIC8A, DEAF1, CTNS, DBH, GRIN1	7.48
GO:0009268	response to pH	4	0.01	ACER1, GPR65, TRPV1, RAB11B	10.87
GO:0050922	negative regulation of chemotaxis	5	0.01	SEMA6B, NOTCH1, SEMA3D, SEMA3A, SEMA3E	6.36
GO:0048843	negative regulation of axon extension involved in axon guidance	4	0.01	SEMA6B, SEMA3D, SEMA3A, SEMA3E	9.57
GO:1902668	negative regulation of axon guidance	4	0.01	SEMA6B, SEMA3D, SEMA3A, SEMA3E	9.20
GO:0072348	sulfur compound transport	3	0.01	LOC486150, SLC19A3, CTNS	19.94
GO:0001755	neural crest cell migration	5	0.01	SEMA6B, SEMA3D, SEMA3A, SEMA3E, NRTN	5.86
GO:0008306	associative learning	5	0.01	RIC8A, DEAF1, CTNS, DBH, GRIN1	5.44
GO:0046514	ceramide catabolic process	3	0.01	GALC, ACER1, CEL	16.31

GO:0001959	regulation of cytokine-mediated signaling pathway	5	0.01	SIGIRR, PIAS4, TRAF2, CACTIN, PAFAH1B1	5.25
GO:0050770	regulation of axonogenesis	7	0.02	SEMA6B, MAP2K2, SEMA3D, SEMA3A, SEMA3E, LRRC4C, GRIN1	3.43
GO:0030217	T cell differentiation	8	0.02	FUT7, TNFSF9, TSC1, NRARP, RAG2, GLI3, VAV1, RAG1	3.01
GO:0048846	axon extension involved in axon guidance	4	0.02	SEMA6B, SEMA3D, SEMA3A, SEMA3E	7.25
GO:0048841	regulation of axon extension involved in axon guidance	4	0.02	SEMA6B, SEMA3D, SEMA3A, SEMA3E	7.25
GO:1902284	neuron projection extension involved in neuron projection guidance	4	0.02	SEMA6B, SEMA3D, SEMA3A, SEMA3E	7.25
GO:0008361	regulation of cell size	7	0.02	RAP1GAP2, SEMA6B, SEMA3D, SEMA3A, TSC1, SEMA3E, VAV1	3.38
GO:0031175	neuron projection development	17	0.02	RAP1GAP2, SEMA6B, NOTCH1, MAP2K2, SEMA3D, SEMA3A, SEMA3E, GLI3, CAMSAP1, GRIN1, SETX, TTC8, ATCAY, FLRT2, NRTN, LRRC4C, PAFAH1B1	1.89
GO:0045664	regulation of neuron differentiation	13	0.02	RAP1GAP2, SEMA6B, NOTCH1, MAP2K2, SEMA3D, SEMA3A, SEMA3E, GLI3, GRIN1, SETX, HMG20B, LRRC4C, PAFAH1B1	2.15
GO:0060759	regulation of response to cytokine stimulus	5	0.02	SIGIRR, PIAS4, TRAF2, CACTIN, PAFAH1B1	4.90
GO:0006672	ceramide metabolic process	5	0.02	GALC, ACER1, CERS4, P2RX1, CEL	4.90
GO:0030517	negative regulation of axon extension	4	0.02	SEMA6B, SEMA3D, SEMA3A, SEMA3E	7.04
GO:2000241	regulation of reproductive process	6	0.02	FZR1, NOTCH1, RXRA, SEMA3A, INHBA, PTGDS	3.90
GO:0014032	neural crest cell development	5	0.02	SEMA6B, SEMA3D, SEMA3A, SEMA3E, NRTN	4.82
GO:0030030	cell projection organization	24	0.02	RAP1GAP2, SEMA6B, RALA, NOTCH1, MAP2K2, SEMA3D, SEMA3A, SSNA1, RFX2, TSC1, SEMA3E, TMEM141, GLI3, CAMSAP1, TTC17, GRIN1, SETX, TTC8, ATCAY, FLRT2, EPS8L2, NRTN, LRRC4C, PAFAH1B1	1.63
GO:0071526	semaphorin-plexin signaling pathway	4	0.02	SEMA6B, SEMA3D, SEMA3A, SEMA3E	6.65

GO:1902667	regulation of axon guidance	4	0.02	SEMA6B, SEMA3D, SEMA3A, SEMA3E	6.65
GO:0019221	cytokine-mediated signaling pathway	9	0.03	SIGIRR, CCL25, PIAS4, FLRT2, CCL20, TRAF2, CACTIN, LRRC4C, PAFAH1B1	2.55
GO:0010447	response to acidic pH	3	0.03	GPR65, TRPV1, RAB11B	11.96
GO:0007166	cell surface receptor signaling pathway	37	0.03	NOTCH1, LOC611660, SEMA3D, SSNA1, SEMA3A, SEMA3E, NRARP, ITGAE, GLI3, ADGRE1, ADAMTS13, FLRT2, ADAMTSL2, NRTN, LRRC4C, CCL25, SEMA6B, PIAS4, EGFL7, CCL20, CD70, DAPK3, MATK, EMR4, TRAF2, TSC1, INHBA, TICAM1, VAV1, GRIN1, SIGIRR, SETX, LRG1, TAX1BP3, PHPT1, CACTIN, PAFAH1B1	1.42
GO:0045665	negative regulation of neuron differentiation	7	0.03	RAP1GAP2, SEMA6B, NOTCH1, SEMA3D, SEMA3A, SEMA3E, GLI3	3.08
GO:0014033	neural crest cell differentiation	5	0.03	SEMA6B, SEMA3D, SEMA3A, SEMA3E, NRTN	4.33
GO:0030149	sphingolipid catabolic process	3	0.03	GALC, ACER1, CEL	11.21
GO:0010975	regulation of neuron projection development	10	0.03	RAP1GAP2, SETX, SEMA6B, MAP2K2, SEMA3D, SEMA3A, SEMA3E, LRRC4C, GRIN1, PAFAH1B1	2.31
GO:0071934	thiamine transmembrane transport	2	0.03	LOC486150, SLC19A3	59.81
GO:0031344	regulation of cell projection organization	12	0.03	RAP1GAP2, SETX, SEMA6B, RALA, MAP2K2, SEMA3D, SEMA3A, EPS8L2, SEMA3E, LRRC4C, GRIN1, PAFAH1B1	2.04
GO:0050767	regulation of neurogenesis	14	0.03	RAP1GAP2, SEMA6B, NOTCH1, MAP2K2, SEMA3D, SEMA3A, SEMA3E, GLI3, GRIN1, SETX, FLRT2, HMG20B, LRRC4C, PAFAH1B1	1.89
GO:0046466	membrane lipid catabolic process	3	0.04	GALC, ACER1, CEL	9.97
GO:0033198	response to ATP	3	0.04	P2RX5, P2RX1, TRPV1	9.44
GO:0048265	response to pain	3	0.04	CACNA1B, DBH, TRPV1	9.44
GO:0022603	regulation of anatomical structure morphogenesis	19	0.04	SEMA6B, NOTCH1, MAP2K2, RNH1, TNFSF14, SEMA3D, SEMA3A, DAPK3, SEMA3E, NRARP, CAMSAP1, GRIN1, C3, MYDGF, RXRA, LRG1, COL5A1, LRRC4C, PAFAH1B1	1.64
GO:0051674	localization of cell	24	0.04	SEMA6B, BARHL1, CCL25, NOTCH1, MAP2K2, TNFSF14, CCL20, SEMA3D, SEMA3A, DAPK3, MATK, SEMA3E, PRR5L, DBH, GLI3, VAV1, RIC8A, FUT7, FLRT2, COL5A1, CATSPERD, NRTN, PHPT1, PAFAH1B1	1.53

GO:0048870	cell motility	24	0.04	SEMA6B, BARHL1, CCL25, NOTCH1, MAP2K2, TNFSF14, CCL20, SEMA3D, SEMA3A, DAPK3, MATK, SEMA3E, PRR5L, DBH, GLI3, VAV1, RIC8A, FUT7, FLRT2, COL5A1, CATSPERD, NRTN, PHPT1, PAFAH1B1	1.53
GO:0007411	axon guidance	7	0.04	SEMA6B, TTC8, FLRT2, SEMA3D, SEMA3A, SEMA3E, GLI3	2.75
GO:0032102	negative regulation of response to external stimulus	8	0.04	SEMA6B, NOTCH1, NLRP6, SEMA3D, SEMA3A, SEMA3E, CACTIN, GRIN1	2.49
GO:0097485	neuron projection guidance	7	0.04	SEMA6B, TTC8, FLRT2, SEMA3D, SEMA3A, SEMA3E, GLI3	2.74
GO:0010769	regulation of cell morphogenesis involved in differentiation	9	0.04	SEMA6B, NOTCH1, MAP2K2, SEMA3D, SEMA3A, SEMA3E, LRRC4C, GRIN1, PAFAH1B1	2.29
GO:0021987	cerebral cortex development	5	0.04	SEMA3A, TSC1, GLI3, GRIN1, PAFAH1B1	3.74
GO:0031324	negative regulation of cellular metabolic process	32	0.05	UHRF1, EHMT1, MLLT1, PRR5L, NRARP, GLI3, RXRA, ATCAY, FLRT2, NLRP6, DEAF1, NACC2, HMG20B, NELFB, LRRC4C, ZC3H14, RAG1, PPP1R26, PIAS4, KDM4B, LOC100688619, DAPK3, SIRT6, TSC1, TRPV1, SIRT3, SIGIRR, NFIC, TRIP12, CACTIN, PAFAH1B1, PLIN5	1.40
GO:0001938	positive regulation of endothelial cell proliferation	4	0.05	MYDGF, EGFL7, LRG1, NRARP	4.98
GO:0007159	leukocyte cell-cell adhesion	11	0.05	CCL25, FUT7, TNFSF14, EBI3, TNFSF9, TSC1, NRARP, RAG2, GLI3, VAV1, RAG1	2.02
GO:2000249	regulation of actin cytoskeleton reorganization	3	0.05	DAPK3, SEMA3E, PHPT1	8.54
GO:0050771	negative regulation of axonogenesis	4	0.05	SEMA6B, SEMA3D, SEMA3A, SEMA3E	4.88
GO:0006935	chemotaxis	12	0.05	CCL25, SEMA6B, TTC8, NOTCH1, FLRT2, TNFSF14, CCL20, SEMA3D, SEMA3A, SEMA3E, GLI3, VAV1	1.91
GO:0042330	taxis	12	0.05	CCL25, SEMA6B, TTC8, NOTCH1, FLRT2, TNFSF14, CCL20, SEMA3D, SEMA3A, SEMA3E, GLI3, VAV1	1.91
GO:0015888	thiamine transport	2	0.05	LOC486150, SLC19A3	39.87
GO:0060528	secretory columnal luminar epithelial cell	2	0.05	NOTCH1, RXRA	39.87

	differentiation involved in prostate glandular acinus development				
GO:0002331	pre-B cell allelic exclusion	2	0.05	RAG2, RAG1	39.87
GO:0071356	cellular response to tumor necrosis factor	5	0.05	CCL25, PIAS4, CCL20, TRAF2, CACTIN	3.56
GO:0007409	axonogenesis	10	0.05	SEMA6B, TTC8, NOTCH1, MAP2K2, FLRT2, SEMA3D, SEMA3A, SEMA3E, GLI3, PAFAH1B1	2.07
GO:0070489	T cell aggregation	10	0.05	FUT7, TNFSF14, EBI3, TNFSF9, TSC1, NRARP, RAG2, GLI3, VAV1, RAG1	2.07
GO:0042110	T cell activation	10	0.05	FUT7, TNFSF14, EBI3, TNFSF9, TSC1, NRARP, RAG2, GLI3, VAV1, RAG1	2.07
GO:0010810	regulation of cell-substrate adhesion	6	0.05	CCL25, NOTCH1, DAPK3, TSC1, SEMA3E, HSD17B12	2.94
GO:0071593	lymphocyte aggregation	10	0.05	FUT7, TNFSF14, EBI3, TNFSF9, TSC1, NRARP, RAG2, GLI3, VAV1, RAG1	2.06
GO:0010977	negative regulation of neuron projection development	5	0.06	RAP1GAP2, SEMA6B, SEMA3D, SEMA3A, SEMA3E	3.48
GO:0021537	telencephalon development	7	0.06	TTC8, SEMA3A, TSC1, INHBA, GLI3, GRIN1, PAFAH1B1	2.57
GO:0033555	multicellular organismal response to stress	4	0.06	DEAF1, CACNA1B, DBH, TRPV1	4.60
GO:0006643	membrane lipid metabolic process	6	0.06	GALC, ACER1, CERS4, P2RX1, CEL, GBGT1	2.89
GO:0070486	leukocyte aggregation	10	0.06	FUT7, TNFSF14, EBI3, TNFSF9, TSC1, NRARP, RAG2, GLI3, VAV1, RAG1	2.03
GO:0007612	learning	5	0.06	RIC8A, DEAF1, CTNS, DBH, GRIN1	3.40
GO:2000147	positive regulation of cell motility	10	0.06	SEMA6B, NOTCH1, MAP2K2, TNFSF14, CCL20, SEMA3D, SEMA3A, DAPK3, SEMA3E, PHPT1	2.01
GO:0050768	negative regulation of neurogenesis	7	0.06	RAP1GAP2, SEMA6B, NOTCH1, SEMA3D, SEMA3A, SEMA3E, GLI3	2.51
GO:0050789	regulation of biological process	136	0.06	RNH1, LOC611660, GPR65, EHMT1, LDLRAD3, ALKBH7, COR1A3, ATCAY, KHSRP, NRTN, OR3A10, PTGDS, RAG2, RAG1, SEMA6B, PIAS4, ARRDC5, TLE2, MAP2K2, LOC100688619, ENTPD2, DAPK3,	1.09

				RFX2, MATK, HDGFRP2, TSC1, CEL, TICAM1, GTF2F1, LOC491216, SIGIRR, PHPT1, COR1R4, RABL6, FBN3, NOTCH1, CACNA1B, CAMSAP1, RIC8A, C3, GNG10, ADAMTS13, FLRT2, NLRP6, DEAF1, TBXA2R, NACC2, SAFB2, LRRC4C, ZC3H14, RALGDS, PPP1R26, RAP1GAP2, ABCA2, LOC611352, SPHKAP, TNFSF14, API5, SURF4, TRPV3, TRPV1, INHBA, EEF2, RAX2, SETX, ACER1, COL5A1, TNFSF9, CDK13, COR1P2, CD320, BARHL1, NRARP, DBH, ITGAE, ELAVL1, GLI3, ADGRE1, MYDGF, GSG2, ADAMTSL2, SNAPC4, WDR5, HMG20B, NELFB, EGFL7, SH2D3A, LMNTD2, ANO9, TRAF2, SIRT6, SAFB, MED27, BET1L, VAV1, SIRT3, TAX1BP3, ANGPTL4, CACTIN, PLIN5, PAFAH1B1, SDCCAG3, RALA, OR1E2, SEMA3D, UHRF1, SEMA3A, SSNA1, PRR5L, SEMA3E, MLLT1, HSD17B12, AGPAT2, GNA15, FZR1, RXRA, NCLN, TMEM203, EPS8L2, SLC25A23, S1PR4, CCL25, KDM4B, RANBP3, CCL20, CD70, EMR4, RAB11B, GRIN1, GFI1B, TTC8, LRG1, NFIC, P2RX1, TRIP12, APBA3	
GO:0006357	regulation of transcription from RNA polymerase II promoter	27	0.06	BARHL1, NOTCH1, UHRF1, EHMT1, NRARP, GLI3, MYDGF, RXRA, DEAF1, NACC2, SNAPC4, SAFB2, WDR5, NELFB, PIAS4, ABCA2, LOC100688619, RFX2, TRPV1, INHBA, MED27, SAFB, GTF2F1, GRIN1, SETX, NFIC, CDK13	1.42
GO:0050907	detection of chemical stimulus involved in sensory perception	6	0.06	LOC611352, OR1E2, TRPV1, LOC491216, COR1R4, COR1P2	2.78
GO:0014031	mesenchymal cell development	6	0.06	SEMA6B, NOTCH1, SEMA3D, SEMA3A, SEMA3E, NRTN	2.78
GO:0061564	axon development	10	0.06	SEMA6B, TTC8, NOTCH1, MAP2K2, FLRT2, SEMA3D, SEMA3A, SEMA3E, GLI3, PAFAH1B1	1.99
GO:0035461	vitamin transmembrane transport	2	0.06	LOC486150, SLC19A3	29.91
GO:0006665	sphingolipid metabolic process	5	0.07	GALC, ACER1, CERS4, P2RX1, CEL	3.25
GO:0016337	single organismal cell-cell adhesion	14	0.07	CCL25, TNFSF14, EBI3, TSC1, NRARP, CEL, GLI3, VAV1, RIC8A, FUT7, TNFSF9, PKP3, RAG2, RAG1	1.70
GO:0048729	tissue morphogenesis	13	0.07	RALA, NOTCH1, SEMA3A, TSC1, SEMA3E, NRARP, INHBA, GLI3, TTC8, RXRA, COL5A1, DEAF1, PAFAH1B1	1.75
GO:0051272	positive regulation of cellular component movement	10	0.07	SEMA6B, NOTCH1, MAP2K2, TNFSF14, CCL20, SEMA3D, SEMA3A, DAPK3, SEMA3E, PHPT1	1.97

GO:0043087	regulation of GTPase activity	7	0.07	CCL25, TTC8, CCL20, GPR65, TSC1, TAX1BP3, PAFAH1B1	2.42
GO:0016477	cell migration	21	0.07	SEMA6B, BARHL1, CCL25, NOTCH1, TNFSF14, CCL20, SEMA3D, SEMA3A, DAPK3, MATK, SEMA3E, PRR5L, DBH, GLI3, VAV1, RIC8A, FUT7, FLRT2, COL5A1, NRTN, PAFAH1B1	1.49
GO:0071345	cellular response to cytokine stimulus	10	0.07	SIGIRR, CCL25, PIAS4, FLRT2, CCL20, DAPK3, TRAF2, CACTIN, LRRC4C, PAFAH1B1	1.95
GO:0007611	learning or memory	6	0.07	RIC8A, DEAF1, CTNS, DBH, GRIN1, PAFAH1B1	2.70
GO:0040017	positive regulation of locomotion	10	0.07	SEMA6B, NOTCH1, MAP2K2, TNFSF14, CCL20, SEMA3D, SEMA3A, DAPK3, SEMA3E, PHPT1	1.94
GO:0040011	locomotion	25	0.07	BARHL1, NOTCH1, SEMA3D, SEMA3A, PRR5L, SEMA3E, DBH, GLI3, RIC8A, FUT7, FLRT2, NRTN, CCL25, SEMA6B, MAP2K2, TNFSF14, CCL20, DAPK3, MATK, VAV1, TTC8, COL5A1, CATSPERD, PHPT1, PAFAH1B1	1.41
GO:0001960	negative regulation of cytokine-mediated signaling pathway	3	0.07	SIGIRR, PIAS4, CACTIN	6.65
GO:0050919	negative chemotaxis	3	0.07	SEMA3D, SEMA3A, SEMA3E	6.65
GO:0048666	neuron development	17	0.08	RAP1GAP2, SEMA6B, NOTCH1, MAP2K2, SEMA3D, SEMA3A, SEMA3E, GLI3, CAMSAP1, GRIN1, SETX, TTC8, ATCAY, FLRT2, NRTN, LRRC4C, PAFAH1B1	1.56
GO:0034612	response to tumor necrosis factor	5	0.08	CCL25, PIAS4, CCL20, TRAF2, CACTIN	3.12
GO:0002437	inflammatory response to antigenic stimulus	3	0.08	C3, NOTCH1, NLRP6	6.41
GO:0051239	regulation of multicellular organismal process	39	0.08	RNH1, NOTCH1, SEMA3D, SEMA3A, CACNA1B, SEMA3E, NRARP, DBH, ELAVL1, AGPAT2, GLI3, C3, MYDGF, RXRA, FLRT2, NLRP6, TBXA2R, DEAF1, HMG20B, PTGDS, RAG2, LRRC4C, RAG1, RAP1GAP2, SEMA6B, MAP2K2, TRPV3, TRAF2, INHBA, TICAM1, GRIN1, SIGIRR, SETX, LRG1, COL5A1, P2RX1, TNFSF9, CACTIN, PAFAH1B1	1.28
GO:0097466	glycoprotein ERAD pathway	2	0.08	LOC607002, LOC480667	23.92
GO:0002329	pre-B cell differentiation	2	0.08	RAG2, RAG1	23.92
GO:0072531	pyrimidine-containing compound	2	0.08	LOC486150, SLC19A3	23.92

	transmembrane transport				
GO:1904587	response to glycoprotein	2	0.08	LOC607002, LOC480667	23.92
GO:0002009	morphogenesis of an epithelium	11	0.08	RALA, TTC8, NOTCH1, COL5A1, DEAF1, SEMA3A, TSC1, SEMA3E, NRARP, GLI3, PAFAH1B1	1.82
GO:0060761	negative regulation of response to cytokine stimulus	3	0.08	SIGIRR, PIAS4, CACTIN	6.19
GO:0051961	negative regulation of nervous system development	7	0.08	RAP1GAP2, SEMA6B, NOTCH1, SEMA3D, SEMA3A, SEMA3E, GLI3	2.30
GO:0009892	negative regulation of metabolic process	32	0.08	UHRF1, EHMT1, MLLT1, PRR5L, NRARP, GLI3, RXRA, ATCAY, FLRT2, NLRP6, DEAF1, NACC2, HMG20B, NELFB, LRRC4C, ZC3H14, RAG1, PPP1R26, PIAS4, KDM4B, LOC100688619, DAPK3, SIRT6, TSC1, TRPV1, SIRT3, SIGIRR, NFIC, TRIP12, CACTIN, PAFAH1B1, PLIN5	1.32
GO:0031345	negative regulation of cell projection organization	5	0.09	RAP1GAP2, SEMA6B, SEMA3D, SEMA3A, SEMA3E	2.96
GO:0030098	lymphocyte differentiation	8	0.09	FUT7, TNFSF9, TSC1, NRARP, RAG2, GLI3, VAV1, RAG1	2.09
GO:0048762	mesenchymal cell differentiation	6	0.09	SEMA6B, NOTCH1, SEMA3D, SEMA3A, SEMA3E, NRTN	2.53
GO:0031400	negative regulation of protein modification process	11	0.09	KDM4B, FLRT2, NLRP6, HMG20B, MLLT1, PRR5L, TRIP12, CACTIN, LRRC4C, PAFAH1B1, SIRT3	1.78
GO:0051960	regulation of nervous system development	14	0.09	RAP1GAP2, SEMA6B, NOTCH1, MAP2K2, SEMA3D, SEMA3A, SEMA3E, GLI3, GRIN1, SETX, FLRT2, HMG20B, LRRC4C, PAFAH1B1	1.62
GO:0050920	regulation of chemotaxis	6	0.09	SEMA6B, NOTCH1, TNFSF14, SEMA3D, SEMA3A, SEMA3E	2.49
GO:0021761	limbic system development	4	0.09	SEMA3A, TSC1, GLI3, PAFAH1B1	3.68
GO:0030516	regulation of axon extension	4	0.09	SEMA6B, SEMA3D, SEMA3A, SEMA3E	3.68
GO:1902231	positive regulation of intrinsic apoptotic signaling pathway in	2	0.10	PIAS4, NACC2	19.94

	response to DNA damage				
GO:0001952	regulation of cell-matrix adhesion	4	0.10	CCL25, DAPK3, TSC1, SEMA3E	3.62
GO:0006928	movement of cell or subcellular component	27	0.10	BARHL1, NOTCH1, SEMA3D, SSNA1, SEMA3A, PRR5L, SEMA3E, DBH, TMEM141, GLI3, RIC8A, FUT7, FLRT2, NRTN, CCL25, SEMA6B, MAP2K2, TNFSF14, CCL20, DAPK3, MATK, VAV1, TTC8, COL5A1, CATSPERD, PHPT1, PAFAH1B1	1.35
GO:0010883	regulation of lipid storage	3	0.10	C3, ALKBH7, PLIN5	5.61
Molecular functions (GO Terms)		Count	PValue	Genes	Fold Enrichment
GO:0045499	chemorepellent activity	5	0.00	SEMA6B, FLRT2, SEMA3D, SEMA3A, SEMA3E	12.04
GO:0030215	semaphorin receptor binding	4	0.00	SEMA6B, SEMA3D, SEMA3A, SEMA3E	12.67
GO:0016811	hydrolase activity, acting on carbon-nitrogen (but not peptide) bonds, in linear amides	5	0.01	ACER1, NACC2, SIRT6, ASPA, SIRT3	6.84
GO:0038191	neuropilin binding	3	0.01	SEMA3D, SEMA3A, SEMA3E	22.57
GO:0004407	histone deacetylase activity	3	0.03	NACC2, SIRT6, SIRT3	10.03
GO:0033558	protein deacetylase activity	3	0.05	NACC2, SIRT6, SIRT3	8.60
GO:0015234	thiamine transmembrane transporter activity	2	0.05	LOC486150, SLC19A3	40.13
GO:0015403	thiamine uptake transmembrane transporter activity	2	0.05	LOC486150, SLC19A3	40.13
GO:0015563	uptake transmembrane transporter activity	2	0.05	LOC486150, SLC19A3	40.13
GO:0005231	excitatory extracellular ligand-gated ion channel activity	4	0.05	P2RX5, P2RX1, TRPV1, GRIN1	4.63

GO:1901682	sulfur compound transmembrane transporter activity	3	0.06	LOC486150, SLC19A3, CTNS	7.52
GO:0004857	enzyme inhibitor activity	9	0.06	PPP1R26, C3, NOTCH1, RNH1, FLRT2, TNFSF14, ANGPTL4, APBA3, LRRC4C	2.12
GO:0016810	hydrolase activity, acting on carbon-nitrogen (but not peptide) bonds	5	0.06	ACER1, NACC2, SIRT6, ASPA, SIRT3	3.31
GO:0098772	molecular function regulator	22	0.07	RAP1GAP2, PPP1R26, LRRC26, NOTCH1, RNH1, TNFSF14, SH2D3A, TSC1, GTF2F1, VAV1, GALC, RIC8A, C3, FZR1, FLRT2, TRIP10, EPS8L2, ANGPTL4, PHPT1, APBA3, LRRC4C, RALGDS	1.47
GO:0019213	deacetylase activity	3	0.07	NACC2, SIRT6, SIRT3	6.69
GO:0015085	calcium ion transmembrane transporter activity	5	0.08	CACNA1B, ATP2A3, TRPV3, TRPV1, GRIN1	3.07
GO:0046920	alpha-(1->3)-fucosyltransferase activity	2	0.08	FUT5, FUT7	24.08
GO:1901474	azole transmembrane transporter activity	2	0.10	LOC486150, SLC19A3	20.07
GO:0045118	azole transporter activity	2	0.10	LOC486150, SLC19A3	20.07
GO:0090482	vitamin transmembrane transporter activity	2	0.10	LOC486150, SLC19A3	20.07
Cellular component (GO Term)		Count	PValue	Genes	Fold Enrichment
GO:0044441	ciliary part	9	0.02	TTC8, CATSPERD, ODF3, SSNA1, INPP5E, TMEM141, AK8, GLI3, SPATA7	2.73
GO:0097014	ciliary plasm	5	0.02	INPP5E, TMEM141, AK8, GLI3, SPATA7	4.79
GO:0005930	axoneme	5	0.02	INPP5E, TMEM141, AK8, GLI3, SPATA7	4.79
GO:0044463	cell projection part	15	0.03	ODF3, SSNA1, TRPV1, TUBB4A, TMEM141, GLI3, AK8, SPATA7, GRIN1, SETX, TTC8, CATSPERD, INPP5E, EPS8L2, PAFAH1B1	1.90
GO:0032580	Golgi cisterna membrane	3	0.03	FUT5, FUT7, B4GALNT4	10.97

GO:0005929	cilium	11	0.04	TTC8, CATSPERD, ODF3, SSNA1, INPP5E, TUBB4A, TMEM141, AK8, GLI3, SPATA7, PAFAH1B1	2.05
GO:0016363	nuclear matrix	4	0.05	PIAS4, HNRNPM, GFI1B, UHRF1	4.87
GO:0005576	extracellular region	63	0.06	CLIC3, RNH1, DBH, MYDGF, RPL7A, LCNL1, CANF2, C8G, OBP2B, ADAMTSL2, PMPCA, NRTN, PTGDS, UBXN6, ENTPD2, ARRDC1, LOC102153243, EDF1, EML5, PLIN4, TAX1BP3, ANGPTL4, CACTIN, PHPT1, ASPA, PAFAH1B1, LOC491264, FBN3, RALA, PSMD13, SEMA3D, SEMA3A, SEMA3E, HSD17B12, ADAMTS10, C3, GNG10, DPP7, ADAMTS13, FLRT2, LCN15, SAFB2, EPS8L2, LRRC4C, CCL25, LRRC26, PSPN, SPHKAP, CCL20, CD70, CTNS, INHBA, EEF2, TUBB4B, TUBB4A, UBAC1, RAB11B, HNRNPM, LRG1, COL5A1, TRIP10, CDK13, CD320	1.22
GO:0005720	nuclear heterochromatin	3	0.06	KDM4B, UHRF1, SIRT6	7.31
GO:0031985	Golgi cisterna	3	0.09	FUT5, FUT7, B4GALNT4	6.05

Supplementary Table S6. Overall view of functional classification of candidate gene in Bullmastiffs compared across all clades by GO terms and KEGG pathways.

KEGG(Term)		Count	PValue	Genes	Fold Enrichment
cfa00982	Drug metabolism - cytochrome P450	9	0.00	UGT2B31, LOC100856068, FMO1, FMO2, FMO3, UGT2A1, FMO4, UGT1A6, LOC490346	4.50
cfa00040	Pentose and glucuronate interconversions	6	0.00	UGDH, UGT2B31, LOC100856068, RPE, UGT2A1, UGT1A6	6.72
cfa00053	Ascorbate and aldarate metabolism	5	0.01	UGDH, UGT2B31, LOC100856068, UGT2A1, UGT1A6	6.53
cfa01100	Metabolic pathways	65	0.02	PIGS, DGKD, NDUFA11, DBH, ENO1, LOC100856068, LIPC, ACADL, PGM2, MAN1A1, UGT2A1, PTGDS, LOC480667, CERS4, TALDO1, PIPOX, PLA2G4A, CEL, KMO, DCK, GBGT1, APRT, PIGC, UGDH, LOC485024, MVD, ALDOC, LOC611724, ASPA, PAFAH1B1, FH, H6PD, RPE, HSD17B12, LIAS, TYMS, AGPAT2, ABO, AK8, ACAT2, AGPAT4, GK2, FUT5, PTDSS2, FUT7, INPP5E, INPP5K, GCNT3, CKB, PIP5K1C, ADSS, UGT1A6, UGT2B31, NDUFA7, NOS2, LOC476732, PRDX6, GALNS, GALC, ACER1, NMNAT1, CPS1, SARDH, DEGS2, LPIN2	1.29
cfa05222	Small cell lung cancer	9	0.02	RXRA, NOS2, TRAF4, TRAF3, AKT3, COL4A3, PIK3CD, TRAF2, FHIT	2.55
cfa05231	Choline metabolism in cancer	10	0.02	SLC22A3, DGKD, MAP2K2, SLC22A2, AKT3, PLA2G4A, PIK3CD, TSC1, PIP5K1C, RALGDS	2.38
cfa00030	Pentose phosphate pathway	5	0.03	H6PD, RPE, TALDO1, PGM2, ALDOC	4.36
cfa00561	Glycerolipid metabolism	7	0.03	DGKD, LIPC, CEL, LPIN2, AGPAT2, GK2, AGPAT4	2.89
cfa05169	Epstein-Barr virus infection	10	0.04	FCER2, PSMD11, TRAF3, PSMD13, AKT3, ENTPD8, PSMD1, PIK3CD, TRAF2, MAP2K7	2.14
cfa04014	Ras signaling pathway	16	0.04	RALA, MAP2K2, KSR1, PIK3CD, PLA2G4A, FASLG, RASAL2, GRIN1, FGF5, AFDN, GNG10, AKT3, NF1, ABL2, GNB5, RALGDS	1.73
cfa00140	Steroid hormone biosynthesis	6	0.05	UGT2B31, LOC100856068, LOC482182, HSD17B12, UGT2A1, UGT1A6	3.00
cfa04664	Fc epsilon RI signaling pathway	7	0.05	MAP2K2, INPP5D, AKT3, PLA2G4A, PIK3CD, MAP2K7, VAV1	2.57
cfa04062	Chemokine signaling pathway	13	0.05	CCL25, CCL13, ROCK1, CCL20, PIK3CD, VAV1, GNG10, CCL8, CCL7, AKT3, CCL1, GNB5, CCR6	1.81
cfa00230	Purine metabolism	13	0.06	ENTPD2, ENTPD8, FHIT, DCK, AK8, APRT, ADCY10, PDE10A, PDE6D, PGM2, NUDT16, ADSS, LOC611724	1.77
cfa04071	Sphingolipid signaling pathway	10	0.07	ACER1, CERS4, MAP2K2, ROCK1, AKT3, PIK3CD, TRAF2, DEGS2, PPP2R5C, S1PR4	1.94

cfa04152	AMPK signaling pathway	10	0.07	TBC1D1, CAB39, AKT3, PIK3CD, TSC1, PPP2R5C, EEF2, ELAVL1, CAMKK1, RAB11B	1.93
Biological Process (GO Term)		Count	PValue	Genes	Fold Enrichment
GO:0006753	nucleoside phosphate metabolic process	37	0.00	NPFFR2, H6PD, GPR65, RPE, HTR2B, RORA, ENO1, TYMS, FHIT, AK8, NPPC, MPC2, NUDT16, SLC25A23, ADSS, TEFM, SURF1, NDUFA7, ENTPD2, NOS2, LOC102151342, WNT5A, FMO1, CTNS, TALDO1, ENTPD8, FMO2, SIRT6, KMO, DCK, CBFA2T3, NMNAT1, NF1, ALDOC, ACPP, SLC25A33, SMPDL3A	1.97
GO:0055086	nucleobase-containing small molecule metabolic process	39	0.00	NPFFR2, H6PD, GPR65, RPE, HTR2B, RORA, ENO1, TYMS, FHIT, AK8, NPPC, MPC2, NUDT16, SLC25A23, ADSS, TEFM, SURF1, NDUFA7, ENTPD2, NOS2, LOC102151342, WNT5A, FMO1, CTNS, TALDO1, ENTPD8, FMO2, SIRT6, KMO, DCK, CBFA2T3, APRT, NMNAT1, CPS1, NF1, ALDOC, ACPP, SLC25A33, SMPDL3A	1.88
GO:0071346	cellular response to interferon-gamma	10	0.00	CCL25, CCL13, CCL8, NOS2, CCL7, CCL20, WNT5A, DAPK3, CCL1, AQP4	4.65
GO:0019637	organophosphate metabolic process	49	0.00	PIGS, NPFFR2, GPR65, HTR2B, RORA, ENO1, NPPC, MPC2, NUDT16, ENTPD2, LOC102151342, WNT5A, TALDO1, ENTPD8, PLA2G4A, SIRT6, KMO, DCK, CBFA2T3, PIGC, MVD, ALDOC, ACPP, SMPDL3A, H6PD, RPE, PROCA1, TYMS, FHIT, AGPAT2, AK8, GK2, PTDSS2, INPP5D, INPP5E, INPP5K, SLC25A23, ADSS, TEFM, SURF1, NDUFA7, NOS2, FMO1, CTNS, FMO2, NMNAT1, CPS1, NF1, SLC25A33	1.66
GO:0046496	nicotinamide nucleotide metabolic process	12	0.00	H6PD, NMNAT1, MPC2, RPE, FMO1, TALDO1, SIRT6, ALDOC, FMO2, ENO1, KMO, CBFA2T3	3.35
GO:0019362	pyridine nucleotide metabolic process	12	0.00	H6PD, NMNAT1, MPC2, RPE, FMO1, TALDO1, SIRT6, ALDOC, FMO2, ENO1, KMO, CBFA2T3	3.35
GO:0072524	pyridine-containing compound metabolic process	12	0.00	H6PD, NMNAT1, MPC2, RPE, FMO1, TALDO1, SIRT6, ALDOC, FMO2, ENO1, KMO, CBFA2T3	3.26
GO:0048247	lymphocyte chemotaxis	8	0.00	CCL25, CCL13, CCL8, TNFSF14, CCL7, CCL20, WNT5A, CCL1	4.65
GO:0009117	nucleotide metabolic process	33	0.00	NPFFR2, H6PD, GPR65, RPE, HTR2B, RORA, ENO1, TYMS, FHIT, NPPC, MPC2, NUDT16, SLC25A23, ADSS, TEFM, SURF1, NDUFA7, NOS2, LOC102151342, WNT5A, FMO1, CTNS, TALDO1, FMO2, SIRT6, KMO, DCK, CBFA2T3, NMNAT1, NF1, ALDOC, ACPP, SLC25A33	1.80
GO:0044710	single-organism metabolic process	145	0.00	NPFFR2, KLB, GPR65, RORA, ENO1, ALKBH7, ALKBH3, NPPC, LIPC, ATCAY, CDH2, MPC2, NUDT16, PTGDS, LOC480667,	1.26

				ACAD11, MAP3K4, MAP2K2, LOC100688619, ENTPD2, WNT5A, SERPINF2, THOC1, ENTPD8, TALDO1, PIPOX, CYBA, CEL, KMO, GBGT1, SOAT1, DPEP1, H6PD, LIAS, NLK, FHIT, AK8, ACAT2, NEU2, C3, FUT5, PTDSS2, FUT7, NLRP6, LCN15, INPP5D, INPP5E, ST8SIA4, INPP5K, MAP2K7, SURF1, SUCO, MYO5A, TRPV1, ACSF3, QKI, SETX, ACER1, SULT1B1, CPS1, COL5A1, TNFSF4, NF1, CDK10, BLMH, DEGS2, PIGS, LOC607002, ONECUT1, HTR2B, DBH, JCHAIN, MYDGF, ACADL, EXO1, ENOSF1, WDR5, CA6, PGM2, MAN1A1, UGT2A1, SDF2, CERS4, WARS, KSR1, LOC102151342, PLA2G4A, TRAF2, SIRT6, DCK, CBFA2T3, SIRT3, APRT, PIGC, DNM3, TLR1, TRAF4, ASPG, ALDOC, MVD, ACPP, SMPDL3A, PLIN5, PAFAH1B1, CCL13, USP14, FH, CAB39, RPE, PROCA1, TYMS, AGPAT2, GK2, SRR, CCL8, CCL7, ERBB4, CCL1, GC, SLC25A23, ADSS, TEFM, UGT1A6, CCL25, UGT2B31, NDUFA7, KDM4B, NOS2, PNPLA7, CCL20, GPR55, CARD9, CTNS, FMO1, FMO2, FMO3, TPD52L1, SOD2, LOC490346, GALC, NMNAT1, TAOK1, P2RX1, SLC25A33, LPIN2	
GO:0044281	small molecule metabolic process	78	0.00	NPFFR2, GPR65, ONECUT1, HTR2B, RORA, DBH, ENO1, ALKBH7, NPPC, ATCAY, LIPC, ACADL, ENOSF1, MPC2, WDR5, PGM2, CA6, UGT2A1, NUDT16, PTGDS, ACAD11, WARS, ENTPD2, LOC102151342, WNT5A, TALDO1, ENTPD8, PIPOX, PLA2G4A, SIRT6, KMO, DCK, CBFA2T3, APRT, SOAT1, ASPG, DPEP1, MVD, ALDOC, ACPP, SMPDL3A, PLIN5, FH, H6PD, RPE, LIAS, TYMS, FHIT, AK8, ACAT2, GK2, SRR, INPP5E, INPP5K, GC, SLC25A23, ADSS, UGT1A6, TEFM, UGT2B31, SURF1, NDUFA7, NOS2, FMO1, CTNS, MYO5A, FMO2, ACSF3, QKI, ACER1, NMNAT1, CPS1, TNFSF4, NF1, BLMH, DEGS2, LPIN2, SLC25A33	1.41
GO:0008542	visual learning	8	0.00	RIC8A, IFT20, FOXB1, DEAF1, NF1, CTNS, DBH, GRIN1	4.40
GO:0034612	response to tumor necrosis factor	13	0.00	CCL25, PIAS4, CCL13, CCL20, TRAF2, RORA, CCL8, CCL7, TRAF3, INPP5K, CCL1, MAP2K7, CACTIN	2.83
GO:0071356	cellular response to tumor necrosis factor	12	0.00	CCL25, PIAS4, CCL13, CCL8, CCL7, CCL20, TRAF3, INPP5K, CCL1, TRAF2, RORA, CACTIN	2.99
GO:0006733	oxidoreduction coenzyme metabolic process	12	0.00	H6PD, NMNAT1, MPC2, RPE, FMO1, TALDO1, SIRT6, ALDOC, FMO2, ENO1, KMO, CBFA2T3	2.95
GO:0034341	response to interferon-gamma	10	0.00	CCL25, CCL13, CCL8, NOS2, CCL7, CCL20, WNT5A, DAPK3, CCL1, AQP4	3.37
GO:0007632	visual behavior	8	0.00	RIC8A, IFT20, FOXB1, DEAF1, NF1, CTNS, DBH, GRIN1	4.18

GO:0043547	positive regulation of GTPase activity	15	0.00	CCL25, CCL13, ODAM, CCL20, GPR65, WNT5A, TSC1, TBC1D1, ABR, SRR, CCL8, CCL7, TAX1BP3, CCL1, EVI5L	2.43
GO:0043087	regulation of GTPase activity	18	0.00	CCL25, CCL13, ODAM, CCL20, GPR65, WNT5A, TSC1, TBC1D1, ABR, SRR, TTC8, CCL8, CCL7, PDE6D, TAX1BP3, CCL1, EVI5L, PAFAH1B1	2.18
GO:0009812	flavonoid metabolic process	4	0.01	SULT1B1, UGT2B31, UGT2A1, UGT1A6	10.46
GO:0030149	sphingolipid catabolic process	5	0.01	NEU2, GALC, ACER1, CEL, SMPDL3A	6.54
GO:0048854	brain morphogenesis	6	0.01	CDH2, WNT5A, HESX1, NF1, PAFAH1B1, SLC6A4	4.83
GO:0006805	xenobiotic metabolic process	5	0.01	SULT1B1, FMO2, RORA, GRIN1, UGT1A6	6.15
GO:0051492	regulation of stress fiber assembly	8	0.01	TTC8, ROCK1, MYOC, GPR65, SERPINF2, INPP5K, EVL, TSC1	3.42
GO:0031345	negative regulation of cell projection organization	12	0.01	RAP1GAP2, SEMA6B, BAG5, SEMA3D, SEMA3A, WNT5A, SEZ6, EVL, SEMA3E, EVI5L, CDK5R1, ITM2C	2.49
GO:0006796	phosphate-containing compound metabolic process	109	0.01	NPFFR2, KLB, CCNK, GPR65, RORA, ENO1, NPPC, CDH2, MPC2, RPS6KA2, AKT3, NUDT16, MAP3K4, MAP2K2, ENTPD2, DAPK3, WNT5A, SERPINF2, MATK, ENTPD8, TALDO1, TSC1, KMO, WDR81, PHPT1, H6PD, NLK, FHIT, AK8, C3, PTDSS2, VTN, FLRT2, NLRP6, INPP5D, INPP5E, ABL2, INPP5K, LRRC4C, MAP2K7, PPP1R26, SURF1, YES1, INHBA, CAMSAP3, MOB1B, SETX, PKIB, CPS1, NF1, UNC119, CDK10, CDK13, PIGS, HTR2B, MYDGF, GSG2, MYOC, KSR1, LOC102151342, PLA2G4A, TRAF2, SIRT6, DCK, CBFA2T3, PIGC, TLR1, TRAF4, RTN4RL1, ALDOC, MVD, ACPP, CACTIN, SMPDL3A, PAFAH1B1, CCL13, ROCK1, CAB39, RPE, PRR5L, PROCA1, MLLT1, TYMS, AGPAT2, GK2, CCL8, CCL7, ERBB4, CCL1, SLC25A23, ADSS, TEFM, CCL25, NDUFA7, NOS2, CCL20, ODAM, GPR55, CARD9, CTNS, FMO1, FMO2, TPD52L1, MYO1D, NMNAT1, TAOK1, SLC25A33, NEK11, CDK5R1	1.25
GO:0046466	membrane lipid catabolic process	5	0.01	NEU2, GALC, ACER1, CEL, SMPDL3A	5.81
GO:0003018	vascular process in circulatory system	12	0.01	NPPC, ABR, NOS2, CPS1, TBXA2R, P2RX1, SERPINF2, HTR2B, DBH, SOD2, ASIC2, LOC479600	2.46
GO:0006793	phosphorus metabolic process	109	0.01	NPFFR2, KLB, CCNK, GPR65, RORA, ENO1, NPPC, CDH2, MPC2, RPS6KA2, AKT3, NUDT16, MAP3K4, MAP2K2, ENTPD2, DAPK3, WNT5A, SERPINF2, MATK, ENTPD8, TALDO1, TSC1, KMO, WDR81, PHPT1, H6PD, NLK, FHIT, AK8, C3, PTDSS2, VTN, FLRT2, NLRP6, INPP5D, INPP5E, ABL2, INPP5K, LRRC4C, MAP2K7, PPP1R26, SURF1, YES1, INHBA, CAMSAP3, MOB1B, SETX, PKIB, CPS1, NF1, UNC119, CDK10, CDK13, PIGS, HTR2B, MYDGF, GSG2,	1.25

				MYOC, KSR1, LOC102151342, PLA2G4A, TRAF2, SIRT6, DCK, CBFA2T3, PIGC, TLR1, TRAF4, RTN4RL1, ALDOC, MVD, ACPP, CACTIN, SMPDL3A, PAFAH1B1, CCL13, ROCK1, CAB39, RPE, PRR5L, PROCA1, MLLT1, TYMS, AGPAT2, GK2, CCL8, CCL7, ERBB4, CCL1, SLC25A23, ADSS, TEFM, CCL25, NDUFA7, NOS2, CCL20, ODAM, GPR55, CARD9, CTNS, FMO1, FMO2, TPD52L1, MYO1D, NMNAT1, TAOK1, SLC25A33, NEK11, CDK5R1	
GO:0042476	odontogenesis	10	0.01	ODAM, ENAM, NFIC, ANKRD11, AMBN, AMTN, MYO5A, WDR72, INHBA, GLI3	2.75
GO:0016477	cell migration	56	0.01	BARHL1, RERE, ONECUT1, HTR2B, PIK3CD, TNFAIP1, DBH, GLI3, PTPRG, GJA1, CDH2, MYO18A, NRTN, SEMA6B, MYOC, DAPK3, WNT5A, MATK, VAV1, RUFY3, DPEP1, EVL, SDCCAG8, PAFAH1B1, ASTN1, CCL13, NOTCH1, ROCK1, SEMA3D, SEMA3A, PLG, PRR5L, SEMA3E, RIC8A, VTN, ABR, TMEM201, FUT7, CCL8, CCL7, T, FLRT2, ERBB4, ABL2, CCL1, CCL25, FOXB1, TNFSF14, CCL20, CDC42BPB, FOXN1, MYO1C, COL5A1, NF1, FGFR1OP, CDK5R1	1.39
GO:0071466	cellular response to xenobiotic stimulus	5	0.01	SULT1B1, FMO2, RORA, GRIN1, UGT1A6	5.50
GO:0072676	lymphocyte migration	8	0.01	CCL25, CCL13, CCL8, TNFSF14, CCL7, CCL20, WNT5A, CCL1	3.22
GO:0006928	movement of cell or subcellular component	75	0.01	BARHL1, RERE, ONECUT1, HTR2B, PIK3CD, TNFAIP1, DBH, TMEM141, GLI3, PTPRG, GJA1, CDH2, MYO18A, NRTN, KIF1B, SEMA6B, MAP2K2, MYOC, DAPK3, WNT5A, MATK, VAV1, WDR81, CATSPERD, RUFY3, DPEP1, EVL, PHPT1, GAS8, SDCCAG8, PAFAH1B1, ASTN1, CCL13, NOTCH1, ROCK1, SEMA3D, SSNA1, SEMA3A, PLG, PRR5L, SEMA3E, SPG7, RIC8A, VTN, ABR, TMEM201, FUT7, CCL8, RHOT1, CCL7, T, FLRT2, ERBB4, ABL2, CCL1, CCL25, DYNC1H1, FOXB1, DNAH12, TNFSF14, CCL20, WDR19, MYO5A, CDC42BPB, FOXN1, IFT20, ADCY10, TTC8, MYO1C, COL5A1, LHX3, KIF26B, NF1, FGFR1OP, CDK5R1	1.31
GO:0046514	ceramide catabolic process	4	0.01	NEU2, GALC, ACER1, CEL	7.61
GO:0031349	positive regulation of defense response	17	0.01	COLEC12, CCL13, CARD9, WNT5A, CYBA, TICAM1, VAV1, TLR1, C3, CCL8, NLRP6, CCL7, TRAF3, TNFSF4, CCL1, TLR10, CACTIN	1.95
GO:0040011	locomotion	67	0.01	BARHL1, RERE, ONECUT1, HTR2B, PIK3CD, TNFAIP1, DBH, GLI3, PTPRG, GJA1, CDH2, MYO18A, NRTN, CCR6, SEMA6B, MAP2K2, MYOC, DAPK3, WNT5A, MATK, VAV1, CATSPERD, RUFY3, DPEP1, EVL, PHPT1, GAS8, SDCCAG8, PAFAH1B1, ASTN1, CCL13, USP14, NOTCH1, ROCK1, SEMA3D, SEMA3A, PLG, PRR5L,	

				SEMA3E, RIC8A, VTN, ABR, TMEM201, FUT7, CCL8, CCL7, T, FLRT2, ERBB4, ABL2, CCL1, CCL25, FOXB1, TNFSF14, CCL20, MYO5A, CDC42BPB, FOXN1, TTC8, MYO1C, COL5A1, LHX3, KIF26B, ACKR4, NF1, FGFR1OP, CDK5R1	
GO:0044242	cellular lipid catabolic process	13	0.01	PLA2G4A, CEL, ACAT2, GALC, NEU2, ACER1, LIPC, ACADL, CPS1, ACAD11, SMPDL3A, LPIN2, PLIN5	2.21
GO:0008306	associative learning	8	0.02	RIC8A, IFT20, FOXB1, DEAF1, NF1, CTNS, DBH, GRIN1	3.04
GO:0017144	drug metabolic process	4	0.02	FMO1, DPEP1, FMO2, FMO4	6.97
GO:0048870	cell motility	60	0.02	BARHL1, RERE, ONECUT1, HTR2B, PIK3CD, TNFAIP1, DBH, GLI3, PTPRG, GJA1, CDH2, MYO18A, NRTN, SEMA6B, MAP2K2, MYOC, DAPK3, WNT5A, MATK, VAV1, CATSPERD, RUFY3, DPEP1, EVL, PHPT1, GAS8, SDCCAG8, PAFAH1B1, ASTN1, CCL13, NOTCH1, ROCK1, SEMA3D, SEMA3A, PLG, PRR5L, SEMA3E, RIC8A, VTN, ABR, TMEM201, FUT7, CCL8, CCL7, T, FLRT2, ERBB4, ABL2, CCL1, CCL25, FOXB1, TNFSF14, CCL20, CDC42BPB, FOXN1, MYO1C, COL5A1, NF1, FGFR1OP, CDK5R1	1.34
GO:0051674	localization of cell	60	0.02	BARHL1, RERE, ONECUT1, HTR2B, PIK3CD, TNFAIP1, DBH, GLI3, PTPRG, GJA1, CDH2, MYO18A, NRTN, SEMA6B, MAP2K2, MYOC, DAPK3, WNT5A, MATK, VAV1, CATSPERD, RUFY3, DPEP1, EVL, PHPT1, GAS8, SDCCAG8, PAFAH1B1, ASTN1, CCL13, NOTCH1, ROCK1, SEMA3D, SEMA3A, PLG, PRR5L, SEMA3E, RIC8A, VTN, ABR, TMEM201, FUT7, CCL8, CCL7, T, FLRT2, ERBB4, ABL2, CCL1, CCL25, FOXB1, TNFSF14, CCL20, CDC42BPB, FOXN1, MYO1C, COL5A1, NF1, FGFR1OP, CDK5R1	1.34
GO:0042475	odontogenesis of dentin-containing tooth	8	0.02	ODAM, ENAM, NFIC, ANKRD11, AMBN, AMTN, WDR72, GLI3	2.94
GO:0006739	NADP metabolic process	5	0.02	H6PD, RPE, FMO1, TALDO1, FMO2	4.75
GO:0009268	response to pH	5	0.02	ACER1, GPR65, TRPV1, ASIC2, RAB11B	4.75
GO:0032231	regulation of actin filament bundle assembly	8	0.02	TTC8, ROCK1, MYOC, GPR65, SERPINF2, INPP5K, EVL, TSC1	2.89
GO:0071347	cellular response to interleukin-1	8	0.02	CCL25, CCL13, CCL8, CCL7, CCL20, CCL1, RORA, CACTIN	2.89
GO:0021915	neural tube development	13	0.02	FOXB1, RALA, NOTCH1, WDR19, WNT5A, TSC1, LIAS, GLI3, MIB1, T, TBC1D32, DEAF1, NF1	2.11
GO:0072531	pyrimidine-containing compound transmembrane transport	3	0.02	LOC486150, SLC19A3, SLC25A33	12.55
GO:0044712	single-organism catabolic process	34	0.02	USP14, LOC607002, RPE, ENO1, ACAT2, NEU2, LIPC, ACADL, ENOSF1, INPP5E, INPP5K, NUDT16, SDF2, LOC480667, ACAD11,	1.49

				ENTPD2, NOS2, PIPOX, PLA2G4A, SIRT6, CEL, KMO, ACSF3, CBFA2T3, GALC, ACER1, CPS1, ASPG, ALDOC, BLMH, LPIN2, SMPDL3A, PAFAH1B1, PLIN5	
GO:0050684	regulation of mRNA processing	9	0.02	WTAP, SLTM, YTHDC1, LMNTD2, SAFB2, SAFB, NSRP1, SUPT6H, ZC3H14	2.61
GO:0010977	negative regulation of neuron projection development	10	0.02	RAP1GAP2, SEMA6B, BAG5, SEMA3D, SEMA3A, WNT5A, SEZ6, SEMA3E, CDK5R1, ITM2C	2.43
GO:0030517	negative regulation of axon extension	6	0.02	SEMA6B, SEMA3D, SEMA3A, WNT5A, SEMA3E, CDK5R1	3.69
GO:0019693	ribose phosphate metabolic process	24	0.02	NPFFR2, SURF1, NDUFA7, H6PD, NOS2, GPR65, RPE, LOC102151342, WNT5A, HTR2B, TALDO1, CTNS, SIRT6, RORA, ENO1, CBFA2T3, NPPC, NF1, ALDOC, NUDT16, SLC25A23, ADSS, SLC25A33, TEFM	1.63
GO:0006955	immune response	49	0.02	AQP4, FASLG, RORA, TNFAIP1, JCHAIN, EXO1, CCR6, RAG1, DAPK3, WNT5A, MATK, THOC1, TRAF2, TSC1, CYBA, TICAM1, VAV1, TLR1, FCER2, TRAF3, TLR10, CACTIN, PHPT1, CLEC4G, COLEC12, CCL13, NOTCH1, STXBP2, SUPT6H, C3, VTN, ABR, FUT7, CCL8, CCL7, NLRP6, INPP5D, GCNT3, ABL2, CCL1, CCL25, YES1, TNFSF14, NOS2, CCL20, CD70, CARD9, TNFSF4, TNFSF9	1.36
GO:0006672	ceramide metabolic process	8	0.03	NEU2, GALC, ACER1, CERS4, P2RX1, ST8SIA4, DEGS2, CEL	2.74
GO:0001938	positive regulation of endothelial cell proliferation	7	0.03	MYDGF, EGFL7, LRG1, WNT5A, NF1, HTR2B, NRARP	3.05
GO:0050880	regulation of blood vessel size	10	0.03	NPPC, NOS2, CPS1, TBXA2R, P2RX1, SERPINF2, HTR2B, DBH, SOD2, ASIC2	2.35
GO:0035150	regulation of tube size	10	0.03	NPPC, NOS2, CPS1, TBXA2R, P2RX1, SERPINF2, HTR2B, DBH, SOD2, ASIC2	2.32
GO:0016064	immunoglobulin mediated immune response	8	0.03	C3, FCER2, EXO1, TNFSF4, INPP5D, THOC1, GCNT3, SUPT6H	2.70
GO:0010970	establishment of localization by movement along microtubule	9	0.03	IFT20, TMEM201, RHOT1, WDR81, SSNA1, WDR19, KIF1B, SPG7, PAFAH1B1	2.48
GO:0030010	establishment of cell polarity	9	0.03	WDR81, SPAG5, KIF26B, WNT5A, MYO18A, MARK3, NDC80, SDCCAG8, PAFAH1B1	2.48
GO:0002250	adaptive immune response	15	0.03	THOC1, TRAF2, TSC1, RORA, SUPT6H, JCHAIN, TLR1, C3, FCER2, EXO1, TNFSF4, INPP5D, GCNT3, CLEC4G, RAG1	1.88
GO:0050768	negative regulation of neurogenesis	15	0.03	RAP1GAP2, SEMA6B, NOTCH1, SEMA3D, SEMA3A, WNT5A, SEZ6, SEMA3E, GLI3, MIB1, BAG5, ERBB4, NF1, ITM2C, CDK5R1	1.88
GO:0006732	coenzyme metabolic process	16	0.03	H6PD, RPE, TALDO1, PIPOX, FMO1, FMO2, SIRT6, ENO1, LIAS, TYMS, KMO, CBFA2T3, NMNAT1, MPC2, ALDOC, MVD	1.83

GO:0048843	negative regulation of axon extension involved in axon guidance	5	0.03	SEMA6B, SEMA3D, SEMA3A, WNT5A, SEMA3E	4.18
GO:0045665	negative regulation of neuron differentiation	13	0.03	RAP1GAP2, SEMA6B, NOTCH1, SEMA3D, SEMA3A, WNT5A, SEZ6, SEMA3E, GLI3, MIB1, BAG5, ITM2C, CDK5R1	2.00
GO:0002224	toll-like receptor signaling pathway	8	0.03	TLR1, COLEC12, NLRP6, TRAF3, CYBA, TLR10, TICAM1, CACTIN	2.66
GO:0019724	B cell mediated immunity	8	0.03	C3, FCER2, EXO1, TNFSF4, INPP5D, THOC1, GCNT3, SUPT6H	2.66
GO:0006954	inflammatory response	25	0.03	CCL13, NOTCH1, RORA, LIAS, C3, ABR, CCL8, NLRP6, CCL7, TBXA2R, CCL1, CCL25, NOS2, CCL20, ODAM, WNT5A, CYBA, TRPV1, LOC479600, TLR1, SIGIRR, TNFSF4, P2RX1, TLR10, IL17C	1.56
GO:2000241	regulation of reproductive process	10	0.03	NPPC, FZR1, NOTCH1, RXRA, SERPINF1, RPS6KA2, SEMA3A, WNT5A, INHBA, PTGDS	2.27
GO:0010810	regulation of cell-substrate adhesion	12	0.03	CCL25, SMOC2, VTN, NOTCH1, ROCK1, MYOC, ONECUT1, DAPK3, NF1, TSC1, SEMA3E, HSD17B12	2.06
GO:0010447	response to acidic pH	4	0.03	GPR65, TRPV1, ASIC2, RAB11B	5.58
GO:0009410	response to xenobiotic stimulus	5	0.03	SULT1B1, FMO2, RORA, GRIN1, UGT1A6	4.02
GO:1902668	negative regulation of axon guidance	5	0.03	SEMA6B, SEMA3D, SEMA3A, WNT5A, SEMA3E	4.02
GO:0001755	neural crest cell migration	7	0.03	SEMA6B, ERBB4, SEMA3D, SEMA3A, HTR2B, SEMA3E, NRTN	2.87
GO:0050729	positive regulation of inflammatory response	8	0.03	C3, CCL13, CCL8, CCL7, TNFSF4, WNT5A, CCL1, TLR10	2.57
GO:0006643	membrane lipid metabolic process	12	0.03	NEU2, PIGS, GALC, PIGC, ACER1, CERS4, P2RX1, ST8SIA4, DEGS2, CEL, SMPDL3A, GBGT1	2.02
GO:0044282	small molecule catabolic process	15	0.04	NOS2, RPE, PIPOX, KMO, ACSF3, ACAT2, ACADL, ENOSF1, ASPG, INPP5E, INPP5K, BLMH, ACAD11, LPIN2, PLIN5	1.82
GO:0021987	cerebral cortex development	9	0.04	CCDC85C, CDH2, SEMA3A, NF1, TSC1, GLI3, GRIN1, CDK5R1, PAFAH1B1	2.35
GO:0006278	RNA-dependent DNA biosynthetic process	6	0.04	PKIB, TCP1, HNRNPU, MAP2K7, MAP3K4, SMG6	3.22
GO:0007004	telomere maintenance via telomerase	6	0.04	PKIB, TCP1, HNRNPU, MAP2K7, MAP3K4, SMG6	3.22
GO:0001952	regulation of cell-matrix adhesion	8	0.04	CCL25, ROCK1, MYOC, ONECUT1, DAPK3, NF1, TSC1, SEMA3E	2.54
GO:0061572	actin filament bundle organization	10	0.04	TTC8, ROCK1, MYOC, GPR65, SERPINF2, RFLNB, INPP5K, EVL, TSC1, TNFAIP1	2.20
GO:0016054	organic acid catabolic process	12	0.04	NOS2, ACADL, ENOSF1, ASPG, PIPOX, BLMH, KMO, ACAD11, LPIN2, ACSF3, ACAT2, PLIN5	1.99

GO:0072358	cardiovascular system development	44	0.04	RNH1, NOTCH1, ROCK1, NXN, HTR2B, HNRNPU, PLG, FASLG, SEMA3E, NRARP, RORA, GLI3, RIC8A, C3, MYDGF, GJA1, RXRA, ADAP2, T, FLRT2, CDH2, TBC1D32, ERBB4, RPS6KA2, MAP2K2, WARS, SERPINF1, SERPINF2, WNT5A, DAW1, UBE4B, TSC1, SOD2, FOXN1, QKI, MIB1, IFT20, MYO1E, LRG1, COL5A1, NCL, COL4A3, NF1, ANGPTL4	1.34
GO:0072359	circulatory system development	44	0.04	RNH1, NOTCH1, ROCK1, NXN, HTR2B, HNRNPU, PLG, FASLG, SEMA3E, NRARP, RORA, GLI3, RIC8A, C3, MYDGF, GJA1, RXRA, ADAP2, T, FLRT2, CDH2, TBC1D32, ERBB4, RPS6KA2, MAP2K2, WARS, SERPINF1, SERPINF2, WNT5A, DAW1, UBE4B, TSC1, SOD2, FOXN1, QKI, MIB1, IFT20, MYO1E, LRG1, COL5A1, NCL, COL4A3, NF1, ANGPTL4	1.34
GO:0009813	flavonoid biosynthetic process	3	0.04	UGT2B31, UGT2A1, UGT1A6	8.97
GO:0052696	flavonoid glucuronidation	3	0.04	UGT2B31, UGT2A1, UGT1A6	8.97
GO:0021578	hindbrain maturation	3	0.04	RERE, GRIN1, CDK5R1	8.97
GO:0021626	central nervous system maturation	3	0.04	RERE, GRIN1, CDK5R1	8.97
GO:0010721	negative regulation of cell development	17	0.04	RAP1GAP2, SEMA6B, NOTCH1, SEMA3D, SEMA3A, WNT5A, SEZ6, RFLNB, SEMA3E, GLI3, MIB1, NPPC, BAG5, ERBB4, NF1, ITM2C, CDK5R1	1.71
GO:0016525	negative regulation of angiogenesis	7	0.04	NOTCH1, ROCK1, SERPINF1, COL4A3, NF1, SEMA3E, FASLG	2.71
GO:0002548	monocyte chemotaxis	6	0.04	CCL25, CCL13, CCL8, CCL7, CCL20, CCL1	3.06
GO:1904353	regulation of telomere capping	4	0.04	PKIB, MAP2K7, MAP3K4, SMG6	4.92
GO:0019221	cytokine-mediated signaling pathway	17	0.05	CCL25, PIAS4, CCL13, CCL20, WNT5A, TRAF2, SIGIRR, GREM2, CCL8, FLRT2, CCL7, TRAF3, RTN4RL1, CCL1, CACTIN, LRRC4C, PAFAH1B1	1.69
GO:0030705	cytoskeleton-dependent intracellular transport	8	0.05	TMEM201, RHOT1, WDR81, KIF26B, MYO5A, KIF1B, SPG7, PAFAH1B1	2.43
GO:0021537	telencephalon development	14	0.05	FOXB1, SEMA3A, WNT5A, TSC1, INHBA, GLI3, GRIN1, TTC8, CCDC85C, CDH2, ERBB4, NF1, CDK5R1, PAFAH1B1	1.80
GO:0070555	response to interleukin-1	8	0.05	CCL25, CCL13, CCL8, CCL7, CCL20, CCL1, RORA, CACTIN	2.39
GO:2000181	negative regulation of blood vessel morphogenesis	7	0.05	NOTCH1, ROCK1, SERPINF1, COL4A3, NF1, SEMA3E, FASLG	2.61
GO:0044255	cellular lipid metabolic process	37	0.05	PIGS, HTR2B, PROCA1, LIAS, ALKBH7, AGPAT2, ACAT2, GK2, NEU2, C3, PTDSS2, LIPC, ACADL, INPP5D, INPP5E, ST8SIA4, INPP5K, PTGDS, ACAD11, CERS4, MYO5A, PLA2G4A, CEL, ACSF3,	1.36

				QKI, GBGT1, GALC, PIGC, ACER1, SOAT1, CPS1, P2RX1, MVD, DEGS2, LPIN2, SMPDL3A, PLIN5	
GO:0051336	regulation of hydrolase activity	34	0.05	CCL13, ROCK1, GPR65, HTR2B, FASLG, VTN, ABR, SRR, CCL8, CCL7, PDE6D, ABL2, CCL1, EVI5L, RAG1, PPP1R26, CCL25, CCL20, ODAM, GPR55, WNT5A, TSC1, CAMSAP3, MYO1D, TBC1D1, TTC8, WDR81, P2RX1, COL4A3, DPEP1, TAX1BP3, ANGPTL4, PAFAH1B1, PLIN5	1.38
GO:0045087	innate immune response	24	0.05	COLEC12, CCL25, CCL13, YES1, NOS2, CCL20, CARD9, DAPK3, WNT5A, MATK, CYBA, AQP4, TICAM1, VAV1, JCHAIN, TLR1, CCL8, NLRP6, CCL7, TRAF3, ABL2, CCL1, TLR10, CACTIN	1.50
GO:0007097	nuclear migration	3	0.05	TMEM201, WDR81, PAFAH1B1	7.84
GO:0045117	azole transport	3	0.05	SLC22A3, LOC486150, SLC19A3	7.84
GO:0052695	cellular glucuronidation	3	0.05	UGT2B31, UGT2A1, UGT1A6	7.84
GO:0010833	telomere maintenance via telomere lengthening	6	0.05	PKIB, TCP1, HNRNPU, MAP2K7, MAP3K4, SMG6	2.92
GO:0051293	establishment of spindle localization	5	0.05	DYNC1H1, WDR81, SPAG5, NDC80, PAFAH1B1	3.49
GO:0019748	secondary metabolic process	5	0.05	WNT5A, FMO1, MYO5A, FMO2, KMO	3.49
GO:0046068	cGMP metabolic process	5	0.05	NPPC, NOS2, WNT5A, HTR2B, RORA	3.49
GO:0072521	purine-containing compound metabolic process	24	0.05	NPFFR2, SURF1, NDUFA7, NOS2, GPR65, LOC102151342, WNT5A, HTR2B, CTNS, SIRT6, RORA, ENO1, FHIT, CBFA2T3, APRT, NPPC, NF1, ALDOC, NUDT16, SLC25A23, ACPP, ADSS, SLC25A33, TEFM	1.49
GO:0051961	negative regulation of nervous system development	15	0.05	RAP1GAP2, SEMA6B, NOTCH1, SEMA3D, SEMA3A, WNT5A, SEZ6, SEMA3E, GLI3, MIB1, BAG5, ERBB4, NF1, ITM2C, CDK5R1	1.72
GO:0006082	organic acid metabolic process	36	0.06	FH, ENO1, LIAS, ALKBH7, ACAT2, SRR, LIPC, ATCAY, ACADL, MPC2, ENOSF1, UGT2A1, PTGDS, ACAD11, UGT1A6, UGT2B31, WARS, NOS2, FMO1, PIPOX, MYO5A, PLA2G4A, FMO2, SIRT6, KMO, ACSF3, CBFA2T3, QKI, NMNAT1, CPS1, ASPG, DPEP1, ALDOC, BLMH, LPIN2, PLIN5	1.35
GO:0070098	chemokine-mediated signaling pathway	6	0.06	CCL25, CCL13, CCL8, CCL7, CCL20, CCL1	2.85
GO:1901564	organonitrogen compound metabolic process	78	0.06	NPFFR2, GPR65, HTR2B, RORA, DBH, ENO1, RPL9, ELAVL1, SLC9C2, MRPL41, RPL7A, NPPC, MRPL3, YAE1D1, ATCAY, ACADL, ENOSF1, MPC2, NUDT16, SLC25A47, LOC102157323, CERS4, WARS, LOC102151342, DAPK3, WNT5A, TALDO1, PIPOX, SIRT6, TSC1, CEL, KMO, DCK, CBFA2T3, APRT, UGDH, ASPG, DPEP1, ALDOC, ACPP, SMPDL3A, H6PD, RPE, MRPL18, TYMS,	1.21

				FHIT, GIGYF2, NEU2, PTDSS2, SRR, SLC25A29, RXRA, ADAMTS13, ST8SIA4, RPL13, SLC25A23, ADSS, TEFM, SURF1, NDUFA7, NOS2, FMO1, CTNS, FMO2, EEF2, SOD2, QKI, GALC, ACER1, SULT1B1, NMNAT1, CPS1, P2RX1, CPD, NF1, BLMH, DEGS2, SLC25A33	
GO:0090407	organophosphate biosynthetic process	21	0.06	PIGS, NPFFR2, SURF1, NOS2, GPR65, LOC102151342, HTR2B, ENTPD8, TYMS, KMO, AGPAT2, AK8, GK2, PIGC, PTDSS2, NPPC, NMNAT1, CPS1, NF1, MVD, ADSS	1.53
GO:0046209	nitric oxide metabolic process	5	0.06	NOS2, CPS1, RORA, SOD2, TICAM1	3.37
GO:0007595	lactation	4	0.06	FOXB1, ERBB4, CSN3, CSN2	4.40
GO:0060249	anatomical structure homeostasis	18	0.06	FH, ANKRD11, XRCC3, GPR55, RPA1, HNRNPU, ILDR2, PLG, GIGYF2, SPATA7, SMG6, JCHAIN, PKIB, OBP2B, INPP5D, TCP1, MAP2K7, MAP3K4	1.60
GO:0071345	cellular response to cytokine stimulus	22	0.06	CCL25, PIAS4, CCL13, NOS2, CCL20, DAPK3, WNT5A, TRAF2, AQP4, RORA, SIGIRR, GREM2, CCL8, FLRT2, CCL7, TRAF3, RTN4RL1, INPP5K, CCL1, CACTIN, LRRC4C, PAFAH1B1	1.50
GO:0044699	single-organism process	465	0.06	RNH1, EHMT1, HNRNPU, ENO1, ALKBH7, SLC4A4, ALKBH3, GJA1, LIPC, AKT3, TMEM8C, LOC480667, RNF111, UNC13C, WSB1, SERPINF1, DAPK3, SERPINF2, CACNA2D3, RFX2, WDR72, HDGFRP2, CEL, EML1, LOC491216, SIGIRR, WDR81, RUFY3, COR1R4, NGEF, RABL6, ASTN1, MRPL18, GIGYF2, C3, RIC8A, ABR, ADAMTS13, RHOT1, T, FLRT2, HESX1, SLC19A3, DNAH12, ABCA2, PRRC2C, PRDX6, SETX, SULT1B1, TNFSF4, CDK10, TNFSF9, UNC119, CDK13, COR1P2, PIGS, MYOM1, SETD3, GLI3, SLC6A4, ADAMTSL2, DLGAP1, APPL1, SS18, CLUL1, MYOC, TIMM22, SIRT6, MED27, SIRT3, PIGC, TCP1, PLIN4, TLR10, ANGPTL4, VAMP4, CACTIN, GAS8, ANGPTL1, PAFAH1B1, PLIN5, CCL13, FH, LOC486150, SEZ6, PROCA1, CHRND, CHRNG, GPA33, GCNT3, CKB, GC, GPR157, CCL25, UTP3, CCL20, ODAM, CARD9, GPR55, ECEL1, WDR19, RFLNB, FMO1, EMR4, FMO2, FMO3, DEF8, TUBB4B, LOC479600, TUBB4A, SMOC2, XAB2, SPSB1, LRG1, NMNAT1, TAOK1, NFIC, SARM1, CRLF3, DGKD, GPR65, RORA, SLC9C2, FGF5, YY1, ATCAY, CDH2, MPC2, MAEL, NUDT16, MAP3K4, SLC25A47, SEMA6B, PIAS4, LOC100688619, MAP2K2, FBXW5, THOC1, PIPOX, TICAM1, HIC1, NCL, RAB34, PKP3, PHPT1, CLEC4G, NOTCH1, ANKRD11, ILDR2, RASAL2, FHIT, SPATA7, AK8, ACAT2, VTN, BAG5, ADAP2, TBXA2R, NACC2, SAFB2, ABL2, LRRC4C, MAP2K7, SEC16B, LOC611352, API5, SEC16A, AMBN, FANCA, EEF2, FOXN1, QKI, NDC80, MOB1B, ADCY10, LHX3, NF1,	1.04

FGFR1OP, PPP1R13B, ONECUT1, HTR2B, PIK3CD, FASLG, NRARP, ITGAE, EXO1, TAGAP, WDR5, HMG20B, PGM2, UGT2A1, EVI5L, DIS3L2, CERS4, KSR1, SH2D3A, LOC102151342, LANCL1, TRAF2, TRAF4, TRAF3, CATSPERD, RTN4RL1, ASPG, SPACA3, EVL, ACPP, SMPDL3A, ROCK1, UHRF1, SEMA3D, SEMA3A, RPE, BECN2, SEMA3E, PLG, PRR5L, HSD17B12, SRR, FZR1, CCL8, NCLN, CCDC85C, CCL7, CCL1, SLC25A23, RILP, ADSS, UGT1A6, UGT2B31, NDUFA7, RPH3AL, SPAG5, LOC102151683, KCNJ13, CTNS, CDC42BPB, TPD52L1, LOC490346, MYO1E, MYO1C, P2RX1, VPS41, CCDC66, SLC25A33, NPFFR2, CCNK, SLC46A1, LOC611660, CSN3, AQP4, CSN2, COR1A3, SNRPD1, RPS6KA2, PIEZO1, OR3A10, ACAD11, PTGDS, ARRDC5, ENTPD2, ESCO1, KCNK10, OMG, TALDO1, ENTPD8, UNC93A, GBGT1, SOAT1, SLC22A3, SLC45A1, SLC22A2, STXBP2, EBI3, NLK, LIAS, NEU2, PTDSS2, GNG10, MAP2, DPH1, LCN15, INPP5D, INPP5E, ST8SIA4, INPP5K, RALGDS, RAB11FIP4, CDT1, SURF1, YES1, SPHKAP, TNFSF14, XRCC3, MYO5A, SUCO, ZWINT, ACER1, CORO6, ITM2C, BARHL1, CLIC3, LOC607002, DOC2B, JCHAIN, PTPRG, MYDGF, ACADL, MAP1LC3C, GSG2, CHMP1A, DNER, KIF1B, QSOX2, SDF2, GIT1, RALGPS2, WARS, ANO9, CBFA2T3, VAV1, APRT, TLR1, UGDH, ENAM, ARHGEF3, ALDOC, TAX1BP3, SDCCAG8, SDCCAG3, CAB39, SSNA1, TYMS, MNT, RXRA, MPZL1, TEFM, SUZ12, DYNC1H1, FOXB1, PNPLA7, CD70, MIB1, RAB11B, GRIN1, TTC8, GFI1B, KIF26B, TRIP10, TRIP12, APBA3, IL17C, NEK11, CDK5R1, KLB, LOC489640, TTF1, TMEM141, NPPC, TBC1D32, NRTN, RAG2, RAG1, RGS7, WNT5A, MATK, CYBA, UBE4B, TSC1, KMO, PRSS56, COL4A3, DPEP1, H6PD, PSMD11, PSMD13, UBA5, CACNA1B, SUPT6H, CAMSAP1, FUT5, BRINP2, FUT7, NLRP6, SOHLH1, DEAF1, ASIC2, MARK3, RAP1GAP2, TDRD9, TRPV3, DNAJC13, TIMM44, TRPV1, INHBA, SSH2, TDRD5, ACSF3, IFT20, CAMSAP3, CPS1, COL5A1, TAF4B, BLMH, DEGS2, GNB5, GPSM1, RERE, TNFAIP2, ATP2A3, DBH, TNFAIP1, ADGRE1, RASGEF1B, OBP2B, ENOSF1, SNAPC4, DACT2, MYO18A, CA6, MAN1A1, TOPBP1, EGFL7, ARAP2, PLA2G4A, PPP2R5C, SAFB, DCK, TRDN, DNM3, GREM2, DNLZ, SAG, AMTN, MVD, PAPPA2, COLEC12, DTHD1, USP14, RALA, POLDIP2, OR1E2, NXN, GOSR1, ASB14, SPG7, AGPAT2, GK2, TMEM201, GNA15, TMEM203, ERBB4, EPS8L2, S1PR4, KDM4B, NOS2, DAW1, LOC482182, SOD2, TBX19, DLK1, TTC17, GALC, PDE10A, LPIN2

GO:0023014	signal transduction by protein phosphorylation	32	0.06	KLB, CCL13, CAB39, HTR2B, NLK, MYDGF, CCL8, NLRP6, CCL7, CDH2, ERBB4, INPP5K, CCL1, MAP2K7, MAP3K4, CCL25, MAP2K2, KSR1, CCL20, GPR55, SERPINF2, CARD9, WNT5A, TRAF2, TPD52L1, TLR1, SETX, TRAF4, TAOK1, NF1, CDK10, PAFAH1B1	1.37
GO:0090630	activation of GTPase activity	7	0.06	TBC1D1, SRR, GPR65, WNT5A, TSC1, TAX1BP3, EVI5L	2.48
GO:1901343	negative regulation of vasculature development	7	0.06	NOTCH1, ROCK1, SERPINF1, COL4A3, NF1, SEMA3E, FASLG	2.48
GO:0045765	regulation of angiogenesis	13	0.06	NOTCH1, RNH1, WARS, ROCK1, SERPINF1, WNT5A, SEMA3E, FASLG, C3, MYDGF, LRG1, COL4A3, NF1	1.78
GO:1990542	mitochondrial transmembrane transport	5	0.06	MPC2, DNLZ, TIMM22, TIMM44, SLC25A33	3.27
GO:0030900	forebrain development	19	0.06	FOXB1, NOTCH1, SEMA3A, WNT5A, TSC1, INHBA, TBX19, GLI3, GRIN1, SLC6A4, TTC8, LHX3, CCDC85C, CDH2, ERBB4, HESX1, NF1, CDK5R1, PAFAH1B1	1.55
GO:0019585	glucuronate metabolic process	3	0.07	UGT2B31, UGT2A1, UGT1A6	6.97
GO:0006098	pentose-phosphate shunt	3	0.07	H6PD, RPE, TALDO1	6.97
GO:0072348	sulfur compound transport	3	0.07	LOC486150, SLC19A3, CTNS	6.97
GO:0006063	uronic acid metabolic process	3	0.07	UGT2B31, UGT2A1, UGT1A6	6.97
GO:0007158	neuron cell-cell adhesion	3	0.07	CEL, CDK5R1, ASTN1	6.97
GO:0030593	neutrophil chemotaxis	7	0.07	CCL25, CCL13, CCL8, CCL7, CCL20, CCL1, VAV1	2.44
GO:0007018	microtubule-based movement	14	0.07	DNAH12, DYNC1H1, SSNA1, WDR19, SPG7, TMEM141, IFT20, ADCY10, TMEM201, RHOT1, WDR81, KIF26B, KIF1B, PAFAH1B1	1.71
GO:1900542	regulation of purine nucleotide metabolic process	10	0.07	NPFFR2, NPPC, NOS2, GPR65, WNT5A, NF1, SIRT6, SLC25A23, SLC25A33, CBFA2T3	1.97
GO:0045089	positive regulation of innate immune response	11	0.07	TLR1, COLEC12, NLRP6, TRAF3, WNT5A, CARD9, CYBA, TLR10, TICAM1, CACTIN, VAV1	1.89
GO:0035556	intracellular signal transduction	100	0.07	NPFFR2, KLB, DGKD, RORA, GJA1, CDH2, RPS6KA2, AKT3, MAEL, MAP3K4, RGS7, PIAS4, UNC13C, MAP2K2, WSB1, DAPK3, WNT5A, SERPINF2, TSC1, TICAM1, HIC1, RAB34, NGEF, RABL6, NOTCH1, RASAL2, NLK, FHIT, ABR, RHOT1, BAG5, FLRT2, NLRP6, TBXA2R, INPP5D, NACC2, INPP5K, LRRC4C, MAP2K7, RALGDS, RAP1GAP2, SPHKAP, TNFSF14, MYO5A, INHBA, MOB1B, SETX, ADCY10, NF1, CDK10, PPP1R13B, HTR2B, PIK3CD, FASLG, TNFAIP1, MYDGF, RASGEF1B, GSG2, RALGPS2, SS18, MYOC, KSR1, SH2D3A, TRAF2, VAV1, TRDN, TLR1, TRAF4, RTN4RL1, ARHGEF3, TAX1BP3, PAFAH1B1, CCL13, RALA, ROCK1, CAB39, SEZ6, ASB14, PRR5L,	1.16

				CCL8, CCL7, ERBB4, CCL1, EPS8L2, CCL25, NOS2, CCL20, GPR55, CARD9, CDC42BPB, TPD52L1, SOD2, DEF8, RAB11B, SPSB1, PDE10A, TAOK1, CRLF3, NEK11, CDK5R1	
GO:0009150	purine ribonucleotide metabolic process	21	0.07	NPFFR2, SURF1, NDUFA7, NOS2, GPR65, LOC102151342, WNT5A, HTR2B, CTNS, SIRT6, RORA, ENO1, CBFA2T3, NPPC, NF1, ALDOC, NUDT16, SLC25A23, ADSS, SLC25A33, TEFM	1.50
GO:0032755	positive regulation of interleukin-6 production	6	0.07	TLR1, TNFSF4, WNT5A, CARD9, CYBA, TICAM1	2.73
GO:0060322	head development	31	0.07	BARHL1, RERE, NOTCH1, SEMA3A, ANKRD11, SEZ6, RORA, GLI3, AK8, SLC6A4, ABR, CCDC85C, CDH2, ERBB4, HESX1, CKB, FOXB1, MAP2K2, UTP3, WNT5A, CTNS, TSC1, INHBA, TBX19, EML1, GRIN1, TTC8, LHX3, NF1, CDK5R1, PAFAH1B1	1.37
GO:0006163	purine nucleotide metabolic process	22	0.07	NPFFR2, SURF1, NDUFA7, NOS2, GPR65, LOC102151342, WNT5A, HTR2B, CTNS, SIRT6, RORA, ENO1, FHIT, CBFA2T3, NPPC, NF1, ALDOC, NUDT16, SLC25A23, ADSS, SLC25A33, TEFM	1.48
GO:0050896	response to stimulus	288	0.07	NPFFR2, CCNK, SLC46A1, LOC611660, CPNE7, AQP4, ENO1, ALKBH7, COR1A3, ALKBH3, GJA1, SNRPD1, RPS6KA2, AKT3, OR3A10, LOC480667, RNF111, ARRDC5, UNC13C, ENTPD2, WSB1, DAPK3, SERPINF2, LOC491216, SIGIRR, COR1R4, NGEF, RABL6, SLC22A3, STXBP2, NLK, LIAS, GIGYF2, C3, RIC8A, ABR, GNG10, ADAMTS13, RHOT1, T, FLRT2, INPP5D, INPP5K, RALGDS, ABCA2, YES1, SPHKAP, TNFSF14, XRCC3, MYO5A, SETX, SULT1B1, ACER1, TNFSF4, TNFSF9, UNC119, CDK10, COR1P2, ITM2C, MYOM1, CETN1, LOC607002, GLI3, JCHAIN, SLC6A4, MYDGF, MAP1LC3C, GSG2, CHMP1A, ADAMTSL2, CCR6, SDF2, GIT1, RALGPS2, APPL1, SS18, MYOC, LOC102153243, SIRT6, CBFA2T3, VAV1, TLR1, FCER2, ARHGEF3, TLR10, TAX1BP3, CACTIN, ANGPTL1, PLIN5, PAFAH1B1, CCL13, LOC475935, CAB39, SSNA1, SEZ6, TYMS, OVCA2, CHRND, MNT, RXRA, CHRNG, GCNT3, MPZL1, GPR157, CCL25, FOXB1, CD70, CCL20, ODAM, GPR55, CARD9, ECEL1, WDR19, RPA1, FMO2, EMR4, DEF8, LOC479600, MIB1, RAB11B, GRIN1, SMOC2, XAB2, TTC8, SPSB1, LRG1, NMNAT1, RFWD2, TAOK1, KIF26B, SARM1, TRIP12, CRLF3, IL17C, CDK5R1, NEK11, KLB, TUSC5, DGKD, LOC489640, GPR65, RORA, FGF5, YY1, NPPC, CDH2, TBC1D32, MPC2, MAEL, NRTN, NUDT16, MAP3K4, RAG1, RGS7, SEMA6B, PIAS4, MAP2K2, WNT5A, THOC1, MATK, CYBA, UBE4B, TSC1, TICAM1, GTF2F1, KMO, HIC1, RAB34, COL4A3, DPEP1, PHPT1, CLEC4G, FBN3, NOTCH1, UBA5, CACNA1B, ILDR2, RASAL2, FHIT, SUPT6H, VTN, BRINP2, FUT7,	1.07

				BAG5, NLRP6, DEAF1, TBXA2R, NACC2, SAFB2, ABL2, LRRC4C, ASIC2, MAP2K7, RAP1GAP2, LOC611352, FANCA, TRPV3, TRPV1, INHBA, IFT20, MOB1B, ADCY10, CPS1, LHX3, COL5A1, NF1, BLMH, GNB5, GPSM1, PPP1R13B, ONECUT1, HTR2B, PIK3CD, FASLG, NRARP, TNFAIP1, DBH, ITGAE, ADGRE1, RASGEF1B, EXO1, TAGAP, DACT2, CA6, TOPBP1, HSP90AA1, EGFL7, KSR1, RFC1, SH2D3A, LANCL1, ARAP2, PLA2G4A, TRAF2, PPP2R5C, SAFB, TRDN, GREM2, TRAF4, RTN4RL1, TRAF3, SAG, SPACA3, NCBP3, ACPP, COLEC12, DTHD1, USP14, RALA, OR1E2, ROCK1, SEMA3D, NXN, SEMA3A, BECN2, ASB14, SEMA3E, PLG, PRR5L, GNA15, FZR1, NCLN, CCL8, CCL7, TMEM203, ERBB4, PDE6D, LOC483397, EPS8L2, CCL1, SLC25A23, S1PR4, UGT1A6, RPH3AL, NOS2, CTNS, CDC42BPB, SOD2, TPD52L1, DLK1, MYO1E, P2RX5, MYO1C, PDE10A, P2RX1, ACKR4, CCDC66, SLC25A33	
GO:0009123	nucleoside monophosphate metabolic process	14	0.07	SURF1, NDUFA7, LOC102151342, ENTPD8, CTNS, SIRT6, ENO1, TYMS, CBFA2T3, ALDOC, SLC25A23, ADSS, SLC25A33, TEFM	1.70
GO:0051345	positive regulation of hydrolase activity	21	0.07	CCL25, CCL13, ODAM, CCL20, GPR65, GPR55, WNT5A, HTR2B, TSC1, FASLG, TBC1D1, ABR, SRR, CCL8, CCL7, P2RX1, COL4A3, ABL2, TAX1BP3, CCL1, EVI5L	1.50
GO:0007420	brain development	29	0.07	BARHL1, RERE, NOTCH1, SEMA3A, SEZ6, RORA, GLI3, AK8, SLC6A4, ABR, CCDC85C, CDH2, ERBB4, HESX1, CKB, FOXB1, UTP3, WNT5A, CTNS, TSC1, INHBA, TBX19, EML1, GRIN1, TTC8, LHX3, NF1, CDK5R1, PAFAH1B1	1.39
GO:0043410	positive regulation of MAPK cascade	23	0.07	KLB, CCL25, CCL13, KSR1, CCL20, GPR55, SERPINF2, CARD9, WNT5A, HTR2B, TRAF2, TPD52L1, TLR1, MYDGF, CCL8, CCL7, CDH2, TRAF4, ERBB4, TAOK1, CDK10, CCL1, MAP3K4	1.46
GO:0016052	carbohydrate catabolic process	8	0.07	NEU2, CPS1, RPE, ENOSF1, SIRT6, ALDOC, ENO1, CBFA2T3	2.20
GO:0048841	regulation of axon extension involved in axon guidance	5	0.07	SEMA6B, SEMA3D, SEMA3A, WNT5A, SEMA3E	3.17
GO:0048846	axon extension involved in axon guidance	5	0.07	SEMA6B, SEMA3D, SEMA3A, WNT5A, SEMA3E	3.17
GO:0032210	regulation of telomere maintenance via telomerase	5	0.07	PKIB, TCP1, HNRNPU, MAP2K7, MAP3K4	3.17
GO:1902284	neuron projection extension involved in neuron projection guidance	5	0.07	SEMA6B, SEMA3D, SEMA3A, WNT5A, SEMA3E	3.17
GO:0050922	negative regulation of chemotaxis	6	0.07	SEMA6B, NOTCH1, SEMA3D, SEMA3A, WNT5A, SEMA3E	2.67

GO:0019752	carboxylic acid metabolic process	32	0.07	FH, ENO1, LIAS, ALKBH7, ACAT2, SRR, LIPC, ATCAY, ACADL, MPC2, ENOSF1, UGT2A1, PTGDS, ACAD11, UGT1A6, UGT2B31, WARS, NOS2, PIPOX, MYO5A, PLA2G4A, SIRT6, KMO, ACSF3, CBFA2T3, QKI, NMNAT1, CPS1, ASPG, ALDOC, LPIN2, PLIN5	1.35
GO:0006665	sphingolipid metabolic process	9	0.07	NEU2, GALC, ACER1, CERS4, P2RX1, ST8SIA4, DEGS2, CEL, SMPDL3A	2.05
GO:0046330	positive regulation of JNK cascade	9	0.07	TLR1, TRAF4, TAOK1, WNT5A, SERPINF2, CARD9, TRAF2, TPD52L1, MAP3K4	2.05
GO:0000723	telomere maintenance	8	0.07	PKIB, XRCC3, TCP1, RPA1, HNRNPU, MAP2K7, MAP3K4, SMG6	2.17
GO:0032200	telomere organization	8	0.07	PKIB, XRCC3, TCP1, RPA1, HNRNPU, MAP2K7, MAP3K4, SMG6	2.17
GO:0014032	neural crest cell development	7	0.07	SEMA6B, ERBB4, SEMA3D, SEMA3A, HTR2B, SEMA3E, NRTN	2.36
GO:0051017	actin filament bundle assembly	9	0.08	TTC8, ROCK1, MYOC, GPR65, SERPINF2, INPP5K, EVL, TSC1, TNFAIP1	2.02
GO:2000403	positive regulation of lymphocyte migration	4	0.08	TNFSF14, CCL7, CCL20, WNT5A	3.98
GO:0043436	oxoacid metabolic process	32	0.08	FH, ENO1, LIAS, ALKBH7, ACAT2, SRR, LIPC, ATCAY, ACADL, MPC2, ENOSF1, UGT2A1, PTGDS, ACAD11, UGT1A6, UGT2B31, WARS, NOS2, PIPOX, MYO5A, PLA2G4A, SIRT6, KMO, ACSF3, CBFA2T3, QKI, NMNAT1, CPS1, ASPG, ALDOC, LPIN2, PLIN5	1.34
GO:0009132	nucleoside diphosphate metabolic process	7	0.08	ENTPD2, ENTPD8, SIRT6, ALDOC, NUDT16, ENO1, CBFA2T3	2.32
GO:0051186	cofactor metabolic process	17	0.08	H6PD, RPE, TALDO1, PIPOX, FMO1, FMO2, SIRT6, ENO1, LIAS, TYMS, KMO, CBFA2T3, NDOR1, NMNAT1, MPC2, ALDOC, MVD	1.56
GO:0042033	chemokine biosynthetic process	3	0.08	SIGIRR, WNT5A, TICAM1	6.28
GO:0050667	homocysteine metabolic process	3	0.08	CPS1, DPEP1, BLMH	6.28
GO:0070166	enamel mineralization	3	0.08	ENAM, AMTN, WDR72	6.28
GO:0045073	regulation of chemokine biosynthetic process	3	0.08	SIGIRR, WNT5A, TICAM1	6.28
GO:0019682	glyceraldehyde-3-phosphate metabolic process	3	0.08	H6PD, RPE, TALDO1	6.28
GO:1903311	regulation of mRNA metabolic process	9	0.08	WTAP, SLTM, YTHDC1, LMNTD2, SAFB2, SAFB, NSRP1, SUPT6H, ZC3H14	2.00
GO:0031175	neuron projection development	34	0.08	RERE, NOTCH1, SEMA3D, SEMA3A, SEZ6, SEMA3E, GLI3, CAMSAP1, BAG5, ATCAY, FLRT2, MAP2, ABL2, NRTN, LRRC4C, RAP1GAP2, SEMA6B, FOXB1, MAP2K2, MYOC, WNT5A, OMG,	1.32

				UBE4B, SSH2, GRIN1, CAMSAP3, SETX, TTC8, LHX3, KIF26B, SARM1, ITM2C, CDK5R1, PAFAH1B1	
GO:0046486	glycerolipid metabolic process	14	0.08	PIGS, HTR2B, AGPAT2, GK2, PIGC, C3, PTDSS2, LIPC, CPS1, INPP5D, INPP5E, INPP5K, LPIN2, PLIN5	1.65
GO:0002443	leukocyte mediated immunity	14	0.08	STXBP2, THOC1, TRAF2, DBH, SUPT6H, VAV1, C3, FCER2, ABR, EXO1, TNFSF4, INPP5D, GCNT3, CLEC4G	1.65
GO:0032635	interleukin-6 production	8	0.08	TLR1, NOS2, TNFSF4, INPP5D, WNT5A, CARD9, CYBA, TICAM1	2.12
GO:0015931	nucleobase-containing compound transport	8	0.08	MRPL18, THOC1, TSC1, SLC25A23, SUPT6H, SLC25A33, QKI, SMG6	2.12
GO:0009259	ribonucleotide metabolic process	21	0.08	NPFFR2, SURF1, NDUFA7, NOS2, GPR65, LOC102151342, WNT5A, HTR2B, CTNS, SIRT6, RORA, ENO1, CBFA2T3, NPPC, NF1, ALDOC, NUDT16, SLC25A23, ADSS, SLC25A33, TEFM	1.46
GO:0030038	contractile actin filament bundle assembly	6	0.08	MYOC, GPR65, SERPINF2, INPP5K, EVL, TNFAIP1	2.56
GO:0050771	negative regulation of axonogenesis	6	0.08	SEMA6B, SEMA3D, SEMA3A, WNT5A, SEMA3E, CDK5R1	2.56
GO:0043149	stress fiber assembly	6	0.08	MYOC, GPR65, SERPINF2, INPP5K, EVL, TNFAIP1	2.56
GO:0002460	adaptive immune response based on somatic recombination of immune receptors built from immunoglobulin superfamily domains	12	0.08	TLR1, C3, FCER2, EXO1, TNFSF4, INPP5D, THOC1, GCNT3, TRAF2, RORA, SUPT6H, CLEC4G	1.74
GO:1904356	regulation of telomere maintenance via telomere lengthening	5	0.08	PKIB, TCP1, HNRNPU, MAP2K7, MAP3K4	2.99
GO:0051653	spindle localization	5	0.08	DYNC1H1, WDR81, SPAG5, NDC80, PAFAH1B1	2.99
GO:1901606	alpha-amino acid catabolic process	5	0.08	NOS2, ASPG, PIPOX, BLMH, KMO	2.99
GO:0098657	import into cell	5	0.08	SLC22A3, LOC489640, TRPV1, SLC9C2, SLC6A4	2.99
GO:2001057	reactive nitrogen species metabolic process	5	0.08	NOS2, CPS1, RORA, SOD2, TICAM1	2.99
GO:0000165	MAPK cascade	31	0.09	KLB, CCL13, HTR2B, NLK, MYDGF, CCL8, NLRP6, CCL7, CDH2, ERBB4, INPP5K, CCL1, MAP2K7, MAP3K4, CCL25, MAP2K2, KSR1, CCL20, GPR55, SERPINF2, CARD9, WNT5A, TRAF2, TPD52L1, TLR1, SETX, TRAF4, TAOK1, NF1, CDK10, PAFAH1B1	1.34
GO:0032787	monocarboxylic acid metabolic process	21	0.09	UGT2B31, MYO5A, SIRT6, ENO1, LIAS, KMO, ALKBH7, ACSF3, CBFA2T3, QKI, ACAT2, LIPC, ACADL, MPC2, ALDOC, UGT2A1, ACAD11, PTGDS, LPIN2, UGT1A6, PLIN5	1.45
GO:0021761	limbic system development	7	0.09	FOXB1, SEMA3A, NF1, TSC1, GLI3, CDK5R1, PAFAH1B1	2.25

GO:1901565	organonitrogen compound catabolic process	14	0.09	NOS2, PIPOX, DBH, CEL, KMO, GALC, NEU2, ACER1, ADAMTS13, ENOSF1, ASPG, BLMH, NUDT16, SMPDL3A	1.63
GO:1901135	carbohydrate derivative metabolic process	45	0.09	KLB, NPFFR2, PIGS, H6PD, GPR65, LOC607002, RPE, HTR2B, RORA, ENO1, TYMS, GK2, NEU2, FUT5, NPPC, FUT7, ST8SIA4, MAN1A1, NUDT16, SDF2, SLC25A23, LOC480667, ADSS, TEFM, LOC607011, SURF1, NDUFA7, NOS2, LOC102151342, WNT5A, CTNS, TALDO1, SIRT6, CBFA2T3, GBGT1, APRT, GALC, PIGC, UGDH, SOAT1, NF1, ALDOC, ACPP, SLC25A33, ITM2C	1.25
GO:0002712	regulation of B cell mediated immunity	5	0.09	C3, FCER2, TNFSF4, THOC1, SUPT6H	2.91
GO:0002889	regulation of immunoglobulin mediated immune response	5	0.09	C3, FCER2, TNFSF4, THOC1, SUPT6H	2.91
GO:1902667	regulation of axon guidance	5	0.09	SEMA6B, SEMA3D, SEMA3A, WNT5A, SEMA3E	2.91
GO:0002221	pattern recognition receptor signaling pathway	8	0.09	TLR1, COLEC12, NLRP6, TRAF3, CYBA, TLR10, TICAM1, CACTIN	2.07
GO:0071705	nitrogen compound transport	24	0.09	SLC22A3, SLC46A1, LOC486150, NOS2, SLC22A2, MRPL18, THOC1, MYO5A, CTNS, ILDR2, TSC1, DOC2B, TRPV1, SUPT6H, QKI, RAB11B, SLC6A4, SMG6, P2RX1, MPC2, SLC19A3, NF1, SLC25A23, SLC25A33	1.40
GO:0071934	thiamine transmembrane transport	2	0.09	LOC486150, SLC19A3	20.92
GO:0003213	cardiac right atrium morphogenesis	2	0.09	NOTCH1, WNT5A	20.92
GO:0070175	positive regulation of enamel mineralization	2	0.09	ENAM, AMTN	20.92
GO:0070995	NADPH oxidation	2	0.09	FMO1, FMO2	20.92
GO:0001976	neurological system process involved in regulation of systemic arterial blood pressure	3	0.09	RPS6KA2, SOD2, ASIC2	5.71
GO:0007350	blastoderm segmentation	3	0.09	SEMA3A, WNT5A, TDRD5	5.71
GO:0010863	positive regulation of phospholipase C activity	3	0.09	GPR55, HTR2B, ABL2	5.71
GO:0050755	chemokine metabolic process	3	0.09	SIGIRR, WNT5A, TICAM1	5.71
GO:0060831	smoothened signaling pathway involved in dorsal/ventral neural tube patterning	3	0.09	TBC1D32, WDR19, GLI3	5.71
GO:0051923	sulfation	3	0.09	SULT1B1, SULT1D1, LOC482182	5.71

GO:0043408	regulation of MAPK cascade	30	0.09	KLB, NPFFR2, CCL13, HTR2B, MYDGF, CCL8, NLRP6, CCL7, CDH2, ERBB4, INPP5K, CCL1, MAP3K4, CCL25, KSR1, MYOC, CCL20, GPR55, SERPINF2, CARD9, WNT5A, TRAF2, INHBA, TPD52L1, TLR1, TRAF4, TAOK1, NF1, CDK10, PAFAH1B1	1.33
GO:0006491	N-glycan processing	4	0.09	LOC607002, ST8SIA4, MAN1A1, LOC480667	3.64
GO:0016071	mRNA metabolic process	23	0.09	WTAP, YTHDC1, LMNTD2, SAFB, LSM5, PRPF8, SUPT6H, QKI, SMG6, SETX, XAB2, HNRNPM, SLTM, LOC480186, SNRPD1, KHSRP, SAFB2, GEMIN4, NUDT16, CDK13, NSRP1, ZC3H14, DIS3L2	1.41
GO:0006140	regulation of nucleotide metabolic process	10	0.09	NPFFR2, NPPC, NOS2, GPR65, WNT5A, NF1, SIRT6, SLC25A23, SLC25A33, CBFA2T3	1.83
GO:0046395	carboxylic acid catabolic process	10	0.09	NOS2, ACADL, ENOSF1, ASPG, PIPOX, ACAD11, LPIN2, ACSF3, ACAT2, PLIN5	1.83
GO:0032526	response to retinoic acid	6	0.09	SETX, BRINP2, RXRA, YES1, ABL2, OVCA2	2.46
GO:0001944	vasculature development	29	0.10	RNH1, NOTCH1, ROCK1, PLG, FASLG, SEMA3E, NRARP, RORA, GLI3, RIC8A, C3, MYDGF, GJA1, T, CDH2, WARS, SERPINF1, SERPINF2, WNT5A, FOXN1, QKI, MIB1, MYO1E, LRG1, COL5A1, NCL, COL4A3, NF1, ANGPTL4	1.33
GO:0042592	homeostatic process	63	0.10	ATP2A3, HTR2B, HNRNPU, AQP4, RORA, DBH, TMEM97, SLC4A4, SLC9C2, JCHAIN, SMG6, GJA1, LIPC, ACADL, CDH2, OBP2B, MPC2, QSOX2, ACAD11, RAG2, MAP3K4, RAG1, MYOC, SIRT6, CYBA, TRDN, SOAT1, TCP1, ANGPTL4, FH, NXN, BECN2, ANKRD11, ILDR2, PLG, SPATA7, GIGYF2, GNA15, CCL8, RHOT1, TMEM203, TBXA2R, INPP5D, INPP5K, ABL2, CKB, MAP2K7, SLC25A23, ABCA2, RPH3AL, GPR55, XRCC3, RPA1, MYO5A, TRPV1, INHBA, SOD2, FOXN1, PRDX6, RAB11B, GRIN1, PKIB, NF1	1.19
GO:1901342	regulation of vasculature development	13	0.10	NOTCH1, RNH1, WARS, ROCK1, SERPINF1, WNT5A, SEMA3E, FASLG, C3, MYDGF, LRG1, COL4A3, NF1	1.64
GO:0090066	regulation of anatomical structure size	23	0.10	RAP1GAP2, SEMA6B, NOS2, SEMA3D, SEMA3A, SERPINF2, WNT5A, HTR2B, TSC1, SEMA3E, AQP4, DBH, SOD2, SSH2, VAV1, NPPC, CPS1, TBXA2R, P2RX1, EVL, ASIC2, RILP, CDK5R1	1.40
Cellular component (GO Term)		Count	PValue	Genes	Fold Enrichment
GO:0005576	extracellular region	172	0.00	RNH1, CPNE4, CPNE7, LOC489640, PITPNA, CSN3, ENO1, CSN2, SLC4A4, FGF5, NPPC, LCNL1, GJA1, LIPC, CDH2, PSMD1, PMPCA, NRTN, PTGDS, ENTPD2, ARRDC1, SERPINF1, WNT5A, SERPINF2, THOC1, KMO, EML5, NCL, COL4A3, GEMIN4, DPEP1, PHPT1,	1.22

				FBN3, PSMD11, PSMD13, MRPL18, SLC22A2, STXBP2, FHIT, ADAMTS10, ACAT2, C3, VTN, GNG10, DPP7, ADAMTS13, FLRT2, LCN15, SAFB2, PRR27, LRRC4C, RAB11FIP4, YES1, SPHKAP, PSPN, MYO5A, DNAJC13, INHBA, EEF2, PRDX6, IGF2R, UBAC1, IFT20, MOB1B, HNRNPM, COL5A1, TNFSF4, CPD, UNC119, BLMH, CDK13, ITM2C, CD320, CLIC3, FASLG, DBH, JCHAIN, PTPRG, MYDGF, RPL7A, CANF2, C8G, OBP2B, ADAMTSL2, CHMP1A, CA6, PGM2, MAN1A1, QSOX2, CSN1S1, UBXN6, APPL1, SLC13A2, CLUL1, WARS, MYOC, RFC1, ANXA2, LOC102153243, LANCL1, EDF1, APRT, DNM3, GREM2, FCER2, UGDH, ENAM, RTN4RL1, TCP1, PLIN4, AMTN, SPACA3, TAX1BP3, ALDOC, ANGPTL4, CDH15, ACPP, CACTIN, SMPDL3A, ASPA, ANGPTL1, PAPPA2, PAFAH1B1, COLEC12, LOC491264, CCL13, USP14, FH, RALA, CAB39, SEMA3D, SEMA3A, SEZ6, PROCA1, SEMA3E, PLG, HSD17B12, RETN, PSMA8, GK2, CCL8, CCL7, GPA33, FAM184A, CREG1, GCNT3, CCL1, EPS8L2, CKB, GC, ADSS, CCL25, DYNC1H1, LRRC26, CCL20, CD70, ODAM, CTNS, CDC42BPB, TUBB4B, TUBB4A, DLK1, RAB11B, MYO1D, GALNS, SMOC2, MYO1E, LRG1, MYO1C, TAOK1, TRIP10, IL17C	
GO:0044421	extracellular region part	158	0.01	RNH1, CPNE4, CPNE7, LOC489640, PITPNA, CSN3, ENO1, CSN2, SLC4A4, GJA1, LIPC, CDH2, PSMD1, PMPCA, PTGDS, ENTPD2, ARRDC1, SERPINF1, WNT5A, SERPINF2, THOC1, KMO, EML5, NCL, COL4A3, GEMIN4, DPEP1, PHPT1, FBN3, PSMD11, PSMD13, MRPL18, SLC22A2, STXBP2, FHIT, ADAMTS10, ACAT2, C3, VTN, GNG10, DPP7, ADAMTS13, FLRT2, SAFB2, PRR27, LRRC4C, RAB11FIP4, YES1, SPHKAP, MYO5A, DNAJC13, INHBA, EEF2, PRDX6, IGF2R, UBAC1, IFT20, MOB1B, HNRNPM, COL5A1, TNFSF4, CPD, UNC119, BLMH, CDK13, ITM2C, CD320, CLIC3, FASLG, DBH, JCHAIN, PTPRG, MYDGF, RPL7A, C8G, OBP2B, ADAMTSL2, CHMP1A, CA6, PGM2, MAN1A1, QSOX2, UBXN6, APPL1, SLC13A2, WARS, MYOC, RFC1, ANXA2, LANCL1, EDF1, APRT, DNM3, GREM2, FCER2, UGDH, ENAM, RTN4RL1, TCP1, AMTN, SPACA3, TAX1BP3, ALDOC, ANGPTL4, CDH15, ACPP, CACTIN, SMPDL3A, ASPA, ANGPTL1, PAPPA2, PAFAH1B1, COLEC12, CCL13, USP14, FH, RALA, CAB39, SEMA3D, SEMA3A, SEZ6, SEMA3E, HSD17B12, RETN, PSMA8, GK2, CCL8, CCL7, GPA33, FAM184A, CREG1, GCNT3, CCL1, EPS8L2, CKB, GC, ADSS, CCL25, DYNC1H1, LRRC26, CCL20, CD70, ODAM, CTNS,	1.21

				CDC42BPB, TUBB4B, TUBB4A, DLK1, RAB11B, MYO1D, GALNS, SMOC2, MYO1E, LRG1, MYO1C, TAOK1, TRIP10, IL17C	
GO:0005720	nuclear heterochromatin	5	0.02	SUZ12, KDM4B, UHRF1, TCP1, SIRT6	4.46
GO:0005615	extracellular space	55	0.03	FASLG, CSN3, DBH, CSN2, ENO1, JCHAIN, MYDGF, C8G, LIPC, OBP2B, CA6, PMPCA, QSOX2, PTGDS, MYOC, SERPINF1, SERPINF2, WNT5A, GREM2, SPACA3, ANGPTL4, ACPP, SMPDL3A, ANGPTL1, CCL13, SEMA3D, SEMA3A, MRPL18, SEZ6, SEMA3E, RETN, C3, VTN, CCL8, ADAMTS13, CCL7, FLRT2, FAM184A, CREG1, CCL1, CKB, GC, LRRC4C, RAB11FIP4, CCL25, CCL20, ODAM, INHBA, PRDX6, DLK1, LRG1, TNFSF4, CPD, CDK13, IL17C	1.31
GO:0070062	extracellular exosome	115	0.04	RNH1, CPNE4, CPNE7, LOC489640, PITPNA, ENO1, SLC4A4, GJA1, CDH2, PSMD1, PTGDS, ENTPD2, ARRDC1, SERPINF1, SERPINF2, KMO, EML5, NCL, GEMIN4, DPEP1, PHPT1, PSMD11, PSMD13, SLC22A2, STXBP2, FHIT, ACAT2, C3, VTN, GNG10, DPP7, FLRT2, SAFB2, PRR27, YES1, SPHKAP, MYO5A, DNAJC13, EEF2, PRDX6, IGF2R, UBAC1, IFT20, MOB1B, HNRNPM, COL5A1, CPD, BLMH, ITM2C, CD320, CLIC3, FASLG, JCHAIN, PTPRG, MYDGF, RPL7A, C8G, OBP2B, CHMP1A, CA6, PGM2, MAN1A1, UBXN6, APPL1, SLC13A2, WARS, MYOC, RFC1, LANCL1, EDF1, APRT, DNM3, FCER2, UGDH, RTN4RL1, TCP1, TAX1BP3, ALDOC, CDH15, ACPP, CACTIN, SMPDL3A, ASPA, ANGPTL1, PAPPA2, PAFAH1B1, COLEC12, USP14, FH, RALA, CAB39, RETN, PSMA8, GK2, GPA33, CREG1, GCNT3, EPS8L2, GC, ADSS, DYNC1H1, LRRC26, CD70, CTNS, CDC42BPB, TUBB4B, TUBB4A, RAB11B, MYO1D, GALNS, MYO1E, LRG1, MYO1C, TAOK1, TRIP10	1.18
GO:0032391	photoreceptor connecting cilium	5	0.04	IFT20, TTC8, CETN1, WDR19, SPATA7	3.82
GO:0031090	organelle membrane	70	0.04	NDUFA11, ATP2A3, DBH, TMEM97, ATCAY, MAP1LC3C, MPC2, RPS6KA2, CHMP1A, MYO18A, PMPCA, CEP170, KIF1B, MAN1A1, QSOX2, PTGDS, ACAD11, C20H19ORF70, APPL1, MYOC, LOC102151342, TRAF2, TIMM22, KMO, MFSD12, GBGT1, SIRT3, TRDN, DNM3, ACPP, PAFAH1B1, STXBP2, GOSR1, SPG7, TYMS, FUT5, TMEM201, FZR1, FUT7, RHOT1, INPP5E, ST8SIA4, PDE6D, EMC6, RILP, RAB11FIP4, B4GALNT4, RAP1GAP2, SEC16B, UGT2B31, SURF1, NDUFA7, SEC16A, FMO1, CTNS, TIMM44, DNAJC13, FMO2, FMO3, SOD2, FMO4, RAB11B, LOC490346, TMEM199, P2RX5, ATG16L1, P2RX1, VPS41, SARM1, SLC25A33	1.24
GO:0043230	extracellular organelle	115	0.05	RNH1, CPNE4, CPNE7, LOC489640, PITPNA, ENO1, SLC4A4, GJA1, CDH2, PSMD1, PTGDS, ENTPD2, ARRDC1, SERPINF1, SERPINF2,	1.17

				KMO, EML5, NCL, GEMIN4, DPEP1, PHPT1, PSMD11, PSMD13, SLC22A2, STXBP2, FHIT, ACAT2, C3, VTN, GNG10, DPP7, FLRT2, SAFB2, PRR27, YES1, SPHKAP, MYO5A, DNAJC13, EEF2, PRDX6, IGF2R, UBAC1, IFT20, MOB1B, HNRNPM, COL5A1, CPD, BLMH, ITM2C, CD320, CLIC3, FASLG, JCHAIN, PTPRG, MYDGF, RPL7A, C8G, OBP2B, CHMP1A, CA6, PGM2, MAN1A1, UBXN6, APPL1, SLC13A2, WARS, MYOC, RFC1, LANCL1, EDF1, APRT, DNM3, FCER2, UGDH, RTN4RL1, TCP1, TAX1BP3, ALDOC, CDH15, ACPP, CACTIN, SMPDL3A, ASPA, ANGPTL1, PAPPA2, PAFAH1B1, COLEC12, USP14, FH, RALA, CAB39, RETN, PSMA8, GK2, GPA33, CREG1, GCNT3, EPS8L2, GC, ADSS, DYNC1H1, LRRC26, CD70, CTNS, CDC42BPB, TUBB4B, TUBB4A, RAB11B, MYO1D, GALNS, MYO1E, LRG1, MYO1C, TAOK1, TRIP10	
GO:1903561	extracellular vesicle	115	0.05	RNH1, CPNE4, CPNE7, LOC489640, PITPNA, ENO1, SLC4A4, GJA1, CDH2, PSMD1, PTGDS, ENTPD2, ARRDC1, SERPINF1, SERPINF2, KMO, EML5, NCL, GEMIN4, DPEP1, PHPT1, PSMD11, PSMD13, SLC22A2, STXBP2, FHIT, ACAT2, C3, VTN, GNG10, DPP7, FLRT2, SAFB2, PRR27, YES1, SPHKAP, MYO5A, DNAJC13, EEF2, PRDX6, IGF2R, UBAC1, IFT20, MOB1B, HNRNPM, COL5A1, CPD, BLMH, ITM2C, CD320, CLIC3, FASLG, JCHAIN, PTPRG, MYDGF, RPL7A, C8G, OBP2B, CHMP1A, CA6, PGM2, MAN1A1, UBXN6, APPL1, SLC13A2, WARS, MYOC, RFC1, LANCL1, EDF1, APRT, DNM3, FCER2, UGDH, RTN4RL1, TCP1, TAX1BP3, ALDOC, CDH15, ACPP, CACTIN, SMPDL3A, ASPA, ANGPTL1, PAPPA2, PAFAH1B1, COLEC12, USP14, FH, RALA, CAB39, RETN, PSMA8, GK2, GPA33, CREG1, GCNT3, EPS8L2, GC, ADSS, DYNC1H1, LRRC26, CD70, CTNS, CDC42BPB, TUBB4B, TUBB4A, RAB11B, MYO1D, GALNS, MYO1E, LRG1, MYO1C, TAOK1, TRIP10	1.17
GO:0005891	voltage-gated calcium channel complex	5	0.05	CATSPERD, CACNA1B, CACNA2D3, C7H1ORF101, TRDN	3.45
GO:0043186	P granule	3	0.08	TDRD9, MAEL, TDRD5	6.42
GO:0060293	germ plasm	3	0.08	TDRD9, MAEL, TDRD5	6.42
GO:0045495	pole plasm	3	0.08	TDRD9, MAEL, TDRD5	6.42
GO:0016459	myosin complex	6	0.08	MYO1D, MYO1E, MYO1C, MYO18A, MYO5A, MYO1F	2.62
GO:0002945	cyclin K-CDK13 complex	2	0.09	CCNK, CDK13	21.41

GO:0005929	cilium	21	0.10	MYOC, CETN1, ODF3, SSNA1, WDR19, MYO5A, TUBB4A, TMEM141, GLI3, AK8, SPATA7, IFT20, ADCY10, TTC8, ATG16L1, CATSPERD, INPP5E, SAG, GNB5, GAS8, PAFAH1B1	1.43
GO:0005759	mitochondrial matrix	12	0.10	MRPL41, MRPL3, CPS1, POLDIP2, CLPP, TIMM44, ERAL1, TYMS, SOD2, ALKBH7, TEFM, SIRT3	1.69
Molecular functions (GO Terms)		Count	PValue	Genes	Fold Enrichment
GO:0004499	N,N-dimethylaniline monooxygenase activity	5	0.00	FMO1, FMO2, FMO3, FMO4, LOC490346	15.54
GO:0016709	oxidoreductase activity, acting on paired donors, with incorporation or reduction of molecular oxygen, NAD(P)H as one donor, and incorporation of one atom of oxygen	7	0.00	NOS2, FMO1, FMO2, FMO3, KMO, FMO4, LOC490346	6.09
GO:0050660	flavin adenine dinucleotide binding	11	0.00	NDOR1, NOS2, ACADL, FMO1, FMO2, FMO3, KMO, ACAD11, FMO4, DUS3L, LOC490346	3.47
GO:0030234	enzyme regulator activity	48	0.00	GPSM1, USP14, CCNK, RNH1, NOTCH1, CAB39, HTR2B, CSN2, RASAL2, RIC8A, C3, ABR, FZR1, SRR, ADAP2, CCL8, FLRT2, PDE6D, PSMD1, EVI5L, LRRC4C, RALGDS, GIT1, TEFM, RGS7, RAP1GAP2, PPP1R26, TNFSF14, ANXA2, SERPINF1, SERPINF2, ARAP2, TSC1, PPP2R5C, GTF2F1, PCP2, GALC, MOB1B, TBC1D1, PKIB, RTN4RL1, NF1, FGFR1OP, DPEP1, GNB5, ANGPTL4, APBA3, CDK5R1	1.60
GO:0048020	CCR chemokine receptor binding	6	0.00	CCL25, CCL13, CCL8, CCL7, CCL20, CCL1	6.22
GO:0030695	GTPase regulator activity	19	0.00	RAP1GAP2, GPSM1, HTR2B, ARAP2, TSC1, RASAL2, PCP2, RIC8A, TBC1D1, ABR, SRR, ADAP2, PDE6D, NF1, GNB5, EVI5L, RALGDS, GIT1, RGS7	2.14
GO:0098772	molecular function regulator	60	0.00	GPSM1, CCNK, RNH1, HTR2B, CSN2, RASGEF1B, PSMD1, EVI5L, GIT1, RALGPS2, RGS7, ANXA2, SH2D3A, SERPINF1, SERPINF2, ARAP2, CACNA2D3, TSC1, PPP2R5C, GTF2F1, VAV1, PCP2, TBC1D1, RTN4RL1, ARHGEF3, DPEP1, AMPH, ANGPTL4, PHPT1, NGEF, USP14, NOTCH1, CAB39, RASAL2, RIC8A, C3, ABR, SRR, FZR1, CCL8, ADAP2, FLRT2, PDE6D, EPS8L2, LRRC4C, RALGDS, TEFM, PPP1R26, RAP1GAP2, LRRC26, TNFSF14, MOB1B, GALC, PKIB, TRIP10, NF1, FGFR1OP, GNB5, APBA3, CDK5R1	1.45

GO:0050662	coenzyme binding	16	0.00	H6PD, NOS2, FMO1, FMO2, SIRT6, FMO3, FMO4, KMO, LOC490346, SIRT3, UGDH, NDOR1, ACADL, SOAT1, ACAD11, DUS3L	2.31
GO:0050661	NADP binding	7	0.00	NDOR1, H6PD, FMO1, FMO2, FMO3, FMO4, LOC490346	4.35
GO:0060589	nucleoside-triphosphatase regulator activity	19	0.01	RAP1GAP2, GPSM1, HTR2B, ARAP2, TSC1, RASAL2, PCP2, RIC8A, TBC1D1, ABR, SRR, ADAP2, PDE6D, NF1, GNB5, EVI5L, RALGDS, GIT1, RGS7	1.99
GO:0008009	chemokine activity	6	0.01	CCL25, CCL13, CCL8, CCL7, CCL20, CCL1	4.35
GO:0048037	cofactor binding	19	0.01	H6PD, NOS2, FMO1, FMO2, SIRT6, FMO3, FMO4, KMO, SLC9C2, LOC490346, SIRT3, UGDH, SRR, NDOR1, ACADL, SOAT1, CREG1, ACAD11, DUS3L	1.90
GO:0042379	chemokine receptor binding	6	0.01	CCL25, CCL13, CCL8, CCL7, CCL20, CCL1	4.21
GO:0003824	catalytic activity	234	0.02	CCNK, EHMT1, ENO1, ALKBH3, LIPC, LOC100856068, RPS6KA2, AKT3, ACAD11, PTGDS, LOC480667, RNF111, ENTPD2, WSB1, DAPK3, ENTPD8, HSD11B1L, TALDO1, CEL, GBGT1, SOAT1, CLPP, DUS3L, EXD3, NLK, LIAS, ABO, ADAMTS10, NEU2, PTDSS2, ADAMTS13, RHOT1, INPP5D, INPP5E, ST8SIA4, INPP5K, PIP5K1C, B4GALNT4, LOC607011, DNAH12, ABCA2, SURF1, YES1, XRCC3, MYO5A, ERAL1, PRDX6, SULT1B1, ACER1, LOC480074, CDK10, CDK13, PIGS, SETD3, LOC607002, SMG6, PTPRG, ACADL, GSG2, ADAMTSL2, KIF1B, QSOX2, SDF2, WARS, SIRT6, SIRT3, APRT, PIGC, UGDH, CTU2, SULT1D1, FAM20B, ALDOC, RNF166, LOC610994, FH, CAB39, PROCA1, TYMS, CAMKK1, PSMA8, CREG1, METTL4, GCNT3, CKB, SUZ12, DYNC1H1, PNPLA7, ECEL1, MOK, FMO1, FMO2, FMO3, DOHH, FMO4, TUBB4B, TUBB4A, MIB1, RAB11B, SPSB1, NMNAT1, RFWD2, TAOK1, KIF26B, TRIP12, NEK11, KLB, DGKD, STKLD1, SLC9C2, PMPCA, NUDT16, RAG2, PAMR1, MAP3K4, RAG1, PIAS4, MAP2K2, MATK, PIPOX, CYBA, UBE4B, GTF2F1, KMO, PRSS56, LOC485024, DPEP1, PHPT1, H6PD, ABHD3, UBA5, PLD5, FHIT, PRPF8, ACAT2, AK8, FUT5, FUT7, NDOR1, DPP7, DPP9, NACC2, ABL2, MAP2K7, MARK3, TDRD9, EEF2, SSH2, ACSF3, MARCH2, ADCY10, CPS1, CPD, TADA1, BLMH, DEGS2, ATP2A3, PIK3CD, PTPN21, TNFAIP1, DBH, METTL13, EXO1, ENOSF1, MYO18A, WDR5, CA6, PRKG2, PGM2, MAN1A1, UGT2A1, METTL16, DIS3L2, CERS4, KSR1, RFC1, RNASET2, LANCL1, PLA2G4A, TRAF2, DUSP27, DCK, DNM3, TRAF4, TRAF3, ASPG, SPACA3, MVD, ACPP, SMPDL3A, ASPA, PAPPA2, USP14, RALA, ROCK1, UHRF1, NXN, RPE, ASB14, PLG, GLT6D1, SPG7, HSD17B12, AGPAT2, GK2, MRM3, AGPAT4,	1.12

				GNA15, CCL8, ERBB4, FAM63B, PDE6D, ADSS, UGT1A6, UGT2B31, NDUFA7, KDM4B, NOS2, NAA11, LOC476732, LOC482182, CDC42BPB, SOD2, LOC490346, MYO1D, GALNS, GALC, MYO1E, MYO1C, PDE10A, SARDH, TRMT61A, LPIN2, MYO1F	
GO:0045499	chemorepellent activity	5	0.03	SEMA6B, FLRT2, SEMA3D, SEMA3A, SEMA3E	4.35
GO:0036094	small molecule binding	109	0.03	DGKD, STKLD1, SMCHD1, LCNL1, RPS6KA2, AKT3, NUDT16, PTGDS, ACAD11, MAP3K4, MAP2K2, LOC100688619, DAPK3, MATK, UBE4B, KMO, SOAT1, RAB34, NCL, DUS3L, RABL6, H6PD, NLK, AK8, RHOT1, ADAP2, NDOR1, NLRP6, LCN15, SAFB2, ABL2, MAP2K7, MARK3, DNAH12, ABCA2, YES1, XRCC3, MYO5A, TRPV1, ERAL1, EEF2, IGF2R, HNRNPM, CPS1, CDK10, CDK13, ITM2C, CD320, ATP2A3, CELF5, DBH, ELAVL1, CHD1, ACADL, GSG2, MYO18A, PRKG2, KIF1B, HSP90AA1, WARS, RFC1, KSR1, SIRT6, SAFB, DCK, SIRT3, APRT, DNM3, UGDH, SLTM, TCP1, NCBP3, MVD, LOC491264, RALA, ROCK1, SPG7, CAMKK1, GNA15, ERBB4, CREG1, CKB, GC, ADSS, DYNC1H1, NOS2, ATAD5, MOK, FMO1, FMO2, CDC42BPB, FMO3, FMO4, TUBB4B, TUBB4A, SEPT14, RAB11B, LOC490346, MYO1D, MYO1E, P2RX5, MYO1C, PDE10A, NMNAT1, TAOK1, KIF26B, P2RX1, MYO1F, NEK11	1.19
GO:0004497	monooxygenase activity	9	0.04	NOS2, FMO1, FMO2, DBH, DOHH, FMO3, KMO, FMO4, LOC490346	2.36
GO:0015020	glucuronosyltransferase activity	4	0.04	UGT2B31, LOC100856068, UGT2A1, UGT1A6	5.12
GO:0004857	enzyme inhibitor activity	19	0.05	PPP1R26, USP14, NOTCH1, RNH1, TNFSF14, ANXA2, SERPINF1, SERPINF2, CSN2, C3, PKIB, FLRT2, RTN4RL1, PDE6D, FGFR1OP, DPEP1, ANGPTL4, APBA3, LRRC4C	1.62
GO:0016758	transferase activity, transferring hexosyl groups	13	0.05	UGT2B31, GLT6D1, ABO, GBGT1, PIGC, FUT5, FUT7, LOC100856068, GCNT3, SDF2, UGT2A1, B4GALNT4, UGT1A6	1.86
GO:0051183	vitamin transporter activity	4	0.05	SLC46A1, LOC486150, SLC19A3, GC	4.83
GO:0038191	neuropilin binding	3	0.05	SEMA3D, SEMA3A, SEMA3E	8.16
GO:0015651	quaternary ammonium group transmembrane transporter activity	3	0.05	SLC22A3, SLC25A29, SLC22A2	8.16
GO:0016811	hydrolase activity, acting on carbon-nitrogen (but not peptide) bonds, in linear amides	6	0.05	ACER1, NACC2, ASPG, SIRT6, ASPA, SIRT3	2.97
GO:0003774	motor activity	10	0.05	MYO1D, MYO1E, DYNC1H1, DNAH12, MYO1C, KIF26B, MYO18A, MYO5A, KIF1B, MYO1F	2.07
GO:0005515	protein binding	70	0.05	KLB, PPP1R13B, HTR2B, TNFAIP2, DOC2B, JCHAIN, FGF5, MAP1LC3C, DACT2, EVI5L, SEMA6B, MAP2K2, ANXA2, MYOC,	1.22

				WNT5A, MATK, CEL, TRDN, TLR1, TMEM210, DNM3, TBC1D1, WDR81, DNLZ, VAMP4, RNF166, GAS8, PAFAH1B1, CCL13, GTF2A2, SEMA3D, SEMA3A, STXBP2, GOSR1, ASB14, SEMA3E, RETN, NLK, RIC8A, SRR, TMEM201, CCL8, CCL7, T, MAP2, PDE6D, CCL1, KANSL1L, PPP1R26, CCL25, RPH3AL, RANBP3, YES1, SPHKAP, NOS2, CCL20, CD70, PHF12, TIMM44, TRPV1, INHBA, SSH2, LOC479600, QKI, TNFSF4, VPS41, CORO6, TNFSF9, IL17C, RCOR1	
GO:0030215	semaphorin receptor binding	4	0.05	SEMA6B, SEMA3D, SEMA3A, SEMA3E	4.58
GO:0005096	GTPase activator activity	14	0.06	RAP1GAP2, HTR2B, ARAP2, RASAL2, RIC8A, TBC1D1, ABR, SRR, ADAP2, NF1, GNB5, EVI5L, GIT1, RGS7	1.76
GO:0015924	mannosyl-oligosaccharide mannosidase activity	3	0.06	LOC607002, MAN1A1, LOC480667	7.25
GO:0015101	organic cation transmembrane transporter activity	3	0.06	SLC22A3, SLC25A29, SLC22A2	7.25
GO:0004571	mannosyl-oligosaccharide 1,2-alpha-mannosidase activity	3	0.06	LOC607002, MAN1A1, LOC480667	7.25
GO:0008017	microtubule binding	5	0.06	DNM3, WDR81, MAP1LC3C, MAP2, PAFAH1B1	3.30
GO:0005126	cytokine receptor binding	10	0.07	CCL25, CCL13, CCL8, CCL7, CCL20, TNFSF4, TNFSF9, CCL1, INHBA, IL17C	1.98
GO:0005102	receptor binding	26	0.07	KLB, CCL13, SEMA3D, SEMA3A, SEMA3E, RETN, JCHAIN, FGF5, CCL8, CCL7, CCL1, CCL25, SEMA6B, YES1, MYOC, CCL20, CD70, WNT5A, MATK, CEL, INHBA, TRDN, TLR1, TNFSF4, TNFSF9, IL17C	1.42
GO:0008504	monoamine transmembrane transporter activity	3	0.07	SLC22A3, SLC22A2, SLC6A4	6.53
GO:0016740	transferase activity	97	0.08	CCNK, DGKD, EHMT1, STKLD1, LOC100856068, RPS6KA2, AKT3, RAG2, MAP3K4, RNF111, RAG1, PIAS4, MAP2K2, WSB1, DAPK3, MATK, TALDO1, UBE4B, GBGT1, SOAT1, LIAS, NLK, ABO, AK8, ACAT2, FUT5, PTDSS2, FUT7, ST8SIA4, ABL2, PIP5K1C, MAP2K7, MARK3, B4GALNT4, LOC607011, YES1, MARCH2, SULT1B1, CPS1, LOC480074, TADA1, CDK10, CDK13, SETD3, PIK3CD, TNFAIP1, METTL13, GSG2, WDR5, PRKG2, UGT2A1, SDF2, METTL16, CERS4, KSR1, TRAF2, SIRT6, DCK, APRT, PIGC, CTU2, SULT1D1, TRAF4, TRAF3, FAM20B, RNF166, ROCK1, CAB39, UHRF1, ASB14, GLT6D1, TYMS, AGPAT2, CAMKK1, MRM3, AGPAT4, GK2, CCL8, ERBB4, METTL4, GCNT3, CKB, UGT1A6, SUZ12, UGT2B31, NAA11,	

				MOK, LOC482182, CDC42BPB, MIB1, SPSB1, NMNAT1, RFWD2, TAOK1, TRIP12, TRMT61A, NEK11	
GO:1901265	nucleoside phosphate binding	99	0.08	DGKD, STKLD1, SMCHD1, RPS6KA2, AKT3, NUDT16, ACAD11, MAP3K4, MAP2K2, LOC100688619, DAPK3, MATK, UBE4B, KMO, RAB34, NCL, DUS3L, RABL6, H6PD, NLK, AK8, RHOT1, NDOR1, NLRP6, SAFB2, ABL2, MAP2K7, MARK3, DNAH12, ABCA2, YES1, XRCC3, MYO5A, TRPV1, ERAL1, EEF2, HNRNPM, CPS1, CDK10, CDK13, ITM2C, ATP2A3, CELF5, ELAVL1, CHD1, ACADL, GSG2, MYO18A, PRKG2, KIF1B, HSP90AA1, WARS, RFC1, KSR1, SIRT6, SAFB, DCK, SIRT3, APRT, DNM3, UGDH, SLTM, TCP1, NCBP3, MVD, RALA, ROCK1, SPG7, CAMKK1, GNA15, ERBB4, CREG1, CKB, ADSS, DYNC1H1, NOS2, ATAD5, MOK, FMO1, FMO2, CDC42BPB, FMO3, FMO4, TUBB4B, TUBB4A, SEPT14, RAB11B, LOC490346, MYO1D, MYO1E, P2RX5, MYO1C, PDE10A, NMNAT1, TAOK1, KIF26B, P2RX1, MYO1F, NEK11	1.15
GO:0000166	nucleotide binding	99	0.08	DGKD, STKLD1, SMCHD1, RPS6KA2, AKT3, NUDT16, ACAD11, MAP3K4, MAP2K2, LOC100688619, DAPK3, MATK, UBE4B, KMO, RAB34, NCL, DUS3L, RABL6, H6PD, NLK, AK8, RHOT1, NDOR1, NLRP6, SAFB2, ABL2, MAP2K7, MARK3, DNAH12, ABCA2, YES1, XRCC3, MYO5A, TRPV1, ERAL1, EEF2, HNRNPM, CPS1, CDK10, CDK13, ITM2C, ATP2A3, CELF5, ELAVL1, CHD1, ACADL, GSG2, MYO18A, PRKG2, KIF1B, HSP90AA1, WARS, RFC1, KSR1, SIRT6, SAFB, DCK, SIRT3, APRT, DNM3, UGDH, SLTM, TCP1, NCBP3, MVD, RALA, ROCK1, SPG7, CAMKK1, GNA15, ERBB4, CREG1, CKB, ADSS, DYNC1H1, NOS2, ATAD5, MOK, FMO1, FMO2, CDC42BPB, FMO3, FMO4, TUBB4B, TUBB4A, SEPT14, RAB11B, LOC490346, MYO1D, MYO1E, P2RX5, MYO1C, PDE10A, NMNAT1, TAOK1, KIF26B, P2RX1, MYO1F, NEK11	1.15
GO:0017110	nucleoside-diphosphatase activity	3	0.09	ENTPD2, ENTPD8, NUDT16	5.93
GO:0005231	excitatory extracellular ligand-gated ion channel activity	6	0.09	CHRND, P2RX5, CHRNG, P2RX1, TRPV1, GRIN1	2.51
GO:0030345	structural constituent of tooth enamel	2	0.09	ENAM, AMBN	21.76
GO:0030021	extracellular matrix structural constituent conferring compression resistance	2	0.09	ENAM, AMBN	21.76
GO:0004553	hydrolase activity, hydrolyzing O-glycosyl compounds	8	0.09	NEU2, KLB, GALC, ACER1, LOC607002, SPACA3, MAN1A1, LOC480667	2.05

GO:0016705	oxidoreductase activity, acting on paired donors, with incorporation or reduction of molecular oxygen	11	0.10	ALKBH3, NOS2, FMO1, FMO2, DBH, DEGS2, DOHH, FMO3, KMO, FMO4, LOC490346	1.76

Supplementary Table S7. Summary of combined significant GO terms, KEGG pathways and involved genes for pairwise comparison of Bullmastiff vs each clade.

Function	Group	Group Genes
transcription corepressor binding	1	EHMT1|PHF12|RORA|SUZ12|ZBTB7A
ubiquitin protein ligase binding	2	ANAPC2|ARRDC1|ARRDC5|BAG5|BECN2|CRK|FHIT|MAP1LC3C|NLK|PACRG|PIAS4|PRDX6|PRKN|PRR5L|PSMD1|RALA|TCP1|TRAF2|TRAF3|TRAF6
aryl sulfotransferase activity	3	LOC482182|SULT1B1|SULT1D1
ATP-dependent peptidase activity	4	CLPP|LONP1|SPG7
brain morphogenesis	5	AKT3|CDH2|HESX1|PAFAH1B1|SLC6A4|WNT5A
protein kinase C signaling	6	DGKD|HTR2B|MAS1|SEZ6|WNT5A
regulation of protein targeting to membrane	7	CDK5R1|HRAS|INPP5K|MFF|MYO1C
regulation of protein localization to cilium	8	CCDC66|ENTR1|GAS8|INPP5E
mitochondrial matrix	9	ACADL|ALKBH7|CLPP|CPS1|ERAL1|GRSF1|LONP1|METTL4|MRPL18|MRPL3|MRPL41|MRPL54|MRPS2|PMPCA|POLDIP2|SARDH|SIRT3|SOD2|TEFM|TIMM44
Purine metabolism	10	ADCY10|ADSS2|AK8|APRT|DCK|ENTPD8|FHIT|LOC611724|NUDT16|PDE10A|PDE6D|PGM2
Glycerolipid metabolism	11	AGPAT2|AGPAT4|CEL|DGKD|GK2|LIPC|LPIN2
Thiamine metabolism	12	AK8|ALPI|LOC611724
PPAR signaling pathway	13	ACADL|ACSBG2|ANGPTL4|FABP7|GK2|LOC480641|LOC485024|PLIN4|PLIN5|RXRA
MicroRNAs in cancer	14	CRK|HRAS|MAP2K2|MIR1-2|MIR126|MIR129-2|MIR133A|MIR203|MIR210|MIR214|MIR34A|MIR423A|MIR451|MIR494|MIR7-3|NOTCH1|PIK3CD|ROCK1
mitochondrial transport	15	ALKBH7|DNLZ|MFF|MPC1|MPC2|MRPL18|PMPCA|PRKN|RHOT1|SLC25A29|SLC25A33|SPG7|TIMM22|TIMM44
blastoderm segmentation	16	SEMA3A|TASOR|TDRD5|WNT5A
organophosphate metabolic process	17	ABHD3|ACP3|ADCY10|ADSS2|AGPAT2|AGPAT4|AK8|ALDOC|APRT|CBFA2T3|CPS1|DCK|DGKD|ENTPD2|ENTPD8|FHIT|GIT1|GK2|H6PD|HTR2B|INPP5D|INPP5E|INPP5K|KMO|LOC100855552|LOC102151342|LOC611724|MPC2|MVD|NMNAT1|NOS2|NPPC|NUDT16|PID1|PIGC|PIGS|PIK3CD|PIP5K1C|PIPOX|PLA2G4A|PNPLA7|PRDX6|PROCA1|PTDSS2|RORA|RPE|SARM1|SHPK|SLC2A6|SLC4A4|SMPDL3A|SULT1B1|TALDO1|TYMS|VAV2|WDR81|ZBTB7A
cellular lipid metabolic process	18	ABHD15|ABHD3|ABO|ACAD11|ACADL|ACAT2|ACER1|ACSBG2|ACSF3|AGPAT2|AGPAT4|ALKBH7|ANAPC2|ANGPTL4|C3|CEL|CERS4|CPS1|CRK|CYP46A1|DEGS2|DGKD|DHRS13|DPEP1|FUT7|GALC|GBGT1|GC|GK2|GLT6D1|H6PD|HTR2B|INPP5D|INP

		P5E|INPP5K|LIAS|LIPC|LPIN2|MVD|NEU2|NPHP3|P2RX1|PAFAH1B1|PIGC|PIGS|PIK3CD|PIP5K1C|PLA2G4A|PLIN5|PNPLA7|PRDX6|PROCA1|PTDSS2|PTGDS|QKI|RORA|SMPDL3A|SOAT1|ST8SIA4|SULT1B1|TRPV1|VAV2|WDR81
negative regulation by host of viral genome replication	19	CARD9|CCL8|CCNK|CHD1|IGF2R|INPP5K|NLRP6|PLG|PRKN
regulation of cell adhesion molecule production	20	ANAPC2|COLEC12|FUT5|FUT7|NOTCH1
pyrimidine-containing compound transmembrane transport	21	LOC486150|LOC486151|SLC22A2|SLC25A29|SLC25A33
attachment of mitotic spindle microtubules to kinetochore	22	BECN2|CDT1|DYNC1H1|HNRNPU|NDC80
regulation of mRNA processing	23	CELF5|DIS3L2|GIGYF2|HNRNPM|HNRNPU|METTL16|NCL|NSRP1|PNLDC1|PRDX6|PRR5L|QKI|SAFB|SAFB2|SLTM|SUPT6H|TRAF2|WTAP|YTHDC1|ZBTB7A|ZC3H14
negative regulation of viral entry into host cell	24	FCN2|LOC475935|LOC483397
response to pH	25	ACER1|ASIC2|GPR31|GPR65|RAB11B|TRPV1
phosphotyrosine residue binding	26	ABL2|CRK|IGF2R|PAFAH1B1|SAG|SH2D3A|SHD|TRPV1|VAV1|VAV2|YES1
Longevity regulating pathway	27	AKT3|APPL1|CAB39|CCNB2|CREB3L3|EEF2|EHMT1|EIF4E2|ELAVL1|FASLG|HRAS|MAP2K2|NLK|PIK3CD|PPP2R5C|RAB11B|RAG2|S1PR4|SOD2|TBC1D1|TSC1
negative regulation of axon extension	28	BAG5|CDK5R1|CRK|FOXB1|GLI3|HRAS|ITM2C|LOC607207|LRRC4C|NGEF|PAFAH1B1|PIK3CD|PTPRS|ROCK1|RTN4RL1|SEMA3A|SEMA3D|SEMA3E|SEMA6B|SSH2|TSC1|WNT5A
toll-like receptor signaling pathway	29	APPL1|CACTIN|COLEC12|FCN2|GPR108|NLRP6|PTPRS|RNF135|SARM1|TICAM1|TLR1|TLR10|TLR6|TRAF3
regulation of DNA-templated transcription, elongation	30	CCNK|CDK13|GTF2F1|HNRNPU|NELFB|SUPT6H|TEFM|THOC1
ceramide metabolic process	31	ACER1|ANAPC2|CEL|CERS4|DEGS2|FUT7|GALC|GBGT1|NEU2|P2RX1|SMPDL3A|ST8SIA4
Pentose phosphate pathway	32	ALDOC|H6PD|PGM2|RPE|SHPK|TALDO1
Alzheimer disease	33	ADAM10|AKT3|ATP2A3|BECN2|C8G|CACNA1B|CDK5R1|CREB3L3|CYBA|DNAH12|FASLG|GRIN1|HRAS|KLC1|LOC102151342|LOC102152879|MAP2K2|MAP2K7|NDUFA11|NDUFA7|NOS2|NOTCH1|PIK3CD|PRKN|PSMA8|PSMD1|PSMD11|PSMD13|RCOR1|RORA|SOD2|TAF4B|TRAF2|TUBB4A|TUBB4B|WNT5A
monoamine transmembrane transporter activity	34	ADAM10|ANTXR2|CCT6B|P2RX1|SLC22A2|SLC22A3|SLC25A29|SLC25A47|SLC6A4|TCP1
regulation of secondary metabolic process	35	APPL1|CTNS|MFSD12|OPN3|WNT5A
Chemokine signaling pathway	36	ABR|ACKR4|ADAM10|ADAP2|AKT3|ANAPC2|ARAP2|CCL1|CCL13|CCL20|CCL25|CCL7|CCL8|CCR6|CRK|EVI5L|FUT7|GNB5|GNG10|GPR65|HRAS|INPP5D|MAP2K2|NF1|NGEF|ODAM|PAFAH1B1|PIK3CD|RAP1GAP2|RASAL2|ROCK1|SGSM2|TAX1BP3|TBC1D1|TNFSF14|TSC1|TTC8|VAV1|VAV2|WNT5A

positive regulation of GTPase activity	37	ABR|ADAP2|AGFG1|AKT3|ARAP2|ARHGEF3|ATCAY|BAG5|BNIP2|CCL1|CCL13|CCL20|CCL25|CCL7|CCL8|CCR6|CRK|DENND1C|DNAH12|DNM3|DYNC1H1|EEF2|ENTPD2|ENTPD8|EPS8L2|EVI5L|FHIT|GIT1|GNA15|GNB5|GNG10|GPR65|GPSM1|HRAS|HTR2B|KIF1B|KIF25|KIF26A|KIF26B|MARK3|MYO18A|MYO1C|MYO1D|MYO1E|MYO1F|NF1|NGEF|NUDT16|ODAM|PAFAH1B1|PCP2|PDE6D|PIK3CD|RAB11B|RAB34|RALA|RALGDS|RALGPS2|RANBP3|RAP1GAP2|RASAL2|RASGEF1B|RHOT1|RIC8A|ROCK1|SEPTIN14|SGSM2|SH2D3A|TAX1BP3|TBC1D1|TCF25|TDRD9|TMEM250|TSC1|TSR1|TTC8|TUBB4A|TUBB4B|VAV1|VAV2|WNT5A
N,N-dimethylaniline monooxygenase activity	38	ACAD11|ACADL|AQP4|CYP46A1|DUS3L|ENSCAFG00000002857|ENSCAFG00000029232|ENSCAFG00000029376|FCER2|FMO1|FMO2|FMO3|FMO4|H6PD|HSD11B1L|HSD17B12|HTR2B|KMO|LOC482182|LOC490346|NDOR1|NOS2|RPE|RXRA|SLC4A4|UGDH|UGT1A6
Lipid and atherosclerosis	39	AKT3|C3|CCL20|CCL7|CD70|COL4A3|CYBA|ELAVL1|FASLG|FHIT|GNA15|HRAS|HSP90AA1|IL17C|MAP2K2|MAP2K7|MIB1|NOS2|PIK3CD|POU2F1|RXRA|SOD2|TICAM1|TLR1|TLR6|TNFSF14|TNFSF18|TNFSF4|TRAF2|TRAF3|TRAF6|VAV1|VAV2
lymphocyte migration	40	ACKR4|ADAM10|AKT3|ANAPC2|AQP4|BMP3|CACTIN|CCL1|CCL13|CCL20|CCL25|CCL7|CCL8|CCR6|CD70|CRK|DAPK3|EBI3|FASLG|FUT7|GNB5|GNG10|HRAS|IL17C|INHBA|INPP5K|LOC475935|LOC483397|MAP2K7|MYO1C|NOS2|PIAS4|PID1|PIK3CD|PRKN|ROCK1|RORA|SP100|TNFSF14|TNFSF18|TNFSF4|TRAF2|TRAF3|TRAF6|VAV1|VAV2|WNT5A
Fc epsilon RI signaling pathway	41	ABL2|ACER1|ADCY10|ADCYAP1|AFDN|AGPAT2|AGPAT4|AKT3|AMPH|ANXA2|APPL1|ATP2A3|C3|CACNA1B|CACNA2D3|CCL1|CCL13|CCL20|CCL25|CCL7|CCL8|CCNB2|CCR6|CERS4|COL4A3|CREB3L3|CRK|CYBA|DEGS2|DGKD|DNM3|DYNC1H1|EHMT1|EIF4E2|ERBB4|EVL|FASLG|FGF5|FHIT|GLI3|GNA15|GNB5|GNG10|GRIN1|HRAS|HSP90AA1|INPP5D|KLC1|KSR1|LOC489647|LOC607207|MAP2K2|MAP2K7|MAP3K4|MATK|MIB1|NF1|NLK|NOS2|PDE10A|PIK3CD|PIP5K1C|PLA2G4A|POU2F1|PPP2R5C|RAG2|RALA|RALGDS|RASAL2|RILP|ROCK1|RPS6KA2|RXRA|S1PR4|SLC22A2|SLC22A3|SOD2|TAOK1|TICAM1|TLR1|TLR6|TRAF2|TRAF3|TRAF6|TSC1|TUBB4A|TUBB4B|VAV1|VAV2|VPS41|YWHAE
